# d^1^ Imidate-umpolung addition to activated imines for the asymmetric synthesis of amino acid derivatives: a combined DFT and experimental study

**DOI:** 10.1039/d6ra07192a

**Published:** 2026-09-16

**Authors:** Arianna Quintavalla, Emanuèl Bruno Savini, Simone di Remigio, Marco Lombardo

**Affiliations:** a Department of Chemistry “Giacomo Ciamician”, Center for Chemical Catalysis – C3, Alma Mater Studiorum – University of Bologna via P. Gobetti 85 I-40129 Bologna Italy marco.lombardo@unibo.it arianna.quintavalla@unibo.it; b Consorzio C.I.N.M.P.I.S. (National Interuniversity Research Consortium in Innovative Synthesis Methodologies and Processes) c/o Alma Mater Studiorum – University of Bologna via P. Gobetti 85 I-40129 Bologna Italy

## Abstract

Herein, we report the asymmetric synthesis of highly enantioenriched amino acid derivatives exploiting a lithiated Ellman-derived imidate as a nucleophilic d^1^ umpolung synthon. While traditional d^1^ strategies focus on aldehyde-derived acyl anions, this reagent acts as a formal ester anion equivalent. While unactivated imines fail to react, the addition to non-enolizable Ellman sulfinimines—including aromatic and α,β-unsaturated derivatives—proceeds in good yields and with excellent stereocontrol enabled by double stereodifferentiation. The matched stereoisomeric combination provides the corresponding adducts in good chemical yields (up to 96%) and almost complete diastereoselectivity (dr >99 : 1), while the mismatched pair leads to complex mixtures and much lower conversions. A comprehensive Density Functional Theory (DFT) study revealed the structural origins of both the substrate scope and the absolute stereocontrol, while also identifying a low-barrier self-condensation pathway that rationalizes the strict requirement for an *in situ* one-pot protocol to suppress nucleophile decomposition.

## Abstract 2

Lithiated Ellman-derived imidates act as formal ester anion equivalents in asymmetric d^1^ umpolung additions, enabling robust access to α-amino acid derivatives with up to 96% yield and excellent diastereocontrol (dr >99 : 1).

## Graphical abstract

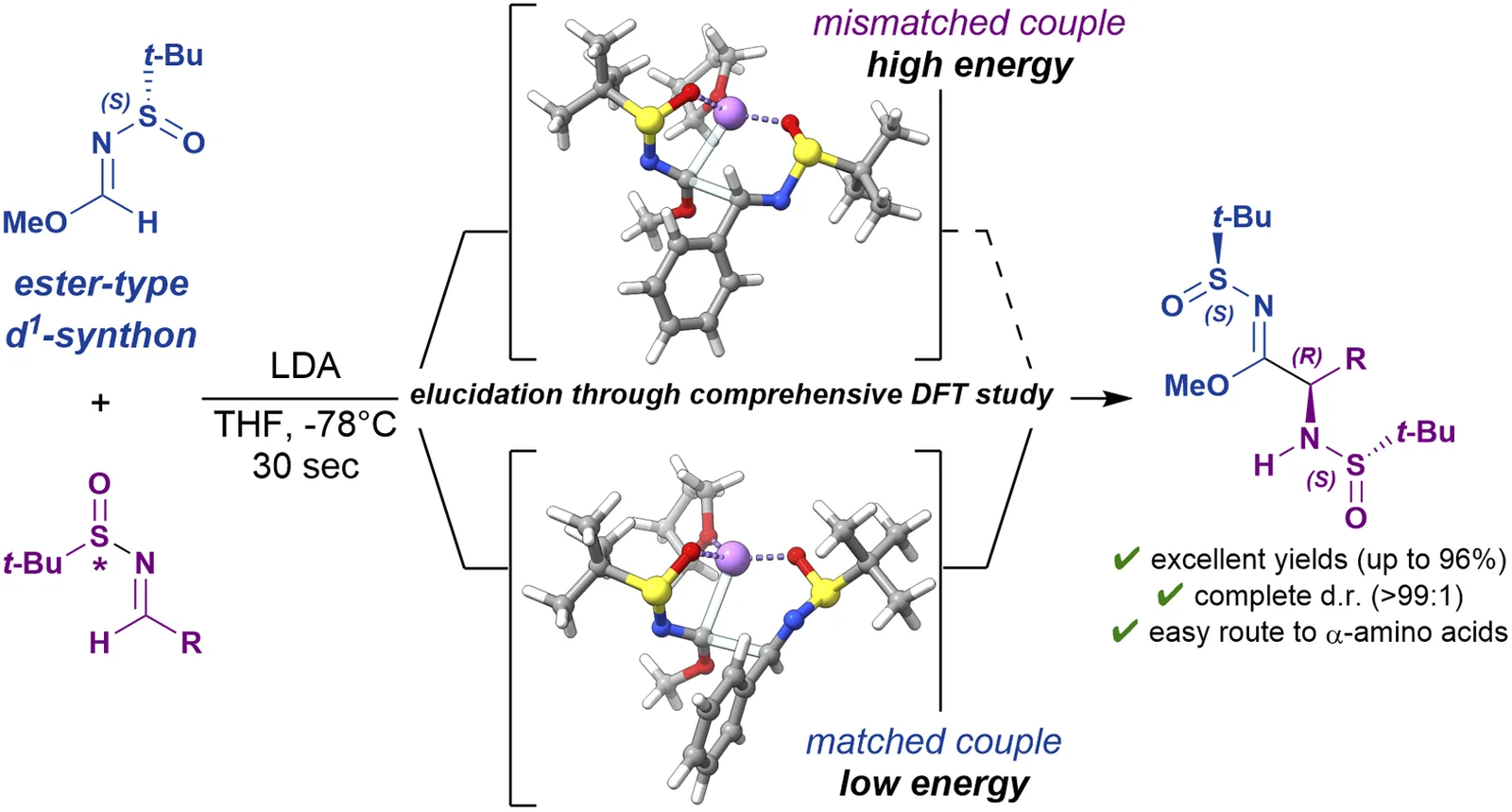

## Introduction

The construction of stereodefined carbon–carbon bonds remains a cornerstone of modern synthetic organic chemistry. In this context, carbonyl transformations have classically relied on the natural polarization of the functional group, where the carbonyl carbon exhibits an electrophilic acceptor character (a^1^). This innate reactivity enables fundamental reaction pathways like nucleophilic additions and aldol-type transformations, but it inherently restricts the functionalization pattern to odd-numbered distances (functionality span). To address these limitations, the conceptual framework of reactivity umpolung (inversion of polarity) – pioneered by Dieter Seebach and Elias J. Corey – has been established as a powerful strategy.^1,2^ Specifically, traditional d^1^ umpolung promotes the inversion of the a^1^ electrophilic center of an aldehyde into a formal acyl anion equivalent, turning the carbon into a nucleophilic donor. This strategy commonly requires the temporary installation of a masking group capable of stabilizing a negative charge on the former carbonyl carbon. Over the past decades, several robust methodologies have been established to prepare reliable d^1^ synthons (Fig. 1A). The most classic approach relies on the Corey–Seebach reaction, which converts aldehydes into 1,3-dithianes.^2,3^

**Fig. 1:**
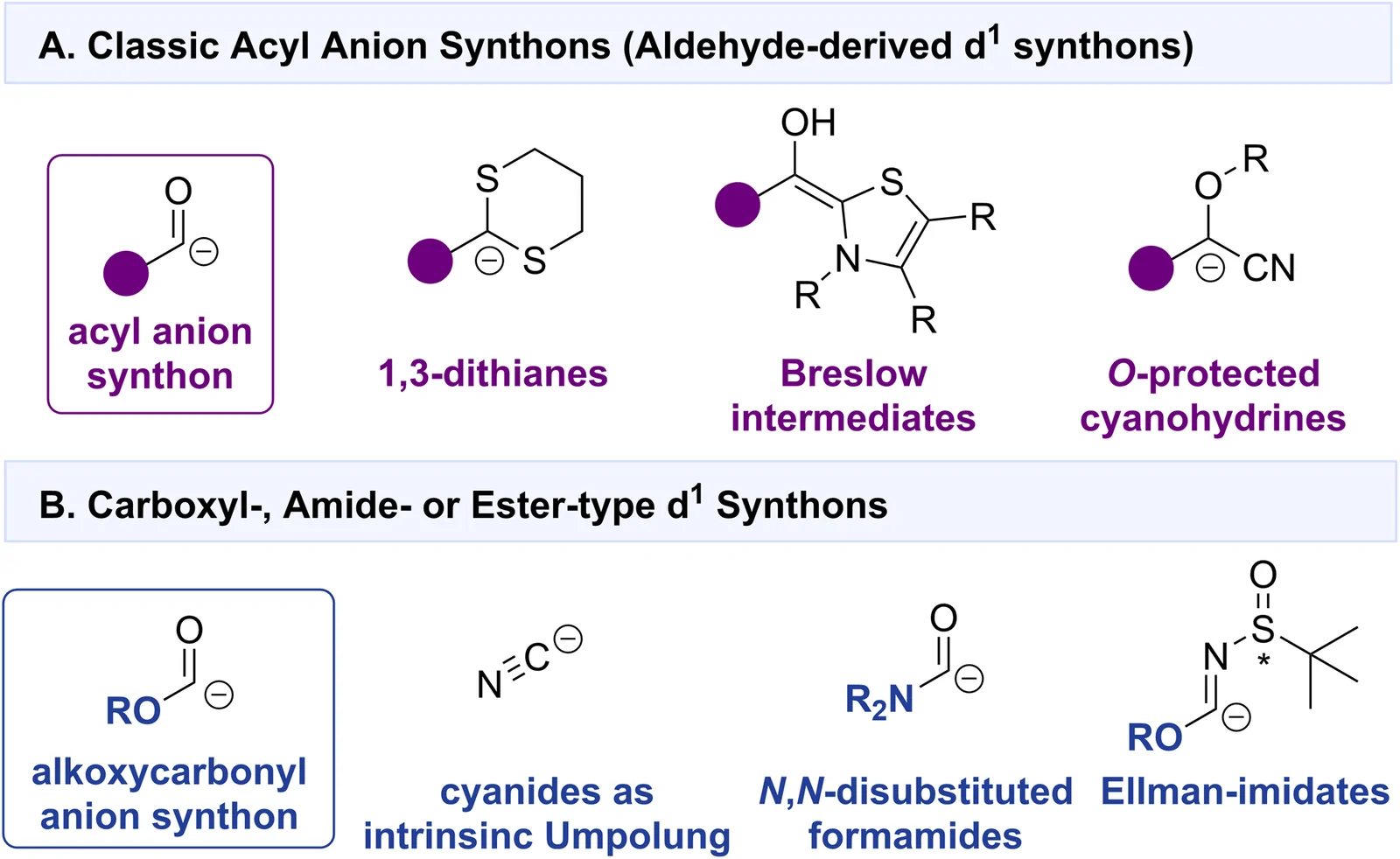
d^1^ Umpolung: (A) classic aldehyde-derived acyl anions *versus* (B) carboxyl-, amide- or ester-type equivalents.

The high polarizability and σ*-stabilizing effects of the adjacent sulfur atoms significantly lower the p*K*_a_ of the formyl proton, allowing clean deprotonation by strong organolithium bases to give a highly nucleophilic carbanion capable of attacking different electrophiles.^4^ However, the method suffers from low atom economy and, more critically, the harsh conditions required for the final deprotection step – typically employing toxic mercury(ii) salts or strong oxidizing agents – which significantly limit functional group tolerance and late-stage functionalization.^4^ To find milder and more atom-economic conditions, N-heterocyclic carbene (NHC) organocatalysis was subsequently introduced.^5,6^ Indeed, the nucleophilic addition of an NHC to an aldehyde generates a transient Breslow intermediate that allows easy access to d^1^ coupling reactions,^7^ such as the benzoin condensation and the Stetter reaction, without the need for stoichiometric auxiliary groups or harsh basic conditions. Despite its mild operational conditions, NHC catalysis is frequently hampered by narrow substrate scopes, low tolerance of the catalyst to air and moisture, and the costly procedures required to access stereoselective NHC catalysts.^8^ Finally, protected cyanohydrins – pioneered by Gilbert Stork – offer a chemically orthogonal route to stable d^1^ synthons.^9^

While the cyanide anion inherently serves as a classical synthetic equivalent for a carboxyl or ester d^1^ anion in Strecker reactions (Fig. 1B),^10^ its implementation relies on highly toxic reagents, posing significant safety and environmental concerns.^11^ Furthermore, despite their utility, the development of general, highly stereoselective protocols using cyanides or protected cyanohydrin anions remains limited.^12^ While these classical methodologies are heavily tailored toward aldehyde-derived acyl anion equivalents – or rely on toxic cyanide salts to access carboxyl-type derivatives – expanding the d^1^ umpolung framework to non-toxic, structurally versatile carboxylic acid derivatives remains significantly more challenging.

In this context, carbamoyl anions generated from formamides have emerged as powerful tools for the direct introduction of amide functionality. Reeves and co-workers pioneered the first asymmetric addition of carbamoyllithium species (Fig. 1B) to chiral *N*-sulfinyl imines, providing an elegant and reliable route to enantiopure α-amino amides.^13^ In this approach, the carbamoyllithium species exhibits sufficient stability to be pre-generated prior to electrophile addition, even enabling reactions with enolizable imines. While this methodology represents a milestone for accessing α-amino amide architectures, achieving high stereocontrol frequently relies on specific steric combinations, such as the use of bulky diisopropylcarbamoyl derivatives and *N*-TIPPS sulfinimines (TIPPS = *N*-2,4,6-triisopropylphenylsulfonyl). More recently, Prasad and co-workers reported the addition of lithio tris(methylthio)methane to Ellman sulfinimines as a versatile d^1^ synthon for the stereoselective preparation of α-amino acid derivatives, including esters, thioesters, amides, and peptide fragments.^14^ Despite these important advances, the conversion of the resulting sulfur-stabilized intermediates into the desired carbonyl derivatives requires oxidative demasking conditions, including stoichiometric silver salts, highlighting the need for complementary ester-type d^1^ reagents that directly deliver synthetically versatile amino ester frameworks.

Consequently, developing a complementary strategy that employs more readily modifiable ester-type d^1^ synthons (alkoxycarbonyl anions) remains highly desirable, as it would grant direct access to versatile amino ester frameworks.

In 2016, Xu and Lu addressed this problem by introducing a promising ester-type d^1^ synthon,^15^ obtained by the metallation of an Ellman-derived imidate^16^ with lithium diisopropylamide (LDA) at −78 °C (Fig. 1B). This intermediate was successfully exploited in a one-pot addition to non-enolizable aldehydes, yielding α-hydroxyimidates in good yields but with moderate diastereoselectivity (dr = 8 : 2), which were subsequently used for further synthetic elaborations. Crucially, unlike their carbamoyl counterparts, this protocol required a strict *in situ* one-pot procedure involving the simultaneous mixing of the imidate, the aldehyde, and LDA. Moreover, despite the synthetic potential of this ester anion equivalent, a detailed mechanistic investigation rationalizing its unique reactivity and limitations was not reported, and its application toward nitrogen-based electrophiles for amino acid synthesis remained completely unexplored. Herein, we present a comprehensive computational and experimental study that addresses these limitations, establishing a highly stereoselective d^1^ umpolung strategy for the synthesis of enantiopure amino acid derivatives. A rigorous density functional theory (DFT) study allowed us to elucidate that competitive self-condensation of the metallated imidate represents the primary, highly favoured decomposition pathway, rationalizing why an *in situ* one-pot protocol is strictly required to successfully intercept the transient nucleophilic species. Furthermore, our DFT models demonstrated that the reaction is highly sensitive to the electrophilic nature of the substrate: while non-enolizable aldehydes possess sufficient electrophilicity to outcompete decomposition, simple unactivated imine counterparts completely fail to react. To overcome this limitation, we successfully employed non-enolizable Ellman sulfinimines as activated, chiral electrophiles. Computational analysis, fully supported by experimental results, revealed that the reaction outcome is governed by a double stereodifferentiation effect. While the mismatched combination is characterized by a higher activation barrier that directly competes with the self-condensation pathway – resulting in poor yields – the matched stereoisomeric combination provides the corresponding adducts not only in excellent yields (up to 96%) but also with virtually complete diastereocontrol (dr >99 : 1).

## Results and discussion

We started our investigation by modelling the ground-state conformational behavior of (*S*)-imidate 1 and its subsequent deprotonation by lithium diisopropylamide (LDA, Fig. 2). Calculations were performed with Orca 6.0,^17^ optimizing geometries at the ωB97X-D3/def2-TZVP level of theory, followed by single-point energy evaluations using the double-hybrid ωB97M(2)/def2-TZVP functional, implicitly modelling the THF environment with the SMD continuum solvation model. Starting imidate 1 preferentially adopts a s-*cis* conformation (Fig. 2A), in rapid equilibrium (Δ*G*^‡^ = 2.7 kcal mol^−1^) with the slightly less stable s-*trans* conformer (Δ*G* = 1.7 kcal mol^−1^). Only the s-*cis* conformer enables the effective chelation of the lithium cation in a geometry allowing the sulfoxide moiety to act as a directing group, guiding the base in the optimal spatial arrangement required for the abstraction of the imidate C1–H. Thanks to the low rotational barrier, a rapid dynamic equilibrium is maintained, ensuring complete and efficient equilibration toward the reactive s-*cis* pathway. Next, we turned our attention to the metallation step. While LDA is known to predominantly exist as a dimeric aggregate in THF solution, pioneering studies by Collum and co-workers^18^ demonstrated that the presence of catalytic amounts of LiCl—generated during the *in situ* preparation of LDA from *n*-BuLi and diisopropylamine—drastically accelerates deaggregation.

**Fig. 2:**
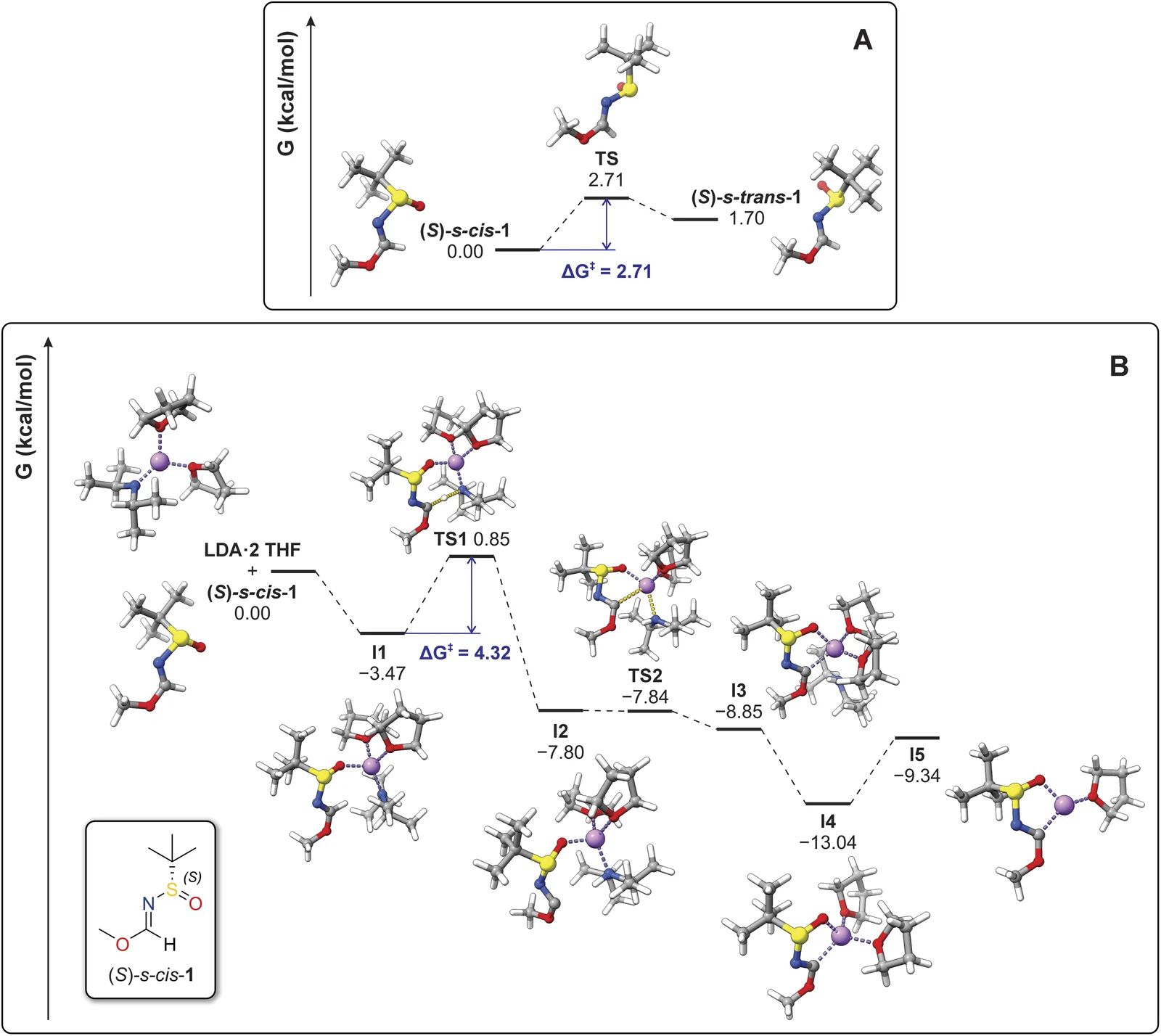
Calculated free energy profile (Δ*G*, kcal mol^−1^) for (A) s-*cis*-1/s-*trans*-1 conformational equilibrium and (B) lithiation cascade of (*S*)-s-*cis*-imidate 1 at −78 °C. All geometries were optimized at the ωB97X-D3/def2-TZVP level of theory, with single-point energies evaluated using the double-hybrid ωB97M(2)/def2-TZVP functional and the SMD continuum solvation model for THF. Transition states are denoted as TS, while stable intermediates as I.

Under these conditions, the monomeric LDA·2THF complex emerged as a key, highly reactive metallating species. Consequently, the deprotonation protocol of s-*cis*-1 was modelled utilizing monomeric species as the active base (Fig. 2B). Starting from the isolated reagents—specifically, the (*S*)-s-*cis*-1 conformer and the LDA·2THF complex—defined as the energy reference point (Δ*G* = 0.0 kcal mol^−1^), their initial association leads to the exergonic formation of pre-complex I1 (Δ*G* = −3.5 kcal mol^−1^), which subsequently undergoes deprotonation *via* transition state TS1 with an absolute free energy of only 0.85 kcal mol^−1^ (corresponding to an activation barrier of Δ*G*^‡^ = 4.3 kcal mol^−1^ from I1). This low-barrier event affords intermediate I2 (Δ*G* = −7.8 kcal mol^−1^), wherein the lithium cation is not yet bonded to the nucleophilic carbon C1 but remains chelated by the sulfoxide oxygen (S=O) and the nitrogen center of the newly formed diisopropylamine (DIPA). From I2, a virtually barrierless structural reorganization *via*TS2 (Δ*G* = −7.84 kcal mol^−1^) establishes the key carbon–lithium bond, producing intermediate I3 (Δ*G* = −8.9 kcal mol^−1^) where DIPA is displaced from the inner coordination sphere but remains weakly associated within the cluster. Finally, the complete extrusion of DIPA affords the active, stable lithiated nucleophile I4 (Δ*G* = −13.0 kcal mol^−1^), which retains a tetrahedral coordination environment around the lithium centre, completed by two explicit THF molecules. This overall exergonic metalation cascade perfectly rationalizes the rapid and clean generation of the reactive d^1^ synthon. Indeed, this exceptionally low metalation activation barrier is in striking agreement with experimental observations, as the *in situ* one-pot protocol proceeds to completion within a few minutes not only at −78 °C, but also under extreme cryogenic conditions at −116 °C (Trapp mixture). To engage in the subsequent nucleophilic addition I4 must undergo a coordination rearrangement. As reported by Collum,^19^ organolithium resting states are frequently highly solvated, whereas the actual reactive pathways are accessed through higher-energy, coordinatively unsaturated species in dynamic equilibrium (*e.g.*, the desolvation of LDA·3THF to reactive LDA·2THF complex). Analogously, I4 must lose a molecule of THF to generate the active intermediate I5 (Δ*G* = −9.3 kcal mol^−1^), providing the necessary Lewis-acidic site to bind the incoming electrophile, enabling the subsequent addition. The reactivity and chemoselectivity of the lithiated imidate I5 were thus evaluated against different electrophilic partners (Fig. 3A). At cryogenic temperatures, I5 is prone to a competitive, degradative self-condensation pathway. To establish the kinetic boundary of this decomposition, variable-time quench experiments were conducted at −78 °C and −116 °C, adding benzaldehyde as the electrophile after different timeframes (see SI for full experimental details). While the conversion of the starting imidate 1 was always in the 75–80% range, the yield of the desired addition product was 38% if benzaldehyde was added after 10 seconds at −78 °C, dropping to only 7% if addition is made after 30 seconds. The reagents half-life slightly extended at −116 °C, with yields of 48 and 44% after 30 seconds and 15 minutes, respectively. Applying the second-order Eyring–Polanyi relationship incorporating half-life values and concentration values, the experimental activation energy for this degradative pathway was quantified within a narrow range of 11.4–13.0 kcal mol^−1^ at −78 °C and 11.1–12.7 kcal mol^−1^ at −116 °C. Our DFT calculations identified the self-condensation profile of I5 as the operative degradative manifold, with its lowest-energy transition state located at 12.7 kcal mol^−1^, in excellent agreement with the experimental kinetic data (Fig. 3B). We also compared the computed activation energies of the lowest productive transition states for benzaldehyde 2 and the simple unactivated (*E*)-*N-tert*-butyl-1-phenylmethanimine 4, against the self-condensation TS energy taken as the boundary limit for reactivity (Fig. 3).

**Fig. 3:**
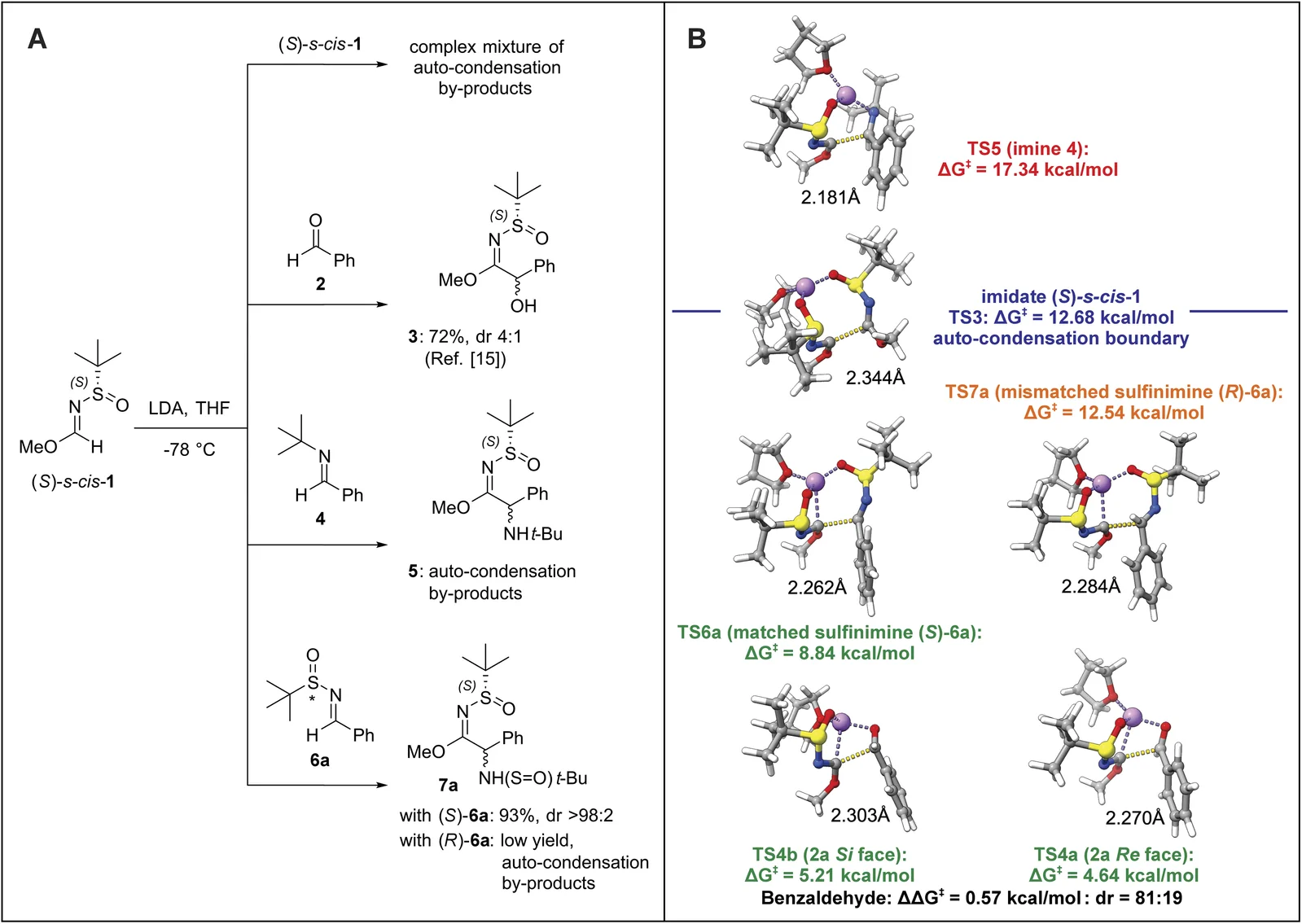
(A) Addition reactions of different electrophiles to imidate (*S*)-s-*cis*-1 (see SI for details); (B) computed absolute free energies of activation (Δ*G*^‡^, kcal mol^−1^) at 195.15 K for competing pathways of nucleophile I5 with different electrophiles.

For the benchmark reaction with benzaldehyde, the lowest barrier is located at 4.6 kcal mol^−1^. The net free-energy gap between this productive path and the degradative pathway (ΔΔG^‡^ = −8.0 kcal mol^−1^) ensures an overwhelming kinetic advantage, allowing the one-pot cross-coupling to proceed rapidly before any significant self-condensation can take place.

Significantly, the calculated dr ratio perfectly matched the experimentally reported result (dr 80 : 20). In sharp contrast, when I5 is exposed to the unactivated imine 4, the calculated nucleophilic addition barrier increased to 17.3 kcal mol^−1^. Because this activation peak is nearly +4.7 kcal mol^−1^ higher than the self-condensation bottleneck, the bimolecular cross-coupling is completely inactive and under these conditions, I5 is exclusively consumed by the rapid background self-condensation pathway. Attempts to pre-activate the unactivated imine 4 with Lewis acid additives (*e.g.*, BF_3_·OEt_2_) yielded only trace amounts of the desired product. This lack of reactivity is presumably due to competitive coordination of the Lewis acid with the THF solvent and the imidate itself upon its addition, despite the rapid lithiation by LDA at −78 °C. This observation highlights a fundamental distinction between ester-type d^1^ synthons and the more established acyl anion equivalents, whose stability often allows their generation and isolation prior to electrophile addition. In contrast, the lithiated imidate I5 behaves as a highly reactive but transient nucleophile, and its synthetic utility is therefore governed not only by intrinsic nucleophilicity but also by the relative kinetics of productive electrophile capture and self-decomposition. The identification of this kinetic boundary provides a general framework for evaluating and further improving ester-based umpolung reagents.

Having established that productive electrophiles must react within a narrow kinetic window defined by the self-condensation barrier, we next sought activated imines capable of satisfying this requirement. Ellman sulfinimines appeared ideally suited for this purpose, as the electron-withdrawing sulfinyl auxiliary simultaneously enhances the electrophilicity of the C=N bond while providing a well-established source of asymmetric induction.^20^ Their versatility as chiral electrophilic platforms has been demonstrated in a variety of stereoselective nucleophilic additions.^21,22^ Gratifyingly, reaction of the lithiated imidate (*S*)-I5 with matched (*S*)-benzaldehyde-derived sulfinimine 6a proceeded smoothly to afford amino-imidate 7a in 56% isolated yield after only 30 s, with virtually complete diastereocontrol (dr >99 : 1). In contrast, the corresponding mismatched sulfinimine (*R*)-6a furnished only 17% yield together with a substantially lower diastereomeric ratio (dr 65 : 35). DFT calculations accurately reproduced both the observed stereochemical outcome and the pronounced matched/mismatched effect (Fig. 3B). Structurally, the calculated transition states reveal that the lithium cation organizes a well-defined, multidentate coordination sphere, interacting simultaneously with the imidate C1 carbon, the S=O bond of the imidate's Ellman auxiliary, a molecule of THF, and the S=O group of the imine's Ellman auxiliary, effectively bridging the two reaction partners in a highly organized, templated assembly. DFT analysis further revealed that the origin of the exceptional stereocontrol does not arise solely from the energy difference between the competing transition states. Interestingly, the matched and mismatched pathways operate under different kinetic regimes. In the matched combination, although conformational interconversion of the imine–lithium complexes remains accessible, the conformational pre-equilibrium is not sufficiently rapid to fulfil the Curtin–Hammett condition. The productive addition pathway therefore competes with conformational equilibration, and the highly reactive s-*trans*-derived transition state acts as a kinetic funnel, selectively consuming the corresponding reactive complex and directing the reaction toward the experimentally observed stereoisomer. Conversely, the mismatched system operates under a classical Curtin–Hammett regime, where rapid pre-equilibration of the reactive conformational ensemble leads to competing transition states of comparable energy, accounting for the poor diastereoselectivity observed experimentally. Moreover, in the matched combination, the kinetically favoured s-*trans* pathway lies below the self-condensation threshold (ΔΔG^‡^ = −3.8 kcal mol^−1^), enabling productive C–C bond formation before significant nucleophile degradation occurs. Conversely, in the mismatched combination, the competing pathways become energetically comparable to the self-condensation manifold (ΔΔG^‡^ = −0.14 kcal mol^−1^), explaining both the reduced conversion and the erosion of stereocontrol observed experimentally. Encouraged by these results, we optimized the reaction conditions (see SI), ultimately increasing the isolated yield of 7a to 93% while maintaining essentially a complete stereocontrol (dr >99 : 1).

With optimized conditions established, the generality of the transformation was investigated using a range of non-enolizable sulfinimines, including aromatic, α,β-unsaturated, and *tert*-butyl derivatives (Scheme 1).

**Scheme 1:**
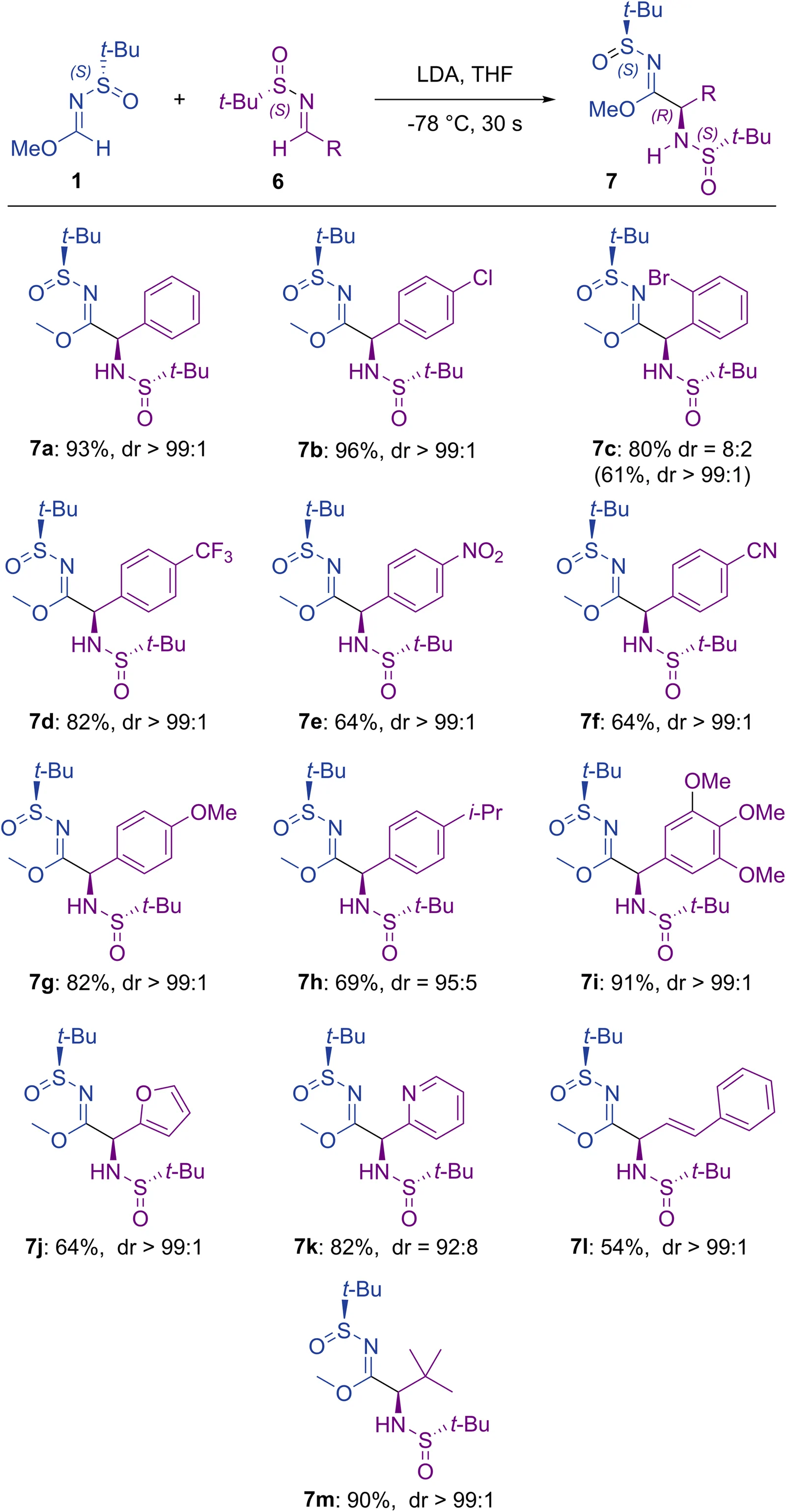
Substrate scope.

Overall, the reaction proved remarkably tolerant toward electronically diverse aromatic sulfinimines. The broad substrate scope indicates that the sulfinyl auxiliary provides sufficient electrophilic activation for productive addition to outcompete self-condensation across a wide range of aromatic imines. Nevertheless, the observed variation in isolated yields indicates that electronic and steric factors still modulate the relative rate of productive C–C bond formation.

Halogen-substituted derivatives were well tolerated, with the 4-chloro derivative reacting with remarkable efficiency and affording the corresponding product in 96% yield (7b), indicating that moderate electronic withdrawal at the aromatic ring does not compromise productive C–C bond formation. In contrast, introduction of a bromine substituent at the 2 position resulted in a decreased yield and dr (7c, 80%, 8 : 2), suggesting that steric congestion around the imine electrophile influences the efficiency of the addition. The major diastereoisomer was isolated in 61% yield and >99 : 1 dr after purification.

Strongly electron-deficient sulfinimines bearing CF_3_, NO_2_ and CN substituents were also tolerated (7d–f), affording the corresponding products in 64–82% yield.

Although these substituents are expected to enhance the electrophilicity of the imine carbon, no direct correlation between electron-withdrawing ability and isolated yield was observed, suggesting that once the electrophilic threshold required to compete with self-condensation is reached, additional electronic activation provides only a limited benefit.

Electron-rich sulfinimines also performed efficiently. The anisyl (7g) and trimethoxy (7i) derivatives afforded the corresponding products in 82% and 91% yield, respectively, while the bulkier 4-isopropyl analogue (7h) gave 69% yield with a slightly reduced diastereoselectivity (dr = 95 : 5)

The methodology was further extended to heteroaromatic sulfinimines. Both furyl- (7j) and pyridyl- (7k) substituted substrates were successfully converted into the corresponding products, affording 64% and 82% yield, respectively. The efficient conversion of the pyridyl derivative is particularly noteworthy, considering the potential for competitive lithium coordination by the heteroaromatic nitrogen, which likely decreased the dr to 92 : 8. Finally, the cinnamaldehyde-derived α,β-unsaturated sulfinimine 7l was also competent, furnishing the desired conjugated amino imidate in 54% yield. This result highlights the ability of the lithiated imidate to selectively engage activated imines even in the presence of an extended π-system.

To further probe the steric and electronic boundaries of this protocol, we also evaluated two non-enolizable aliphatic derivatives. Gratifyingly, the sterically congested pivaldehyde-derived sulfinimine successfully afforded the corresponding amino imidate 7m (Scheme 1) in an excellent 90% isolated yield and with virtually complete stereocontrol (dr >99 : 1). In sharp contrast, attempts to extend the reaction to trifluoroacetaldehyde-derived sulfinimine failed to give the desired product, yielding only degradation products. This lack of reactivity is likely due to competitive β-fluorine elimination under the basic reaction conditions, completely preventing the productive nucleophilic addition.

Overall, these results demonstrate that the sulfinyl auxiliary provides sufficient activation of the imine electrophile to enable productive C–C bond formation across a wide range of substitution patterns, although subtle electronic and steric effects still modulate the reaction efficiency.

To unambiguously establish the absolute configuration of the newly formed stereogenic center, the (*R*,*R*)-phenyl-substituted adduct 7a was subjected to auxiliary removal under standard acidic conditions (MeOH, HCl), efficiently converting the imidate intermediate into the corresponding methyl phenylglycinate derivative 8a, which was directly compared with commercially available (*R*)- and (*S*)-phenylglycine methyl esters. The measured optical rotation of the synthetic amino ester was consistent with that reported for the (*S*) enantiomer, confirming the stereochemical assignment obtained from the matched sulfinimine addition (see SI) (Scheme 2).

**Scheme 2:**
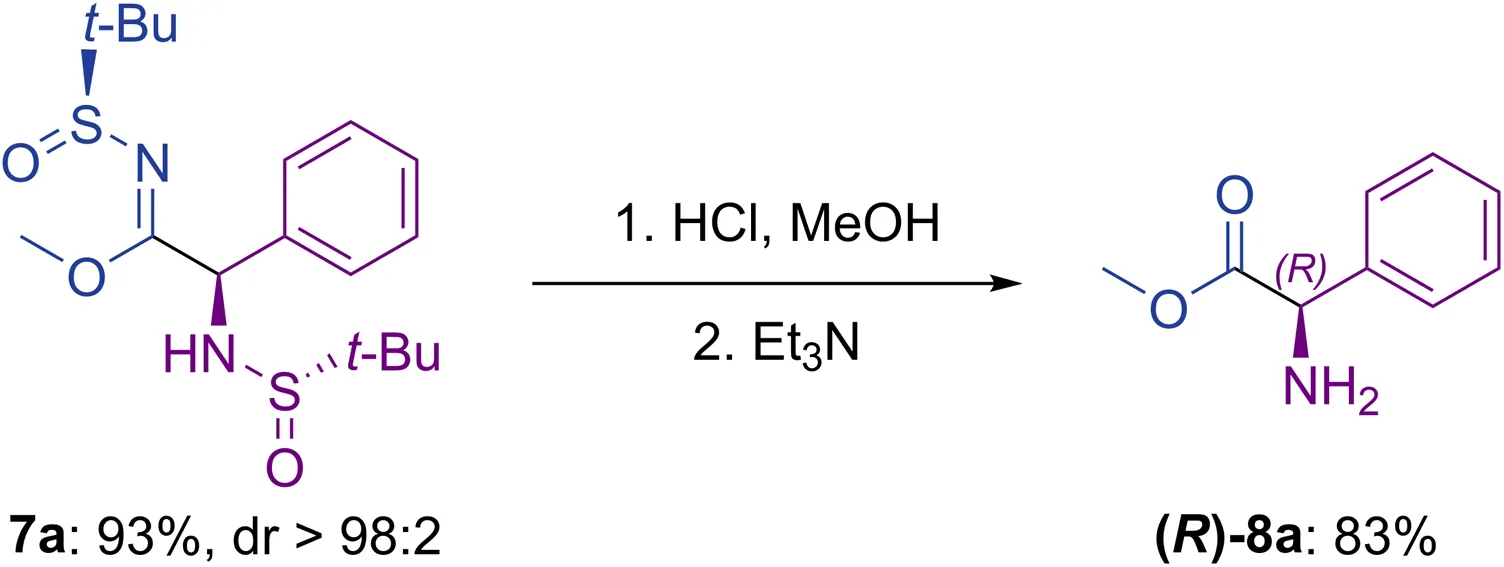
Stereochemical assignment: hydrolysis of imidate 7a to methyl phenylglycinate.

This experimentally established stereochemical assignment was further supported by DFT analysis of the competing imine conformational pathways. Indeed, sulfinimine 6a exists as two accessible s-*cis* and s-*trans* conformers, which lead to distinct transition-state pathways. The lowest-energy transition state leading to the experimentally observed (*R*) configuration was found to originate from the s-*trans* conformer. This pathway is favoured over the corresponding s-*cis*-derived transition state by 1.8 kcal mol^−1^, corresponding to a predicted diastereomeric ratio of approximately 99 : 1 at −78 °C, in excellent agreement with the experimental outcome (see SI).

## Conclusions

In conclusion, we have developed a highly stereoselective d^1^ umpolung strategy for the synthesis of enantiopure α-amino acid derivatives bearing aryl and heteroaryl substituents through the addition of a lithiated Ellman-derived imidate to sulfinyl-activated imines. This methodology provides direct access to ester-type nucleophilic synthons under mild reaction conditions. A combined experimental and computational investigation revealed that the intrinsic limitation of this reagent originates from a rapid self-condensation pathway, which defines a narrow kinetic window for productive electrophile trapping. DFT analysis was crucial not only for rationalizing this reactivity boundary, but also for identifying the mechanistic origin of the substrate dependence: while unactivated imines fail because their addition pathway cannot compete with nucleophile decomposition, Ellman sulfinimines provide sufficient electrophilic activation to enable efficient interception of the transient lithiated intermediate. Furthermore, the computational analysis revealed that the exceptional stereocontrol arises from a double stereodifferentiation effect operating through distinct kinetic regimes for matched and mismatched sulfinimine configurations. This model accurately predicts both the experimentally observed diastereoselectivity and the absolute configuration of the newly formed stereocenter. The identification of self-condensation as the intrinsic limitation of this d^1^ synthon provides a mechanistic rationale for the future optimization of ester-type umpolung reagents. More broadly, this study highlights that the successful implementation of transient d^1^ synthons depends on controlling the kinetic balance between productive electrophile trapping and intrinsic decomposition pathways. This mechanistic perspective provides a general design principle for the development of next-generation umpolung reagents with improved stability and broader synthetic applicability. Rather than relying solely on increasing nucleophilicity, future reagent design should consider structural strategies aimed at controlling aggregation, lithium coordination, or intermediate lifetime to favour productive reactivity over competing decomposition pathways.

## Author contributions

EBS & SDR: investigation, data acquisition, review and editing; ML & AQ: conceptualization, funding acquisition, supervision, writing and editing.

## Conflicts of interest

There are no conflicts to declare.

## Supplementary Material

RA-016-D6RA07192A-s001

## Data Availability

The data that support this study are openly available in the supplementary information (SI). Supplementary information: experimental procedures, NMR spectra and DFT coordinates. See DOI: https://doi.org/10.1039/d6ra07192a.

## References

[cit1] Seebach D. (1979). Angew. Chem., Int. Ed..

[cit2] Corey E. J., Seebach D. (1975). J. Org. Chem..

[cit3] Corey E. J., Seebach D. (1965). Angew. Chem., Int. Ed..

[cit4] Yus M., Nájera C., Foubelo F. (2003). Tetrahedron.

[cit5] Enders D., Niemeier O., Henseler A. (2007). Chem. Rev..

[cit6] Liu F., Bugaut X., Schedler M., Fröhlich R., Glorius F. (2011). Angew. Chem., Int. Ed..

[cit7] Breslow R. (1958). J. Am. Chem. Soc..

[cit8] Flanigan D. M., Romanov-Michailidis F., White N. A., Rovis T. (2015). Chem. Rev..

[cit9] Stork G., Maldonado L. (1971). J. Am. Chem. Soc..

[cit10] Wang J., Liu X., Feng X. (2011). Chem. Rev..

[cit11] Albright J. D. (1983). Tetrahedron.

[cit12] Gröger H. (2003). Chem. Rev..

[cit13] Reeves J. T., Tan Z., Herbage M. A., Han Z. S., Marsini M. A., Li Z., Li G., Xu Y., Fandrick K. R., Gonnella N. C., Campbell S., Ma S., Grinberg N., Lee H., Lu B. Z., Senanayake C. H. (2013). J. Am. Chem. Soc..

[cit14] Kesavulu G., Balachandra B., Prasad K. R. (2023). Org. Lett..

[cit15] Huang W., Liu H., Lu C.-D., Xu Y.-J. (2016). Chem. Commun..

[cit16] Owens T. D., Hollander F. J., Oliver A. G., Ellman J. A. (2001). J. Am. Chem. Soc..

[cit17] Neese F. (2025). Wiley Interdiscip. Rev.: Comput. Mol. Sci..

[cit18] Hoepker A. C., Collum D. B. (2011). J. Org. Chem..

[cit19] Algera R. F., Gupta L., Hoepker A. C., Liang J., Ma Y., Singh K. J., Collum D. B. (2017). J. Org. Chem..

[cit20] Wojaczynska E., Wojaczynski J. (2020). Chem. Rev..

[cit21] Quintavalla A., Carboni D., Simeone M., Lombardo M. (2023). Org. Lett..

[cit22] Carboni D., Casagranda G., Di Remigio S., Mirone A., Quintavalla A., Lombardo M. (2025). J. Org. Chem..

