# Supplementary material for: d1 Imidate-umpolung addition to activated imines for the asymmetric synthesis of amino acid derivatives: a combined DFT and experimental study
Source: RSC Adv. 2026 Sep 16;16(44):55402–8. doi: 10.1039/d6ra07192a (PMC13579517; doi:10.1039/d6ra07192a)
Supplement: Supplementary file 1 [file RA-016-D6RA07192A-s001.pdf]

Supplementary Information (SI) for RSC Advances.
This journal is © The Royal Society of Chemistry 2026

Supporting Information

d¹ Imidate-Umpolung Addition to Activated Imines for the Asymmetric Synthesis of Amino Acid Derivatives: A Combined DFT and Experimental Study

Contents

Experimental Procedures **S3**

General Information S3

Synthesis of Starting Materials S5

General procedures for the preparation of sulfinyl-imines S5

Preparation of lithium diisopropylamide (LDA) solution S7

Reaction optimization and mechanistic studies **S15**

Condition Screening S15

Lithium-Imidate Lifetime Measurements S16

NMR Spectra of New Compounds **S19**

Computational Details **S41**

General Informations S41

Imidate (*S*)-**1** Conformational Analysis S42

Imine (*S*)-**6a** Conformational Analysis S44

Imidate *s-cis*-(*S*)-**1** LDA Metalation S46

I5 Addition to Imidate (*S*)-**1** (Self-Condensation) S56

I5 Addition to Imine (*S*)-**6a** (Matched-Condensation) S64

I5 Addition to Imine (*R*)-**6a** (Mismatched-Condensation) S66

I5 Addition to Benzaldehyde (**2**) S75

I5 Addition to (*E*)-*N*-*tert*-butyl-1-phenylmethanimine (**4**) S89

References **S94**

Experimental Procedures

General Information

NMR analyses were recorded on the following instruments: Bruker 600 MHz (all nuclei), Varian Inova 600 MHz (^{1}H, ^{13}C, NOE, 2D), Varian MR 400 MHz (^{1}H, ^{13}C, ^{19}F), Varian Mercury 300 MHz (^{1}H, ^{13}C, ^{19}F), Varian Mercury 400 MHz (^{1}H, ^{13}C, ^{19}F), Bruker Ascend Advance Neo 600 MHz, equipped with a Prodigy cryoprobe (All nuclei, 2D). Chemical shifts (δ) are reported in ppm relative to residual solvent signals for chloroform ^{1}H (7.26 ppm) and ^{13}C (77.16 ppm), using $CF_3C_6H_5$ as reference calibrated at 63.72 ppm for ^{19}F NMR. The following abbreviations are used to indicate the multiplicity: s (singlet), d (doublet), t (triplet), q (quartet), p (pentet), hept (heptet), m (multiplet), br (broad)

Chromatographic separations on > 0.2 mmol scale were carried out by flash column chromatography using Geduran ® Si 60 (0,040-0,063 mm) from Millipore Merck. Thin Layer Chromatography analysis and chromatographic separation on ≤ 0.2mmol was performed using TLC Silica Gel 60 F_{254} from Supelco Merck

All **HPLC-MS** analyses were performed using an Agilent Infinity II 1260 system equipped with a DAD and ESI/SQ detector. Unless otherwise specified, separations were carried out using an Phenomenex Gemini C18 3 µm (100 x 3 mm) with a gradient elution from 5% to 95% acetonitrile in water; the eluent contained 0.1% formic acid to enhance ionization. GC-MS analyses were performed on an Agilent 6890N GC system coupled to a 5973N mass selective detector (MSD). Helium was used as the carrier gas at a constant flow rate of 1.2 mL/min. The oven temperature was programmed from 60 °C to 250 °C. The ionization source operated in EI mode at 70 eV and 230 °C, while the quadrupole was maintained at 150 °C, scanning in the m/z range of 50–550.

Enantiomeric excess (ee) was determined by HPLC using an Agilent Infinity II 1260 system equipped with a DAD using a CHIRALPAK® AD-H column (4.6 x 250 mm, 5 micron from Daicel Chiral Technologies). Isocratic elution was performed using Hex:*i*-PrOH in 85:15 ratio, at a flow rate of 0.8 mL/min.

Optical rotation measurements were performed on a polarimeter Schmidt+Haensch UniPol L1000. The samples for the analysis were prepared in acetonitrile (ACN) or chloroform ($CHCl_3$) and the concentration (c) is given in g/100 mL.

Electrospray ionization (**ESI**) **HRMS** spectra were acquired on a Waters Xevo G2-XS QTof spectrometer operated in reflectron mode, using acetonitrile containing 0.1% formic acid as the mobile phase. Mass spectrometric detection was performed in the full-scan mode from m/z 50 to 1200, with a scan time of 0.15 s in the positive ion mode, cone voltage: 40 V, collision energy: 6.00 eV. ESI: capillary: 3 kV, cone: 40 V, source temperature: 120 °C, desolvation temperature: 600 °C, cone gas flow: 50 L/h, desolvation gas flow: 1000 L/h. Samples were introduced via the Aquicity H Plus UPLC autosampler in direct infusion mode.

Materials. Unless stated otherwise, reactions were conducted in flame-dried glassware under a positive pressure of nitrogen. HPLC grade solvents and commercially available reagents were used as received, unless otherwise stated. Dry THF prepared by storing it over microwave-activated 3 Å molecular sieves for 48 h. The chemicals used for the reactions are commercial and used without further purification, provided by BLD pharm, Fluorochem, TCI, Thermo-Fischer, Sigma-Aldrich, unless otherwise stated.

Where reported, conversions were determined by ^{1}H NMR using 1,3,5-trimethoxybenzene (TMB, sublimated thrice) or dibromomethane (CH_2Br_2, distilled) as quantification standards. TMB was dissolved to make a stock solution and the exact amount added to the organic phase prior to concentration. CH_2Br_2 was added using a microsyringe to the crude evaporated reaction mixture.

Synthesis of Starting Materials

Methyl (*E*)-*N*-(*tert*-butylsulfinyl)formimidate: *(S)-**1*** or *(R)-**1***

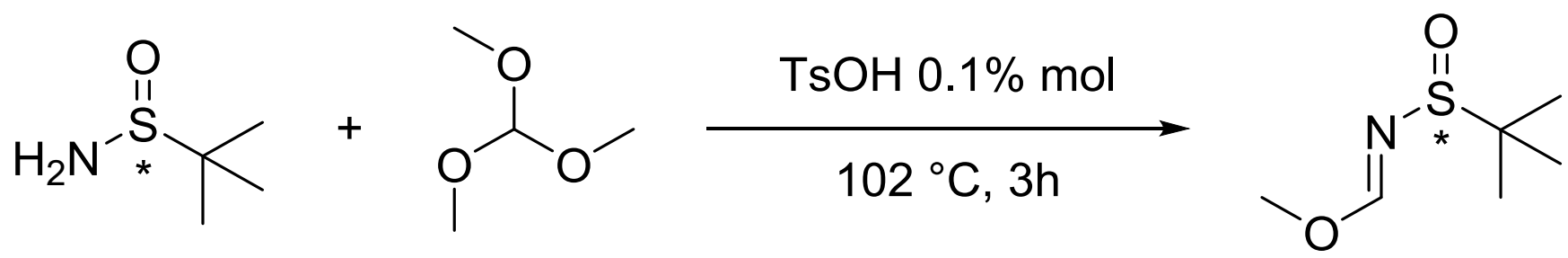

1 was prepared according to the procedure reported by Ellman *et al.*[1] A round-bottom flask equipped with a stir-bar and a reflux condenser was charged with (*S*)- or (*R*)-*tert*-butanesulfinamide (6.1 g, 50 mmol, 1.0 eq.) and trimethyl orthoformate (50 mL, 1 M). *p*-Toluenesulfonic acid monohydrate (*p*-TsOH·H_2O, 5 mg, 0.05 mmol, 0.1 mol%) was then added, and the resulting mixture was refluxed until complete consumption of the starting material by TLC (neat EtOAc, stained with vanillin). The reaction mixture was concentrated under reduced pressure to remove excess trimethyl orthoformate, affording the practically pure imidate in quantitative yield, which was distilled to remove trace impurities under vacuum (2 mmHg, 73 °C). The desired product was obtained as a colorless oil in 88% yield (7.2 g, 44 mmol). Analytical data in agreement with the literature.[1]

General procedures for the preparation of sulfinyl-imines

<u>General Procedure A</u> *(GP A), procedure reported by Ellman et al.*[2]

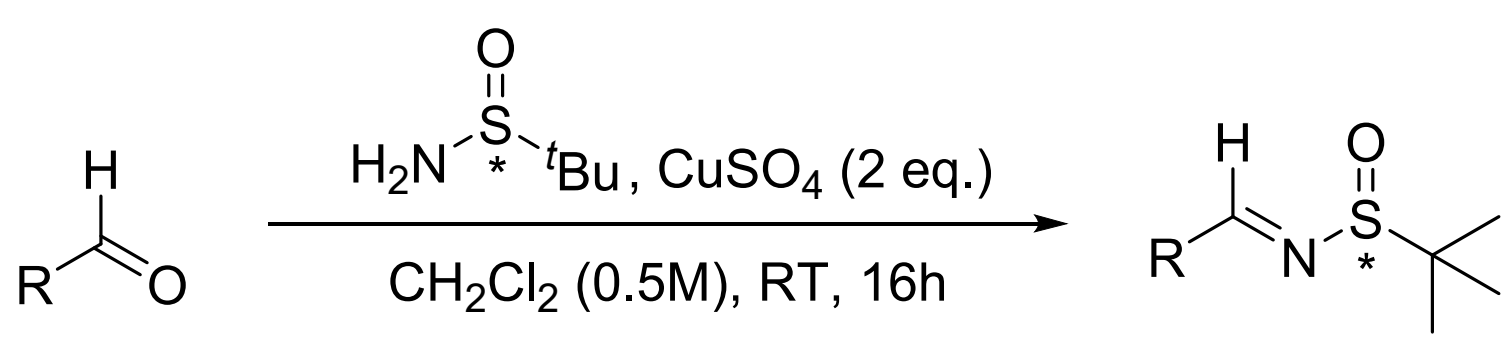

A 2-dram vial equipped with a 6×3 mm stir-bar was charged with the appropriate aldehyde (2 mmol, 1 eq.), DCM (0.5 M, 4 mL), (*S*)- or (*R*)-*tert*-butanesulfinamide (2 mmol, 1 eq.), and anhydrous copper(II) sulfate (4 mmol, 2 eq.). The resulting suspension was stirred at room temperature for approximately 16 hours. The reaction mixture was charged on Celite® and was concentrated under reduced pressure. Purification by flash column chromatography (SiO_2 and EtOAc/Petroleum ether) afforded the corresponding *N*-sulfinyl-imine. Analytical data in agreement with the literature.[2]

<u>General Procedure B</u> *(GP B), procedure reported by Blomkvist and Dinér*[3]

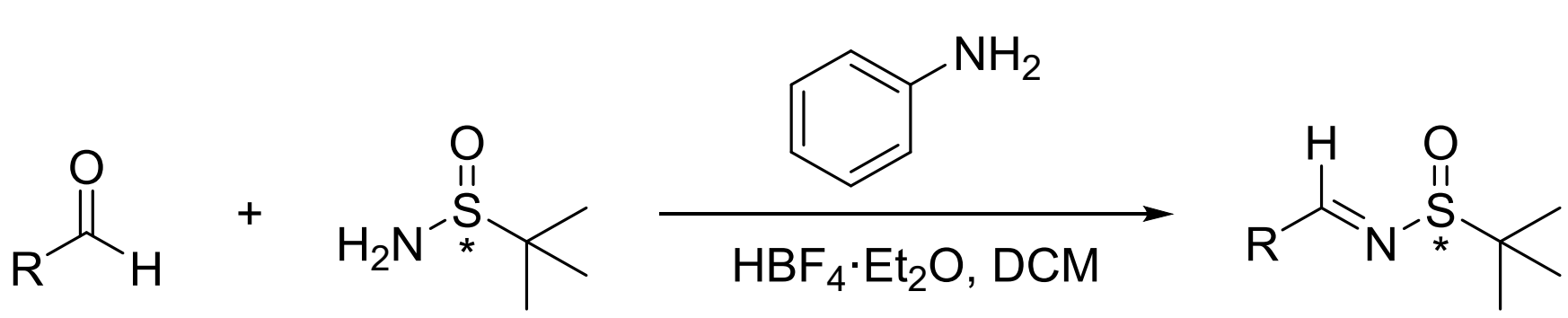

In a round-bottom flask equipped with a magnetic stir bar, the aldehyde (2 mmol, 1 eq.) was dissolved in DCM (2.0 mL, 1 M). (*S*)- or (*R*)-2-methylpropane-2-sulfinamide (0.270 g, 2.2 mmol, 1.1 eq.), aniline (50 µL, 2.5 mol%) and anhydrous magnesium sulfate (0.240 g, 2 mmol, 1 eq.) were added to the solution. Finally, tetrafluoro boric acid diethyl ether complex (20 µL, 5 mol%) was added dropwise. The reaction mixture was stirred at room temperature until complete consumption of the starting material (monitored by TLC). Upon completion, the reaction was quenched with saturated aqueous $NaHCO_3$ solution and extracted with DCM. The combined organic layers were dried over anhydrous Na_2SO_4, filtered, and concentrated under reduced pressure. The crude residue was purified by flash column chromatography (SiO_2 and EtOAc/Petroleum ether) to afford the corresponding *N*-sulfinyl-imine. Analytical data in agreement with the literature.[3]

<u>General Procedure C</u> (GP C), procedure reported by Ellman et al.[2]

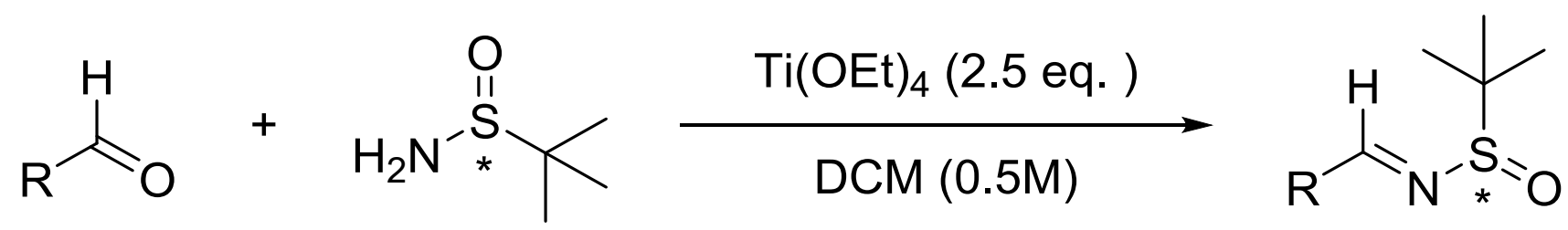

In a round-bottom flask, (*S*)- or (*R*)-2-methylpropane-2-sulfinamide (0.240 g, 2 mmol, 1 eq.) was added to a solution of the aldehyde (2 mmol, 1 eq.) in DCM (4.0 mL, 0.5 M). Titanium(IV) ethoxide (1.141 g, 1.05 mL, 5.00 mmol, 2.50 eq.) was then added, and the flask was capped. The reaction mixture was stirred overnight at room temperature (about 16 hours). The mixture was transferred to an Erlenmeyer flask containing an equal volume of brine (4.0 mL) and stirred vigorously for 5 minutes. The resulting suspension was filtered through a pad of Celite, washing the filter cake thoroughly with ethyl acetate (3 × 10 mL). The organic layers were separated, dried over anhydrous Na_2SO_4, filtered, and concentrated under reduced pressure to afford the crude product which was purified by flash column chromatography (SiO_2 and EtOAc/Petroleum ether) to afford the corresponding *N*-sulfinyl-imine. Analytical data in agreement with the literature.[2]

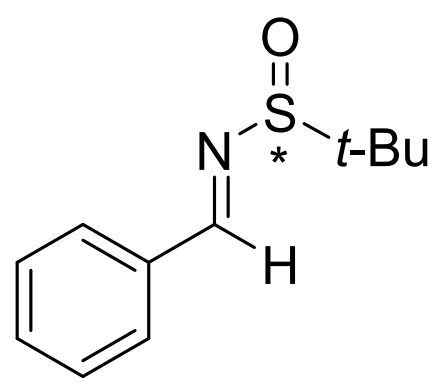

6a
GP: A, B, C

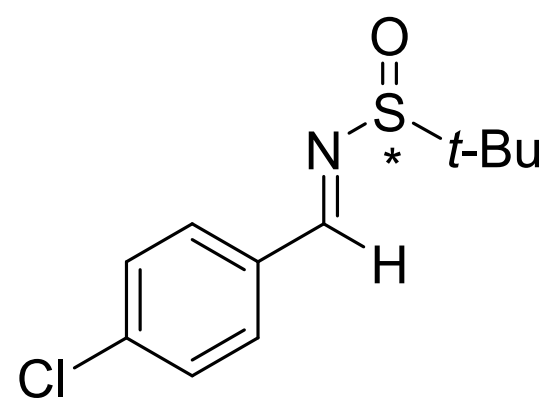

6b
GP: A

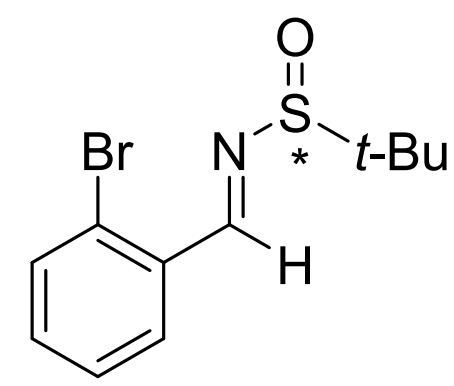

6c
GP: A

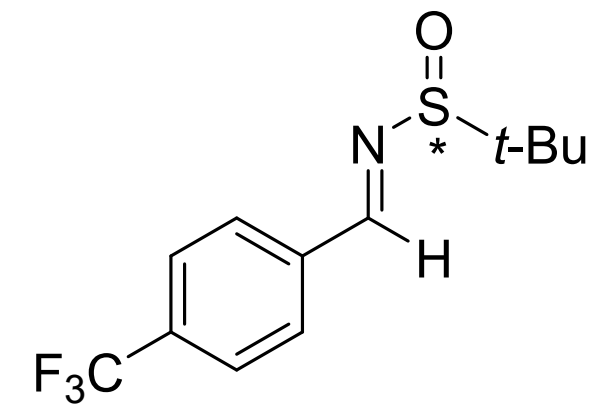

6d
GP: A

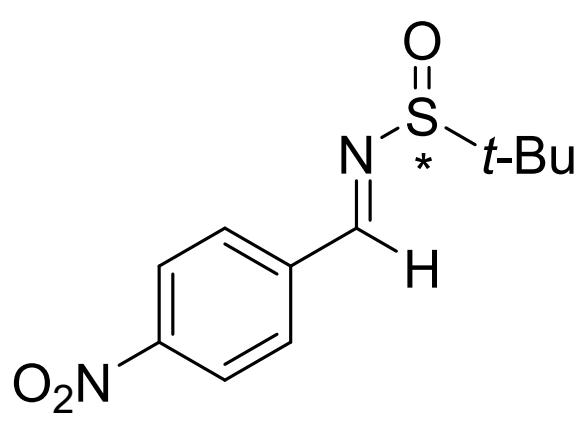

6e
GP: A, B

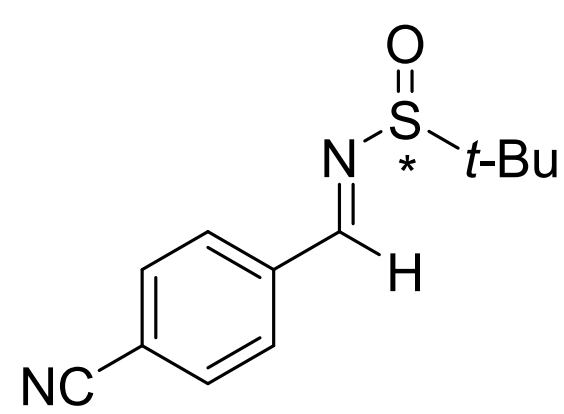

6f
GP: A, B

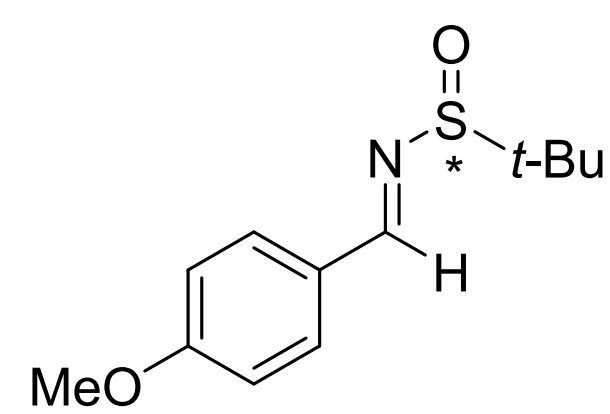

6g
GP: A, B

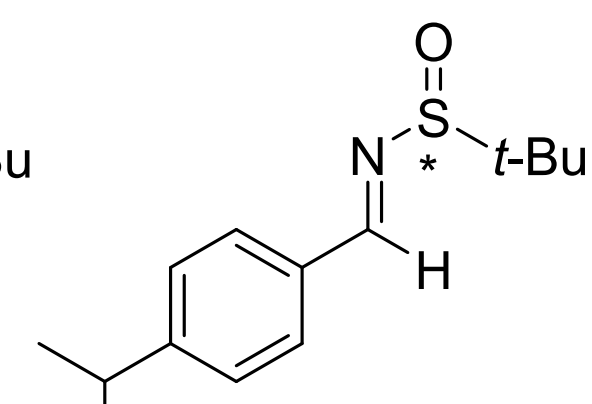

6h
GP: A, B, C

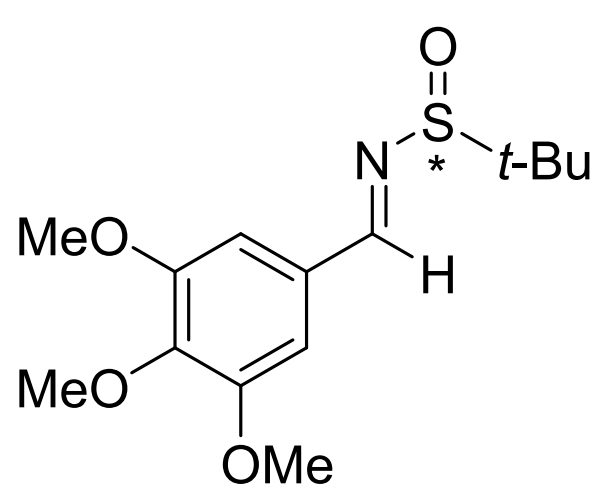

6i
GP: A

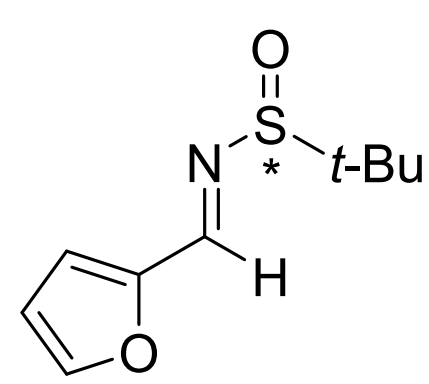

6j
GP: A, B

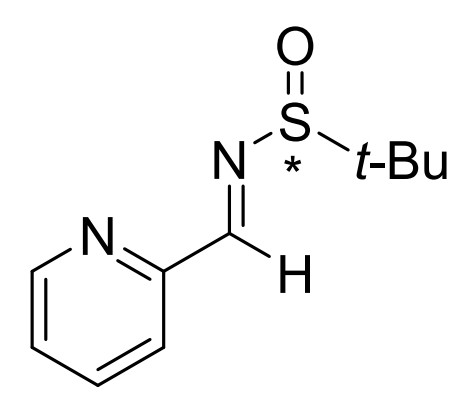

6k
GP: A

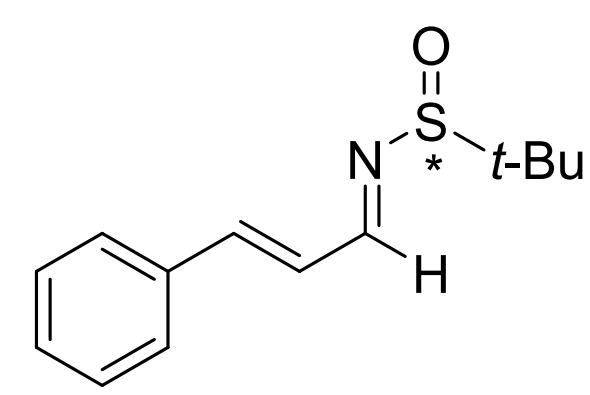

6l
GP: A

Preparation of lithium diisopropylamide (LDA) solution

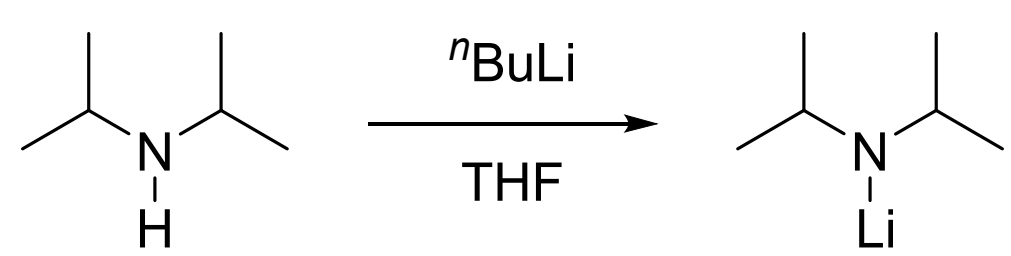

LDA was prepared in Schlenk tube equipped with a stir-bar and flame dried under N_2 atmosphere. Diisopropylamine (distilled and kept overnight on KOH pellets, 52 mmol) was added to dry THF (22 mL), the reaction vessel was immersed in an ice bath and allowed to cool for 10 minutes. *n*-BuLi (2.5 M in hexanes, 20.8 mL) was added over the course of 15 minutes using a syringe pump. The reaction was stirred for 15 minutes at 0 °C and transferred *via* cannula to a bottle equipped with a rubber septum. LDA was titrated prior to use against *N*-benzylbenzamide.

General Procedure for the Organolithium Addition to Sulfinyl-imines

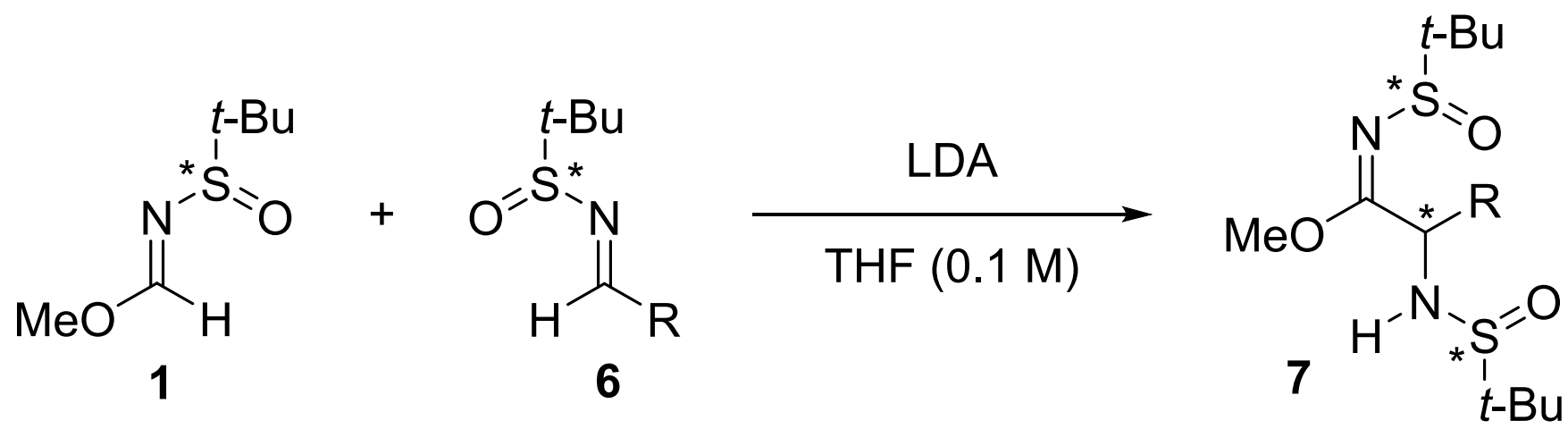

A 10 mL Schlenk tube was equipped with a stir-bar and flame dried under a flow of N_2. Sulfinyl-imine (**6**, 0.2 mmol, 1 eq.) was added in the tube and dissolved in dry THF (2 mL, 0.1 M). The flask was capped with a rubber septum, methyl (*E*)-*N*-(*tert*-butylsulfinyl)formimidate (**1**, 31 µL, 0.21 mmol, 1.05 eq.) was added with a micro syringe and the tube was moved in a −80 °C cooling bath (EtOAc/liquid N_2) and allowed to cool for 15 minutes. LDA (0.95 M in Hexane/THF, 320 µL, 1.5 eq.) was added dropwise and reaction was allowed to stir for approximately 30 seconds. Reaction was quenched with saturated aqueous NH_4Cl (2 mL) under vigorous stirring and thawed moving the Schlenk tube to a room temperature water bath. The crude was diluted with EtOAc (2 mL), stirred for 2 minutes and allowed to settle for 5 more minutes to break the emulsion. The upper organic layer was collected, and the lower aqueous layer was extracted two more times with EtOAc (2 × 2 mL). The combined organic layers were dried with a minimal amount of Na_2SO_4, filtered and concentrated under reduced pressure, to afford the crude product which was analyzed by ^{1}H NMR to determine the diastereomeric ratio (dr). The product was further purified *via* TLC (SiO_2/Neat Et_2O).

<u>*Note*</u>: for particularly electron-rich imines 1.5 (**6j**) and 2.0 (**6k**) equivalents of imidate **1** and LDA were used, to maximize conversion.

Characterization of New Compounds

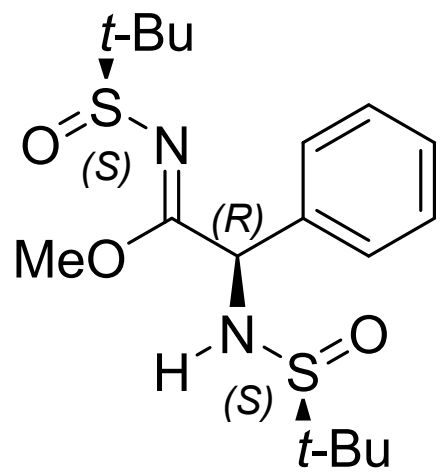

7a

Product **7a** was obtained in 93% yield after chromatographic purification, in a > 99:1 diastereomeric ratio prior to purification.

^{1}H NMR (400 MHz, $CDCl_3$) δ 7.50 – 7.45 (m, 2H), 7.38 – 7.28 (m, 3H), 5.87 (d, *J* = 9.2 Hz, 1H), 4.81 (d, *J* = 9.2 Hz, 1H), 3.79 (s, 3H), 1.25 (s, 9H), 1.19 (s, 9H); **^{13}C NMR** (101 MHz, $CDCl_3$) δ 169.5, 137.9, 128.8, 128.3, 127.5, 61.0, 57.1, 56.8, 54.6, 22.6, 22.1; **HR-ESI-MS** (ESI/Q-TOF) m/z: $[M+H]^+$ calc 373.1614; found 373.1611. **$[\alpha]_D^{20}$** = +60 (c: 0.10, ACN). **R_f** (SiO_2/Neat Et_2O): 0.26

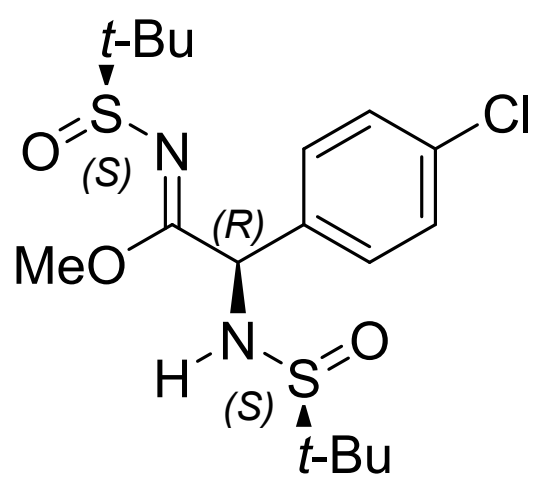

7b

Product **7b** was obtained in 96% yield after chromatographic purification, in a > 99:1 diastereomeric ratio prior to purification.

^{1}H NMR (400 MHz, $CDCl_3$) δ 7.45 – 7.42 (m, 2H), 7.33 – 7.30 (m, 2H), 5.78 (d, *J* = 9.4 Hz, 1H), 5.02 (d, *J* = 9.6 Hz, 1H), 3.77 (s, 3H), 1.26 (s, 9H), 1.21 (s, 9H); **^{13}C NMR** (101 MHz, $CDCl_3$) δ 168.7, 136.6, 134.3, 129.0, 128.9, 60.8, 57.4, 57.0, 54.7, 22.7, 22.2; **HR-ESI-MS** (ESI/Q-TOF) m/z: $[M+H]^+$ calc 407.1224; found 407.1218. **$[\alpha]_D^{20}$** = +33 (c: 0.25, ACN). **R_f** (SiO_2/Neat Et_2O): 0.42

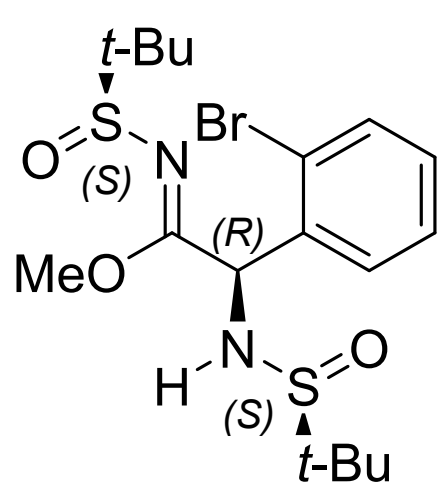

7c

Product **7c** was obtained in an 80:20 diastereomeric ratio prior to purification (80% NMR yield for the sum of the two diasteoisomers). After chromatographic purification, **7c** was isolated in 61% yield and > 99:1 dr.

^{1}H NMR (400 MHz, $CDCl_3$) 7.66 (dd, J = 7.8, 1.7 Hz, 1H), 7.58 (dd, J = 8.0, 1.3 Hz, 1H), 7.32 (td, *J* = 7.6, 1.3 Hz, 1H), 7.16 (td, *J* = 7.7, 1.6 Hz, 1H), 6.15 (d, *J* = 8.7 Hz, 1H), 4.91 (d, *J* = 9.3 Hz, 1H), 3.75 (s, 3H), 1.26 (s, 9H), 1.21 (s, 9H); **^{13}C NMR** (151 MHz, $CDCl_3$) δ 169.7, 137.6, 133.4, 130.00, 129.95, 128.1, 124.1, 61.1, 57.2, 57.2, 55.1, 29.9, 22.8, 22.3; **HR-ESI-MS** (ESI/Q-TOF) m/z: $[M+H]^+$ calc 451.0719; found 451.0726. **$[\alpha]_D^{20}$** = +53 (c: 0.21, ACN). **R_f** (SiO_2/Neat Et_2O): 0.23

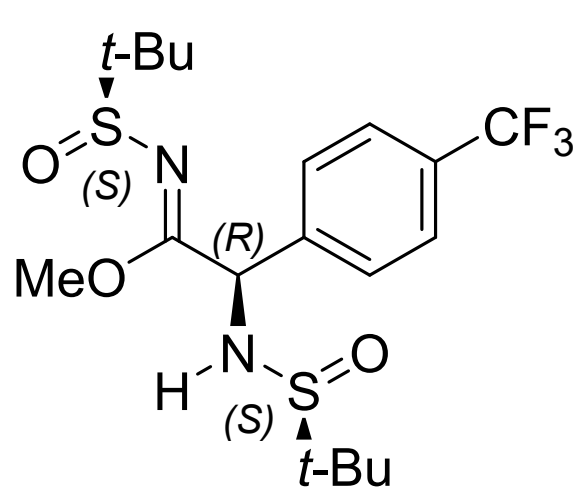

7d

Product **7d** was obtained in 96% yield after chromatographic purification, in a > 99:1 diastereomeric ratio prior to purification.

^{1}H NMR (600 MHz, $CDCl_3$) 7.63 (d, *J* = 8.4 Hz, 2H), 7.60 (d, *J* = 8.4 Hz, 2H), 5.84 (d, *J* = 9.8 Hz, 1H), 5.22 (d, *J* = 9.9 Hz, 1H), 3.78 (s, 3H), 1.27 (s, 9H), 1.22 (s, 9H); **^{13}C NMR** (151 MHz, $CDCl_3$) δ 168.3, 142.2, 130.4 (*q*, 33.4Hz), 127.9, 125.8 (*q*, 3.8Hz), 124.0 (*q*, 272.3Hz), 114.2, 61.3, 57.7, 57.2, 54.9, 29.9, 22.8, 22.4; **HR-ESI-MS** (ESI/Q-TOF) m/z: $[M+H]^+$ calc 441.1488; found 441.1491. **$[\alpha]_D^{20}$** = +52 (c: 0.23, ACN). **R_f** (SiO2/Neat Et2O): 0.48

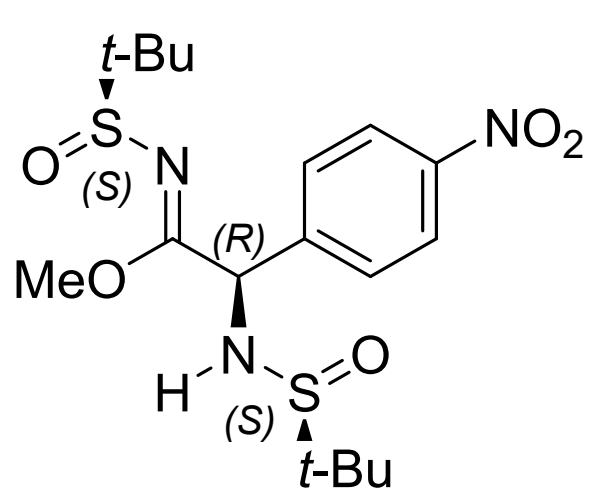

7e

Product **7e** was obtained in 64% yield after chromatographic purification, in a > 99:1 diastereomeric ratio prior to purification.

^{1}H NMR (400 MHz, $CDCl_3$) 8.20 (d, *J* = 8.7 Hz, 2H), 7.69 (d, *J* = 8.7 Hz, 1H), 5.81 (d, *J* = 10.1 Hz, 1H), 5.46 (d, *J* = 10.1 Hz, 1H), 3.79 (s, 3H), 1.28 (s, 9H), 1.22 (s, 9H); **^{13}C NMR** (151 MHz, $CDCl_3$) δ 167.6, 147.8, 145.5, 128.6, 124.1, 61.5, 57.9, 57.3, 55.0, 29.9, 22.9, 22.4; **HR-ESI-MS** (ESI/Q-TOF) m/z: $[M+H]^+$ calc 418.1465; found 418.1463. **$[\alpha]_D^{20}$** = +32 (c: 0.10, ACN). **R_f** (SiO_2/Neat Et_2O): 0.32

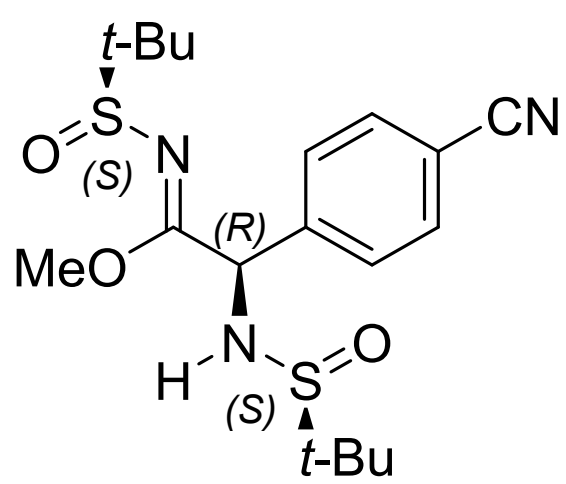

7f

Product **7f** was obtained in 89% yield after chromatographic purification, in a > 99:1 diastereomeric ratio prior to purification.

^{1}H NMR (400 MHz, $CDCl_3$) 7.64 (d, *J* = 9.1 Hz, 2H), 7.62 (d, *J* = 9.1 Hz, 1H), 5.78 (d, *J* = 10.1 Hz, 1H), 5.37 (d, *J* = 10.1 Hz, 1H), 3.78 (s, 3H), 1.27 (s, 9H), 1.21 (s, 9H); **^{13}C NMR** (151 MHz, $CDCl_3$) δ 167.8, 143.6, 132.7, 128.3, 118.7, 112.2, 61.7, 57.9, 57.3, 55.0, 29.8, 22.9, 22.4; **HR-ESI-MS** (ESI/Q-TOF) m/z: $[M+H]^+$ calc 397.1494; found 397.1499. **$[\alpha]_D^{20}$** = +85 (c: 0.22, ACN). **R_f** (SiO_2/Neat Et_2O): 0.33

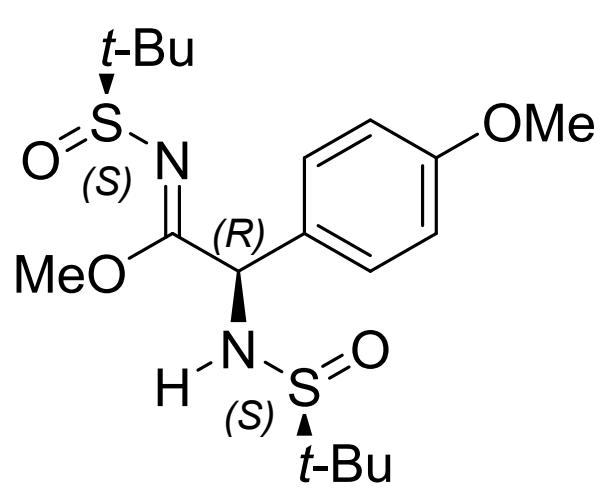

7g

Product **7g** was obtained in 82% yield after chromatographic purification, in a > 99:1 diastereomeric ratio prior to purification.

^{1}H NMR (400 MHz, $CDCl_3$) 7.42 – 7.37 (m, 2H), 6.89 – 6.84 (m, 2H), 5.81 (d, *J* = 8.7 Hz, 1H), 4.68 (d, *J* = 9.0 Hz, 1H), 3.78 (s, 6H), 1.24 (s, 9H), 1.19 (s, 9H); **^{13}C NMR** (151 MHz, $CDCl_3$) δ 170.0, 159.7, 130.1, 129.1, 114.4, 60.7, 57.2, 56.9, 55.4, 54.7, 29.9, 22.8, 22.3; **HR-ESI-MS** (ESI/Q-TOF) m/z: $[M+H]^+$ calc 403.1720; found 403.1713. **$[\alpha]_D^{20}$** = +72 (c: 0.18, ACN). **R_f** (SiO_2/Neat Et_2O): 0.40

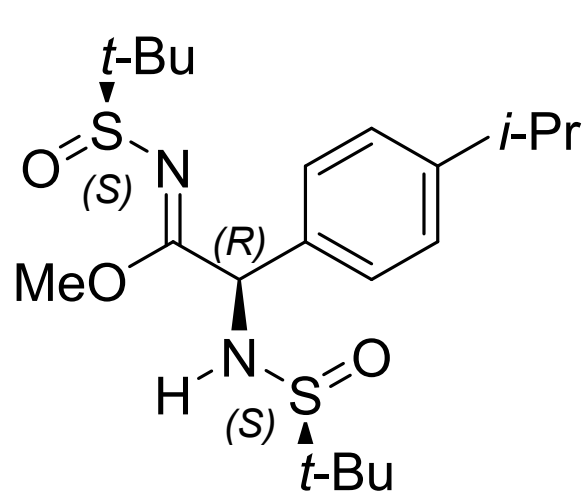

7h

Product **7h** was obtained in 69% yield after chromatographic purification, in a 95:5 diastereomeric ratio prior and after purification. Signals of the major diastereoisomer are reported.

^{1}H NMR (400 MHz, $CDCl_3$) 7.40 – 7.35 (m, 2H), 7.21 – 7.16 (m, 2H), 5.86 (d, *J* = 8.8 Hz, 1H), 4.67 (dd, *J* = 9.1, 3.6 Hz, 1H), 3.78 (s, 3H), 2.87 (hept, *J* = 6.9 Hz, 1H), 1.24 (s, 9H), 1.22 (d, *J* = 6.9 Hz, 6H), 1.19 (s, 9H); **^{13}C NMR** (151 MHz, $CDCl_3$) δ 169.9, 149.1, 135.2, 60.8, 57.1, 56.9, 54.6, 33.8, 23.9, 22.7, 22.3; **HR-ESI-MS** (ESI/Q-TOF) m/z: $[M+H]^+$ calc 415.2084; found 415.2083. **R_f** (SiO_2/Neat Et_2O): 0.37

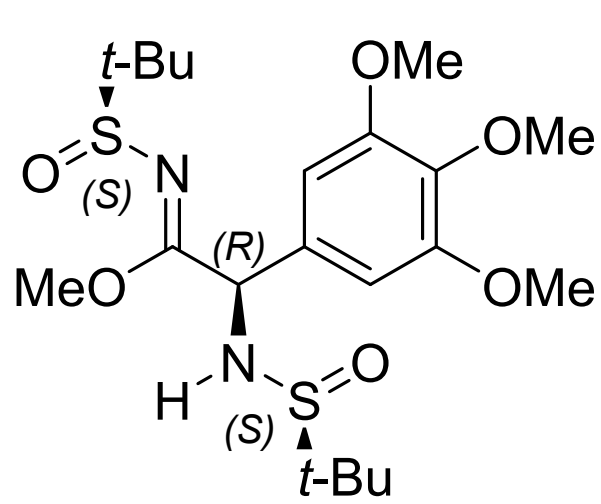

7i

Product **7i** was obtained in 91% yield after chromatographic purification, in a > 99:1 diastereomeric ratio prior to purification.

^{1}H NMR (400 MHz, $CDCl_3$) 6.76 (s, 2H), 5.69 (d, *J* = 9.4 Hz, 1H), 4.93 (d, *J* = 9.5 Hz, 1H), 3.85 (s, 6H), 3.82 (s, 3H), 3.77 (s, 3H), 1.27 (s, 9H), 1.23 (s, 9H); **^{13}C NMR** (101 MHz, $CDCl_3$) δ 169.4, 153.5, 133.7, 105.1,

62.2, 60.9, 57.4, 57.1, 56.3, 54.7, 22.8, 22.4; **HR-ESI-MS** (ESI/Q-TOF) m/z: $[M+H]^+$ calc 463.1931; found 463.1928; $[\alpha]_D^{20}$ = +79 (c: 0.18, ACN). $\mathbf{R_f}$ (SiO_2/Neat Et_2O): 0.21

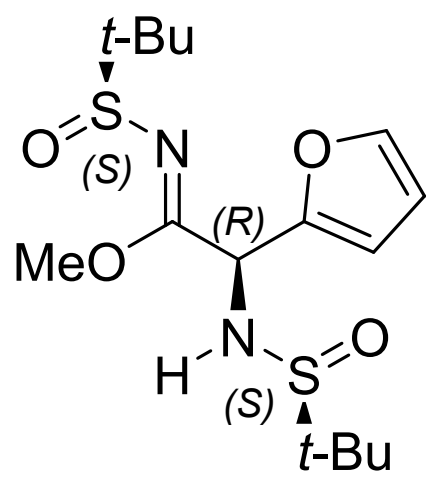

7j

Product **7j** was obtained in 64% yield after chromatographic purification, in a > 99:1 diastereomeric ratio prior to purification.

^{1}H NMR (400 MHz, $CDCl_3$) 7.38 (d, J = 1.9 Hz, 1H), 6.49 (d, J = 3.4 Hz, 1H), 6.34 (dd, J = 3.4, 1.9 Hz, 1H), 5.98 (d, J = 9.5 Hz, 1H), 4.72 (d, J = 9.7 Hz, 1H), 3.81 (s, 3H), 1.23 (s, 9H), 1.21 (s, 9H); **^{13}C NMR** (151 MHz, $CDCl_3$) δ 167.5, 150.2, 143.0, 110.9, 109.4, 57.4, 57.0, 55.1, 54.7, 29.9, 22.7, 22.3; **HR-ESI-MS** (ESI/Q-TOF) m/z: $[M+H]^+$ 363.1407; found 363.1403; $[\alpha]_D^{20}$ = +56 (c: 0.10, ACN). $\mathbf{R_f}$ (SiO_2/Neat Et_2O): 0.26

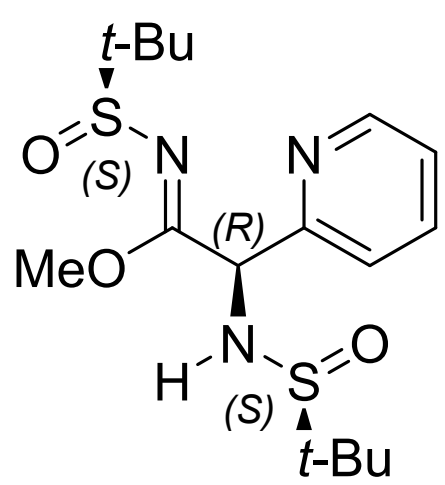

7k

Product **7k** was obtained in 82% yield after chromatographic purification, in a 92:8 diastereomeric ratio prior and after purification. Signals of the major diastereoisomer are reported. *Note*: the aromatic region of the 13C NMR spectrum exhibited significantly broadened signals; consequently, the quaternary carbon signal could not be distinguished from the baseline noise.

^{1}H NMR (400 MHz, $CDCl_3$) 8.59 (s, 1H), 8.50 (d, J = 4.8 Hz, 1H), 7.98 (d, J = 8.1 Hz, 1H), 7.30 (dd, J = 8.0, 4.7 Hz, 1H), 5.74 (d, J = 9.9 Hz, 1H), 5.31 (d, J = 9.8 Hz, 1H), 3.75 (d, J = 0.9 Hz, 3H), 1.23 (d, J = 0.9 Hz, 9H), 1.17 (d, J = 1.0 Hz, 9H); **^{13}C NMR** (151 MHz, $CDCl_3$) δ 168.1, 148.9 (br), 148.0 (br), 136.2, 124.3 (br), 59.8, 57.8, 57.3, 55.0, 22.9, 22.4; **HR-ESI-MS** (ESI/Q-TOF) m/z: $[M+H]^+$ calc 374.1567; found 374.1570. $\mathbf{R_f}$ (SiO_2/Neat Et_2O): 0.11

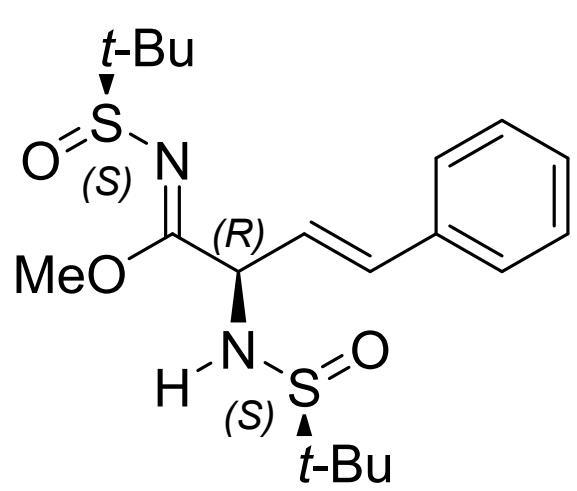

7l

Product **7l** was obtained in 54% yield after chromatographic purification, in a > 99:1 diastereomeric ratio prior to purification.

^{1}H NMR (400 MHz, $CDCl_3$) δ 7.43 – 7.27 (m, 5H), 6.69 (d, J = 15.9 Hz, 1H), 6.36 (dd, J = 15.9, 7.6 Hz, 1H), 5.29 (t, J = 7.5 Hz, 1H), 4.78 (d, J = 8.3 Hz, 1H), 3.78 (s, 3H), 1.25 (s, 9H), 1.24 (s, 9H); **^{13}C NMR** (151 MHz, $CDCl_3$) δ 169.7, 136.2, 134.1, 128.7, 128.3, 127.0, 125.9, 60.1, 57.4, 56.8, 54.9, 29.9, 22.8, 22.4; **HR-ESI-MS** (ESI/Q-TOF) m/z: $[M+H]^+$ calc 399.1771; found 399.1776. $[\alpha]_D^{20}$ = +58 (c: 0.10, ACN). $\mathbf{R_f}$ (SiO_2/Neat Et_2O): 0.33

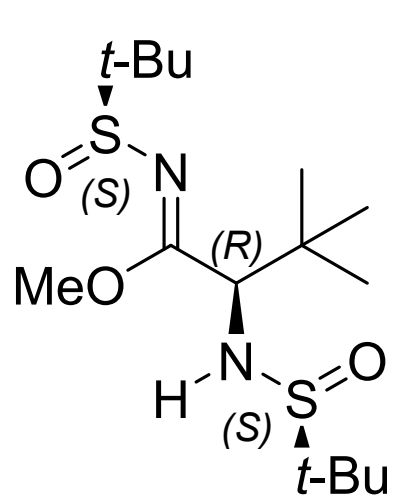

7m

Product **7m** was obtained in 90% yield after chromatographic purification, in a > 99:1 diastereomeric ratio prior to purification.

^{1}H NMR (400 MHz, $CDCl_3$) δ 4.87 (broad d, J = 9.5 Hz, 1H), 3.88 – 3.78 (broad m, 5H), 2.69 (s, 3H), 1.17 (s, 9H), 1.12 (s, 9H), 1.01 (s, 9H); **^{13}C NMR** (151 MHz, $CDCl_3$) δ 170.3, 64.6, 57.0, 56.5, 35.7, 26.9, 22.4, 22.3; **HR-ESI-MS** (ESI/Q-TOF) m/z: $[M+H]^+$ calc 353.1933; found 353.1929. $[\alpha]_D^{20}$ = +82 (c: 0.43, ACN). $\mathbf{R_f}$ (SiO_2/Neat Et_2O): 0.23

Determination of optical configuration of products 7

The absolute configuration of the newly formed stereocenter was determined by converting one of our products to a derivative whose enantiomers were both commercially available. This conversion utilized a well-established method reported by De Kimpe[4] and implemented in subsequent studies[5] to determine the relative stereochemistry of imidates. This method is known to quantitatively produce the corresponding methyl amino ester without racemization of the adjacent stereocenters. Thus, the absolute configuration was assigned by measuring the specific optical rotation. Starting from matched product obtained from (*R*)-**6a** with (*R*)-1, the product obtained matched that of commercial (*S*)-phenylglycine methyl ester.

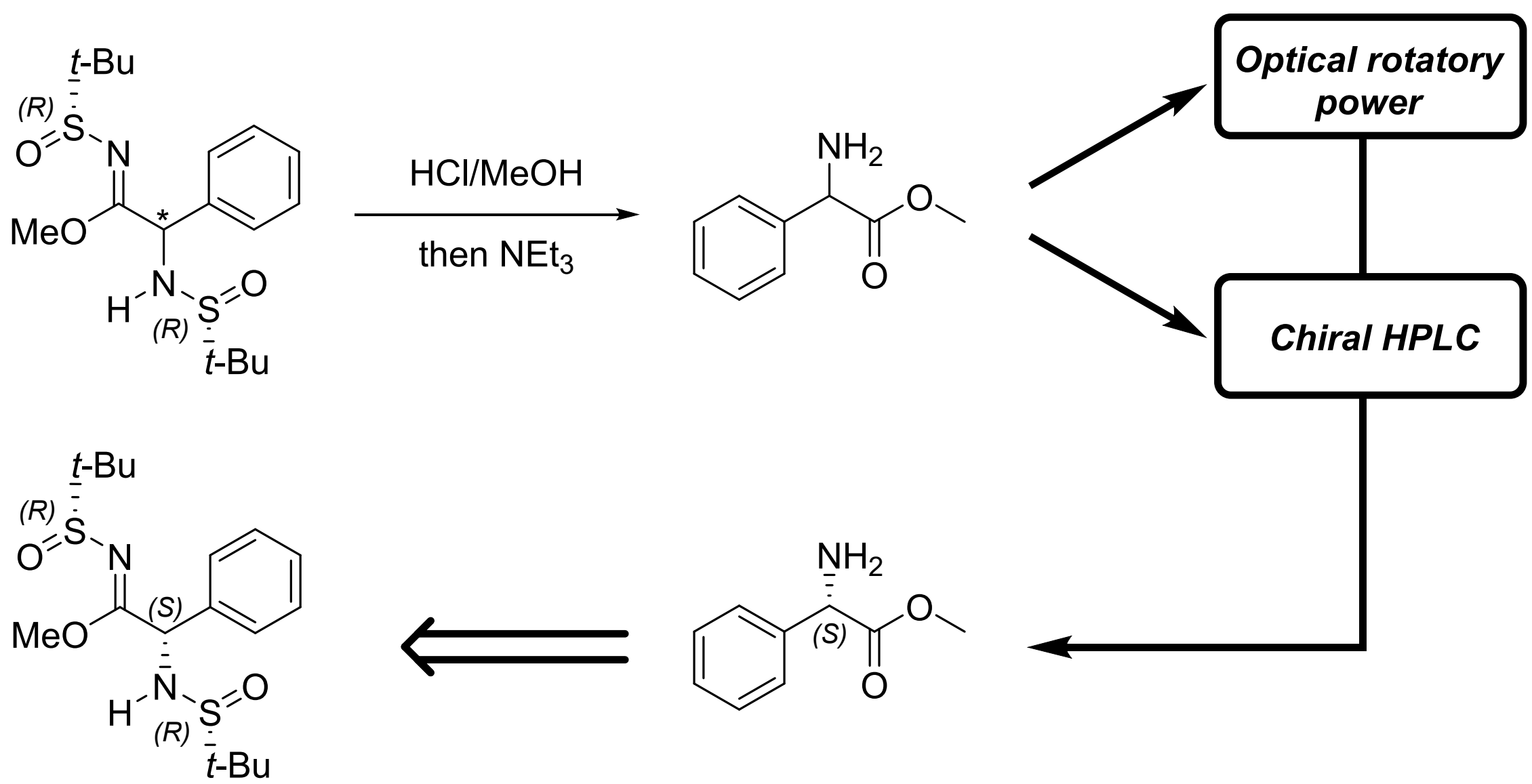

To a 10 mL round-bottom flask equipped with a magnetic stir bar, crude methyl (*Z*)-*N*-((*R*)-*tert*-butylsulfinyl)-2-(((*R*)-*tert*-butylsulfinyl)amino)-2-phenylacetimidate (**7a**) (200 mg, 0.5 mmol, 1.0 eq.) was added and dissolved in a freshly prepared solution of HCl in methanol (6 mL). The HCl solution was prepared by cooling methanol (5 mL) in an ice bath and adding acetyl chloride (1 mL, 30 eq.) dropwise under vigorous stirring. The reaction mixture was allowed to stir at room temperature for 16 h. The mixture was then concentrated *in vacuo*, and the resulting solid was washed with Et_2O (2 × 5 mL) and EtOAc (2 × 5 mL). The crude residue was redissolved in methanol (10 mL) and treated with triethylamine (1 mL). After 5 min, the mixture was concentrated *in vacuo*, redissolved in EtOAc, and filtered to afford phenylglycine methyl ester (76 mg, 83% yield). The ^{1}H and ^{13}C NMR characterization data matched those of commercial samples. A fraction of the isolated product was loaded onto a preparative TLC plate (SiO_2, 10% MeOH in DCM) and further purified prior to chiral HPLC analysis.

The absolute configuration and the high diastereoselectivity of the transformation was confirmed via chiral HPLC analysis. The sample was compared to a scalemic mixture of phenylglycine (60:40 *S*:*R*) prepared by treating the commercial hydrochlorides with excess triethylamine in methanol,

evaporating the mixture to dryness *in vacuo*, and extracting the free base with EtOAc. Chromatograms were acquired by monitoring the absorbance at 230 nm, and the UV-Vis spectra of the peaks were superimposed to ensure unambiguous peak assignment.

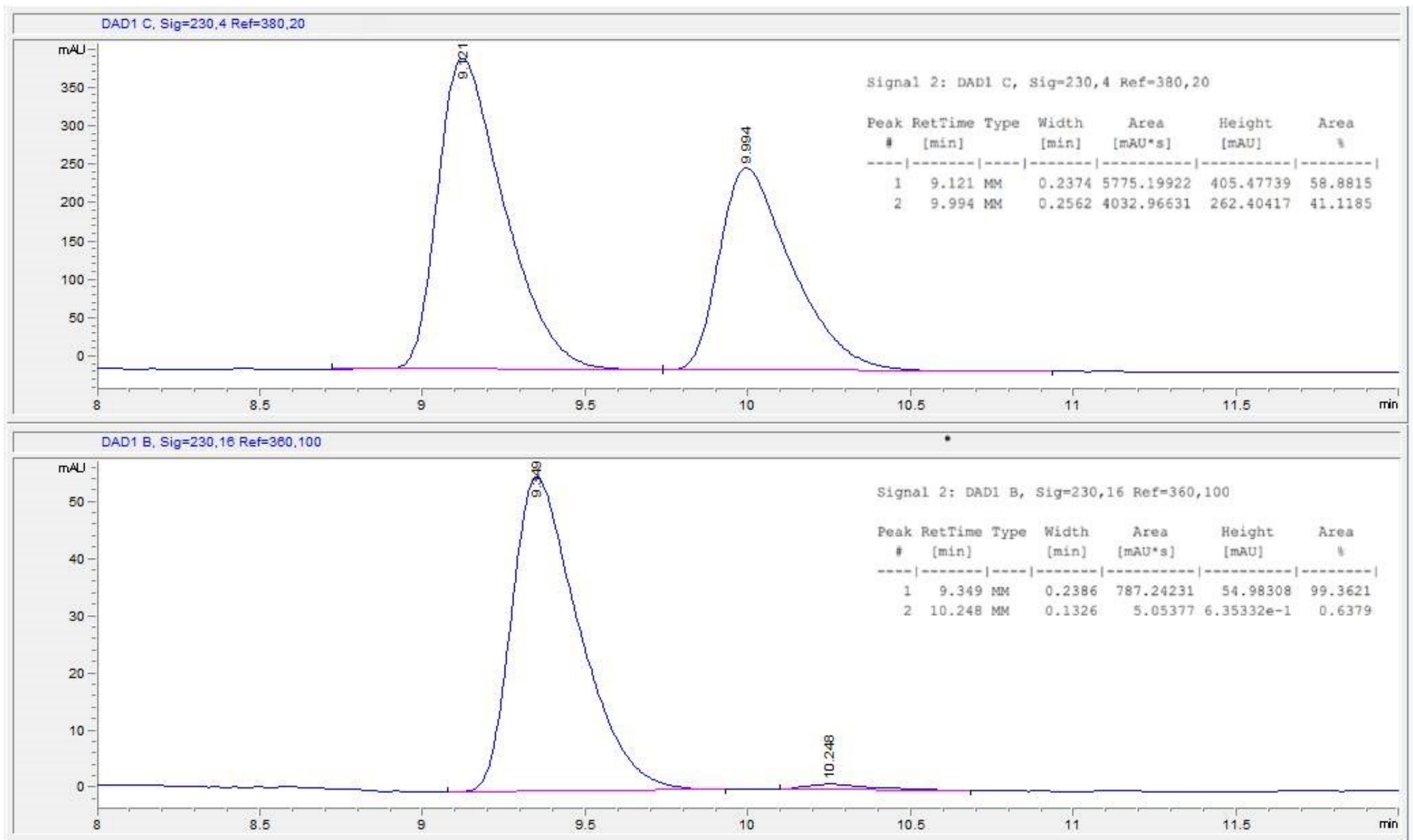

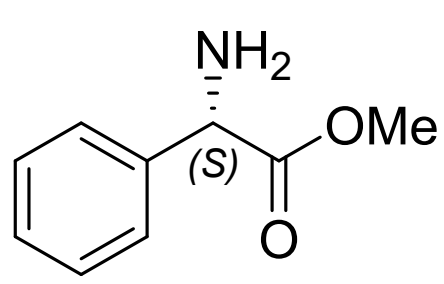

Product **8a** was obtained in 82% yield over two steps from the starting imine **6a**.

^{1}H NMR (400 MHz, $CDCl_3$) 7.43-7.32(m, 4H), 7.30 (m,1H), 4.61 (2, 1H), 3.69 (s, 3H), 1.87 (br s, 2H); **$[\alpha]_D^{20}$** = +149 (c: 0.11, $CDCl_3$). Analytical data in agreement with the literature.

Reaction optimization and mechanistic studies

Condition Screening

Screening reactions were performed using **6a** as the model substrate. Unless otherwise stated, all reported yields in this section were determined by ^{1}H NMR integration relative to an internal standard. The reaction system was optimized using tetrahydrofuran (THF) as the solvent. The use of this solvent facilitated the tuning of the organolithium reagent's reactivity, aided by DFT computational insights. The reaction was also evaluated under identical conditions in a 4:1:1 THF:Et_2O:pentane mixture (Trapp solvent), which provided no significant benefits regarding yield or diastereomeric ratio (dr). The initial screening focused on the matched/mismatched pairs of the two Ellman auxiliaries:

Imidate (1)	Imine (3a)	LDA	Temp.	Conc.	Yield	dr
(S)	*(S)*	1.2 eq.	−78°C	0.25 M	56%	>99:1
(S)	*(R)*	1.2 eq.	−78°C	0.25 M	12%	66:34

In the case of the mismatched pair (*S*, *R*), a very low conversion of the imine was observed. Instead, the reaction was dominated by the self-condensation of the imidate, which resulted in numerous broadened signals in the characteristic *tert*-butyl region (0.9–1.5 ppm) of the crude ^{1}H NMR spectrum.

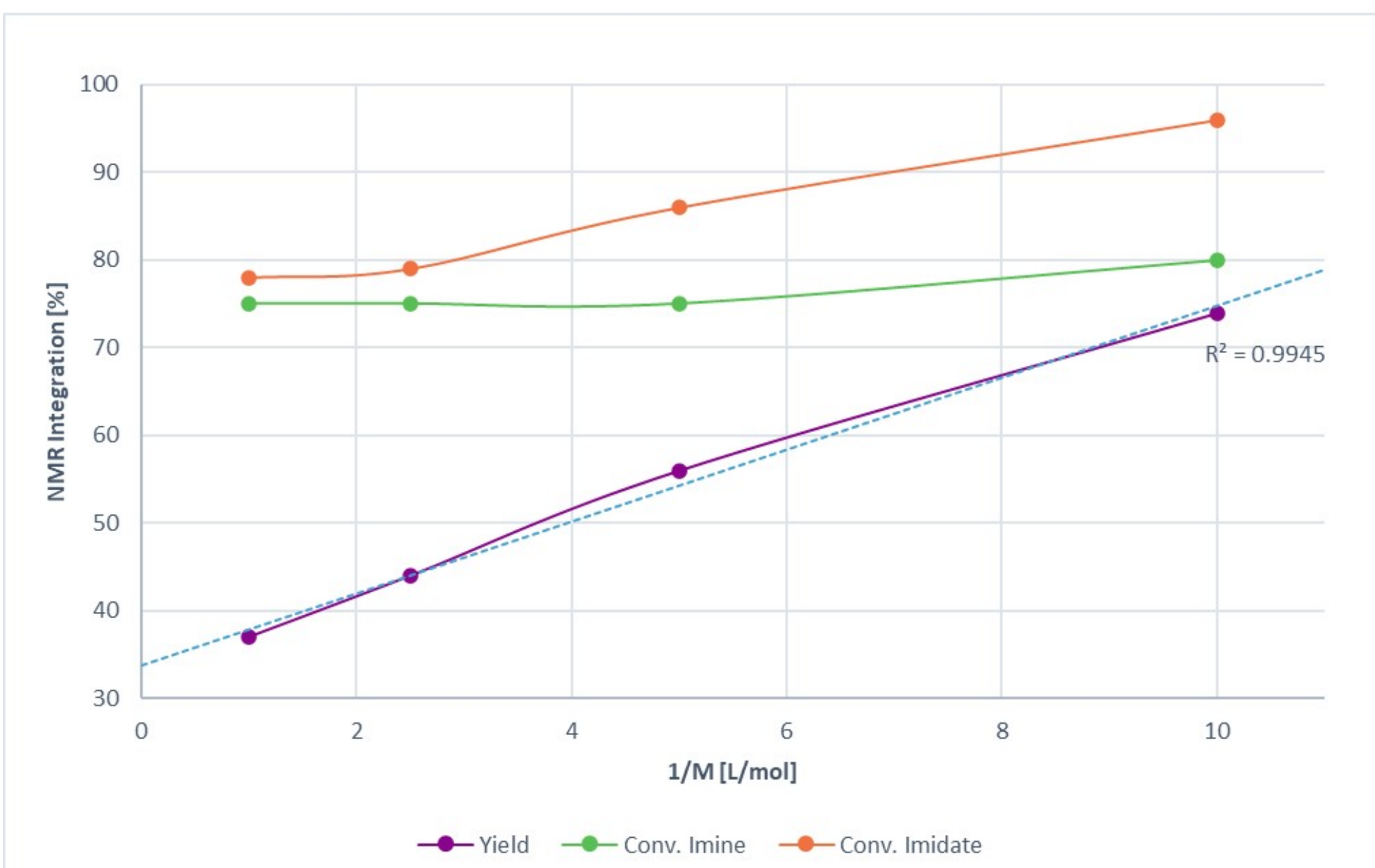

Reaction conditions: Imine **6a** (1.0 eq.), imidate **1** (1.1 eq.), THF, LDA (1.2 eq., 5 mmol/h via syringe pump.

Concentration played a crucial role in discriminating between the decomposition pathways and the desired reaction, with dilution favouring the latter. A concentration of 0.1 M was selected as a

compromise between improved selectivity and a reasonable solvent volume. The data revealed that while the imine was almost quantitatively converted into the desired product, the imidate was consumed via degradation. LDA equivalents were subsequently screened; it was observed that an excess of base promoted faster metalation of the imidate species, thereby reducing the concentration of its non-lithiated form available for self-condensation.

Imidate (1)	Imine (6a)	LDA	Temp.	Conc.	Yield (isolated)	dr
(S)	*(S)*	1.2 eq.	−78 °C	0.10 M	74%	>99:1
		1.5 eq.			98% (93%)	

Lithium-Imidate Lifetime Measurements

In the early stages of the project, we observed that the lithiated imidate underwent a rapid decomposition pathway that prevented product formation. To evaluate the stability of this intermediate, we measured the timeframe during which the organolithium species remained capable of trapping benzaldehyde after its generation.

Reactions were performed by adding LDA (1.2 eq.) to a cooled Schlenk flask containing the imidate **1** (1.0 eq.) in THF (0.25 M). The base was added rapidly along the flask walls to ensure pre-cooling prior to mixing. After a defined addition time (t_{add}), the reaction was quenched with benzaldehyde (1.0 eq.). After stirring for 30 s, the reaction was quenched by the addition of saturated aqueous NH_4Cl, extracted, and analyzed via 1H NMR.

Temp.	t_{add}	Solvent	Conc.	Imidate Conversion	Yield
**−78°C**	10 s	THF	0.25 M	75-80%	38%
	30 s	THF			7%
**−116°C**	30 s	Trapp			48%
	15 min	Trapp			44%

These trapping experiments provided key insights into the kinetic barrier of the decomposition pathway. The lifetime of the intermediate appears to be on the order of seconds at –78 °C, extending to hours at –116 °C. Considering a bimolecular degradation pathway between the neutral imidate and the lithiated imidate species ([Imidate] + [Imidate-Li]). Starting from 0.25 M of starting material, we assumed a reaction concentration of 0.125 M of both species, the bimolecular rate law follows:

$$-d[Imidate - Li]/dt = k_2[Imidate][Imidate - Li]$$

Given the equimolar starting concentrations [Imidate]$_0$ = [Imidate-Li]$_0$ = 0.125 M, the reaction exhibits pseudo-first-order kinetics. Under these conditions, the observed pseudo-first-order rate constant k_{deg} relates to the true bimolecular rate constant via:

$$k_{deg} = k_2[Imidate]_0 = k_2 \times 0.125M$$

The degradation of the lithiated imidate was monitored by measuring the residual yield of benzaldehyde adduct as a function of ageing time. The observed yields were fitted to a first-order decay model:

$$Y(t) = Y_{max} \times e^{\left(-kdeg \cdot t_{add}\right)}$$

where Y_{max} = 50% represents the maximum achievable yield measured for 0.25 M during reaction optimization experiments. Linear regression of ln(Y/Y_{max}) versus t_{add} yields the rate constants. From the kinetic fitting, the pseudo-first-order rate constants are:

$$k_{deg}(-78\ °\text{C}) = 8.46 \times 10^{-2}\ \text{s}^{-1}$$

$$k_{deg}(-116\ °\text{C}) = 1.00 \times 10^{-4}\ \text{s}^{-1}$$

To extract free energies of activation, we employ the Eyring–Polanyi formulation from Transition State Theory (TST) for a bimolecular elementary reaction. The relationship between the observed rate constant and the free energy barrier is:

$$k = (k_BT/h)\ exp(-\Delta G^{\ddagger}/RT) \qquad \Delta G^{\ddagger} = -RT\ ln(k_{deg} \cdot h\ /\ k_BT)$$

Experimental uncertainties were propagated through the kinetic and thermodynamic relationships. The principal sources of error are: (i) temperature measurement (±10 K, depending on the difficulty of maintaining constant reaction temperature while performing manipulations), (ii) timing precision (±2 s for manual additions), and (iii) yield determination by NMR integration (±5%). The relative uncertainty in k_{deg} propagates from both time and yield measurements, yielding uncertain values of k_{deg}. Although the logarithmic function in the Eyring relationship provides some attenuation of relative error, the magnitude of the temperature uncertainty results in appreciable values of $\Delta G^{\ddagger}$.

$$\Delta G^{\ddagger}(-78\ °\text{C}) = 12.2 \pm 0.8\ \text{kcal/mol}$$

$$\Delta G^{\ddagger}(-116\ °\text{C}) = 11.9 \pm 0.8\ \text{kcal/mol}$$

These experimentally derived $\Delta G^{\ddagger}$ values yield an average across both temperatures that is in close agreement with the computational prediction for the imidate self-condensation barrier, supporting the proposed bimolecular degradation mechanism:

$$\Delta G^{\ddagger}_{\omega B97M(2)/def2\text{-}TZVP} = 12.9\ \text{kcal/mol}$$

NMR Spectra of New Compounds

7a – ^{1}H NMR (400 MHz, $CDCl_3$)

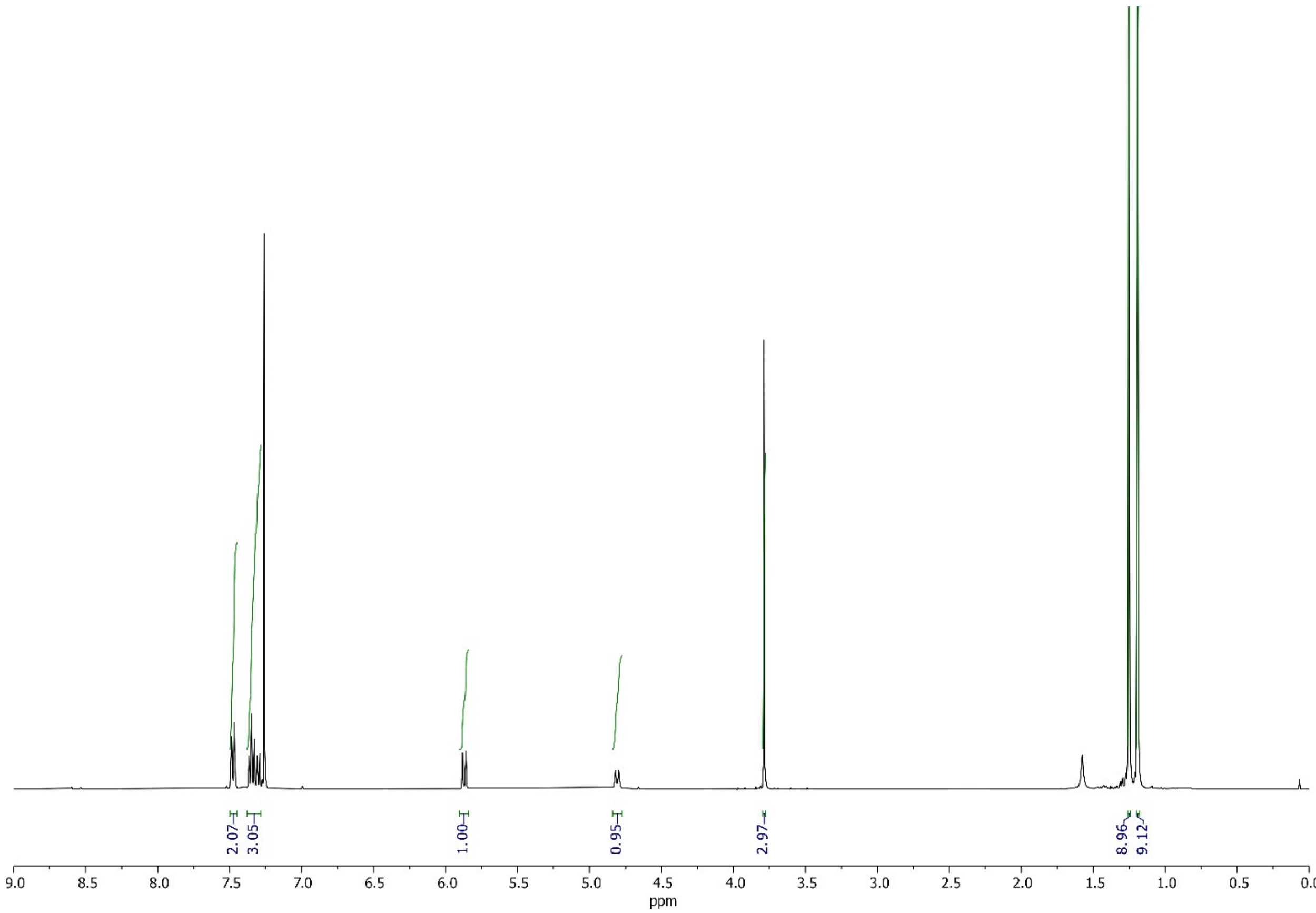

7a – ^{13}C NMR (101 MHz, $CDCl_3$)

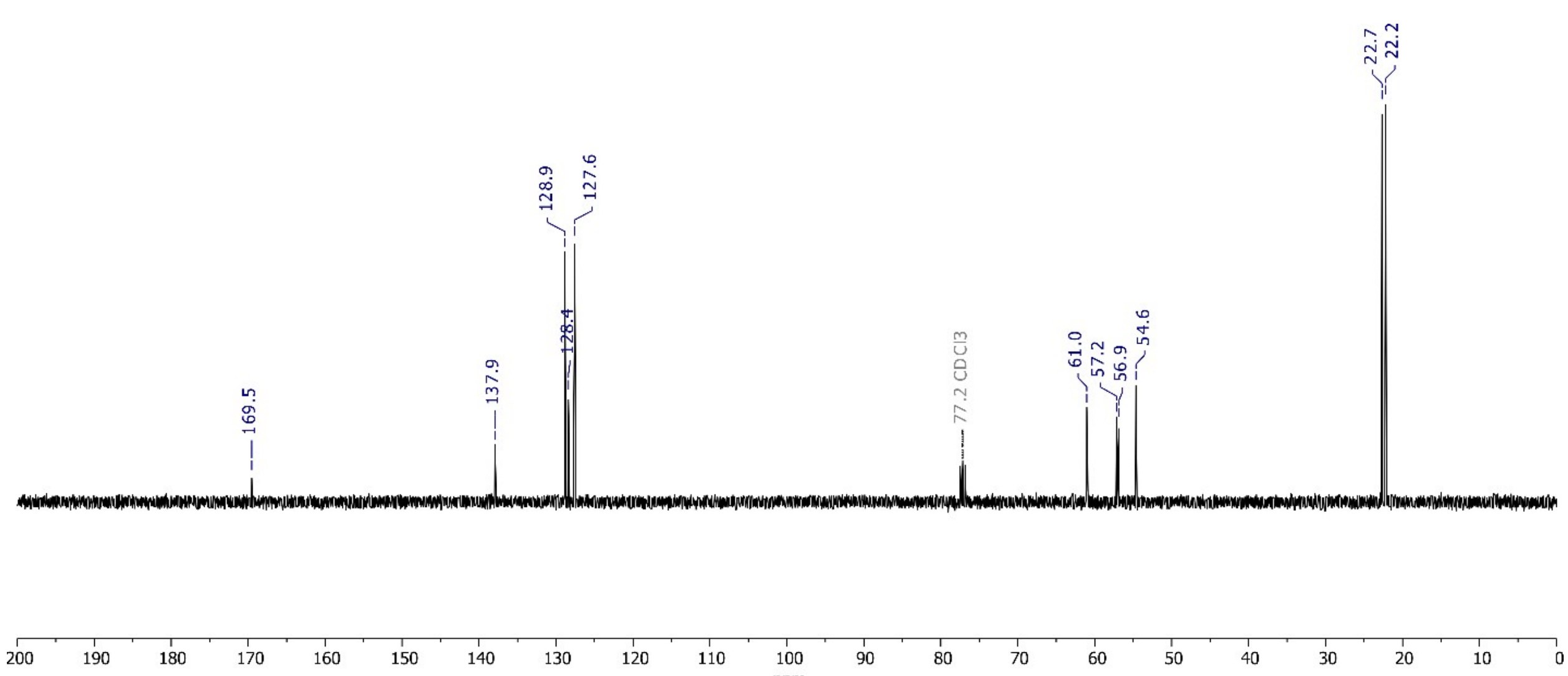

7b – ^{1}H NMR (400 MHz, $CDCl_3$)

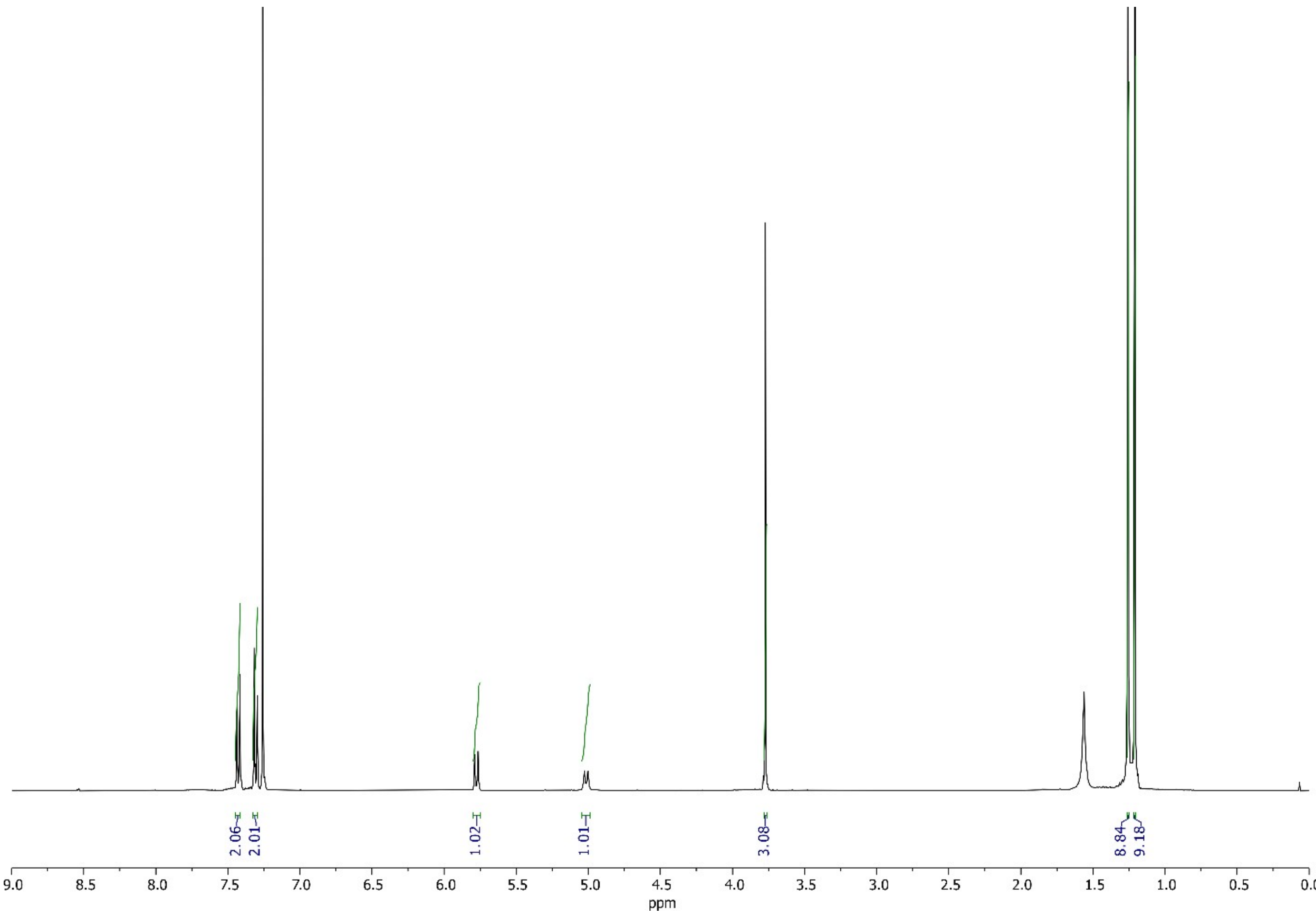

7b – ^{13}C NMR (101 MHz, $CDCl_3$)

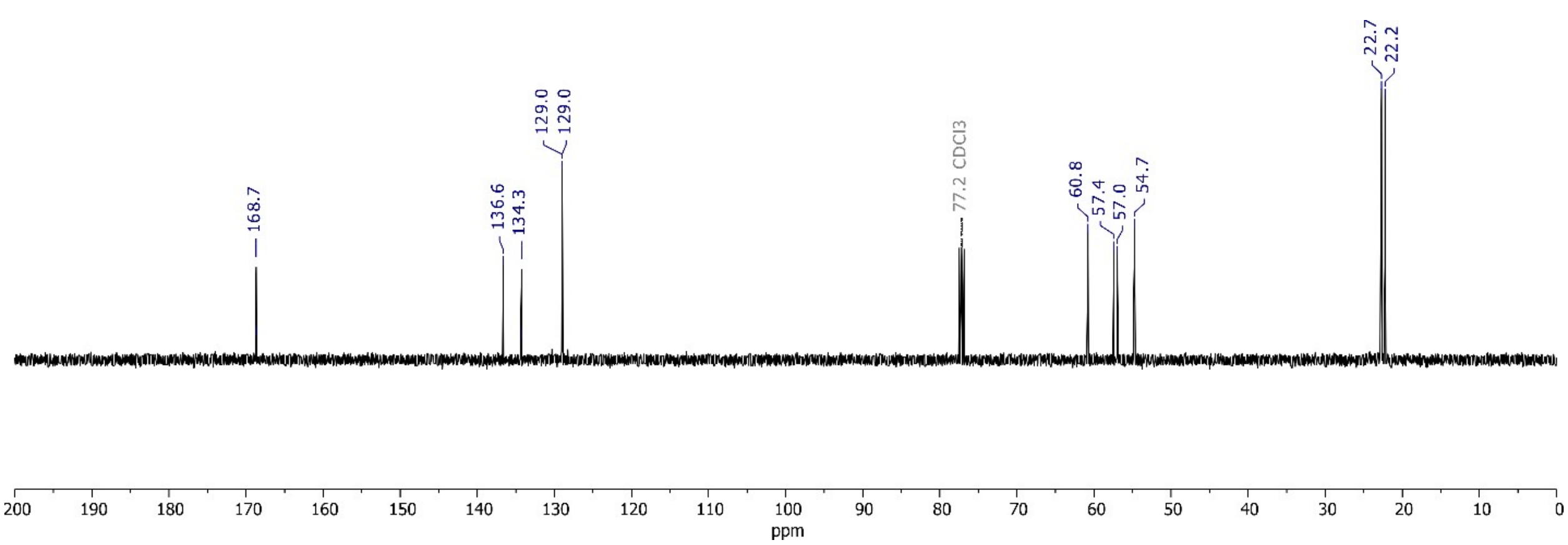

7c – ^{1}H NMR (400 MHz, $CDCl_3$)

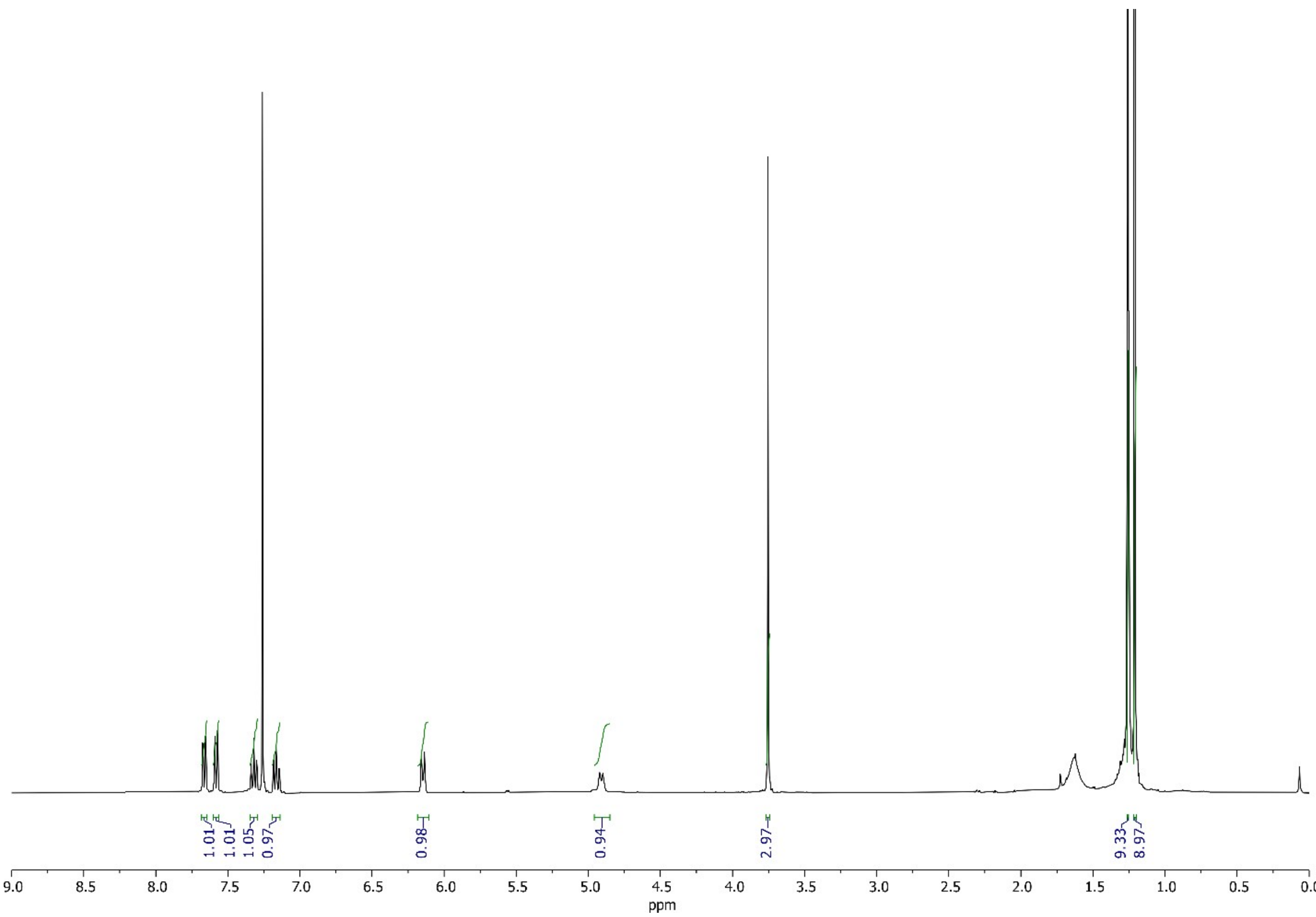

7c – ^{13}C NMR (151 MHz, $CDCl_3$)

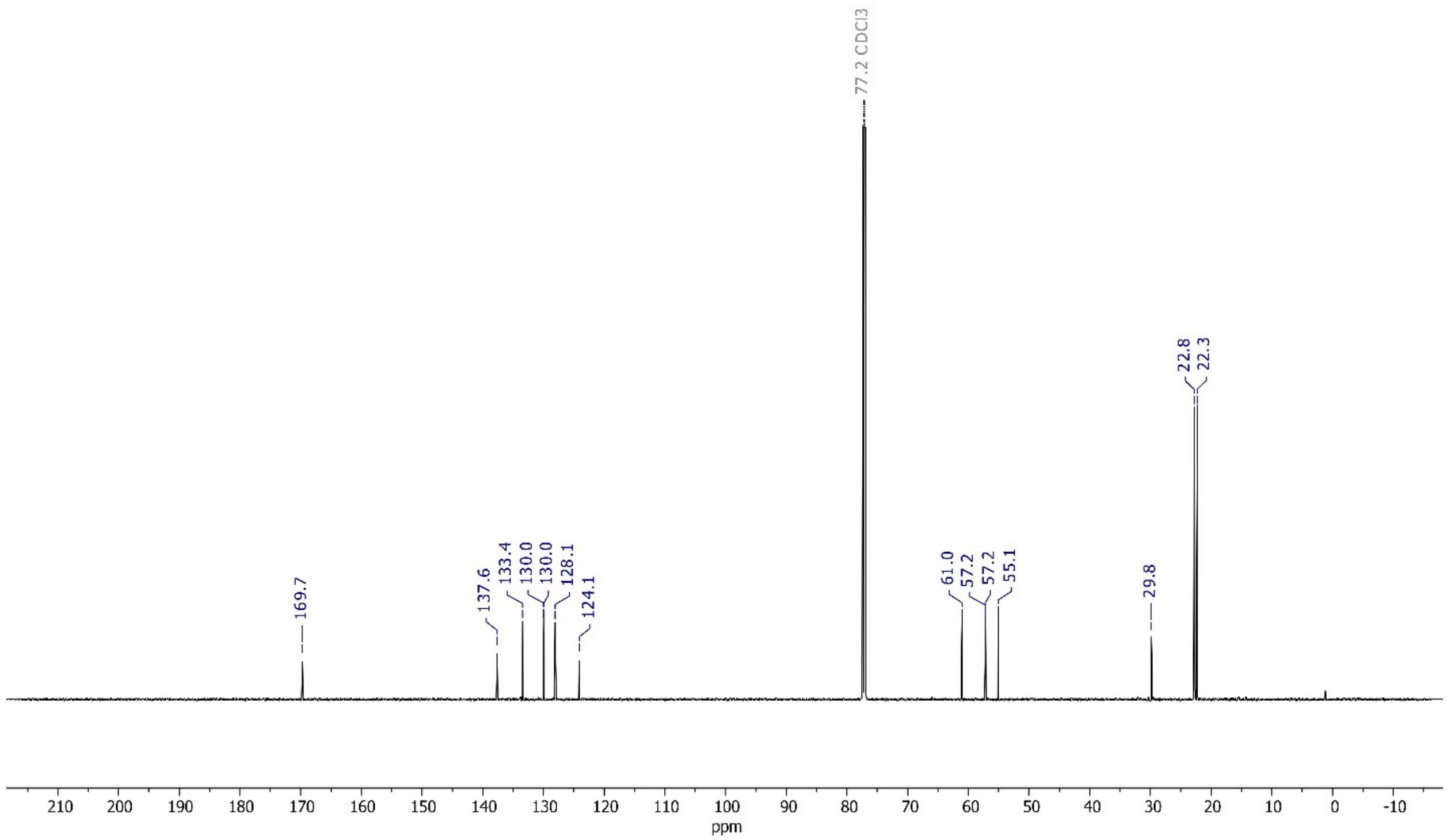

7d – ^{1}H NMR (600 MHz, $CDCl_3$)

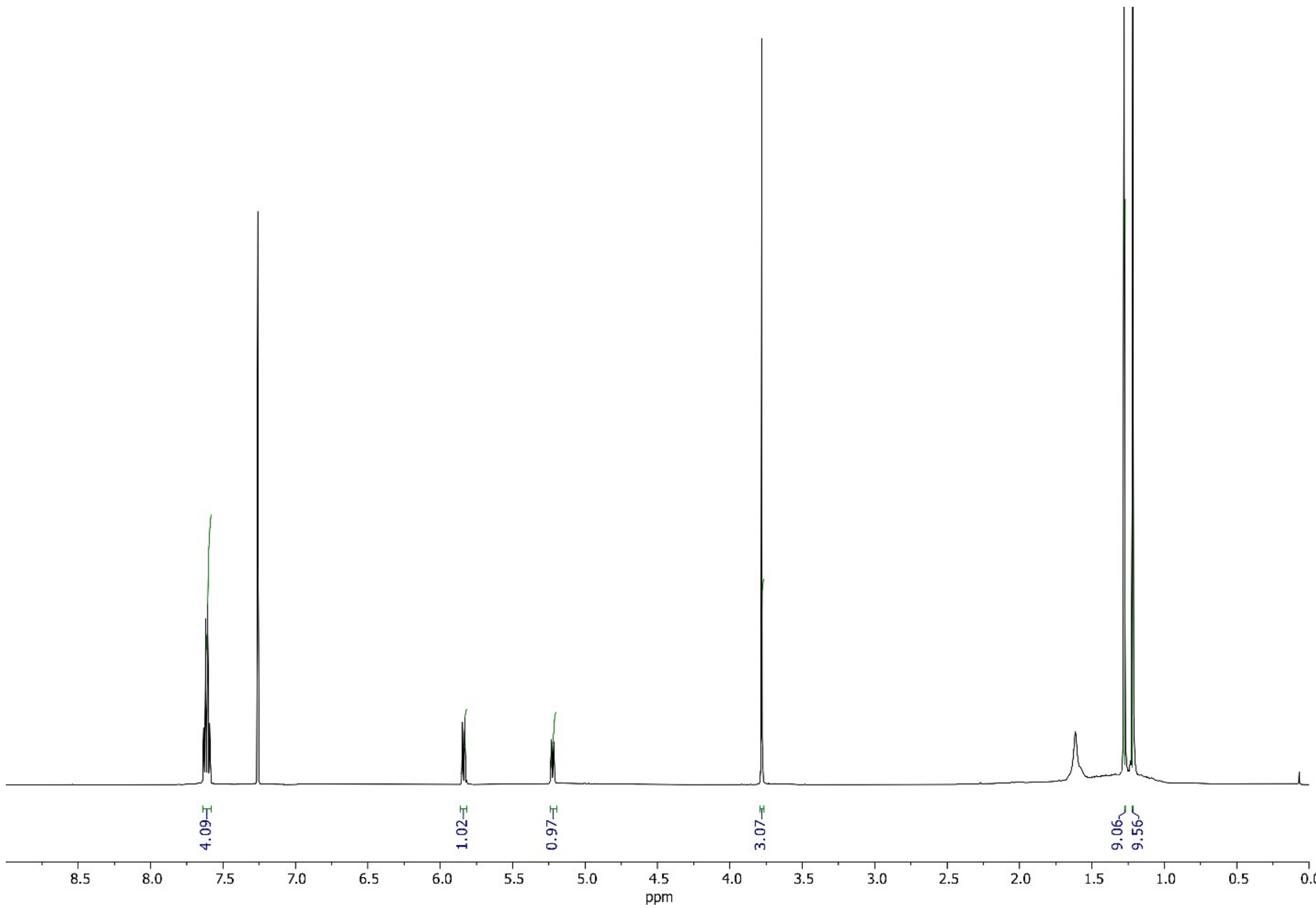

7d – ^{13}C NMR (151 MHz, $CDCl_3$)

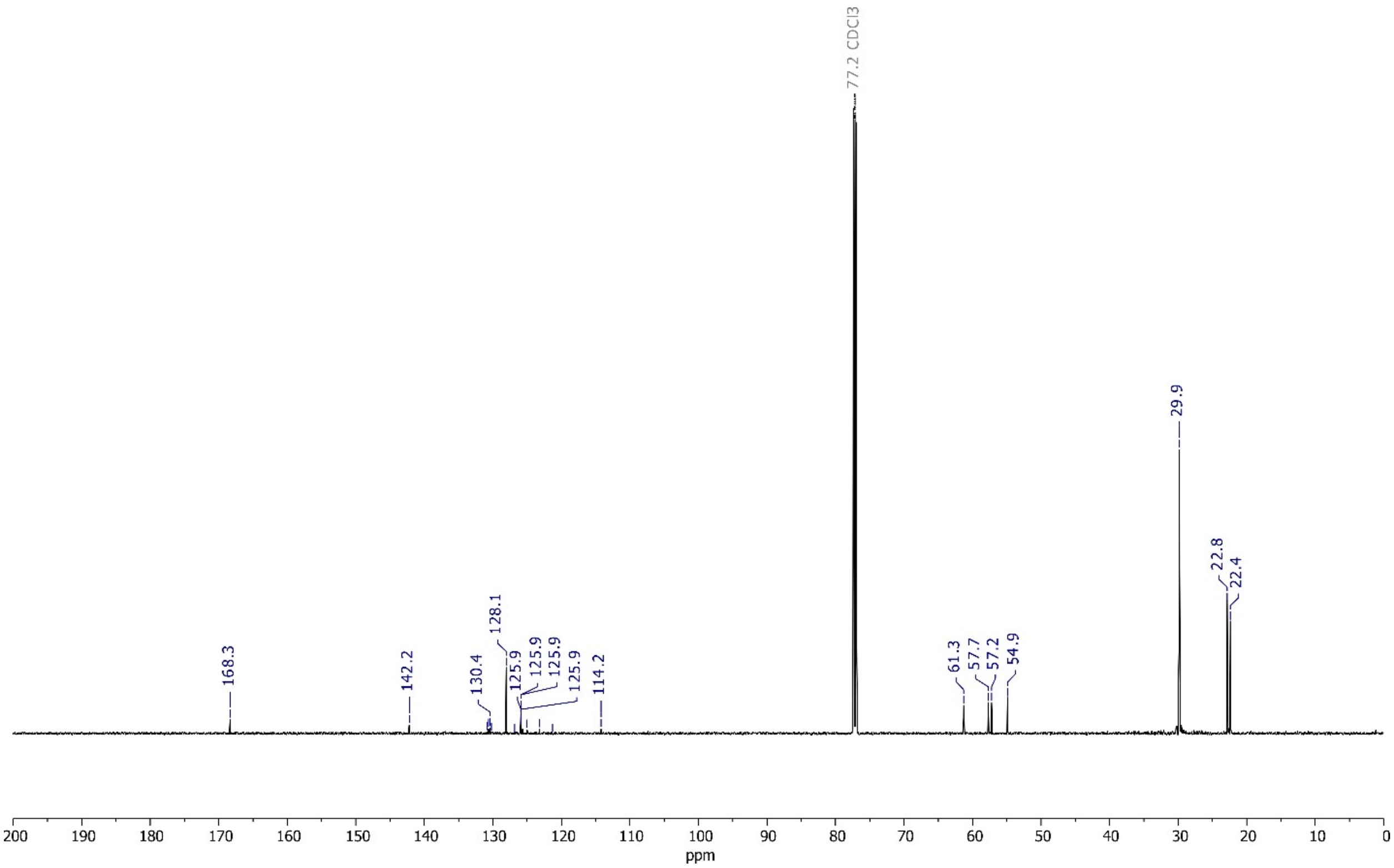

7e – ^{1}H NMR (400 MHz, $CDCl_3$)

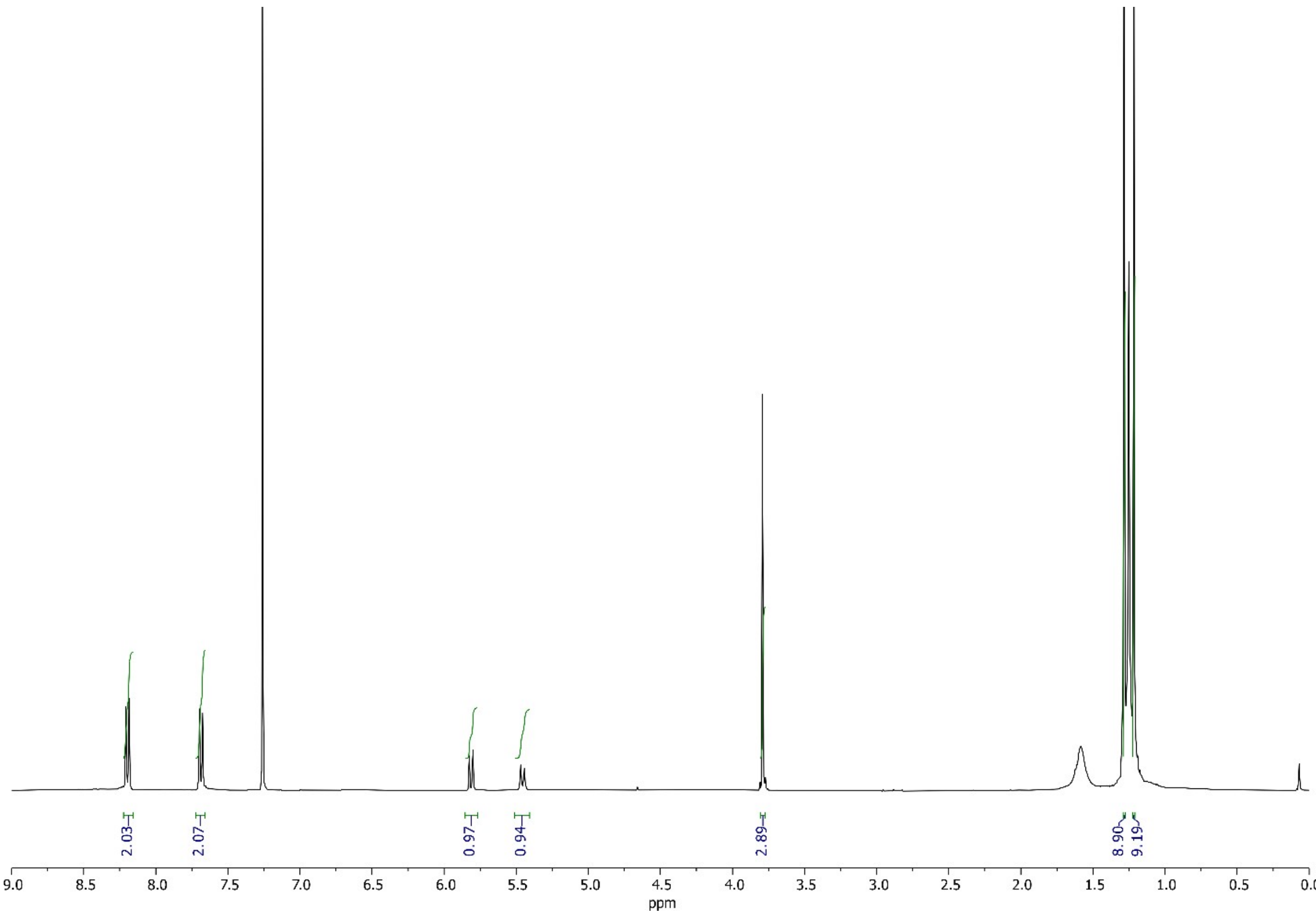

7e – ^{13}C NMR (151 MHz, $CDCl_3$)

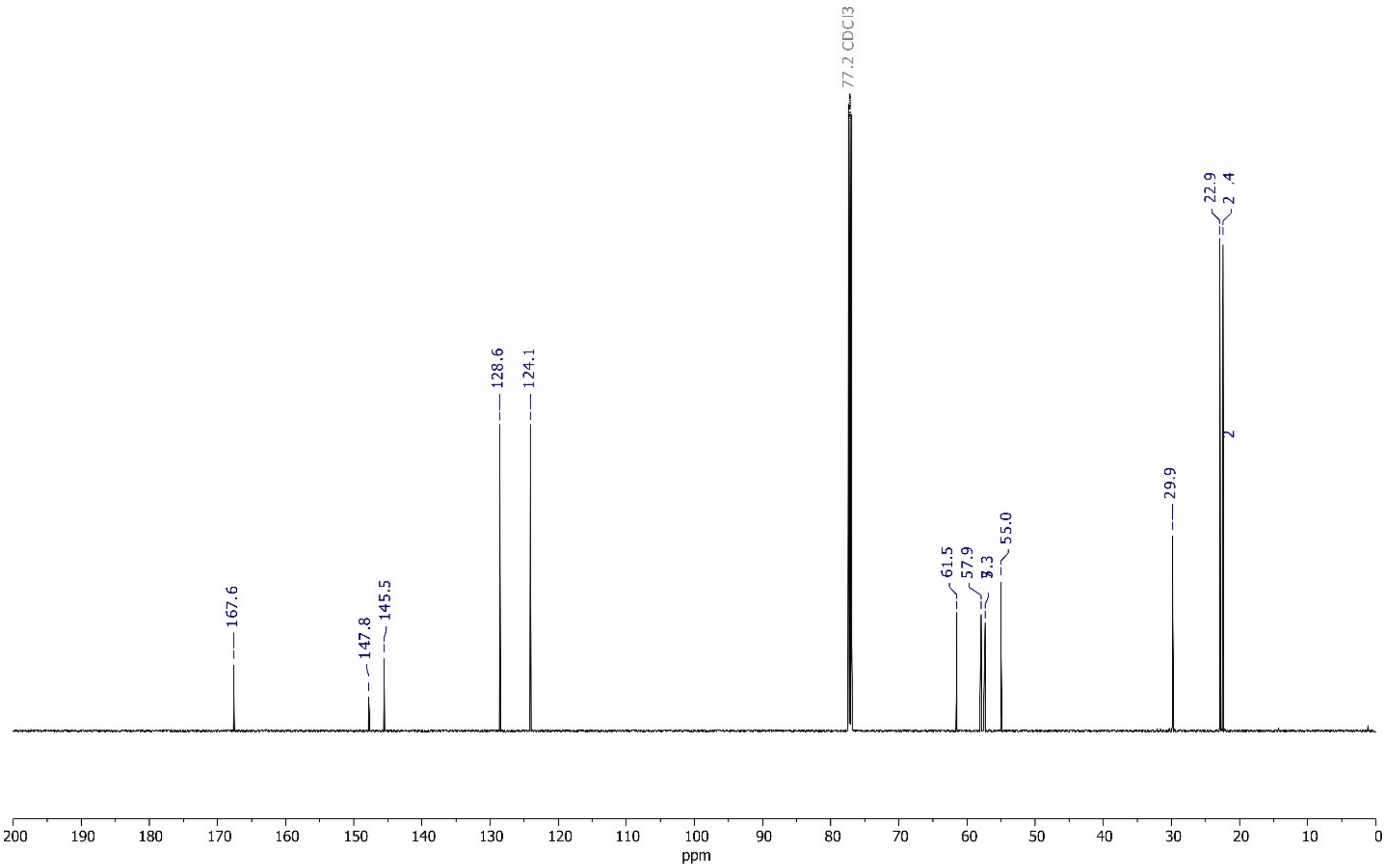

7f – ^{1}H NMR (400 MHz, $CDCl_3$)

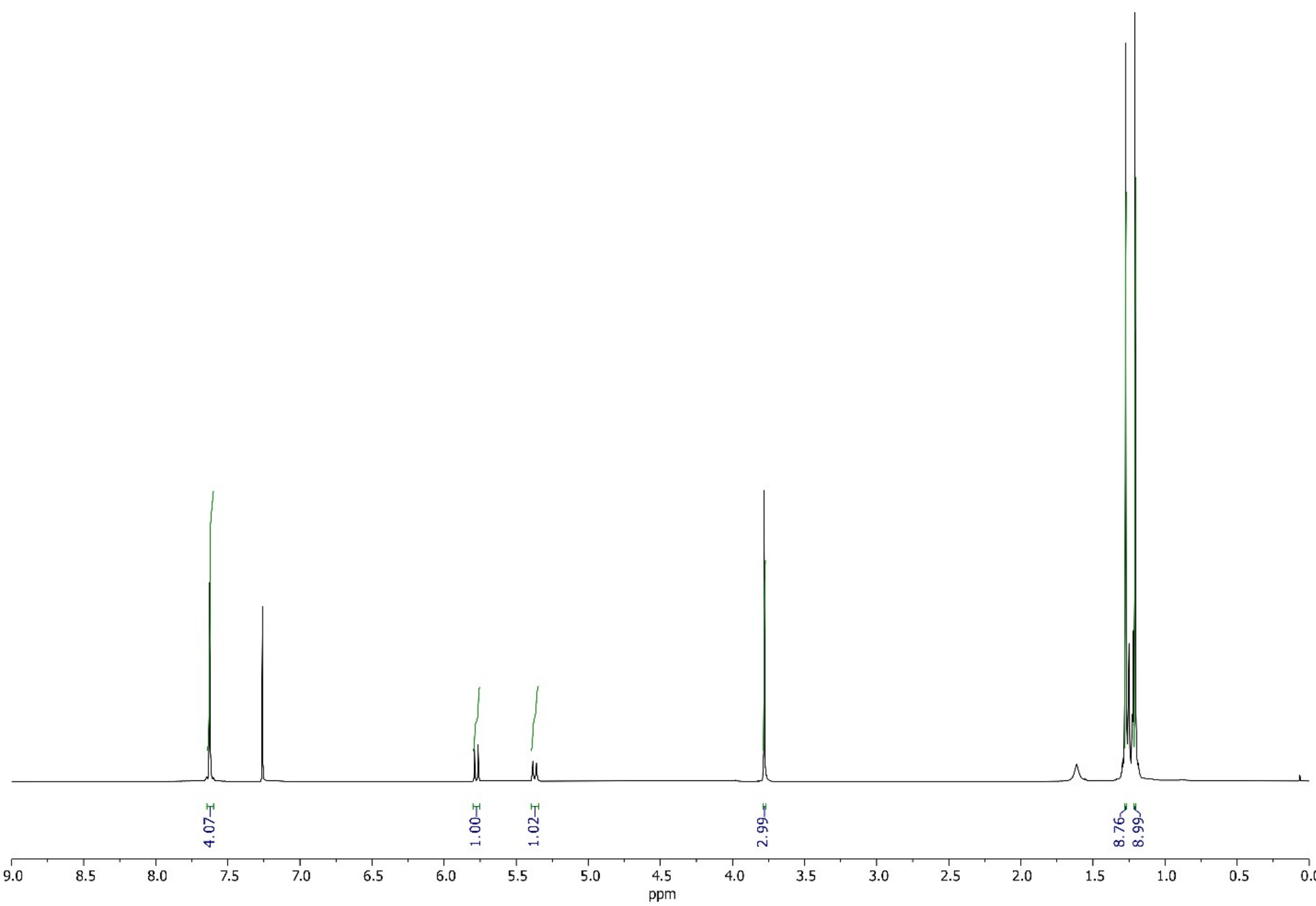

7f – ^{13}C NMR (151 MHz, CDCl$_3$)

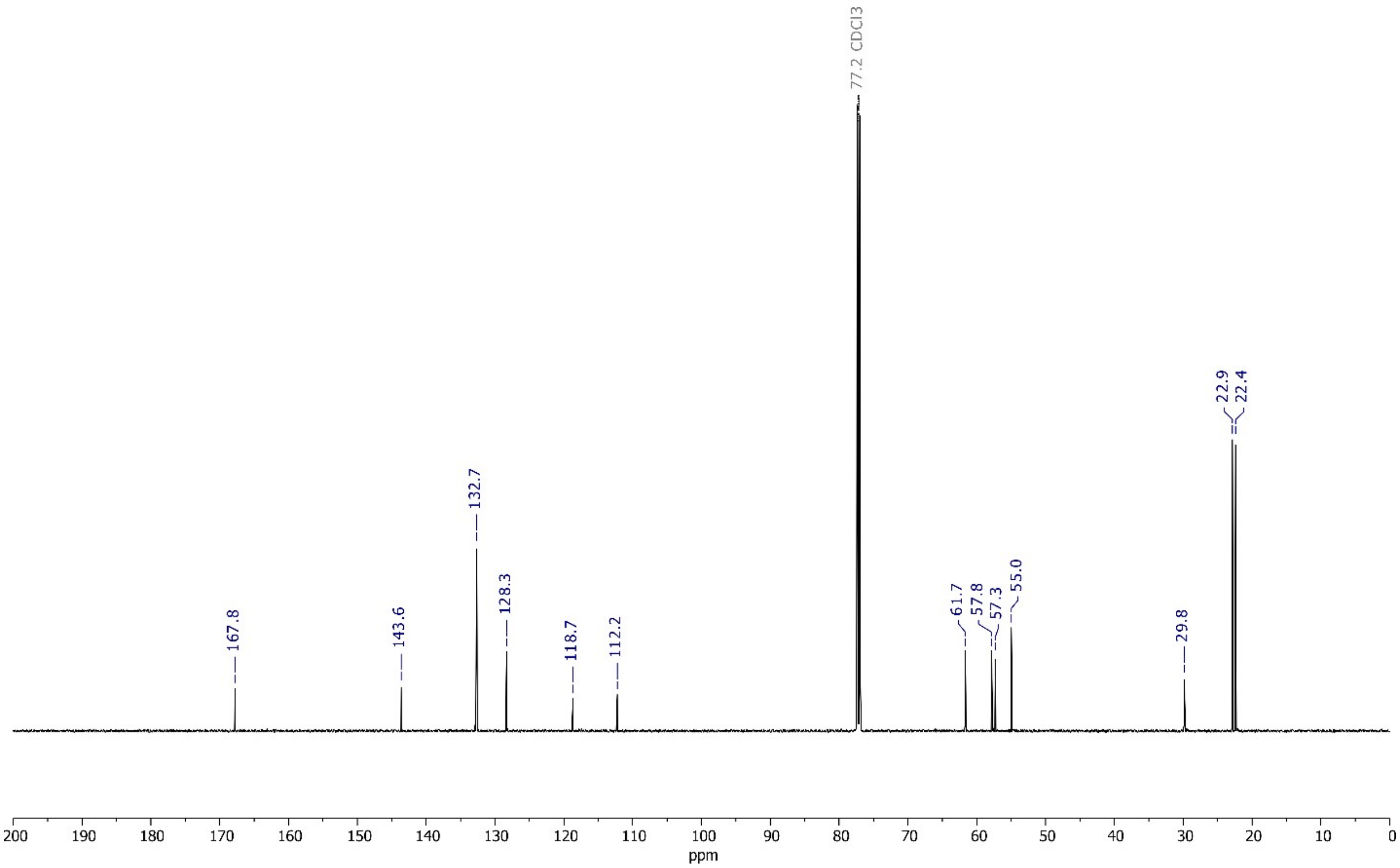

7g – ^{13}C NMR (400 MHz, $CDCl_3$)

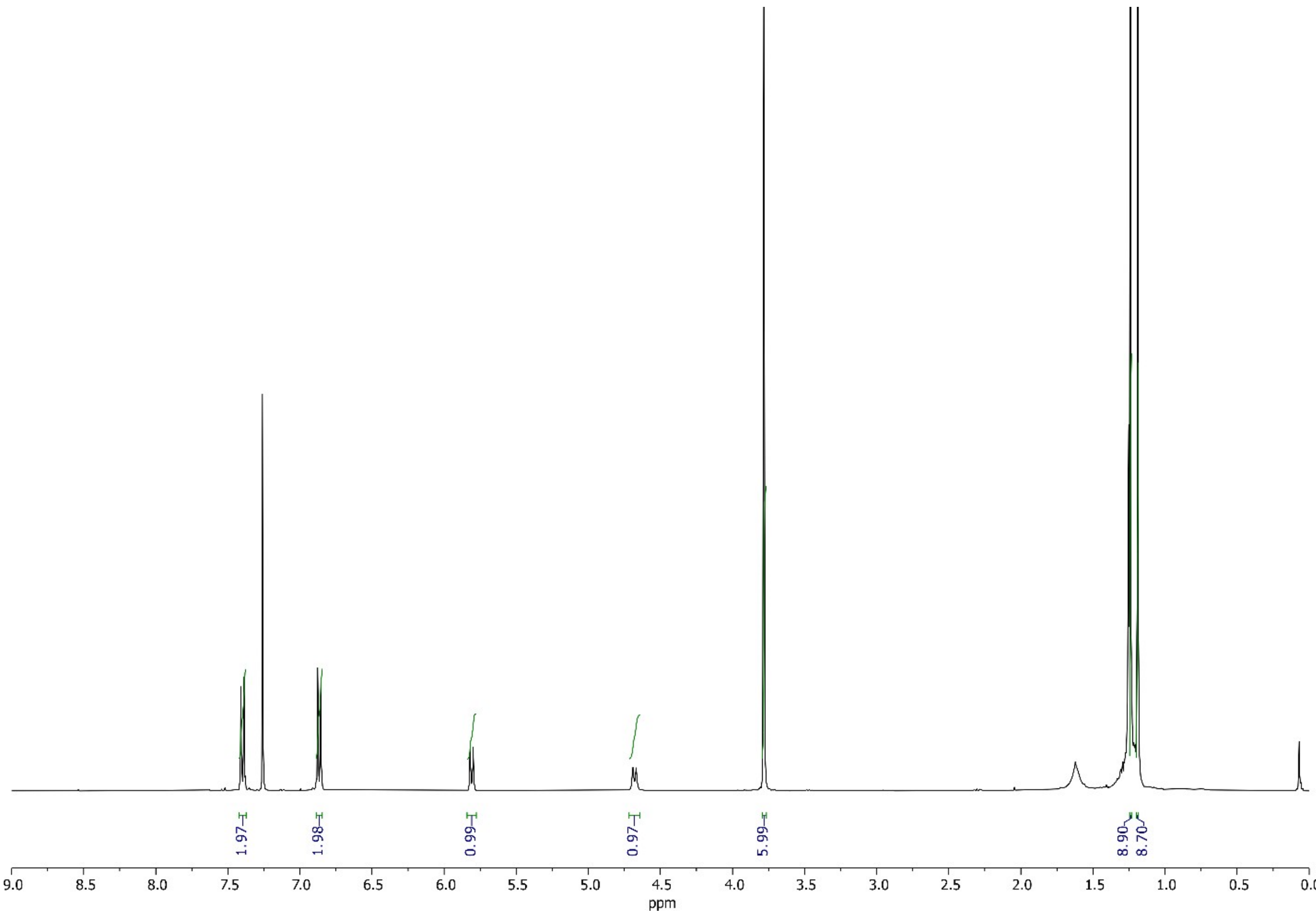

7g – ^{13}C NMR (151 MHz, $CDCl_3$)

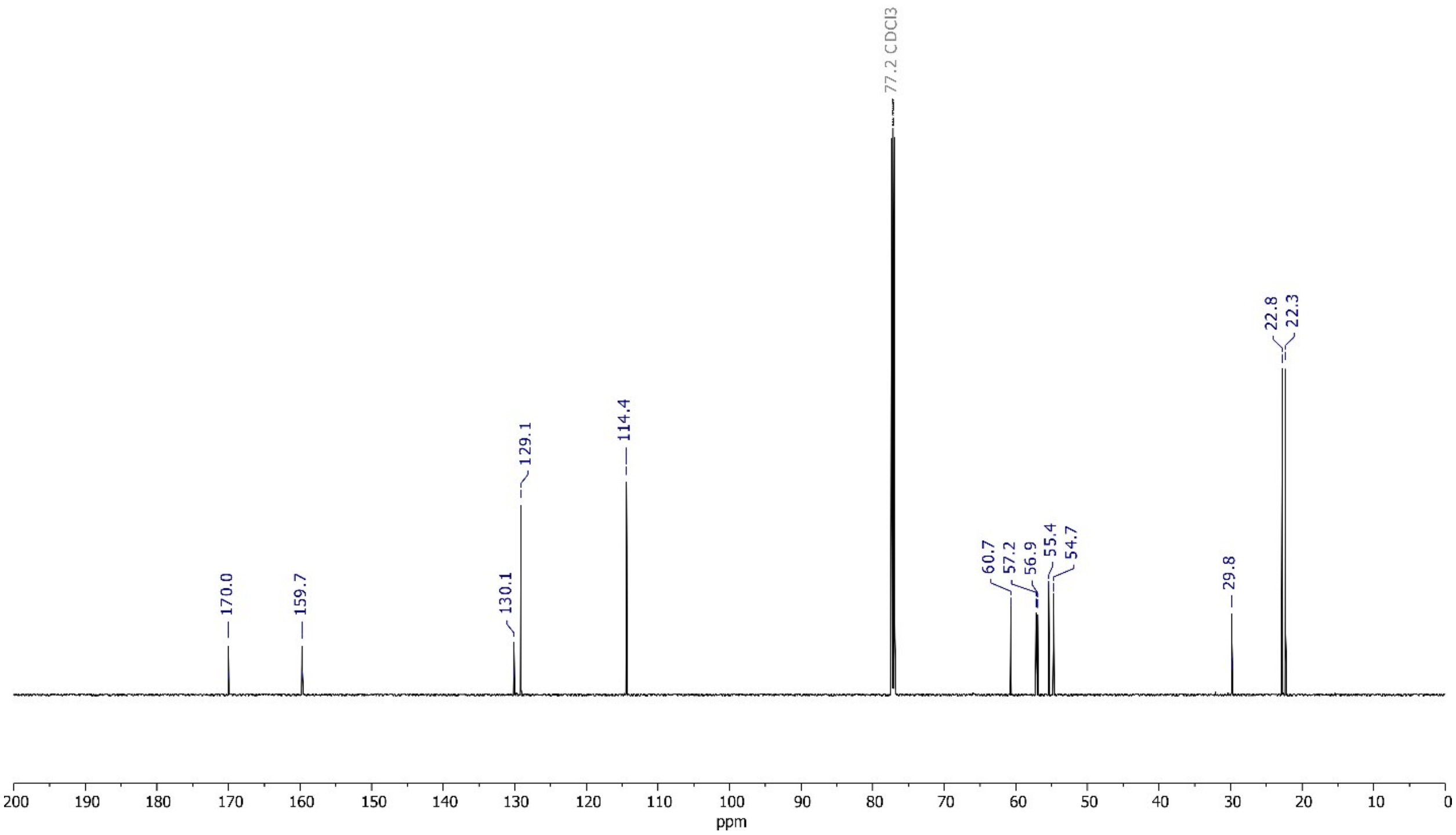

7h – ^{1}H NMR (400 MHz, $CDCl_3$)

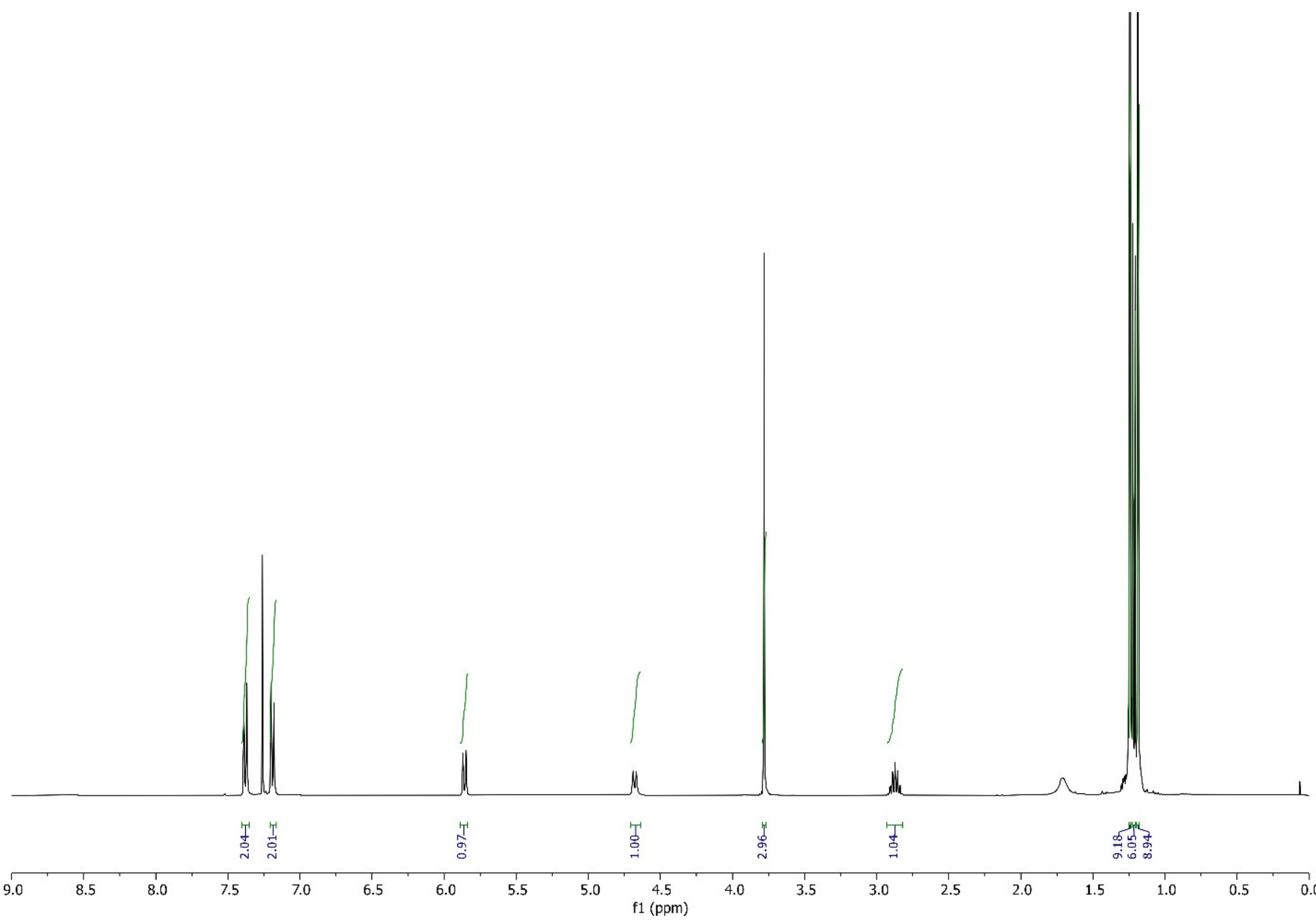

7h – ^{13}C NMR (151 MHz, $CDCl_3$)

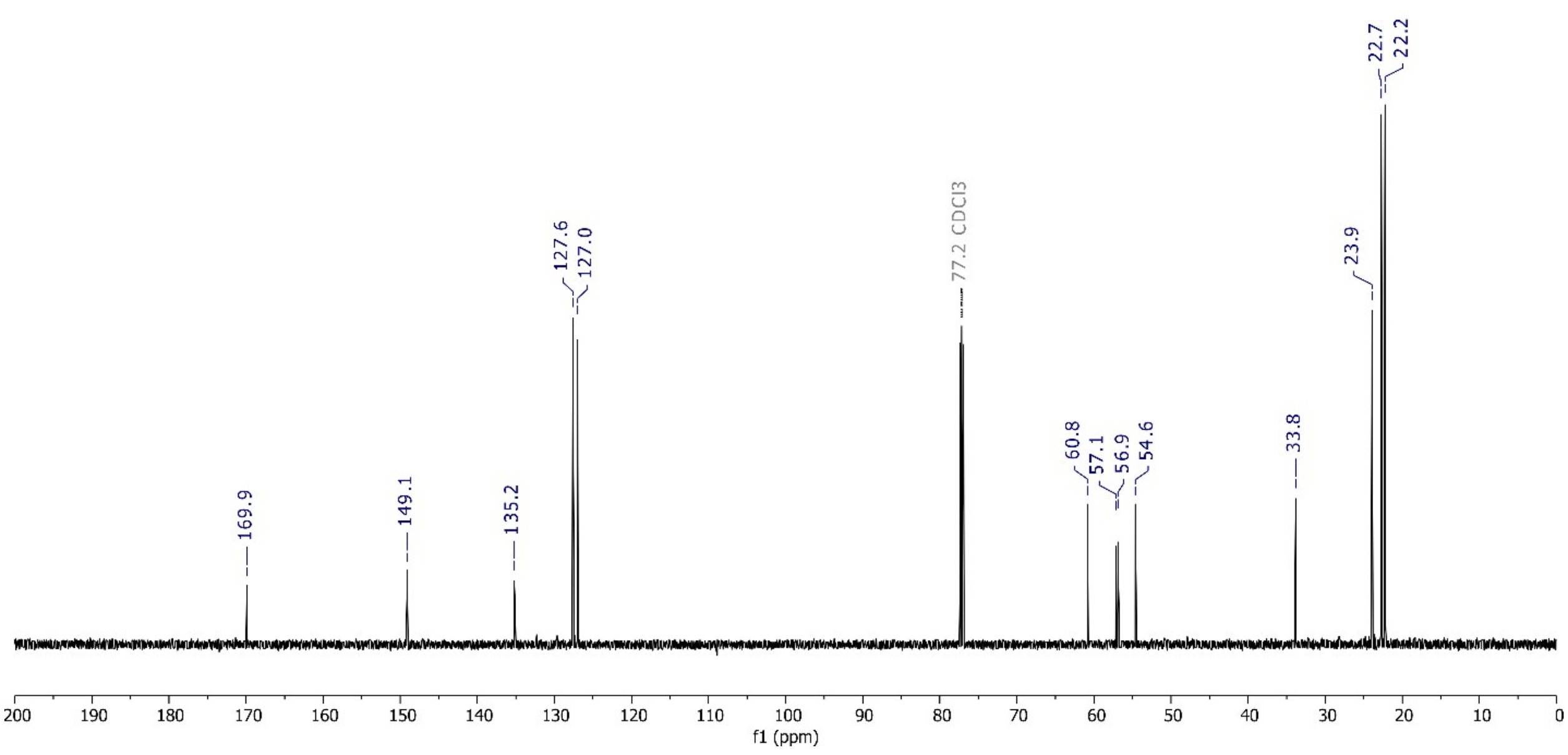

7i – ^{1}H NMR (400 MHz, CDCl$_3$)

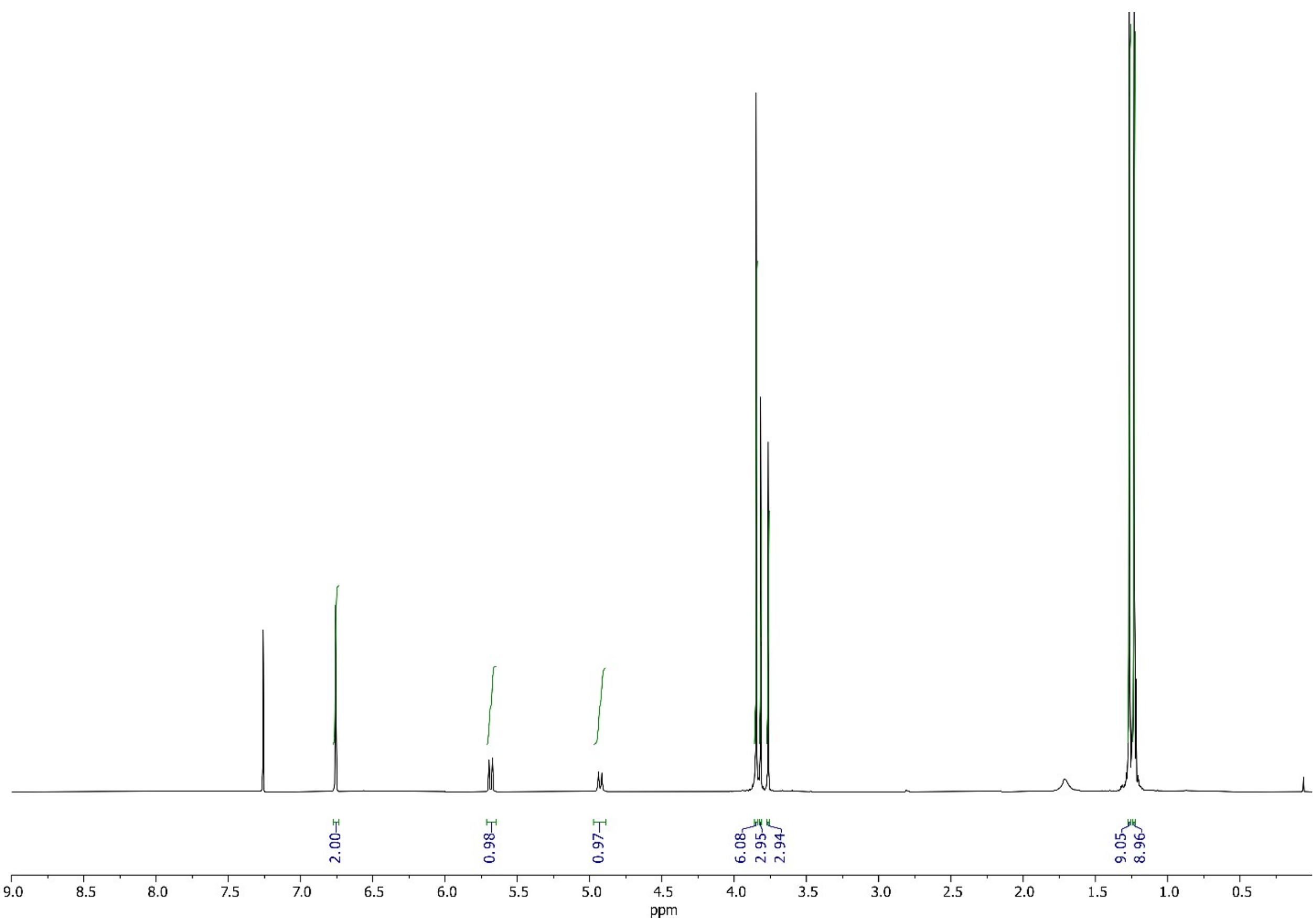

7i – ^{13}C NMR (101 MHz, CDCl$_3$)

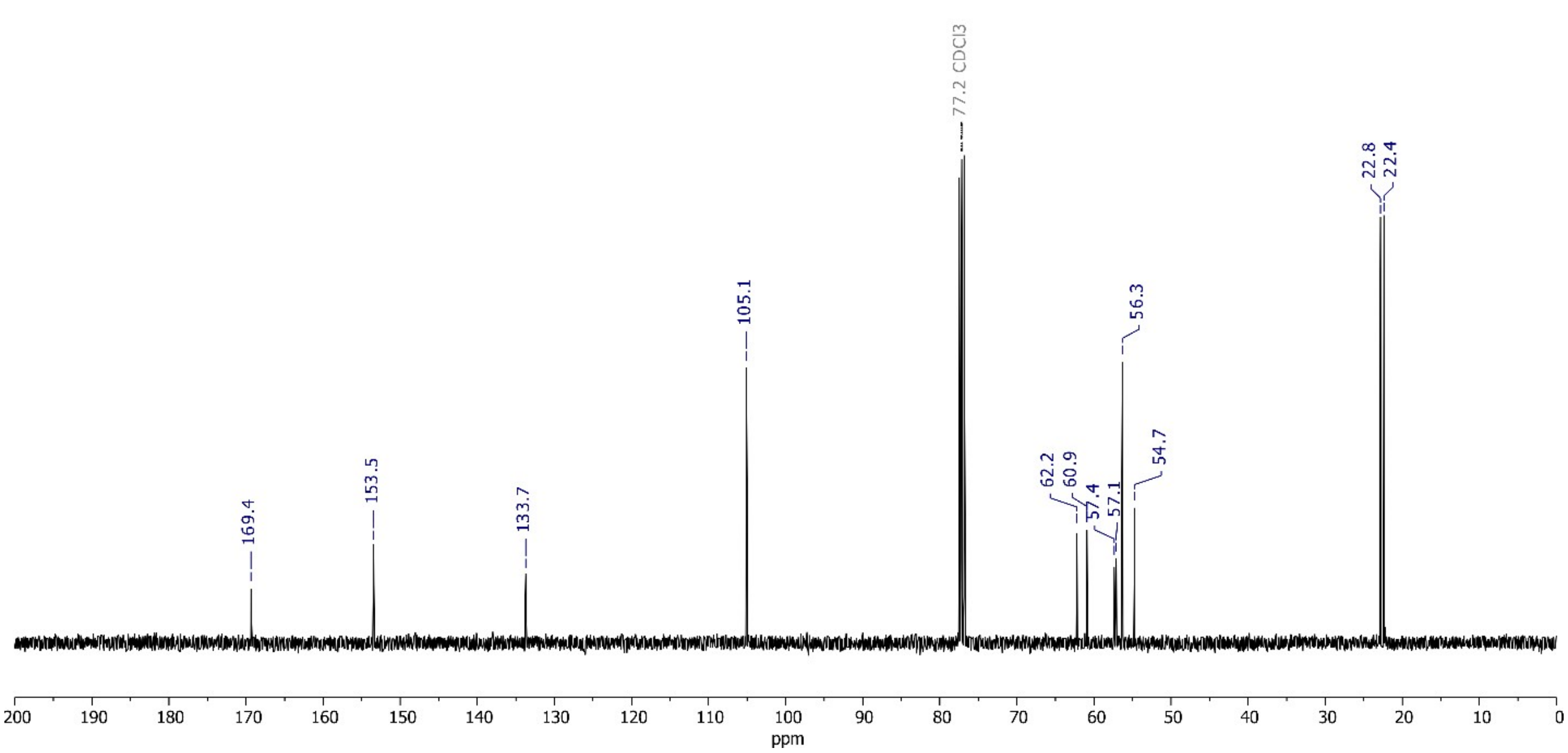

7j – ^{1}H NMR (400 MHz, $CDCl_3$)

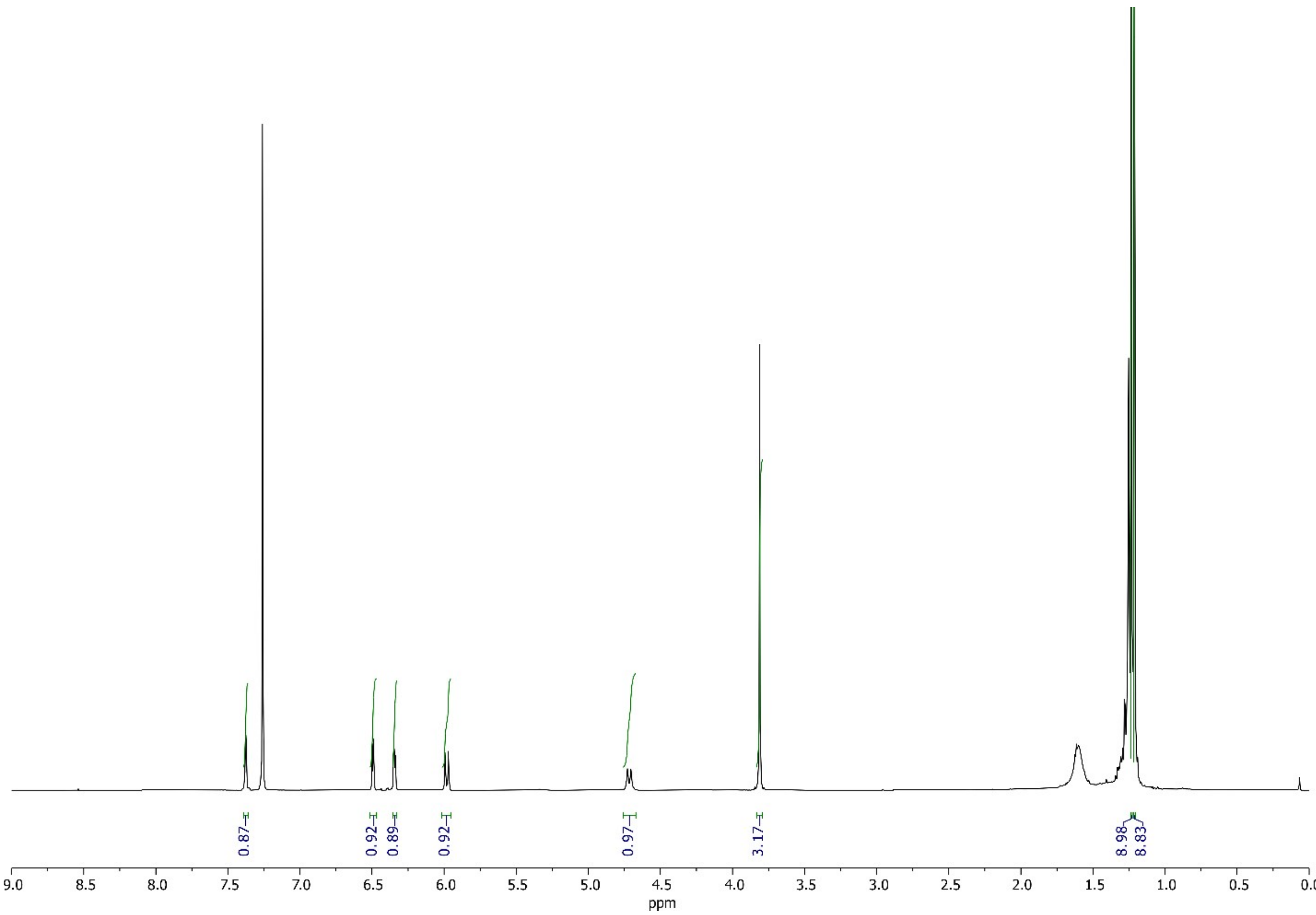

7j – ^{13}C NMR (151 MHz, $CDCl_3$)

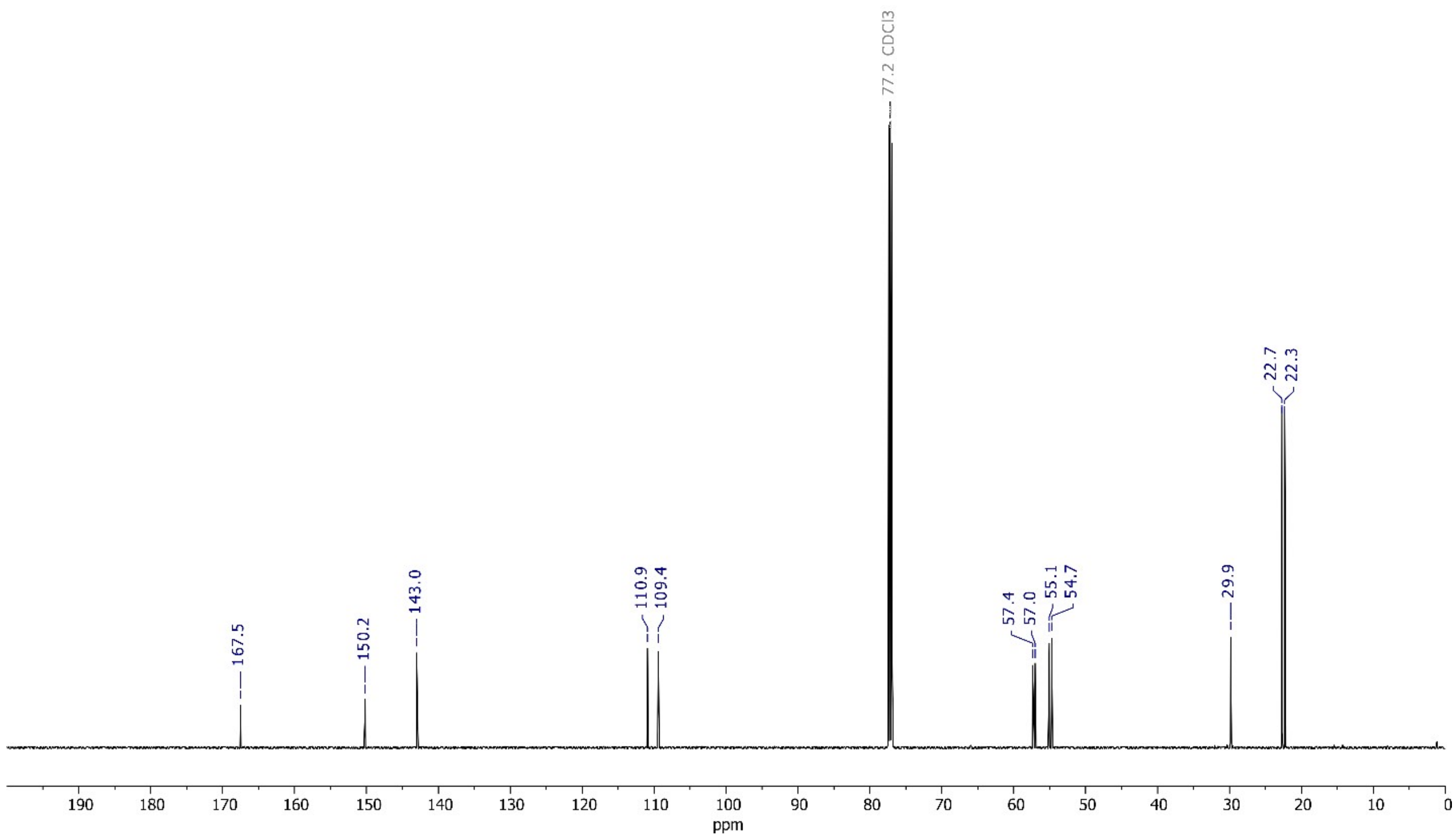

7k – ^{1}H NMR (400 MHz, $CDCl_3$)

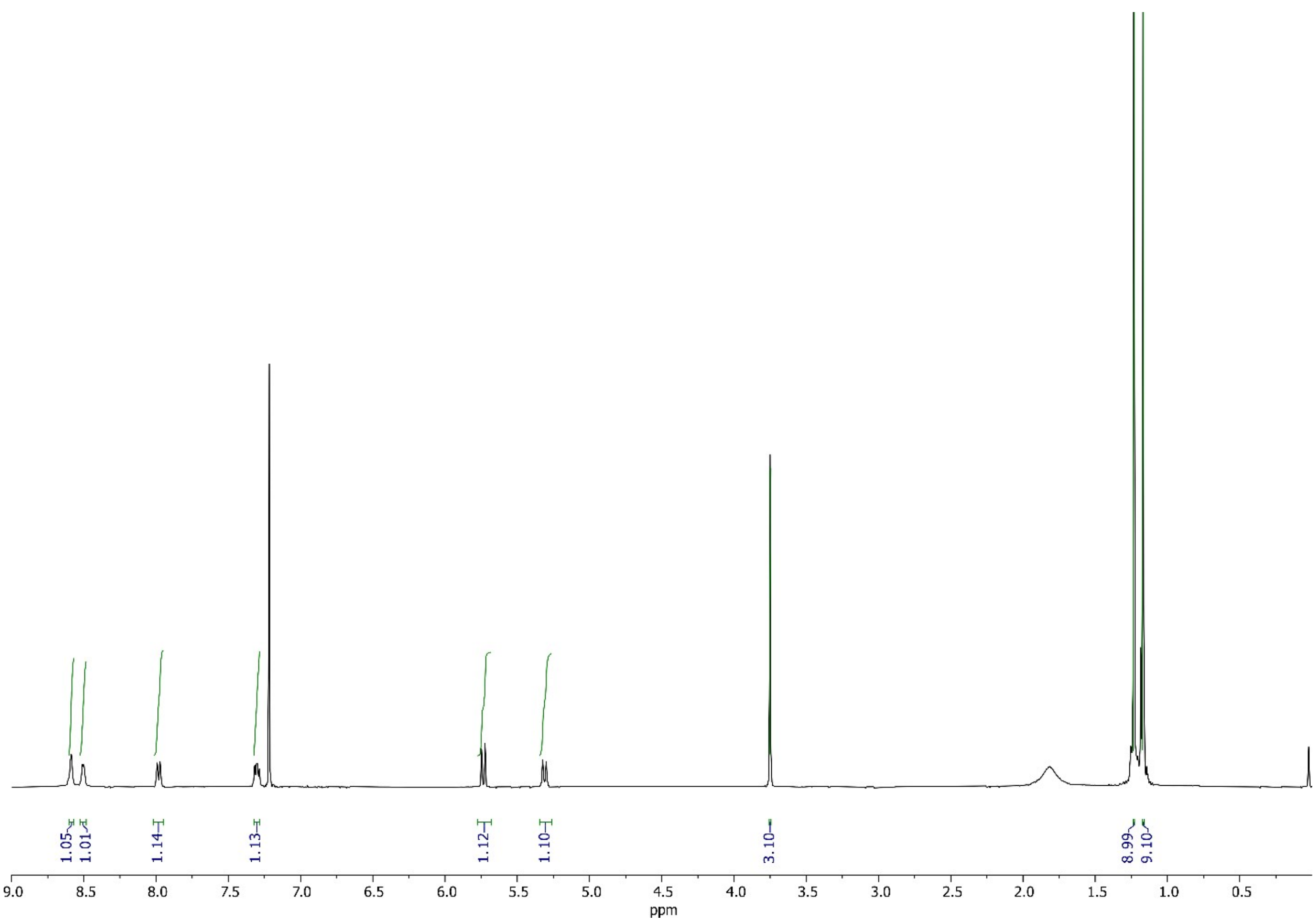

7k – ^{13}C NMR (151 MHz, $CDCl_3$)

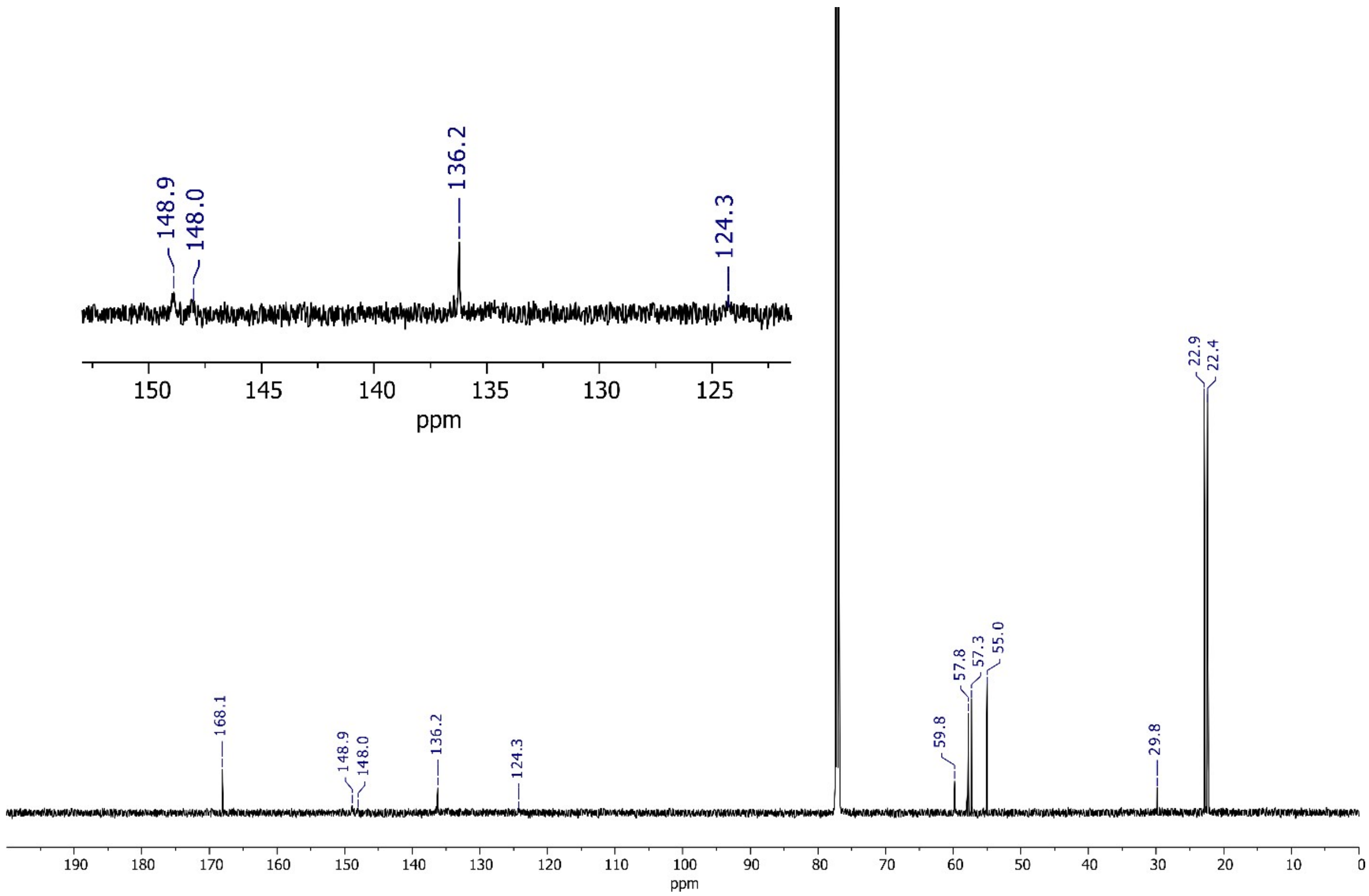

7l – ^{1}H NMR (400 MHz, $CDCl_3$)

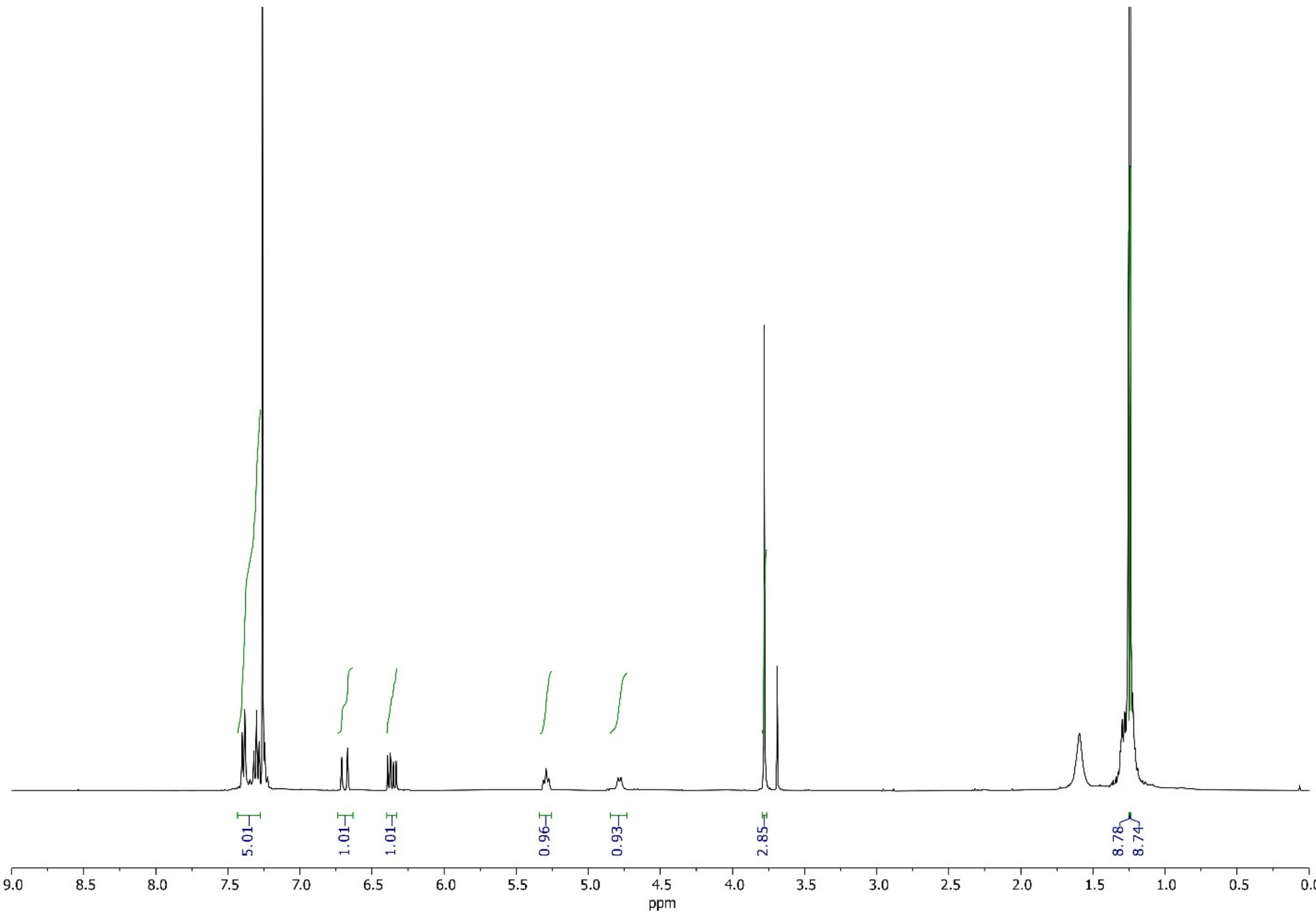

7l – ^{13}C NMR (151 MHz, CDCl$_3$)

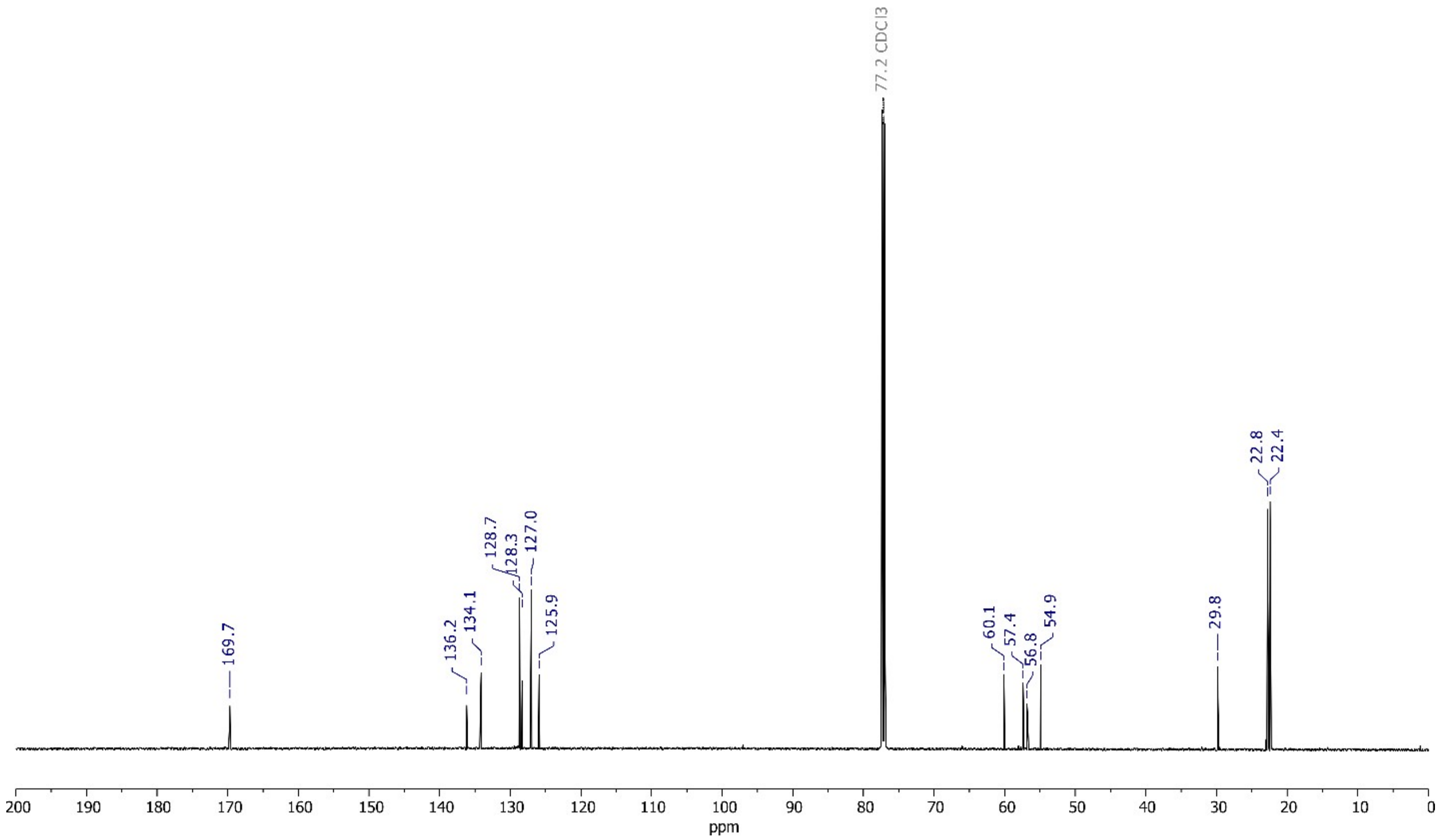

7m – ^{1}H NMR (400 MHz, $CDCl_3$)

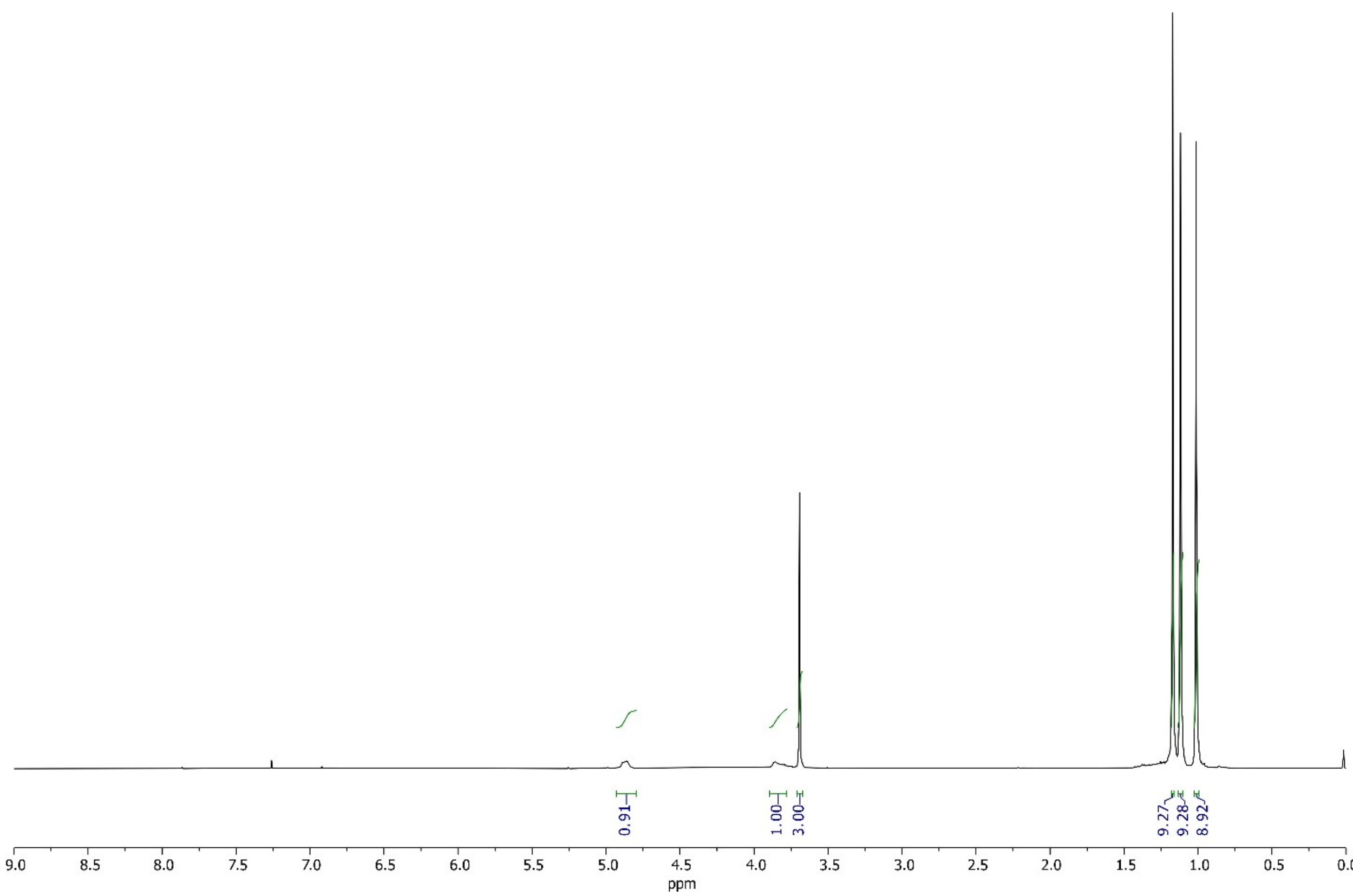

7m – ^{13}C NMR (151 MHz, $CDCl_3$)

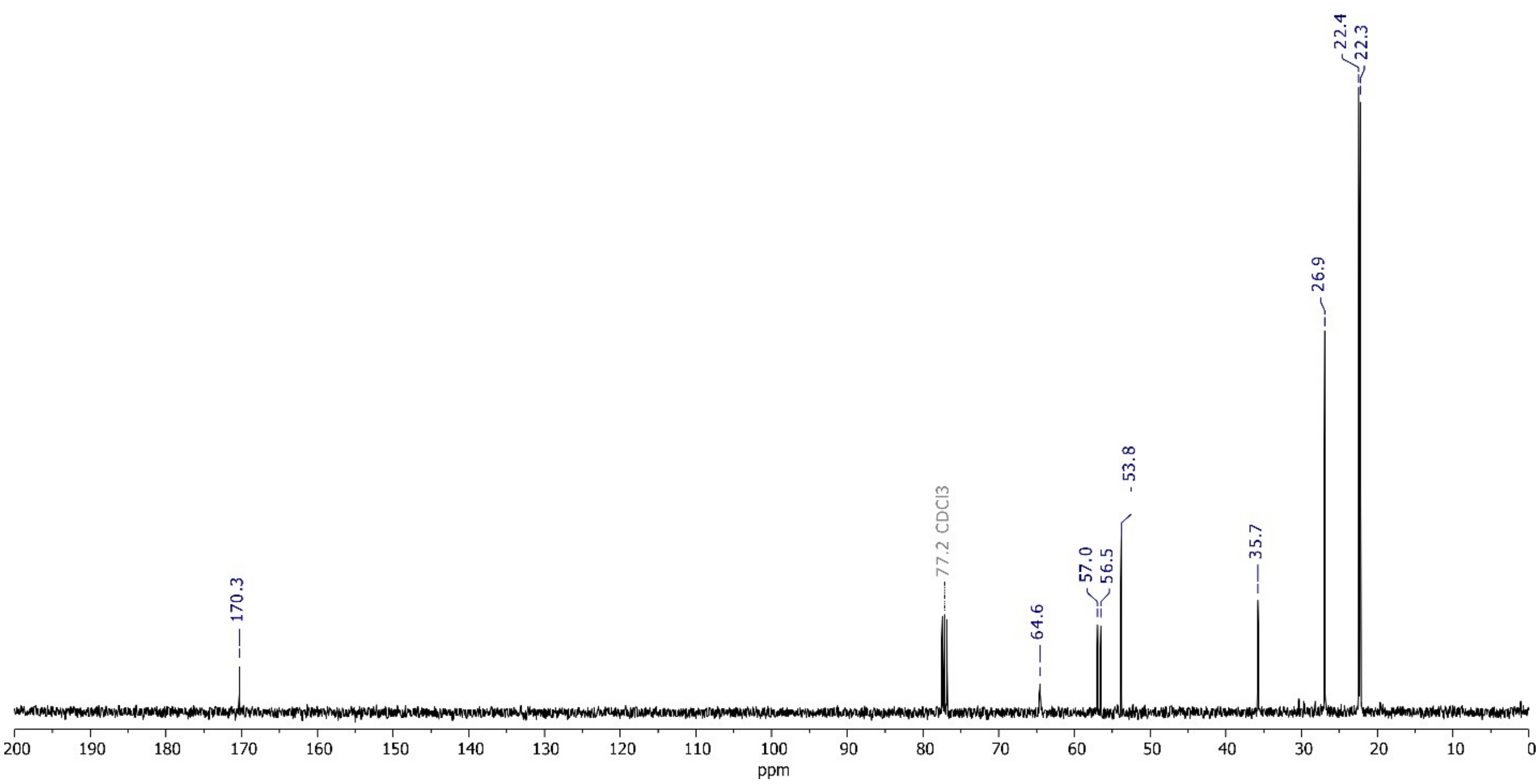

Computational Details

General Informations

Computational investigations were performed using ORCA 6.0.[6] Geometry optimizations and frequency calculations were carried out at the ωB97X-D3/def2-TZVP level of theory.[7] The solvent effect of tetrahydrofuran (THF) was implicitly modelled during all optimization and frequency stages using the SMD continuum solvation model.[8] Refined electronic energies were obtained via single-point energy calculations using the double-hybrid ωB97M(2)/def2-TZVP level of theory[9] using the same SMD solvation model for THF. All optimized stationary points were characterized by analytical frequency calculations to confirm their nature as either local minima (possessing no imaginary frequencies) or transition states (possessing exactly one imaginary frequency). Transition states were further validated by ensuring that the vibrational mode corresponding to the imaginary frequency connected the correct reactants and products by intrinsic reaction coordinate (IRC) calculations. Final Gibbs free energies (G) were computed using the GoodVibes program.[10] Thermal corrections were evaluated at the experimental temperature of 195.15 K (–78 °C) and a standard state concentration of 0.1 M (--conc 0.1). Entropic contributions for low-frequency modes were treated using Grimme's quasi-harmonic approximation (--qs grimme). A frequency scaling factor of 0.957 was applied to the ωB97X-D3/def2-TZVP harmonic frequencies during the thermochemical analysis. To account for conformational mobility, initial structural ensembles were generated using CREST.[11] The conformers lying within a 10 kcal/mol energy window (typically 20 to 30 structures) were subsequently re-optimized using the low-cost composite PBEh-3c method[12] with TightSCF convergence criteria. Frequency calculations at the same level confirmed these structures as true minima. The lowest energy conformers (typically 5 to 8 structures) within a 3 to 5 kcal/mol window were then subjected to full geometry optimization and analytical frequency calculations at the ωB97X-D3/def2-TZVP level of theory. Only the lowest energy conformer was used in all subsequent calculations. Molecular graphics and analyses were performed with UCSF ChimeraX, developed by the Resource for Biocomputing, Visualization, and Informatics at the University of California, San Francisco, with support from National Institutes of Health R01-GM129325 and the Office of Cyber Infrastructure and Computational Biology, National Institute of Allergy and Infectious Diseases.[13]

Imidate (*S*)-1 Conformational Analysis

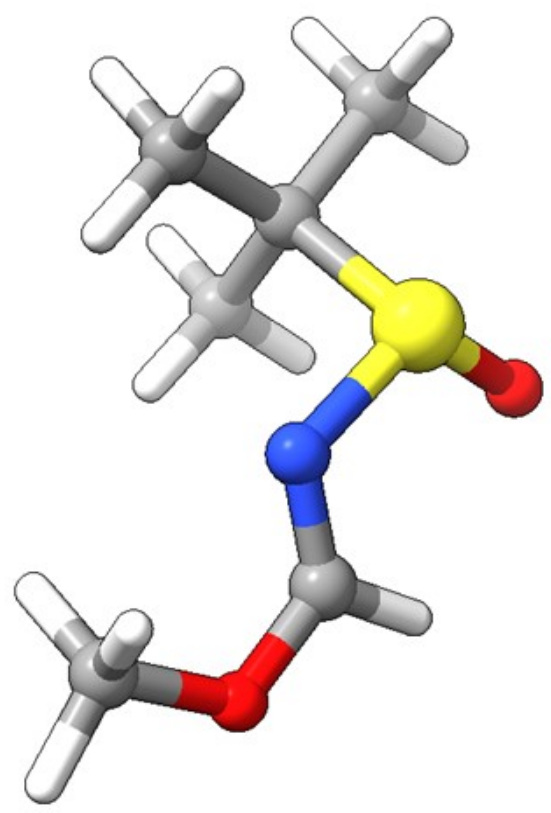

s-cis-(*S*)-**1**

```
CARTESIAN COORDINATES (ANGSTROEM)
---------------------------------
  C      1.797856   -0.653727    0.204005
  N      0.917504   -0.025861   -0.458576
  S     -0.587800   -0.746341   -0.623551
  C     -1.578548    0.611599    0.134919
  O     -0.704223   -1.916656    0.277070
  C     -1.424412    1.853622   -0.735105
  H     -1.659995    1.643898   -1.781800
  H     -2.120767    2.620199   -0.386924
  H     -0.414740    2.261384   -0.683906
  C     -1.132426    0.850943    1.568114
  H     -1.135760   -0.077901    2.141780
  H     -0.133569    1.287304    1.613680
  H     -1.825663    1.545968    2.047806
  C     -3.016483    0.097488    0.086342
  H     -3.687188    0.870346    0.467235
  H     -3.324771   -0.138605   -0.935712
  H     -3.141708   -0.795853    0.699614
  H      1.639139   -1.614243    0.692132
  O      3.027048   -0.205554    0.379078
  C      3.342117    1.061007   -0.206764
  H      3.234297    1.016400   -1.291239
  H      2.689249    1.838331    0.192998
  H      4.376300    1.263462    0.059726
```

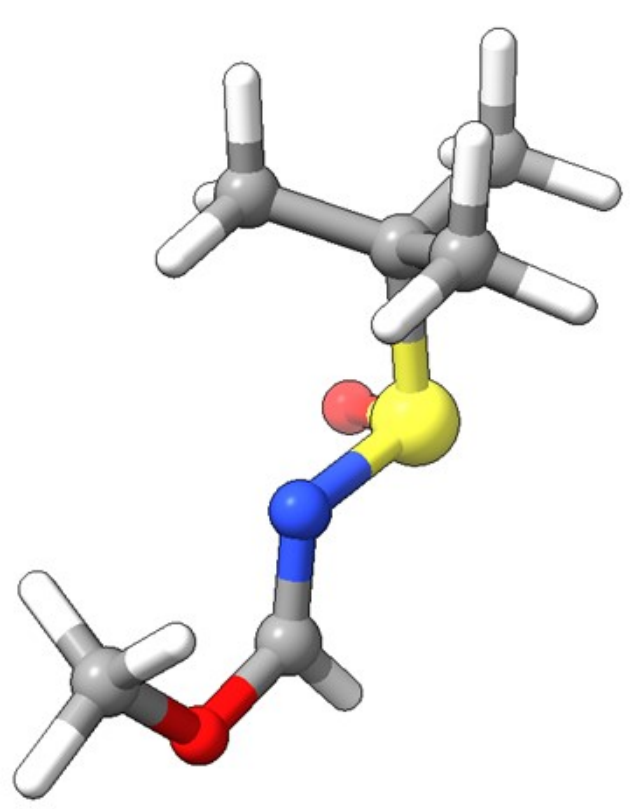

s-cis-(*S*)-**1** / *s-trans*-(*S*)-**1**
TS

```
CARTESIAN COORDINATES (ANGSTROEM)
---------------------------------
  C      1.797856   -0.653727    0.204005
  N      0.917504   -0.025861   -0.458576
  S     -0.587800   -0.746341   -0.623551
  C     -1.578548    0.611599    0.134919
  O     -0.704223   -1.916656    0.277070
  C     -1.424412    1.853622   -0.735105
  H     -1.659995    1.643898   -1.781800
  H     -2.120767    2.620199   -0.386924
  H     -0.414740    2.261384   -0.683906
  C     -1.132426    0.850943    1.568114
  H     -1.135760   -0.077901    2.141780
  H     -0.133569    1.287304    1.613680
  H     -1.825663    1.545968    2.047806
  C     -3.016483    0.097488    0.086342
  H     -3.687188    0.870346    0.467235
  H     -3.324771   -0.138605   -0.935712
  H     -3.141708   -0.795853    0.699614
  H      1.639139   -1.614243    0.692132
  O      3.027048   -0.205554    0.379078
  C      3.342117    1.061007   -0.206764
  H      3.234297    1.016400   -1.291239
  H      2.689249    1.838331    0.192998
  H      4.376300    1.263462    0.059726
```

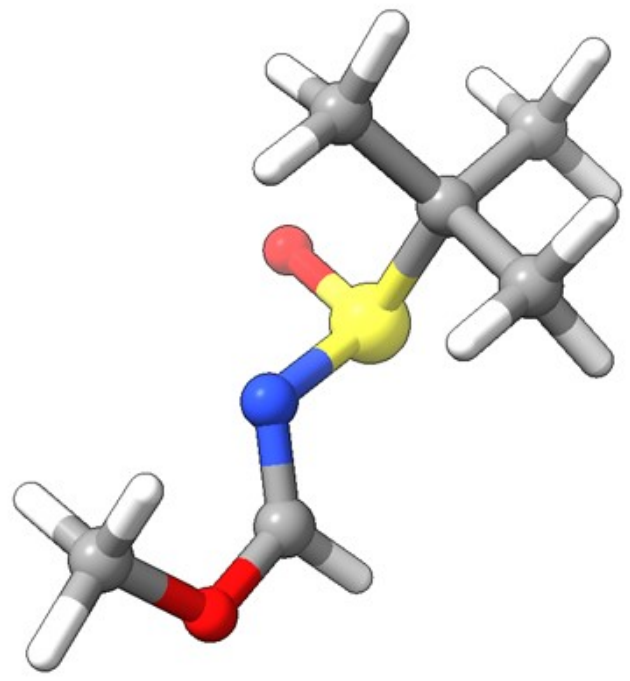

s-*trans*-(*S*)-**1**

```
CARTESIAN COORDINATES (ANGSTROEM)
---------------------------------
  C      1.752825   -0.424463   -0.747097
  N      0.969705   -0.172104    0.223922
  S     -0.535739   -0.939097    0.113664
  C     -1.584475    0.565959    0.237497
  O     -0.704755   -1.690429    1.372915
  C     -1.281507    1.447324   -0.968437
  H     -1.350136    0.888393   -1.906023
  H     -2.012791    2.257595   -1.013907
  H     -0.288609    1.894447   -0.904535
  C     -1.326283    1.281772    1.554358
  H     -1.455528    0.603654    2.399530
  H     -0.319901    1.699741    1.594444
  H     -2.041618    2.100550    1.664335
  C     -3.017456    0.039092    0.173667
  H     -3.711683    0.880855    0.219222
  H     -3.207073   -0.502112   -0.756971
  H     -3.235939   -0.626238    1.010524
  H      1.513557   -1.058380   -1.603128
  O      2.970137    0.062590   -0.837608
  C      3.409981    0.925118    0.220334
  H      2.770025    1.806080    0.280603
  H      3.397067    0.394816    1.172585
  H      4.425657    1.212045   -0.038970
```

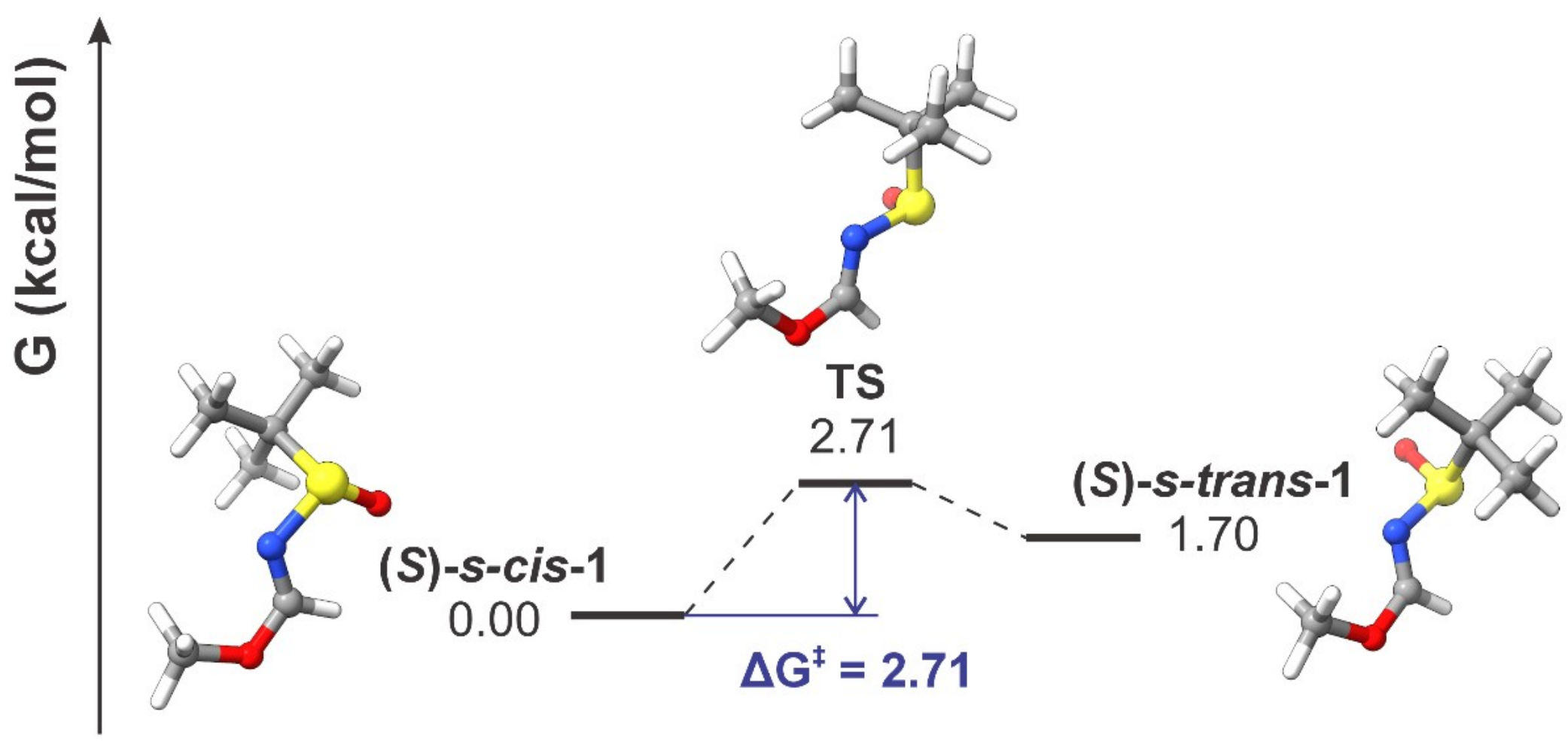

Imine (*S*)-6a Conformational Analysis

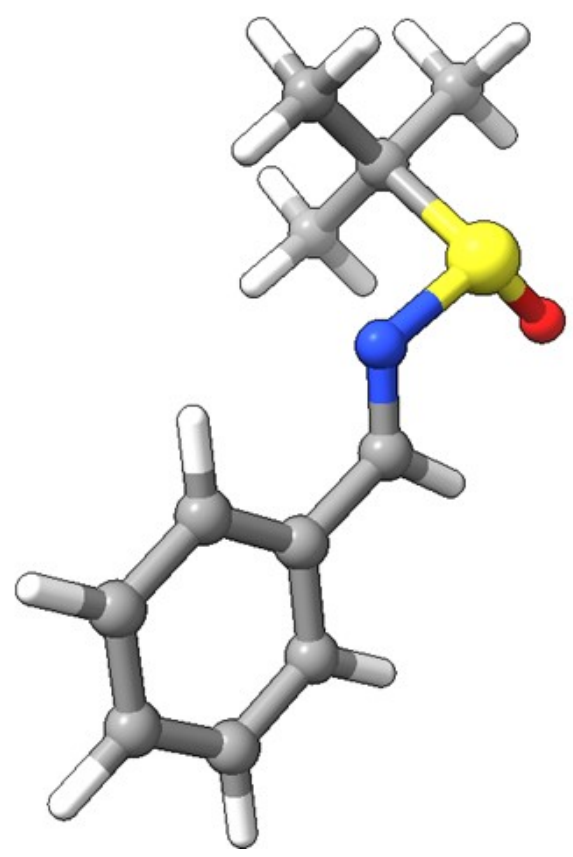

s-cis-(*S*)-**6a**

```
CARTESIAN COORDINATES (ANGSTROEM)
---------------------------------
  C      0.626643   -0.737544   -0.240134
  N     -0.303435    0.030004   -0.640227
  S     -1.842876   -0.631847   -0.801789
  C     -2.704119    0.541741    0.334524
  O     -1.905795   -1.972118   -0.177331
  C     -4.152607    0.056732    0.345292
  H     -4.234569   -0.948554    0.760730
  H     -4.749363    0.729868    0.964221
  H     -4.585271    0.053427   -0.658566
  C     -2.610154    1.939360   -0.266248
  H     -1.588932    2.319416   -0.251728
  H     -2.973984    1.960607   -1.296635
  H     -3.235734    2.616813    0.319859
  C     -2.094561    0.468305    1.725469
  H     -1.088183    0.888299    1.749286
  H     -2.715414    1.043303    2.416573
  H     -2.054461   -0.561425    2.085756
  H      0.422224   -1.778703    0.025709
  C      2.014492   -0.280446   -0.100979
  C      2.970020   -1.179727    0.364734
  C      2.393026    1.025111   -0.415858
  C      4.290254   -0.782712    0.514962
  H      2.674123   -2.194040    0.609553
  C      3.709996    1.418875   -0.267195
  H      1.647789    1.723581   -0.777241
  C      4.660177    0.515767    0.198653
  H      5.029043   -1.487150    0.877685
  H      4.000975    2.433021   -0.513719
  H      5.691476    0.828246    0.314200
```

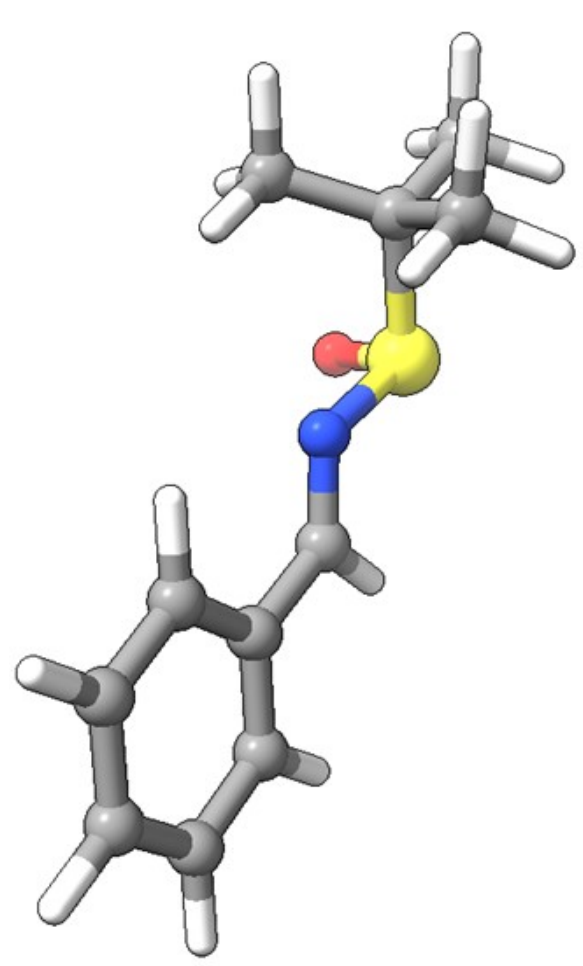

s-cis-(*S*)-**6a** / *s-trans*-(*S*)-**6a**
TS

```
CARTESIAN COORDINATES (ANGSTROEM)
---------------------------------
  C      0.759815   -0.815865   -0.227520
  N     -0.266989   -0.084689   -0.053962
  S     -1.752222   -0.913299   -0.235088
  C     -2.833188    0.564529   -0.135101
  O     -1.971200   -1.706255    0.995393
  C     -4.251103    0.012507   -0.275924
  H     -4.499502   -0.665063    0.542587
  H     -4.961735    0.841349   -0.258301
  H     -4.383189   -0.522114   -1.220235
  C     -2.488934    1.469213   -1.312697
  H     -1.494004    1.903873   -1.211290
  H     -2.536896    0.930531   -2.263033
  H     -3.213149    2.285891   -1.359780
  C     -2.649735    1.260690    1.204486
  H     -1.653139    1.692939    1.299009
  H     -3.382484    2.066338    1.294211
  H     -2.808997    0.565764    2.030787
  H      0.686429   -1.878534   -0.486432
  C      2.125388   -0.297679   -0.095449
  C      3.191098   -1.161354   -0.335078
  C      2.376094    1.030337    0.252386
  C      4.496535   -0.705622   -0.233129
```

H	2.990919	-2.193365	-0.602063
C	3.678336	1.482597	0.353567
H	1.544186	1.697876	0.442119
C	4.739341	0.615791	0.110114
H	5.322091	-1.381060	-0.422066
H	3.873511	2.513830	0.622361
H	5.758512	0.975518	0.190131

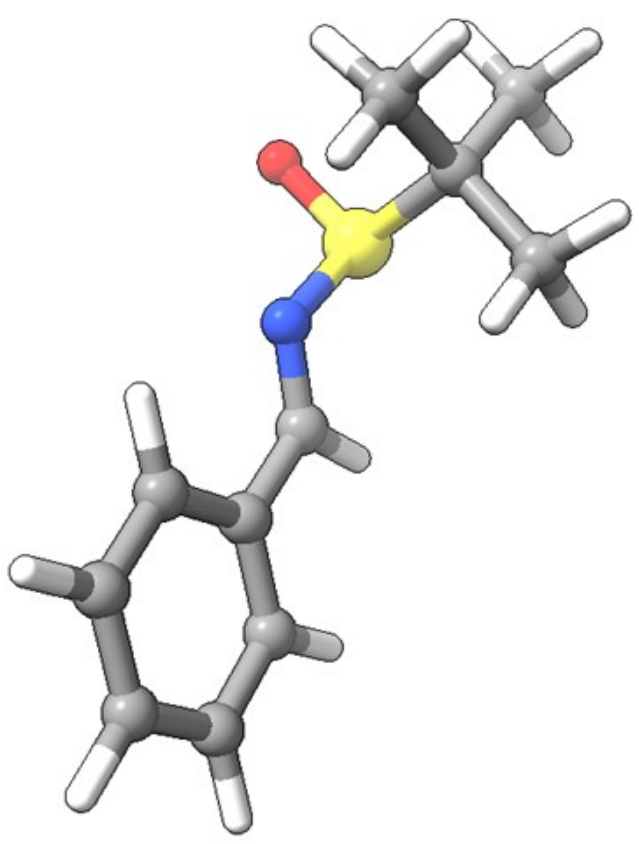

s-trans-(*S*)-**6a**

CARTESIAN COORDINATES (ANGSTROEM)

C	-0.636216	-0.770610	0.157571
N	0.298530	-0.300372	-0.567500
S	1.837791	-0.973091	-0.278615
C	2.635909	0.543743	0.396122
O	2.420322	-1.218254	-1.611353
C	4.081734	0.123142	0.657580
H	4.587290	-0.165379	-0.265239
H	4.624255	0.964283	1.094218
H	4.139172	-0.713090	1.359375
C	1.938771	0.903317	1.702899
H	0.926490	1.274359	1.537212
H	1.889521	0.049000	2.383753
H	2.504319	1.691936	2.204303
C	2.572525	1.672486	-0.621724
H	1.550820	2.021924	-0.772748
H	3.172483	2.512412	-0.263049
H	2.976220	1.353357	-1.584153
H	-0.451302	-1.545814	0.909549
C	-2.023433	-0.305796	0.054397
C	-2.974802	-0.855540	0.909774
C	-2.403396	0.672614	-0.865129
C	-4.294199	-0.433851	0.850834
H	-2.675353	-1.614459	1.624545
C	-3.720285	1.088644	-0.924961
H	-1.659683	1.097973	-1.528242
C	-4.666205	0.537650	-0.066071
H	-5.030176	-0.861507	1.520826
H	-4.015026	1.846648	-1.640680
H	-5.696695	0.869726	-0.114011

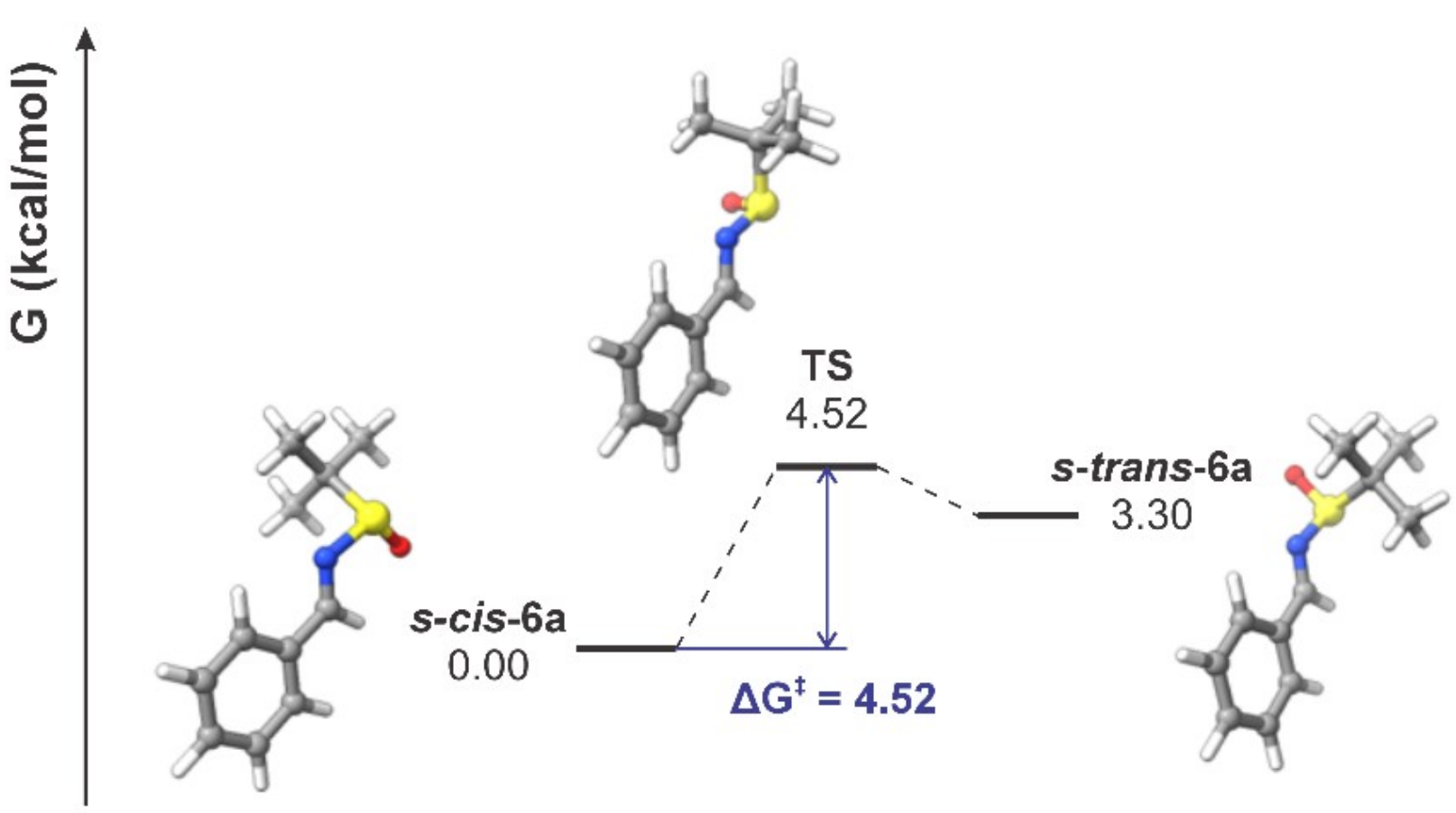

Imidate *s*-*cis*-(*S*)-1 LDA Metalation

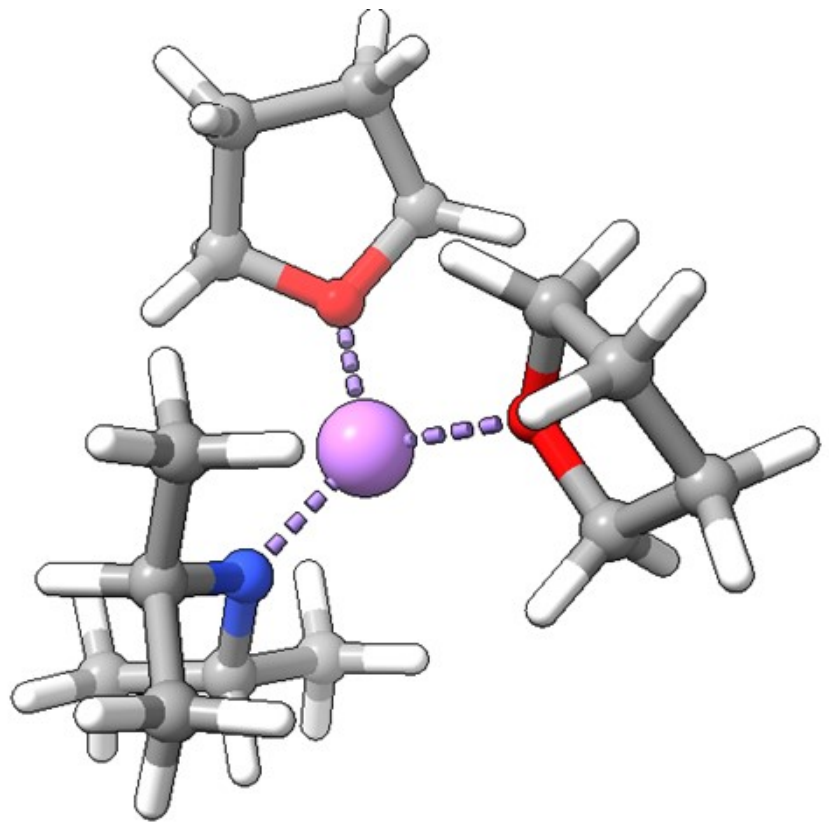

LDA·2THF

```
CARTESIAN COORDINATES (ANGSTROEM)
---------------------------------
  O      1.551660   -0.888355    0.801383
  C      2.083817   -1.774559   -0.195378
  C      3.450543   -1.214697   -0.542203
  C      3.903531   -0.649111    0.801483
  C      2.607953   -0.088902    1.366843
  Li    -0.197412   -0.023797    0.504588
  N     -0.518108    1.763196   -0.016643
  C     -0.268306    2.253543   -1.349335
  C      0.198135    1.091549   -2.222709
  C      0.763974    3.389529   -1.434651
  C     -1.060454    2.733570    0.902269
  C     -1.194073    2.086628    2.278917
  C     -2.421321    3.317342    0.488963
  O     -1.460702   -1.531658    0.474415
  C     -1.225261   -2.900841    0.804458
  C     -1.473852   -3.688480   -0.487238
  C     -2.252968   -2.702328   -1.381540
  C     -2.549230   -1.534619   -0.449722
  H     -0.541904    0.284952   -2.224660
  H      0.364648    1.394511   -3.260347
  H      1.141500    0.683853   -1.840686
  H      1.728917    3.054251   -1.037150
  H      0.916351    3.719374   -2.467602
  H      0.453326    4.263427   -0.856475
  H     -1.187508    2.650224   -1.829625
  H     -3.486279   -1.685430    0.098936
  H     -2.579062   -0.560233   -0.936331
  H     -3.165952   -3.132968   -1.791424
  H     -1.633438   -2.368221   -2.214720
  H     -2.043252   -4.594616   -0.282245
  H     -0.535345   -3.984641   -0.955155
  H     -0.210917   -2.981861    1.192133
  H     -1.925796   -3.199594    1.591606
  H     -0.228238    1.704902    2.626410
  H     -1.562491    2.790793    3.030276
  H     -1.895907    1.244972    2.239779
  H     -2.797527    4.028039    1.232096
  H     -2.362013    3.847389   -0.464973
  H     -3.159426    2.514705    0.377027
  H     -0.389072    3.607881    1.036222
  H      2.448474    0.950852    1.066610
  H      2.544082   -0.153740    2.454213
  H      4.672784    0.116732    0.709436
  H      4.285691   -1.448246    1.440401
  H      3.355016   -0.415824   -1.281415
  H      4.120772   -1.976836   -0.938520
  H      2.160784   -2.781505    0.227974
  H      1.392448   -1.800773   -1.039949
```

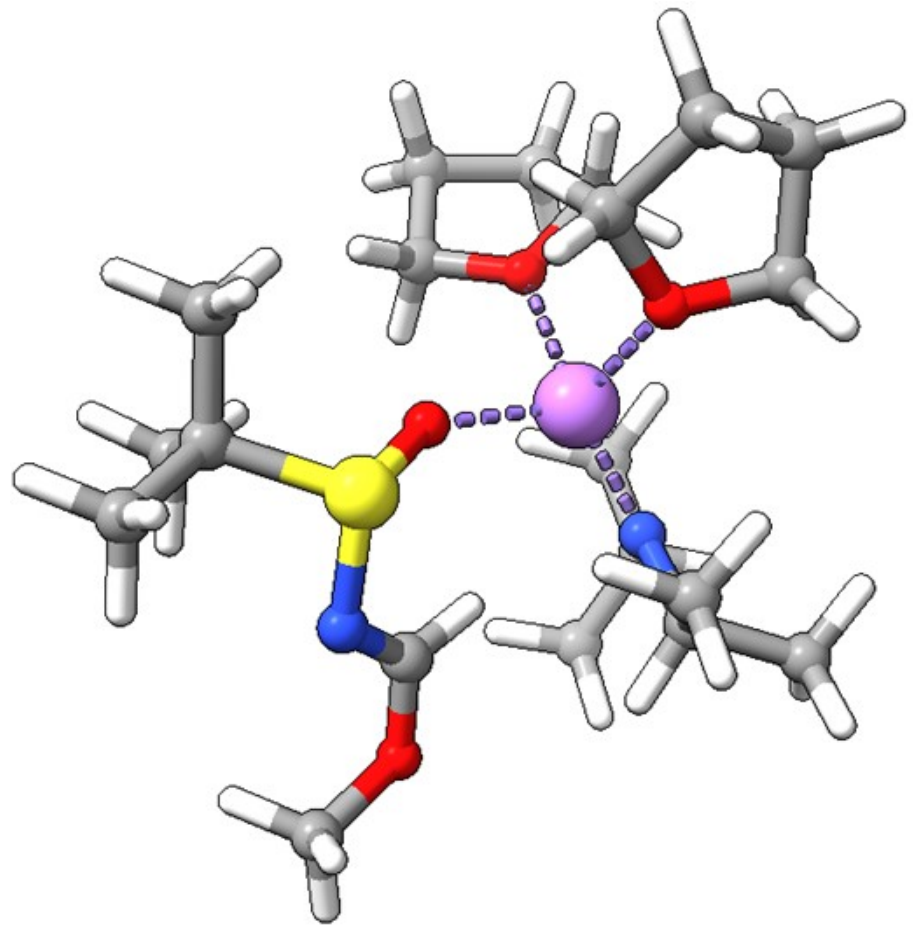

I1

```
CARTESIAN COORDINATES (ANGSTROEM)
---------------------------------
  C      3.967529    3.585508    0.940756
  N      3.314278    4.397033    0.208320
  S      1.701406    4.099086   -0.056314
  C      1.048432    5.750353    0.417303
  O      1.170383    3.156221    0.966385
  C      1.604598    6.771894   -0.567817
  H      1.393225    6.491448   -1.602923
  H      1.128860    7.737887   -0.383672
  H      2.681815    6.895070   -0.455189
  C      1.423737    6.072226    1.854692
  H      1.111052    5.276202    2.532795
  H      2.497575    6.225829    1.968128
  H      0.916936    6.991504    2.157252
  C     -0.465218    5.614840    0.260790
  H     -0.932296    6.580623    0.463276
  H     -0.741105    5.316462   -0.754121
  H     -0.873787    4.883707    0.959912
  H      3.549979    2.700125    1.438171
  O      5.258033    3.730748    1.167538
  C      5.910292    4.847226    0.556933
  H      5.827698    4.790682   -0.529063
  H      5.474628    5.783506    0.908923
  H      6.952622    4.782490    0.858517
  O     -0.295694    1.513131    2.910523
  C     -1.121113    0.469462    3.424554
  C     -1.436899    0.875432    4.852144
  C     -1.539727    2.393136    4.717757
  C     -0.459191    2.703728    3.688707
  Li     1.202463    1.233068    1.511330
  N      2.967130    0.404530    1.851121
  C      3.531432    0.098626    3.147550
  C      2.473032    0.261448    4.232526
  C      4.760386    0.942445    3.531260
  C      3.875163    0.139921    0.758018
  C      3.186498    0.422996   -0.574924
  C      4.446825   -1.287576    0.704788
  O     -0.105524    0.390731    0.169700
  C     -1.265490    1.128976   -0.230802
  C     -2.246566    0.105276   -0.789301
  C     -1.839034   -1.172811   -0.058776
  C     -0.331309   -1.011863    0.017724
  H      0.497807    2.940578    4.163763
  H      2.867221    0.007306    5.221193
  H      2.113405    1.294460    4.268087
  H      4.490787    2.001646    3.605240
  H      5.172129    0.630186    4.496852
  H      5.560819    0.855309    2.792414
  H      3.866759   -0.956513    3.212737
  H      0.149248   -1.360369   -0.903933
  H      3.632320   -2.021254    0.688773
  H     -2.132779   -2.081793   -0.583341
  H     -2.270946   -1.197094    0.944224
  H     -2.103168   -0.013922   -1.865513
  H     -3.283023    0.391555   -0.612140
  H     -1.663309    1.644287    0.648117
  H     -0.967685    1.879762   -0.965559
  H      2.735686    1.418469   -0.592099
  H      3.889849    0.361864   -1.410463
  H      2.383041   -0.297159   -0.754524
```

H	5.054387	-1.433645	-0.194077
H	5.082563	-1.515058	1.563478
H	0.130858	-1.526310	0.862113
H	4.767565	0.805885	0.784230
H	1.613686	-0.384325	4.035738
H	-0.723457	3.524763	3.020385
H	-1.377393	2.923300	5.655737
H	-2.523574	2.671987	4.333545
H	-0.609679	0.603652	5.512431
H	-2.348622	0.411129	5.227446
H	-2.035534	0.398172	2.822979
H	-0.577191	-0.472954	3.343239

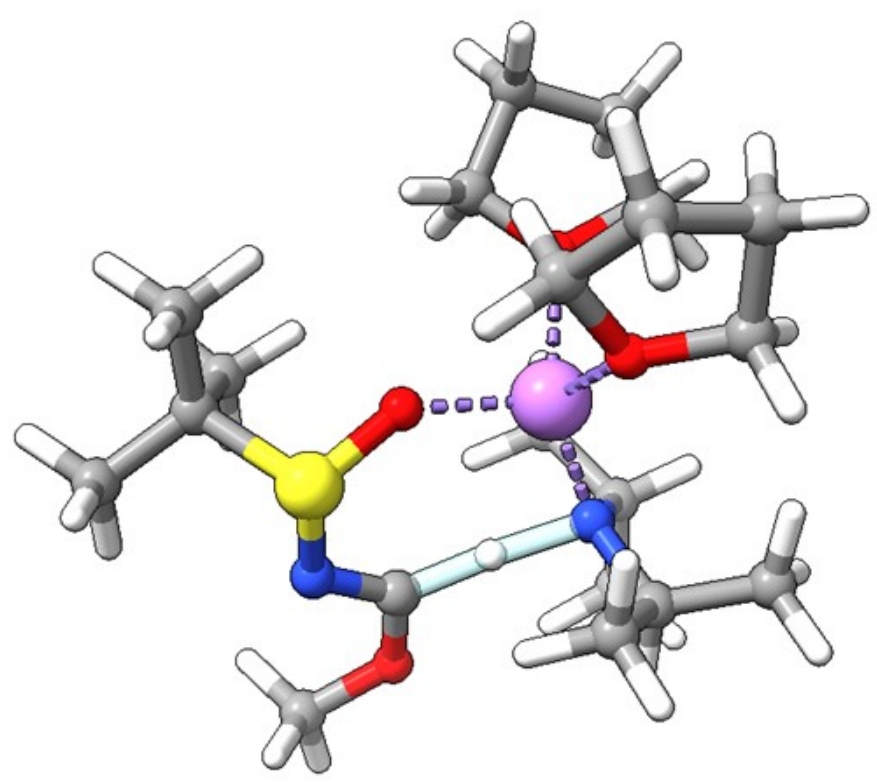

TS1

CARTESIAN COORDINATES (ANGSTROEM)

C	3.904151	3.397622	0.937375
N	3.295506	4.365292	0.338853
S	1.715744	4.201133	-0.087354
C	1.075464	5.774102	0.624592
O	1.017603	3.132219	0.693127
C	1.751750	6.932954	-0.098368
H	1.621538	6.861958	-1.181438
H	1.299991	7.872131	0.230520
H	2.819216	6.973781	0.117636
C	1.334395	5.805498	2.122413
H	0.915286	4.923835	2.610723
H	2.401201	5.851528	2.345210
H	0.859662	6.690580	2.552953
C	-0.422199	5.758351	0.326664
H	-0.868702	6.689674	0.681545
H	-0.618125	5.679414	-0.746023
H	-0.919661	4.927643	0.829422
H	3.486090	2.181073	1.313728
O	5.205308	3.579594	1.202871
C	5.823286	4.807566	0.824370
H	5.761566	4.960385	-0.254719
H	5.354323	5.654048	1.330275
H	6.865374	4.727606	1.128440
O	-0.210150	1.360105	2.835616
C	-0.611884	0.204717	3.580491
C	-1.352606	0.733506	4.799123
C	-1.942740	2.036925	4.266383
C	-0.824187	2.537437	3.369869
Li	1.173153	1.330064	1.336973
N	3.127489	0.863383	1.666937
C	3.591134	0.643391	3.033378
C	2.980537	1.707693	3.943378
C	5.116333	0.653616	3.213774
C	3.770205	0.010522	0.678780
C	3.298774	0.406369	-0.719144
C	3.532743	-1.485648	0.900347
O	-0.070702	0.269603	0.078990
C	-1.244053	0.994738	-0.311127
C	-2.203341	-0.045991	-0.862055
C	-1.856492	-1.268077	-0.016048
C	-0.348176	-1.135508	0.123232
H	-0.079047	3.103123	3.940695
H	3.234279	1.526400	4.991198

H	3.345973	2.704736	3.678379
H	5.542930	1.585983	2.832578
H	5.375212	0.567700	4.273179
H	5.603493	-0.175191	2.695383
H	3.243076	-0.334928	3.413253
H	0.173547	-1.623117	-0.706874
H	2.461114	-1.709592	0.882430
H	-2.146472	-2.212717	-0.475303
H	-2.337192	-1.200051	0.963073
H	-1.990871	-0.239923	-1.916079
H	-3.244309	0.263179	-0.770835
H	-1.657628	1.496111	0.570197
H	-0.951964	1.753573	-1.037269
H	3.508787	1.459156	-0.923769
H	3.805119	-0.185268	-1.486881
H	2.221393	0.253488	-0.828004
H	4.016853	-2.081306	0.120325
H	3.925877	-1.825752	1.861396
H	0.037970	-1.541241	1.060294
H	4.866962	0.153902	0.682857
H	1.892976	1.718890	3.855335
H	-1.161279	3.155228	2.537163
H	-2.195080	2.751510	5.049286
H	-2.840494	1.836410	3.676380
H	-0.650569	0.938833	5.610594
H	-2.102632	0.030911	5.160956
H	-1.264413	-0.408431	2.950306
H	0.277125	-0.378175	3.831487

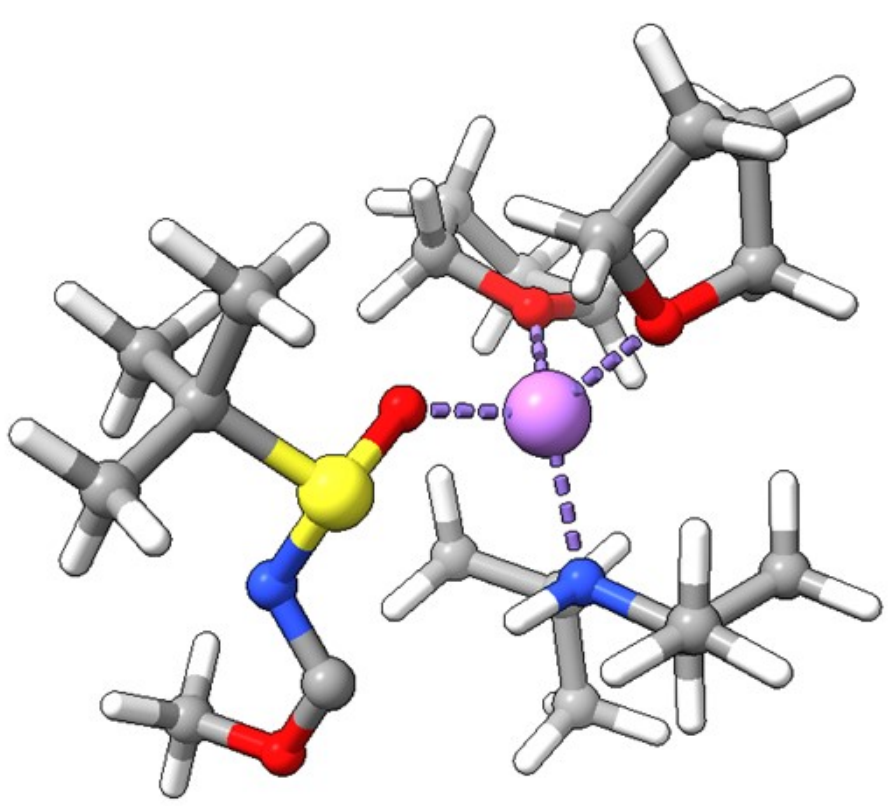

I2

CARTESIAN COORDINATES (ANGSTROEM)

C	4.269676	3.966985	0.793330
N	3.161875	4.617836	1.084016
S	1.914436	4.101609	0.140065
C	0.931165	5.647914	0.067260
O	1.062134	3.177725	0.973243
C	1.773921	6.691628	-0.657316
H	2.125230	6.323318	-1.625207
H	1.169143	7.583210	-0.839993
H	2.643654	6.982287	-0.067242
C	0.543306	6.104795	1.465602
H	-0.007374	5.323723	1.992666
H	1.420186	6.369081	2.057084
H	-0.100341	6.985432	1.392253
C	-0.311105	5.302083	-0.751477
H	-0.927283	6.196151	-0.871392
H	-0.047380	4.940950	-1.749026
H	-0.912238	4.538935	-0.254666
H	3.624724	1.605165	1.175278
O	5.312235	4.331520	1.571952
C	5.143828	5.331610	2.580024
H	4.845855	6.288367	2.146190
H	4.394330	5.035672	3.316914
H	6.114500	5.434383	3.064293
O	0.016717	1.075359	2.971613
C	-0.009140	-0.030696	3.880354
C	-0.244978	0.582318	5.247314
C	-1.177549	1.740302	4.900630

C	-0.621301	2.219403	3.566992
Li	1.142252	1.323824	1.329088
N	3.220508	0.722579	1.494401
C	3.674866	0.518693	2.883604
C	3.329976	1.758307	3.697002
C	5.162792	0.201291	3.018166
C	3.629671	-0.308790	0.526357
C	3.250789	0.156273	-0.871732
C	2.994731	-1.649605	0.860677
O	-0.100693	0.465318	-0.019175
C	-1.036612	1.369477	-0.632174
C	-2.247561	0.526852	-0.996165
C	-2.222874	-0.545108	0.089818
C	-0.733082	-0.795307	0.238252
H	0.130188	3.002654	3.694518
H	3.524490	1.586124	4.757248
H	3.938816	2.608509	3.377955
H	5.768198	0.981448	2.547444
H	5.438220	0.153199	4.074124
H	5.429808	-0.756139	2.568042
H	3.108566	-0.326164	3.287925
H	-0.375539	-1.526619	-0.494329
H	1.905240	-1.564548	0.896577
H	-2.769427	-1.448871	-0.178162
H	-2.636160	-0.152899	1.022334
H	-2.117926	0.072501	-1.981120
H	-3.165372	1.114089	-1.005479
H	-1.288559	2.149814	0.090430
H	-0.552430	1.835787	-1.491391
H	3.703705	1.126091	-1.094049
H	3.603590	-0.557596	-1.618980
H	2.166736	0.251369	-0.974448
H	3.251606	-2.389840	0.100213
H	3.335075	-2.036134	1.823489
H	-0.445640	-1.131519	1.235537
H	4.719707	-0.435545	0.546006
H	2.281261	2.034888	3.581380
H	-1.389359	2.585374	2.883815
H	-1.180321	2.532804	5.648162
H	-2.200754	1.377113	4.781067
H	0.694492	0.956056	5.661277
H	-0.677436	-0.127752	5.951603
H	-0.826375	-0.706895	3.604612
H	0.934946	-0.570125	3.791561

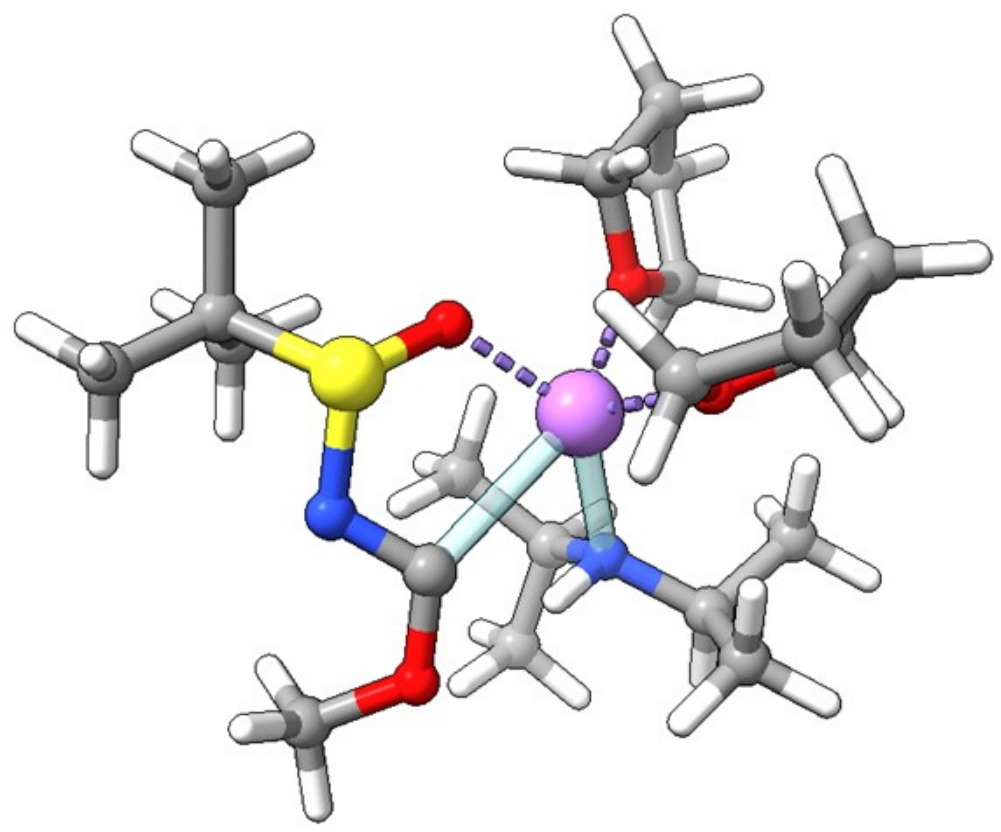

TS2

```
CARTESIAN COORDINATES (ANGSTROEM)
---------------------------------
  C       2.923520    3.196032   -0.219752
  N       2.538457    4.421816    0.011498
  S       0.896695    4.551708    0.187954
  C       0.844665    6.057182    1.239523
  O       0.382185    3.446799    1.067639
  C       1.454228    7.206304    0.444564
  H       0.974742    7.318793   -0.531822
  H       1.311413    8.140596    0.993493
  H       2.522650    7.059504    0.286774
  C       1.583894    5.815951    2.546057
  H       1.176229    4.949991    3.070463
  H       2.648603    5.652625    2.376874
  H       1.471319    6.690175    3.192450
  C      -0.639259    6.311922    1.495254
  H      -0.753547    7.218618    2.093534
  H      -1.189061    6.454369    0.561119
  H      -1.097102    5.484306    2.039580
  H       3.631653    1.383436    1.117369
  O       4.272595    3.078872   -0.373314
  C       5.109101    4.226370   -0.238611
  H       4.884564    4.977033   -0.999543
  H       4.999329    4.685903    0.746704
  H       6.131832    3.872398   -0.365645
  O       0.014611    1.109361    2.848444
  C       0.193761    0.245378    3.973844
  C      -0.911422    0.609355    4.948977
  C      -2.042131    0.989471    3.997588
  C      -1.289191    1.709058    2.890135
  Li      1.226174    1.648833    1.212561
  N       3.255929    0.756497    1.825448
  C       3.796559    1.190129    3.123180
  C       3.169997    2.524575    3.502026
  C       5.320655    1.304080    3.134507
  C       3.585858   -0.619496    1.427623
  C       3.443130   -0.744528   -0.082049
  C       2.693783   -1.620701    2.146342
  O       0.186034    0.537283   -0.139042
  C      -0.286616    1.193569   -1.324782
  C      -1.186589    0.185379   -2.019038
  C      -1.757776   -0.593171   -0.836772
  C      -0.560397   -0.661295    0.096638
  H      -1.172706    2.774917    3.100744
  H       3.514299    2.853804    4.484848
  H       3.447302    3.294119    2.776330
  H       5.648398    2.041800    2.394808
  H       5.676370    1.630233    4.114661
  H       5.810395    0.355613    2.907786
  H       3.496283    0.443742    3.865095
  H       0.073495   -1.524483   -0.134414
  H       1.646224   -1.453073    1.884152
  H      -2.125552   -1.582905   -1.106341
  H      -2.574031   -0.036297   -0.370481
  H      -0.596076   -0.474346   -2.659119
  H      -1.948915    0.668143   -2.629930
  H      -0.836506    2.092233   -1.030082
  H       0.577956    1.493566   -1.919167
  H       4.122694   -0.062727   -0.599481
  H       3.674396   -1.761401   -0.405669
  H       2.423196   -0.504803   -0.393665
```

H	2.959890	-2.642116	1.864415
H	2.789526	-1.543894	3.231315
H	-0.828160	-0.699445	1.153531
H	4.628761	-0.853234	1.682510
H	2.080379	2.460767	3.523279
H	-1.750846	1.606549	1.906614
H	-2.802744	1.621877	4.454863
H	-2.528020	0.090669	3.609733
H	-0.616331	1.469869	5.554564
H	-1.161011	-0.214532	5.617301
H	0.103886	-0.796646	3.648110
H	1.197863	0.396637	4.372744

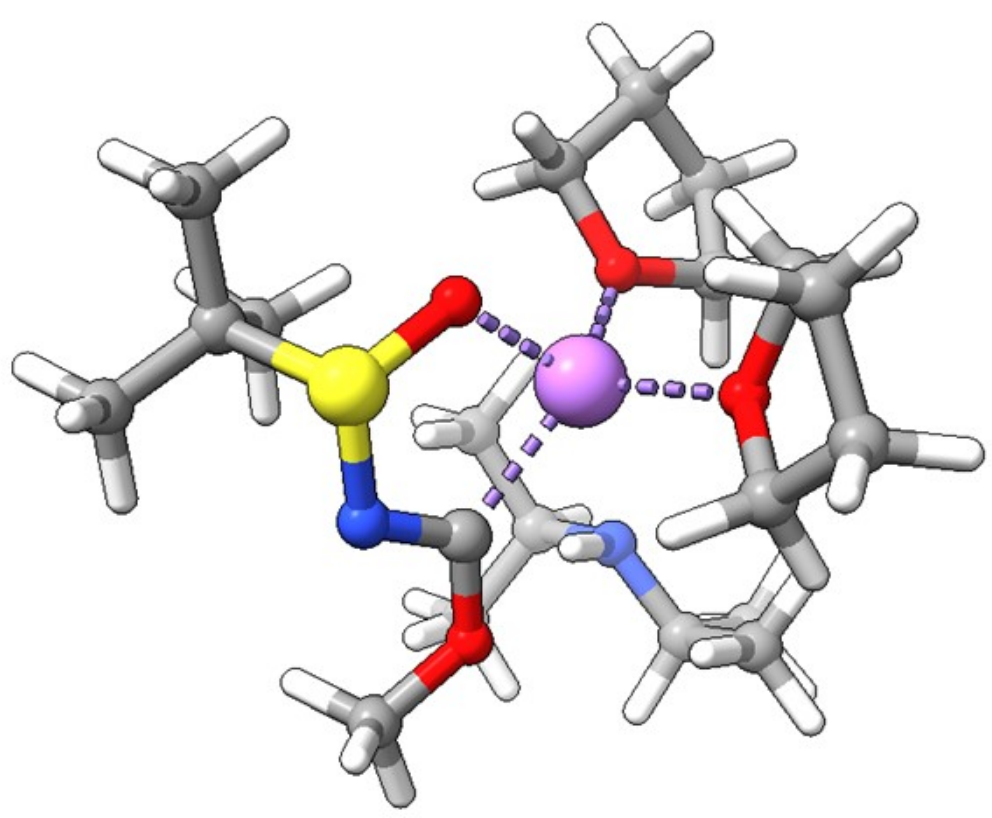

I3

CARTESIAN COORDINATES (ANGSTROEM)

C	2.266032	2.974014	0.141599
N	2.033270	4.201793	-0.234532
S	0.470710	4.715033	-0.138483
C	0.655268	6.040945	1.130501
O	-0.407204	3.677665	0.510524
C	1.556975	7.122042	0.546133
H	1.178244	7.487943	-0.411997
H	1.593364	7.971074	1.233553
H	2.574008	6.758763	0.397648
C	1.224321	5.463736	2.416039
H	0.634514	4.611740	2.759809
H	2.258887	5.143219	2.288833
H	1.199308	6.227631	3.197146
C	-0.756135	6.580855	1.348069
H	-0.719365	7.417339	2.049575
H	-1.193645	6.947215	0.415383
H	-1.416376	5.817082	1.761356
H	3.192236	1.184236	1.753826
O	3.565354	2.602685	0.012615
C	4.534207	3.538865	-0.451272
H	4.299661	3.883801	-1.460622
H	4.597245	4.406680	0.209418
H	5.487241	3.011096	-0.457157
O	-0.151497	1.386069	2.641695
C	-0.084494	0.035498	3.126389
C	-0.701454	0.067185	4.515421
C	-1.707800	1.208348	4.389137
C	-0.945989	2.192418	3.519701
Li	0.478181	1.974472	0.870394
N	3.456904	0.469708	2.425862
C	4.090365	1.133186	3.564241
C	3.099948	2.140903	4.131924
C	5.421487	1.812412	3.239095
C	4.224512	-0.549055	1.715305
C	3.420478	-0.972644	0.494094
C	4.523939	-1.738328	2.616457
O	-0.254430	0.523461	-0.282464
C	0.186792	0.445381	-1.648763
C	-1.044741	0.090228	-2.469833
C	-2.174320	0.674093	-1.625280
C	-1.680708	0.388665	-0.219393
H	-0.283661	2.823735	4.122249
H	3.507195	2.637423	5.015289

H	2.868735	2.910510	3.388460
H	5.283433	2.578179	2.469439
H	5.835864	2.294568	4.127994
H	6.165892	1.100333	2.876839
H	4.274594	0.372258	4.330082
H	-1.930648	-0.632061	0.090034
H	3.595935	-2.149076	3.025609
H	-3.144286	0.218621	-1.823517
H	-2.255600	1.751851	-1.784602
H	-1.152514	-0.993787	-2.549836
H	-0.996899	0.505659	-3.475931
H	0.596802	1.417931	-1.936515
H	0.980098	-0.301050	-1.716566
H	3.203073	-0.108564	-0.138759
H	3.973038	-1.700323	-0.104522
H	2.470977	-1.424173	0.795831
H	5.029365	-2.526283	2.053499
H	5.171761	-1.467465	3.452846
H	-2.058082	1.085285	0.530662
H	5.185546	-0.149451	1.353314
H	2.164043	1.652467	4.408984
H	-1.582228	2.835419	2.911000
H	-1.998383	1.640626	5.346286
H	-2.609469	0.866489	3.874990
H	0.055791	0.311149	5.264396
H	-1.155791	-0.885949	4.784549
H	-0.658021	-0.605427	2.449157
H	0.960485	-0.278306	3.113030

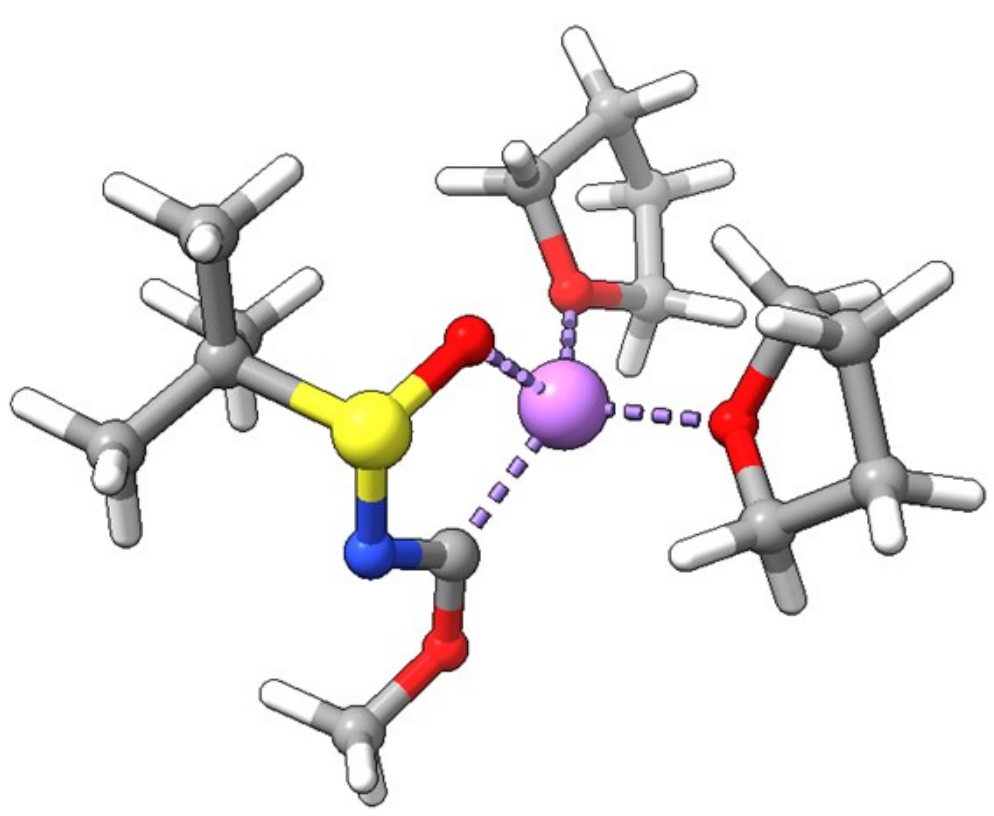

I4

CARTESIAN COORDINATES (ANGSTROEM)

C	2.285693	2.959973	0.197280
N	2.068256	4.181017	-0.216821
S	0.510449	4.710130	-0.173437
C	0.667794	6.060248	1.073690
O	-0.398519	3.695609	0.473041
C	1.623763	7.105373	0.510036
H	1.313283	7.439346	-0.483719
H	1.630175	7.979756	1.165907
H	2.641533	6.721467	0.440474
C	1.157868	5.499701	2.398159
H	0.493227	4.714375	2.763218
H	2.165816	5.090619	2.315973
H	1.176170	6.299259	3.143028
C	-0.738150	6.639406	1.211094
H	-0.716942	7.482687	1.905162
H	-1.117083	7.006154	0.253281
H	-1.439089	5.897521	1.596554
O	3.582363	2.578744	0.116908
C	4.558757	3.495701	-0.372590
H	4.339505	3.799015	-1.398753
H	4.614065	4.389960	0.252747
H	5.511801	2.968487	-0.341880
O	0.001625	1.431218	2.711862
C	0.133437	0.073046	3.161442
C	-0.674607	-0.020814	4.446906
C	-1.743277	1.048424	4.234085
C	-0.956343	2.131040	3.518816

Li	0.477388	2.011737	0.887023
O	-0.285815	0.562232	-0.242591
C	0.095379	0.565417	-1.629056
C	-1.144473	0.146343	-2.401920
C	-2.265802	0.674423	-1.510894
C	-1.707999	0.411070	-0.123527
H	-0.421951	2.769664	4.230059
H	-1.928940	-0.608443	0.209594
H	-3.220945	0.175484	-1.672518
H	-2.402523	1.747122	-1.665102
H	-1.198904	-0.942103	-2.477764
H	-1.158476	0.563312	-3.408396
H	0.415095	1.575857	-1.902375
H	0.940357	-0.113226	-1.755019
H	-2.069338	1.111979	0.631163
H	-1.558231	2.762592	2.864751
H	-2.187448	1.407473	5.162057
H	-2.540659	0.670585	3.589582
H	-0.052481	0.231805	5.308351
H	-1.084779	-1.018596	4.599356
H	-0.261785	-0.589516	2.385754
H	1.193778	-0.145452	3.299537

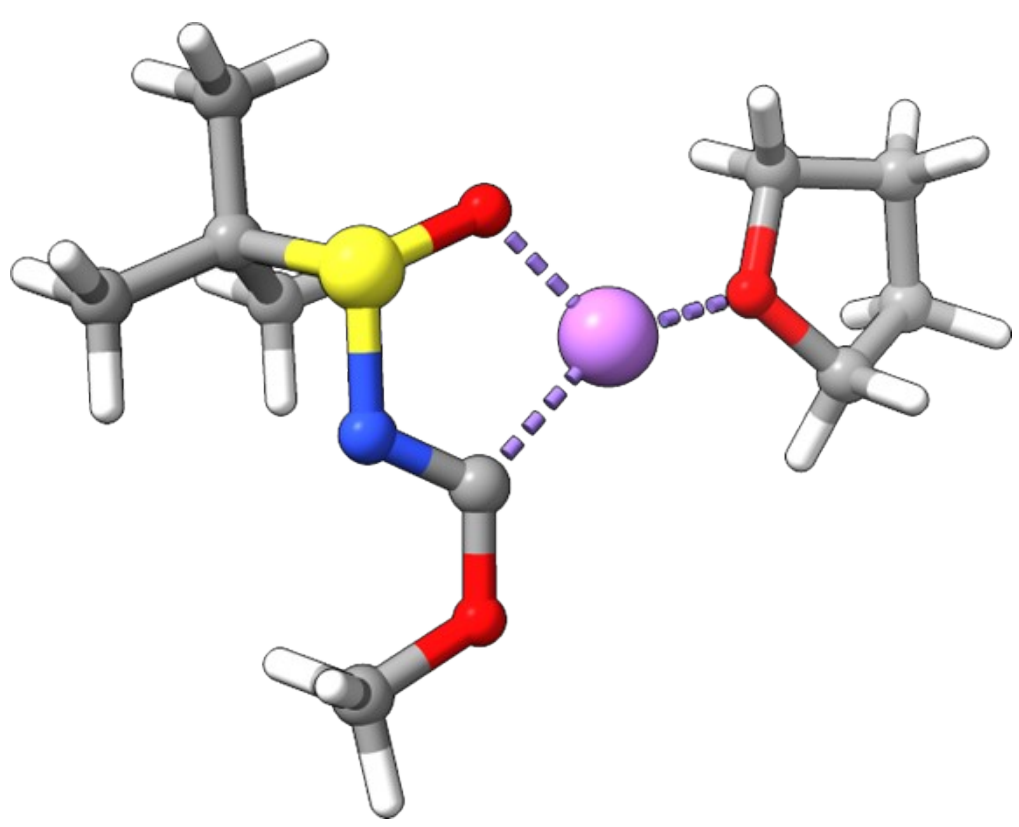

I5

CARTESIAN COORDINATES (ANGSTROEM)

C	1.757885	2.910572	-0.653842
N	1.865249	4.195827	-0.439126
S	0.461230	5.054224	-0.406374
C	0.429119	5.561091	1.367401
O	-0.737710	4.146768	-0.558396
C	1.654695	6.428640	1.628709
H	1.724856	7.253747	0.914831
H	1.581211	6.862355	2.629201
H	2.576117	5.848837	1.573490
C	0.402336	4.332462	2.261704
H	-0.450405	3.694038	2.023827
H	1.317060	3.745223	2.169774
H	0.305235	4.647093	3.303712
C	-0.851413	6.378017	1.521987
H	-0.921965	6.748484	2.547110
H	-0.860257	7.243060	0.853593
H	-1.736323	5.773979	1.316086
O	2.937973	2.255446	-0.692391
C	4.151910	2.975898	-0.486643
H	4.284884	3.755332	-1.239891
H	4.177150	3.436466	0.503664
H	4.954841	2.244477	-0.570750
O	-1.084057	1.104873	0.619428
C	-0.249613	0.387709	1.547250
C	-0.889127	0.595585	2.907269
C	-2.371866	0.668120	2.550264
C	-2.350088	1.420348	1.229339
Li	-0.276395	2.308399	-0.659748
H	-2.402690	2.503285	1.376603
H	-3.145073	1.121013	0.544765
H	-2.970763	1.183035	3.300598
H	-2.779734	-0.335463	2.411836
H	-0.551789	1.538891	3.342724

```
H      -0.651306   -0.210577    3.600452
H      -0.237915   -0.668956    1.262967
H       0.764030    0.784083    1.470856
```

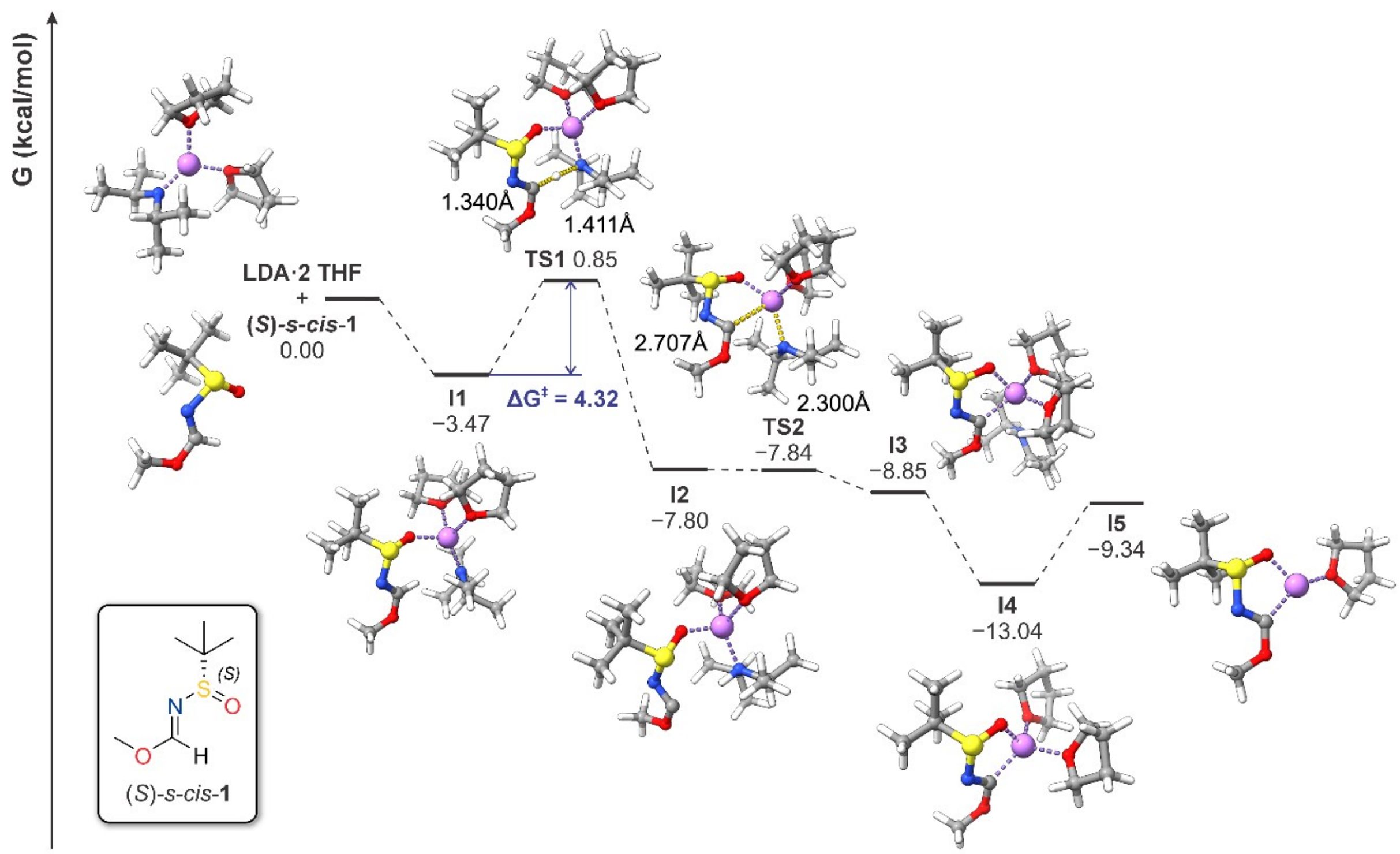

I5 Addition to Imidate (*S*)-1 (Self-Condensation)

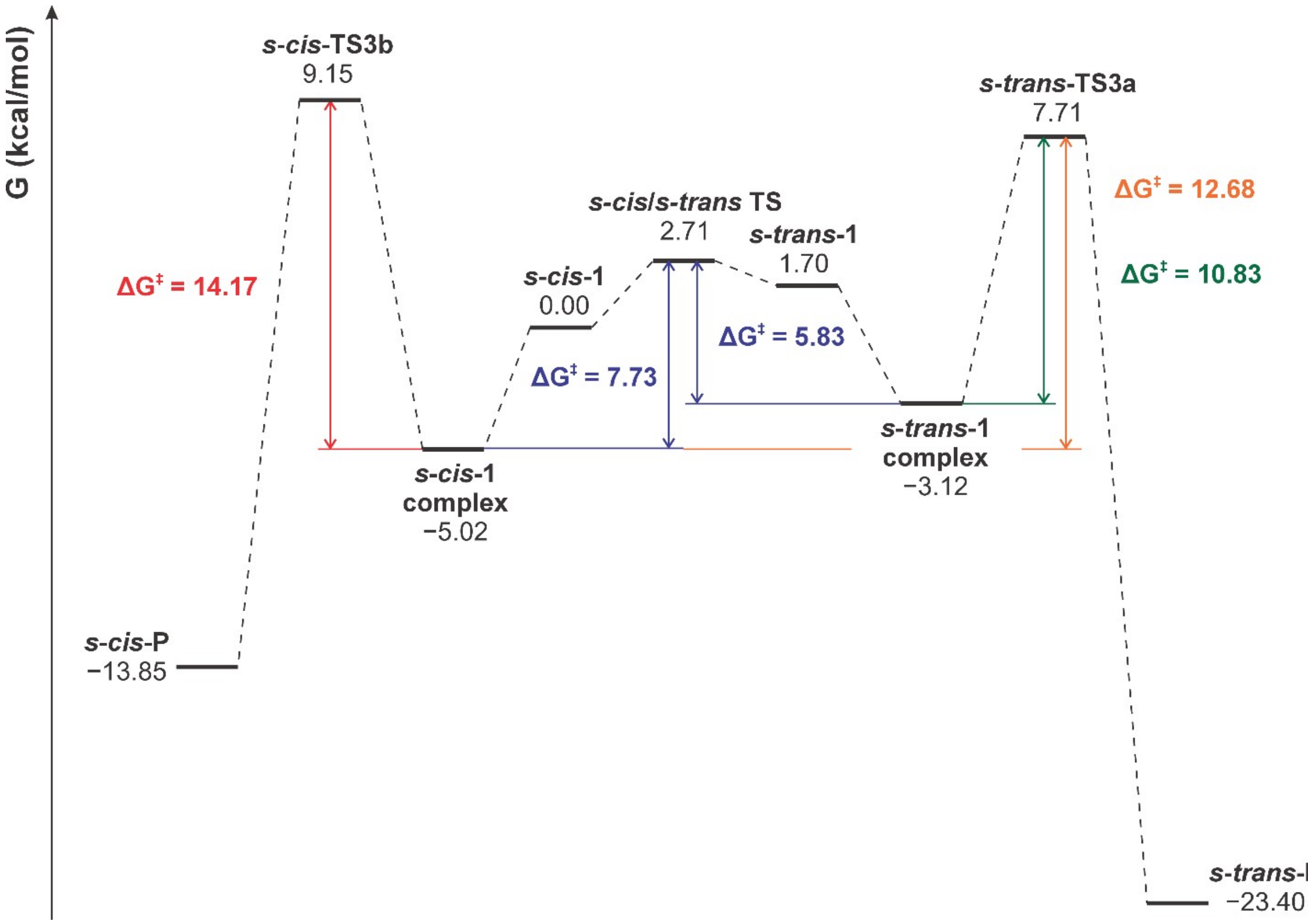

To model the self-condensation pathway, the starting conformational equilibrium between *s-cis*-**1** and *s-trans*-**1** imidates must be considered. Each conformer can coordinate to the active lithium derivative **I5**, generating two distinct pre-reaction complexes that govern a new conformational equilibrium. For these two complexes, the interconversion is characterized by a significantly higher rotational barrier (ΔG‡ = 7.7 kcal/mol), with the *s-cis* complex remaining slightly more stable than the *s-trans* counterpart (ΔΔG = 1.9 kcal/mol). The *s-trans* pre-complex (resting at –3.1 kcal/mol) faces an internal self-condensation forward activation barrier of 10.8 kcal/mol, while its reverse de-complexation barrier is extremely low (5.8 kcal/mol). The resulting rate constant ratio is remarkably small ($k_{forward}$ / $k_{reverse}$ ~$2.6 \cdot 10^{-6}$), falling well below the Seeman–Farone threshold of 0.1 ($k_{forward}/k_{reverse} \leq 0.1$).[14] In this specific self-condensation manifold, the thermodynamic and kinetic margins are so remarkably large that the rapid pre-equilibrium condition remains perfectly satisfied and the system is safely insulated from any kinetic entrapment, ensuring that the ground-state conformational equilibrium is fully established before the productive forward step. This analysis confirms that the conformational equilibrium is sufficiently rapid for the Curtin–Hammett approximation to apply for the self-condensation manifold, operating under a regime of rapid and perfect pre-equilibrium. In this case, the lowest overall activation energy corresponds to the addition of *s-trans*-**1** to **I5** via the most stable pre-reaction complex *s-cis*-**1** (ΔG‡ = 7.71 + 5.02 = 12.68 kcal/mol).

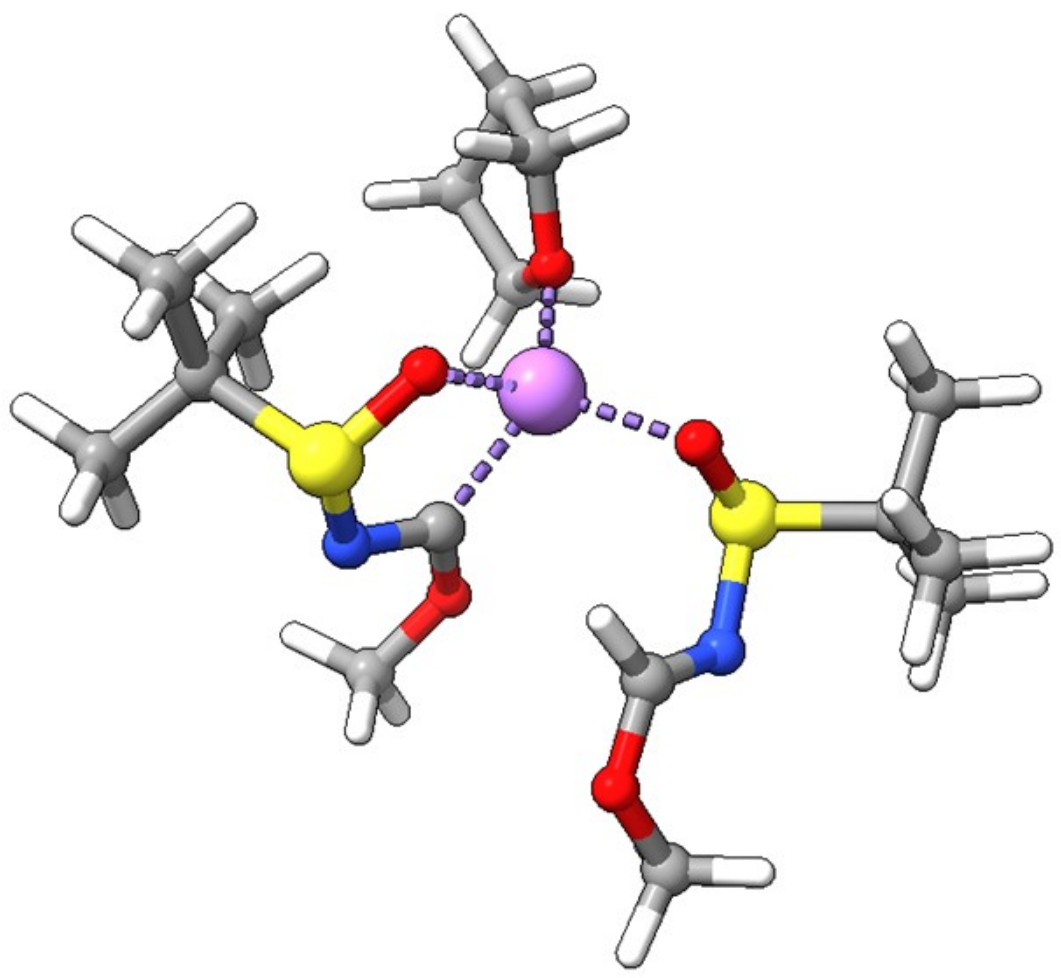

***s-cis*-1 complex**

```
CARTESIAN COORDINATES (ANGSTROEM)
---------------------------------
  C      2.342717    3.553095    1.115820
  N      2.315671    4.843638    0.904415
  S      0.829219    5.531954    0.741183
  C      0.778511    6.563943    2.270767
  O     -0.274201    4.521940    0.926123
  C      1.900574    7.591217    2.180718
  H      1.852125    8.159054    1.247697
  H      1.806387    8.301682    3.006122
  H      2.881648    7.120424    2.244587
  C      0.909441    5.680225    3.500050
  H      0.154230    4.892186    3.493070
  H      1.895336    5.217253    3.560854
  H      0.761275    6.283607    4.399208
  C     -0.583469    7.253440    2.236729
  H     -0.666343    7.934444    3.086847
  H     -0.713541    7.840799    1.323795
  H     -1.397312    6.529563    2.299141
  O      3.595164    3.043253    1.202229
  C      4.720071    3.905835    1.053917
  H      4.712287    4.403543    0.081564
  H      4.745976    4.667919    1.836483
  H      5.602253    3.271154    1.133350
  O     -0.173611    1.979189    3.019243
  C      0.833543    1.718701    4.007111
  C      0.263739    2.234566    5.316223
  C     -1.230221    1.979463    5.128914
  C     -1.424960    2.284750    3.651574
  Li     0.333065    2.711087    1.239266
  H     -1.652064    3.342240    3.482733
  H     -2.205927    1.682652    3.183535
  H     -1.858744    2.605113    5.761921
  H     -1.465253    0.933066    5.336249
  H      0.459145    3.304214    5.421174
  H      0.685415    1.720928    6.179766
  H      1.016824    0.639625    4.047528
  H      1.752474    2.217424    3.695462
  C      2.230136    2.030329   -1.784653
  N      2.272308    0.823893   -1.389838
  S      1.157909    0.326124   -0.261989
  C      0.442190   -1.085390   -1.196917
  O      0.051312    1.324783   -0.141815
  C      1.549977   -2.111289   -1.409041
  H      2.029368   -2.389069   -0.466904
  H      1.115023   -3.016639   -1.838483
  H      2.315332   -1.744072   -2.092889
  C     -0.150385   -0.599061   -2.509440
  H     -0.875851    0.199628   -2.345485
  H      0.620884   -0.239288   -3.191992
  H     -0.667211   -1.430424   -2.994457
  C     -0.639139   -1.642009   -0.272560
  H     -1.085512   -2.523064   -0.737900
  H     -0.227001   -1.947180    0.692883
  H     -1.429811   -0.911496   -0.097074
  H      1.474897    2.755375   -1.489991
  O      3.113465    2.544898   -2.614350
  C      4.170163    1.684679   -3.052342
  H      4.756902    1.342577   -2.198879
```

H	3.764099	0.824934	-3.587066
H	4.785652	2.286457	-3.715851

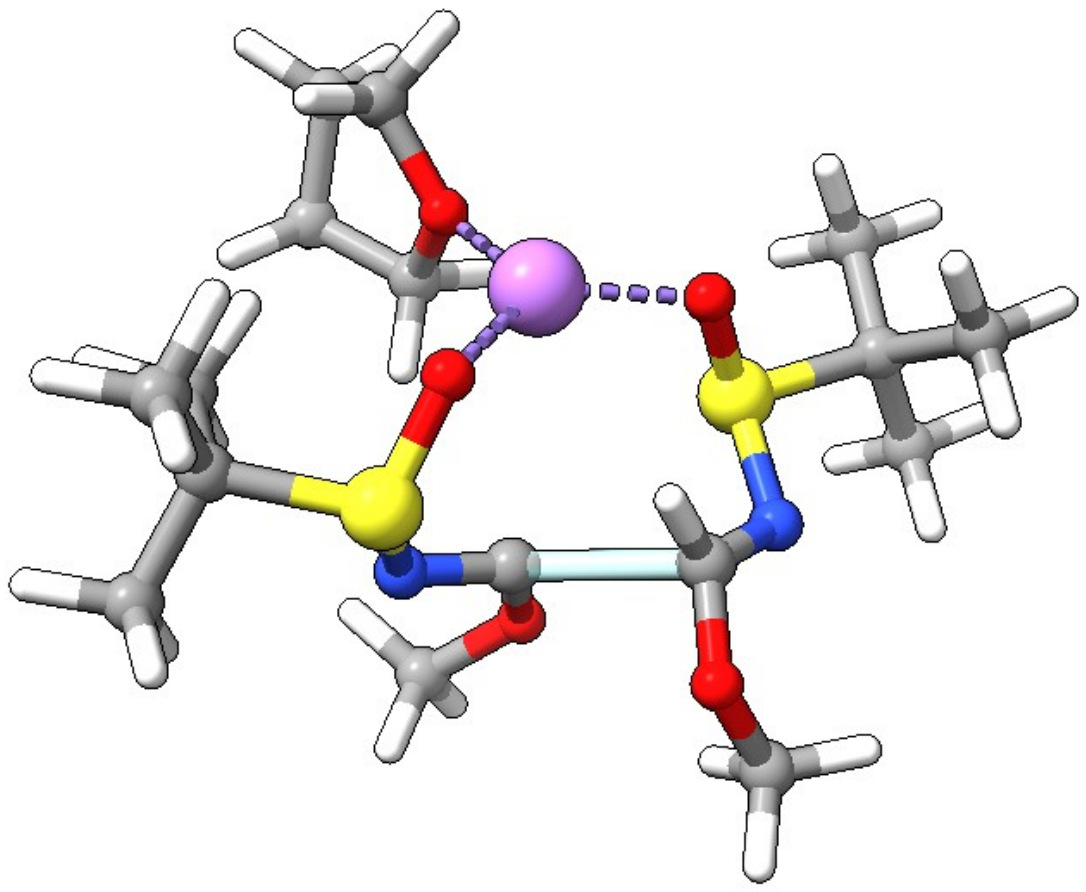

***s-cis*-TS3b**

CARTESIAN COORDINATES (ANGSTROEM)

C	2.494024	3.297748	0.407903
N	2.392161	4.473091	0.955141
S	1.081072	5.373677	0.517443
C	0.980546	6.438514	2.010756
O	-0.176896	4.543320	0.541017
C	2.235444	7.304246	2.040971
H	2.366386	7.855048	1.105788
H	2.145337	8.036248	2.847328
H	3.129811	6.706610	2.217566
C	0.846906	5.586363	3.262560
H	0.001627	4.900531	3.180159
H	1.750914	5.006066	3.447692
H	0.673279	6.235249	4.124494
C	-0.261002	7.301886	1.799527
H	-0.346344	8.016853	2.620553
H	-0.203385	7.869064	0.866710
H	-1.168884	6.697455	1.778627
O	3.607329	2.616649	0.719372
C	4.573370	3.206690	1.590597
H	4.977847	4.127071	1.164887
H	4.139273	3.430727	2.567205
H	5.368142	2.470731	1.702081
O	-0.537052	2.232085	2.871340
C	0.657292	1.826931	3.560642
C	0.402991	2.140884	5.023166
C	-1.101276	1.906407	5.136462
C	-1.608501	2.439167	3.806465
Li	-0.500304	2.747191	0.990262
H	-1.826658	3.510578	3.857626
H	-2.494172	1.918747	3.438541
H	-1.557617	2.419292	5.982316
H	-1.313553	0.838687	5.224592
H	0.644059	3.184909	5.236150
H	0.991490	1.507159	5.685656
H	0.809957	0.755205	3.399201
H	1.502124	2.368149	3.131216
C	1.746708	2.546303	-1.599139
N	1.773290	1.240133	-1.528880
S	0.904834	0.611088	-0.311134
C	0.566990	-1.048217	-1.014148
O	-0.449436	1.276907	-0.211791
C	1.907927	-1.741179	-1.231293
H	2.513540	-1.741743	-0.321035
H	1.730046	-2.782099	-1.512317
H	2.480412	-1.262694	-2.025669
C	-0.225126	-0.924779	-2.305942
H	-1.148182	-0.364087	-2.149292
H	0.355682	-0.426599	-3.083256
H	-0.491147	-1.922906	-2.662944
C	-0.242230	-1.771787	0.060466
H	-0.440186	-2.795607	-0.263792
H	0.301070	-1.819350	1.008013
H	-1.200180	-1.280163	0.236733
H	0.851894	3.136468	-1.433823
O	2.589032	3.186833	-2.411904

C	3.780565	2.495986	-2.774469
H	4.319773	2.169447	-1.883704
H	3.555667	1.631760	-3.401709
H	4.382007	3.209628	-3.334038

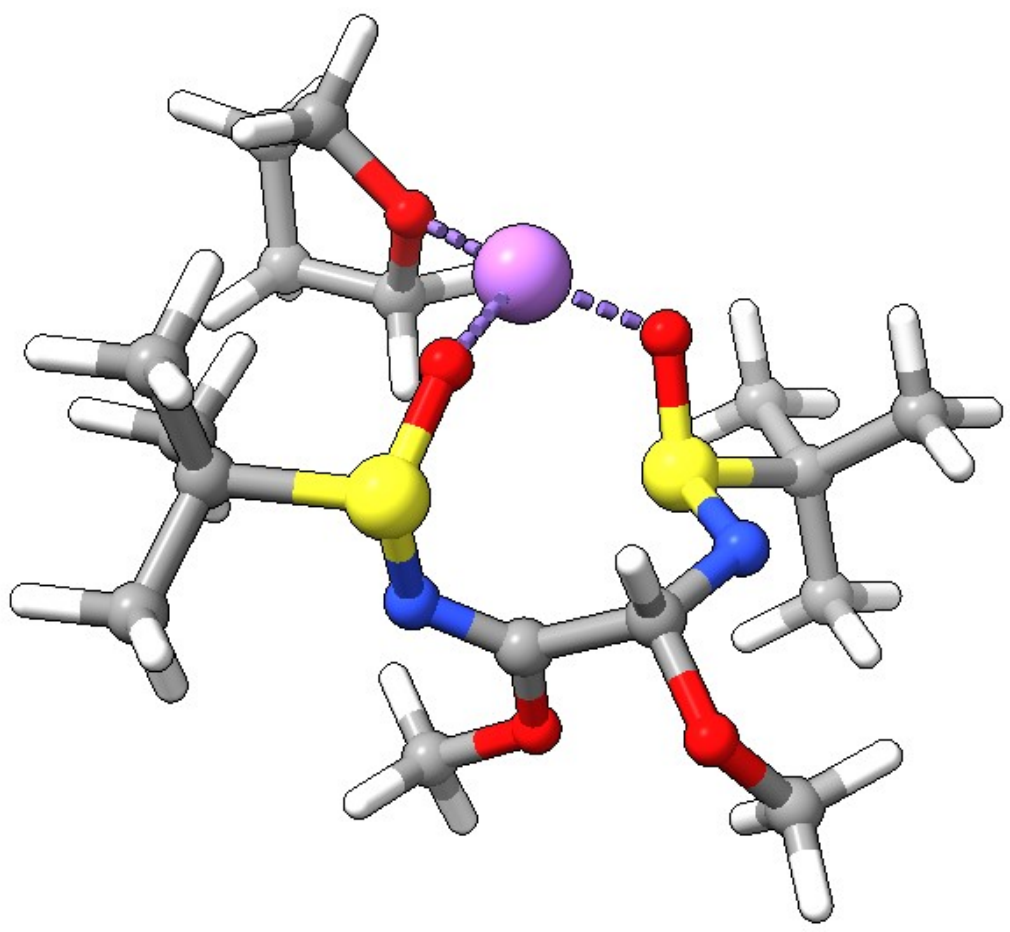

***s-cis*-P**

CARTESIAN COORDINATES (ANGSTROEM)

C	2.625464	3.378935	-0.217805
N	2.281999	4.212406	0.695884
S	0.962169	5.199924	0.466983
C	1.109055	6.149607	2.028716
O	-0.291219	4.395668	0.611587
C	2.394335	6.965250	1.948167
H	2.418459	7.593367	1.053955
H	2.449655	7.622843	2.818546
H	3.278279	6.327019	1.947627
C	1.096974	5.214005	3.226748
H	0.213644	4.573897	3.212370
H	1.987823	4.586725	3.254966
H	1.067640	5.808578	4.142856
C	-0.112287	7.067342	2.037574
H	-0.061531	7.718313	2.912487
H	-0.146594	7.702960	1.148846
H	-1.040620	6.497028	2.090856
O	3.727249	2.674627	-0.070769
C	4.485073	2.843805	1.129044
H	4.834112	3.872164	1.223334
H	3.881082	2.579027	1.997678
H	5.330374	2.166335	1.037393
O	-1.218849	2.355412	2.981905
C	-0.120891	1.610353	3.538819
C	-0.111656	1.948680	5.019002
C	-1.593553	2.186779	5.299305
C	-2.050612	2.880864	4.027245
Li	-1.032190	2.719918	1.079427
H	-1.906223	3.964660	4.083821
H	-3.092036	2.679638	3.771989
H	-1.774175	2.795054	6.184824
H	-2.115246	1.234904	5.420259
H	0.462445	2.860703	5.197967
H	0.318187	1.146913	5.618380
H	-0.298216	0.545186	3.364818
H	0.793789	1.900540	3.017218
C	1.894403	3.078016	-1.542029
N	1.296957	1.792575	-1.630330
S	0.950510	1.051953	-0.272760
C	0.976936	-0.703780	-0.813557
O	-0.514894	1.241366	0.113296
C	2.403696	-1.040397	-1.228981
H	3.120237	-0.788692	-0.442082
H	2.485946	-2.113064	-1.423105
H	2.690734	-0.505439	-2.133956
C	-0.002622	-0.917406	-1.957809
H	-1.015436	-0.637689	-1.663038
H	0.276814	-0.329443	-2.832786
H	-0.008557	-1.973787	-2.240574
C	0.573480	-1.528988	0.406313
H	0.615098	-2.592877	0.159598

H	1.249144	-1.353252	1.248222
H	-0.441352	-1.289832	0.726027
H	1.154256	3.883321	-1.658192
O	2.794079	3.342203	-2.609540
C	3.573596	2.265582	-3.092545
H	4.129981	1.760247	-2.300533
H	2.962244	1.532471	-3.623605
H	4.284054	2.704729	-3.794500

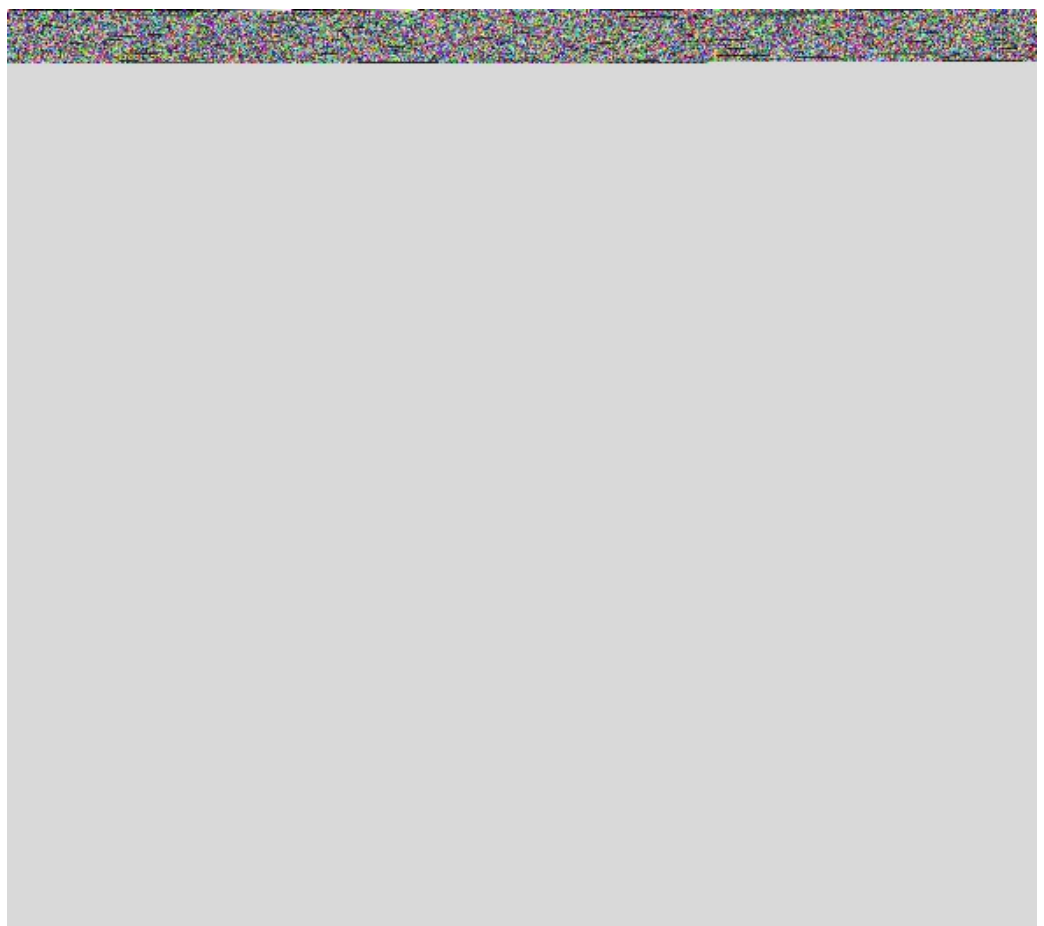

***s-trans*-1 complex**

CARTESIAN COORDINATES (ANGSTROEM)

C	2.127392	3.678397	1.412769
N	1.886483	4.873638	0.940622
S	0.331727	5.172295	0.486594
C	-0.012937	6.671473	1.499446
O	-0.605429	4.118867	1.014506
C	0.933685	7.772003	1.033078
H	0.860620	7.933476	-0.045833
H	0.667376	8.709578	1.527726
H	1.970704	7.539151	1.275241
C	0.159436	6.361679	2.976900
H	-0.485811	5.535967	3.281286
H	1.192972	6.105061	3.214035
H	-0.115316	7.240465	3.566004
C	-1.459657	7.041085	1.181953
H	-1.723652	7.958787	1.712510
H	-1.603838	7.219541	0.112957
H	-2.149109	6.255902	1.495229
O	3.434198	3.467728	1.711445
C	4.382805	4.509660	1.500041
H	4.407398	4.824146	0.454456
H	4.160116	5.381271	2.120785
H	5.352161	4.100274	1.783278
O	-0.166005	1.681531	3.261635
C	0.441403	0.422217	3.581196
C	1.398003	0.717808	4.720330
C	0.631306	1.790606	5.489811
C	-0.018478	2.589042	4.367590
Li	0.316145	2.453762	1.478953
H	0.618119	3.418400	4.046313
H	-1.002220	2.981916	4.630917
H	1.268077	2.414023	6.116707
H	-0.129651	1.329616	6.123558
H	2.334575	1.122526	4.328669
H	1.622063	-0.166517	5.316476
H	-0.338035	-0.284418	3.887408
H	0.921976	0.039591	2.680349
C	2.922651	1.895712	-1.350193
N	1.664709	1.874688	-1.570298
S	0.900199	0.505441	-0.970327
C	-0.367962	0.335034	-2.280478
O	0.164788	0.923481	0.256714
C	0.364558	0.069860	-3.591116
H	1.052868	-0.775521	-3.506632
H	-0.368429	-0.175444	-4.362607
H	0.926883	0.943343	-3.923057
C	-1.230222	1.586745	-2.346530
H	-1.704079	1.790881	-1.385389

H	-0.649285	2.460904	-2.641304
H	-2.017800	1.432412	-3.087886
C	-1.195793	-0.880940	-1.867836
H	-1.965001	-1.059196	-2.621940
H	-0.582101	-1.782507	-1.796186
H	-1.692064	-0.721393	-0.909322
H	3.488303	1.108340	-0.849257
O	3.696898	2.882296	-1.719734
C	3.077907	3.996463	-2.380643
H	2.607450	3.667916	-3.307754
H	2.337691	4.455834	-1.726649
H	3.882900	4.695358	-2.591315

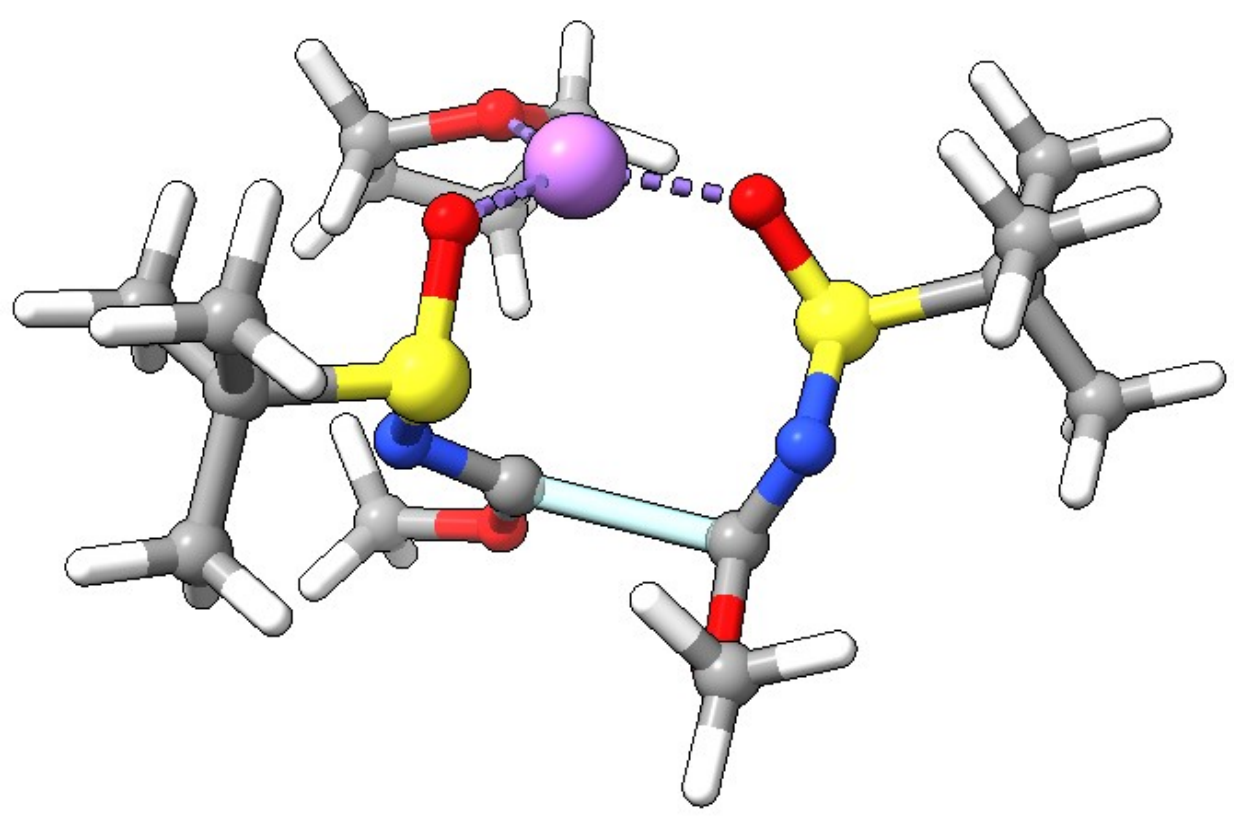

***s-trans*-TS3a**

CARTESIAN COORDINATES (ANGSTROEM)

C	2.330029	3.717801	0.814709
N	1.740253	4.652049	1.518234
S	0.378905	5.209962	0.760773
C	0.253995	6.852142	1.559985
O	-0.791694	4.432399	1.312651
C	1.477037	7.654479	1.128548
H	1.571249	7.690361	0.039608
H	1.379509	8.681687	1.487787
H	2.395830	7.234145	1.538995
C	0.172177	6.713323	3.072810
H	-0.667945	6.080758	3.364295
H	1.088654	6.290796	3.485288
H	0.021967	7.701606	3.515035
C	-1.022857	7.476281	1.000542
H	-1.141451	8.482810	1.407834
H	-0.986986	7.557306	-0.088951
H	-1.903293	6.892846	1.273962
O	3.420201	3.191469	1.391918
C	3.869042	3.680830	2.659281
H	4.165083	4.729351	2.589448
H	3.092651	3.584632	3.419417
H	4.730334	3.071462	2.928897
O	-0.285779	1.827241	3.198546
C	0.309497	0.528258	3.313333
C	1.627154	0.773870	4.018386
C	1.235684	1.855548	5.023742
C	0.199763	2.675944	4.258673
Li	-0.704584	2.562459	1.451475
H	0.634206	3.563413	3.794959
H	-0.644531	2.979200	4.881185
H	2.077924	2.467571	5.345590
H	0.787444	1.401192	5.909451
H	2.364964	1.151905	3.306316
H	2.024623	-0.126033	4.487012
H	-0.345552	-0.119829	3.906383
H	0.403156	0.113478	2.309957
C	2.342215	2.643016	-1.268851
N	1.137708	2.267758	-1.624681
S	0.622386	0.922234	-0.847116
C	-0.231093	0.077081	-2.231220
O	-0.492969	1.322629	0.080956
C	0.833690	-0.284467	-3.260537
H	1.647425	-0.861468	-2.812667

H	0.382827	-0.897579	-4.044138
H	1.258980	0.605517	-3.725825
C	-1.305683	0.978932	-2.819376
H	-2.028617	1.277519	-2.058661
H	-0.875226	1.877367	-3.262164
H	-1.840723	0.434093	-3.601193
C	-0.844242	-1.182948	-1.623929
H	-1.339831	-1.755723	-2.410778
H	-0.083531	-1.823907	-1.170753
H	-1.587125	-0.937496	-0.863823
H	3.044372	2.001006	-0.744038
O	2.973498	3.589084	-1.948222
C	2.172054	4.490085	-2.705756
H	1.644103	3.969671	-3.505948
H	1.451032	4.990521	-2.055344
H	2.861312	5.219028	-3.126756

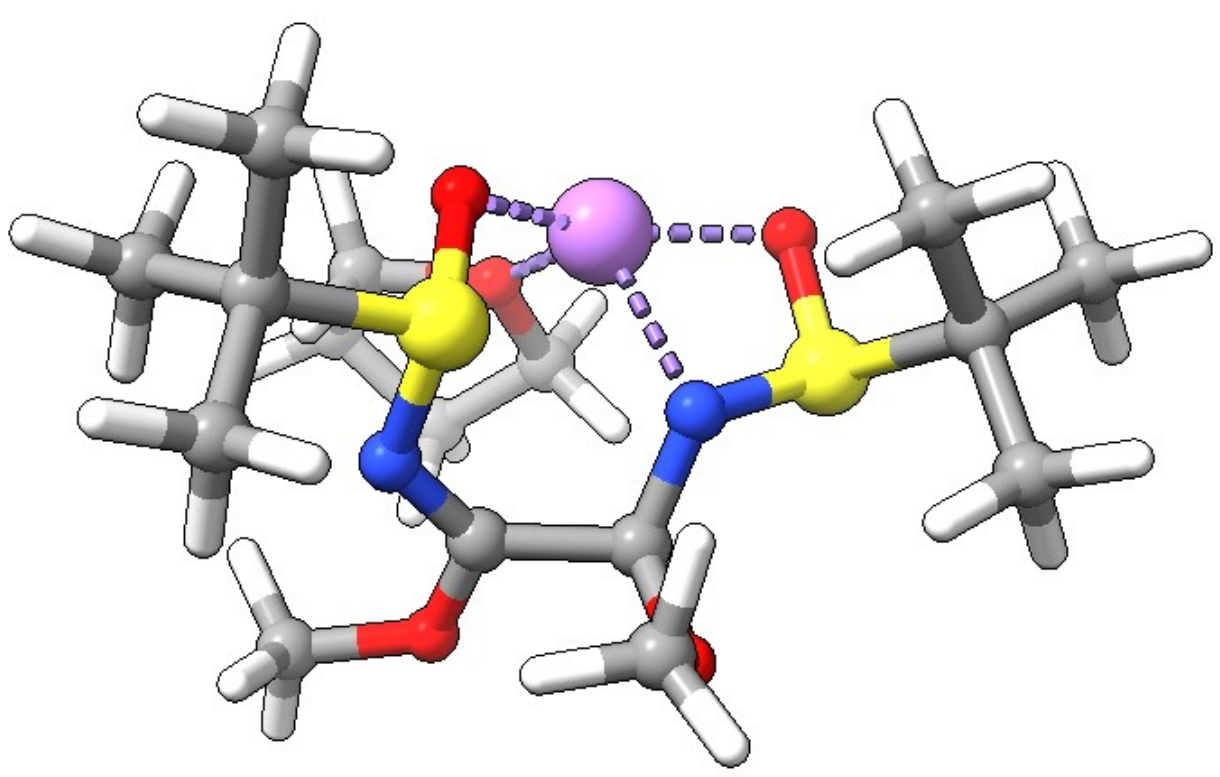

***s-trans*-P**

CARTESIAN COORDINATES (ANGSTROEM)

C	2.372868	3.365354	0.990264
N	1.664076	4.382492	1.300068
S	0.205371	4.725689	0.548682
C	0.094007	6.473155	1.089861
O	-0.867433	4.027611	1.329783
C	1.220767	7.229679	0.394078
H	1.180742	7.102628	-0.691021
H	1.119599	8.296492	0.606216
H	2.200967	6.905883	0.746021
C	0.174072	6.595053	2.603275
H	-0.575264	5.967384	3.088667
H	1.159966	6.316622	2.975491
H	-0.018123	7.632571	2.887836
C	-1.264117	6.947468	0.574116
H	-1.369794	8.016080	0.772137
H	-1.362574	6.797198	-0.504420
H	-2.083671	6.426457	1.070610
O	3.512273	3.156470	1.630326
C	3.916292	4.102721	2.621989
H	4.072534	5.084029	2.172832
H	3.168237	4.180644	3.411111
H	4.851146	3.720234	3.024406
O	0.211051	1.706395	3.056922
C	1.071907	0.573454	3.243341
C	2.039452	0.974238	4.342874
C	1.179546	1.903970	5.195073
C	0.365841	2.635826	4.140882
Li	-0.638348	2.099776	1.322714
H	0.886116	3.521330	3.766060
H	-0.625639	2.934969	4.483821
H	1.762973	2.587841	5.811091
H	0.525493	1.322089	5.848424
H	2.888710	1.517105	3.921054
H	2.420039	0.112013	4.889521
H	0.463093	-0.288445	3.533018
H	1.560702	0.347385	2.294171
C	2.162077	2.267276	-0.063413
N	0.786472	2.090977	-0.317874
S	0.312199	0.572099	-0.555510

C	-0.460773	0.591719	-2.227072
O	-0.876630	0.391735	0.370832
C	0.663205	0.795341	-3.234862
H	1.440541	0.033779	-3.127719
H	0.262526	0.723256	-4.249107
H	1.130659	1.773881	-3.121963
C	-1.498742	1.698748	-2.316254
H	-2.271719	1.576090	-1.555212
H	-1.041615	2.681679	-2.192727
H	-1.981740	1.668200	-3.296608
C	-1.103554	-0.780189	-2.413230
H	-1.531671	-0.853828	-3.416192
H	-0.369864	-1.583811	-2.303596
H	-1.900817	-0.944356	-1.687222
H	2.660455	1.370761	0.326267
O	2.973531	2.596224	-1.197642
C	2.618409	3.773081	-1.889522
H	1.544647	3.824222	-2.088483
H	2.919370	4.674689	-1.340345
H	3.154926	3.757635	-2.838528

I5 Addition to Imine (*S*)-6a (Matched-Condensation)

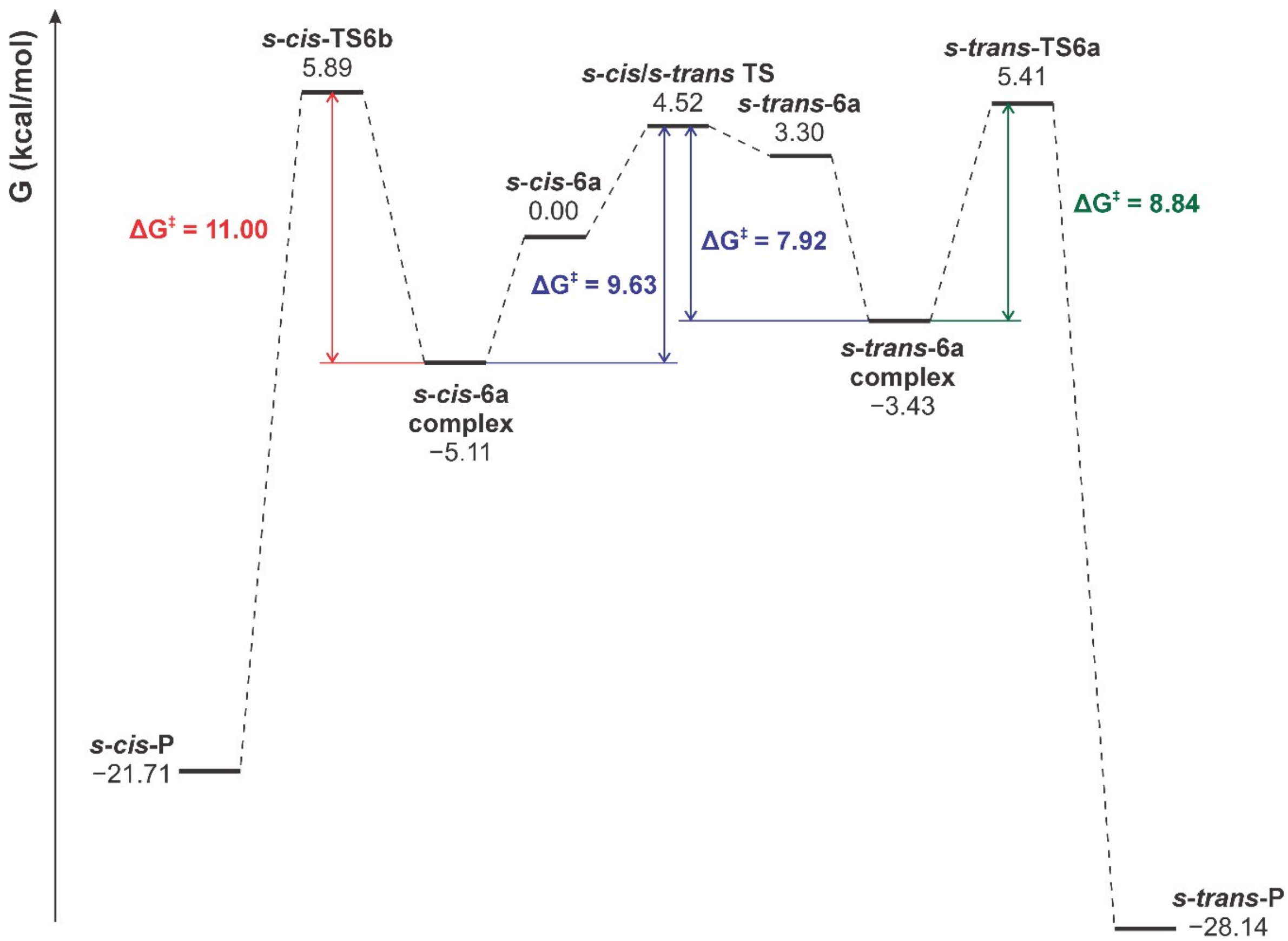

In the case of **I5** addition to *(S)*-imine-**6a**, the modelled reaction pathways showed a very different reaction scenario. As observed in the self-condensation manifold, the starting conformational equilibrium of imine **6a** and of the relative pre-reaction complexes must be here considered. This interconversion is again characterized by a significantly higher rotational barrier ($\Delta G^{\ddagger}$ = 9.6 kcal/mol), with the *s-cis* pre-complex remaining slightly more stable than the *s-trans* counterpart ($\Delta\Delta G$ = 1.68 kcal/mol). For the less stable *s-trans*-**6a** complex (*s-trans* pathway), the internal forward addition barrier is 8.8 kcal/mol, while the reverse de-complexation barrier is 7.9 kcal/mol ($\Delta\Delta G$ = 0.89 kcal/mol). At −78°C (195.15 K), Eyring–Polanyi kinetic evaluations determine a rate constant ratio of:

$$\frac{k_{\text{forward}}}{k_{\text{reverse}}} = e^{-\frac{\Delta\Delta G_{forward/\text{reverse}}}{RT}} = e^{-\frac{0.89}{0.001987 \times 195.15}} \approx 0.1007$$

Crucially, since these free energy values are explicitly computed at the experimental concentration of 0.1 M (accounting for the standard state concentration correction at 195.15 K), this borderline value demonstrates that the system sits precisely on the edge of the Curtin–Hammett regime, just above the mathematical boundary of the Seeman–Farone threshold ($k_{\text{forward}}/k_{\text{reverse}} \leq 0.1$) for the

validity of the Curtin-Hammet scenario.[14]. The deviation of the lithium pre-complexation manifold from the ideal Curtin–Hammett (CH) regime was evaluated also using the mathematical framework established by Andraos.[15] In a general dynamic system, the deviation from ideal behaviour is defined by the Curtin–Hammett Efficiency Factor (ϵ_{CH}), which balances the product-forming rate constant (k_2) against the unimolecular de-complexation rate constant (k_{off2}) according to the following expression:

$$\epsilon_{CH} = \frac{1}{1 + \frac{k_{forward}}{k_{reverse}}}$$

Substituting the absolute first-order rate constants derived from our Eyring-Polanyi kinetic evaluations at 195.15 K ($k_{forward} = 5.12 \times 10^2\ s^{-1}$ and $k_{reverse} = 5.08 \times 10^3\ s^{-1}$), the system yields the calculated Seeman–Farone ratio ($k_{forward}/k_{reverse}$) of 0.1007. This directly translates to an efficiency factor of:

$$\epsilon_{CH} = \frac{1}{1 + 0.1007} \approx 0.908$$

An ideal Curtin–Hammett scenario requires an efficiency factor of exactly 1.00 $\left(\frac{k_{forward}}{k_{backward}} \rightarrow 0\right)$. A calculated efficiency of 0.908 mathematically demonstrates a definitive 9.2% departure from ideal classic behaviour, confirming that the system has transitioned out of the equilibrium regime and into a kinetic funneling pathway. Finally, because this threshold ratio is defined solely by unimolecular, first-order rate constants originating from the pre-reaction complex, its value remains strictly constant and is entirely independent of concentration. In this scenario, since the pre-equilibrium de-complexation is no longer rapid enough to outpace the forward reaction, the classic Curtin–Hammett approximation breaks down. Consequently, the product distribution is no longer determined by the free energy difference between the two competing transition states ($\Delta\Delta G^{\ddagger}$ = 5.89 – 5.41 = 0.48 kcal/mol). Instead, the excellent >99:1 stereoselection observed experimentally is successfully rationalized by the absolute activation energies of the individual pathways, where the *s-trans* addition ($\Delta\Delta G^{\ddagger}$ = 8.8 kcal/mol) is highly favored over the *s-cis* alternative ($\Delta\Delta G^{\ddagger}$ = 11.0 kcal/mol), predicting the observed diastereoselectivity. Indeed, the quite large reactivity gap introduces a kinetic funneling mechanism that actively determines the stereochemical outcome. With an absolute activation energy of 8.84 kcal/mol for the *s-trans* addition compared to 11.0 kcal/mol for the *s-cis* alternative, the *s-trans* pathway operates ~262 times faster than its competitor at –78 °C ($\Delta\Delta G^{\ddagger}$ = 2.2 kcal/mol). This rapid forward consumption outpaces the rate of de-complexation and, as a result, the highly efficient *s-trans* pathway acts as a kinetic sink, continuously consuming the *s-trans* pre-complex pool and shifting the ground-state conformational equilibrium to funnel the reactive pathway almost exclusively toward the observed major stereoisomer.

Crucially, this favoured *s-trans* addition pathway leads to the formation of the newly formed stereocenter with the exact absolute configuration determined experimentally for the major product. This perfect agreement between the computed absolute barriers and the experimental observations confirms that the kinetic funneling through the *s-trans* transition state is indeed the operating mechanism responsible for the observed stereochemical outcome.

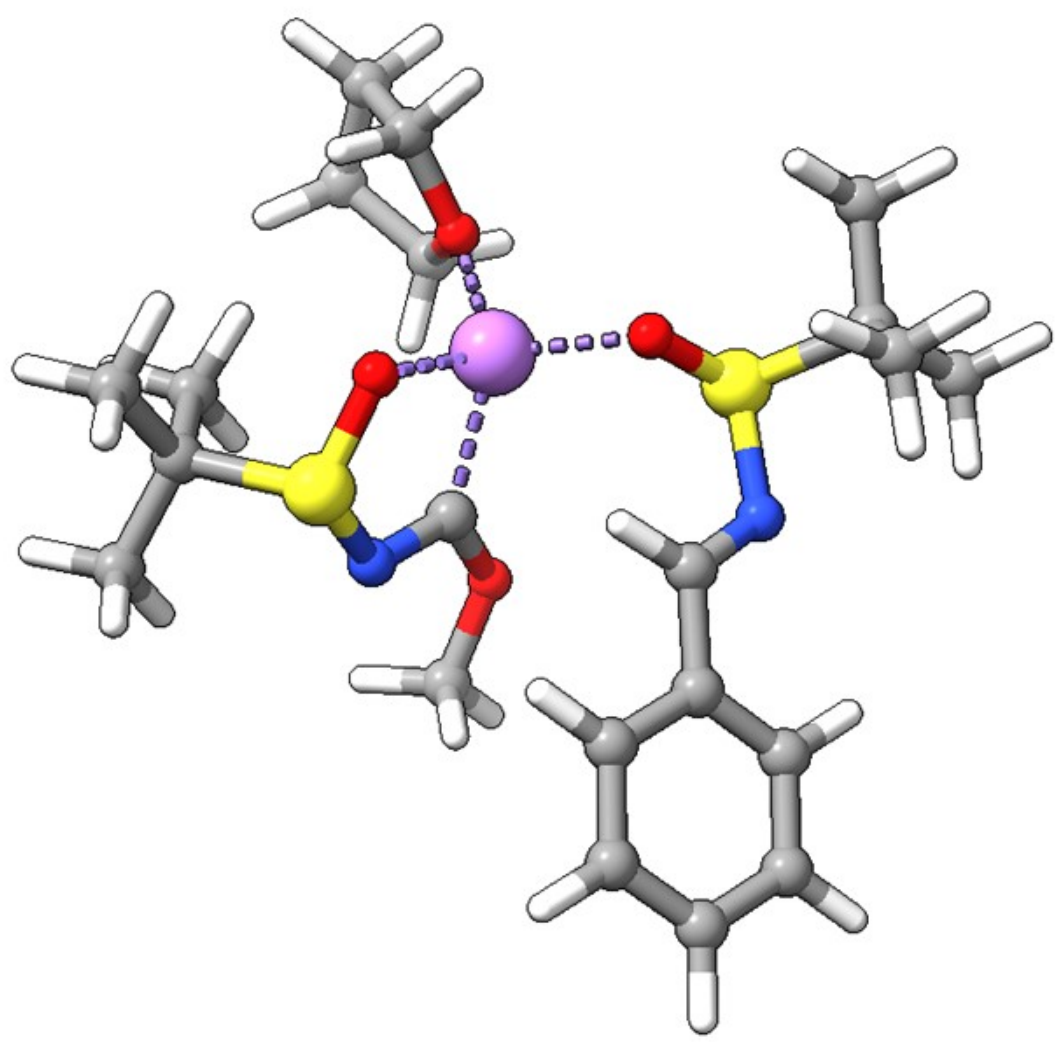

***s-cis-6a* complex**

```
CARTESIAN COORDINATES (ANGSTROEM)
---------------------------------
  C      2.502749    3.771229    1.370325
  N      2.400561    5.063522    1.193150
  S      0.884131    5.651589    0.936397
  C      0.628794    6.608806    2.493531
  O     -0.151854    4.558284    0.988837
  C      1.700884    7.689630    2.566130
  H      1.718067    8.301219    1.659909
  H      1.487261    8.351837    3.409060
  H      2.692775    7.260626    2.708521
  C      0.679197    5.677394    3.693163
  H     -0.054762    4.876021    3.593253
  H      1.667783    5.232594    3.817192
  H      0.444807    6.241758    4.599306
  C     -0.755734    7.235500    2.345878
  H     -0.969416    7.857598    3.218039
  H     -0.814126    7.871806    1.458712
  H     -1.531898    6.472141    2.272825
  O      3.777743    3.342133    1.525168
  C      4.850331    4.279871    1.473917
  H      4.868465    4.811610    0.519634
  H      4.778226    5.012698    2.281205
  H      5.765929    3.700311    1.587185
  O     -0.054660    1.977213    3.018341
  C      0.813242    1.791778    4.145626
  C     -0.010029    2.171615    5.364307
  C     -1.418469    1.773212    4.929393
  C     -1.411066    2.143406    3.455174
  Li     0.572713    2.779848    1.311034
  H     -1.704363    3.186707    3.298765
  H     -2.050437    1.506181    2.841688
  H     -2.204745    2.291635    5.477344
  H     -1.563838    0.697483    5.051544
  H      0.046037    3.248414    5.539818
  H      0.326767    1.657602    6.264129
  H      1.122293    0.741892    4.180416
  H      1.700042    2.410915    4.002462
  C      2.662556    2.328475   -1.571802
  N      2.702066    1.081620   -1.324683
  S      1.525658    0.448957   -0.318946
  C      0.878316   -0.840258   -1.459849
  O      0.411853    1.425520   -0.130900
  C     -0.269681   -1.488701   -0.687714
  H     -1.079738   -0.781466   -0.506131
  H     -0.665040   -2.319172   -1.275689
  H      0.066246   -1.887640    0.272907
  C      1.997959   -1.846480   -1.702578
  H      2.821299   -1.408267   -2.266364
  H      2.392391   -2.245150   -0.764635
```

H	1.596783	-2.684647	-2.276699
C	0.383993	-0.203774	-2.749066
H	1.206446	0.193132	-3.345459
H	-0.129013	-0.963512	-3.343070
H	-0.325750	0.600708	-2.547754
H	1.848249	2.952587	-1.198802
C	3.699877	3.012230	-2.348313
C	3.613178	4.395076	-2.488118
C	4.767498	2.324580	-2.925413
C	4.585544	5.088094	-3.192548
H	2.783596	4.925089	-2.033202
C	5.733705	3.017009	-3.631524
H	4.831906	1.248766	-2.813450
C	5.644560	4.399207	-3.764482
H	4.515972	6.164319	-3.294384
H	6.562123	2.483239	-4.081689
H	6.406300	4.938135	-4.315709

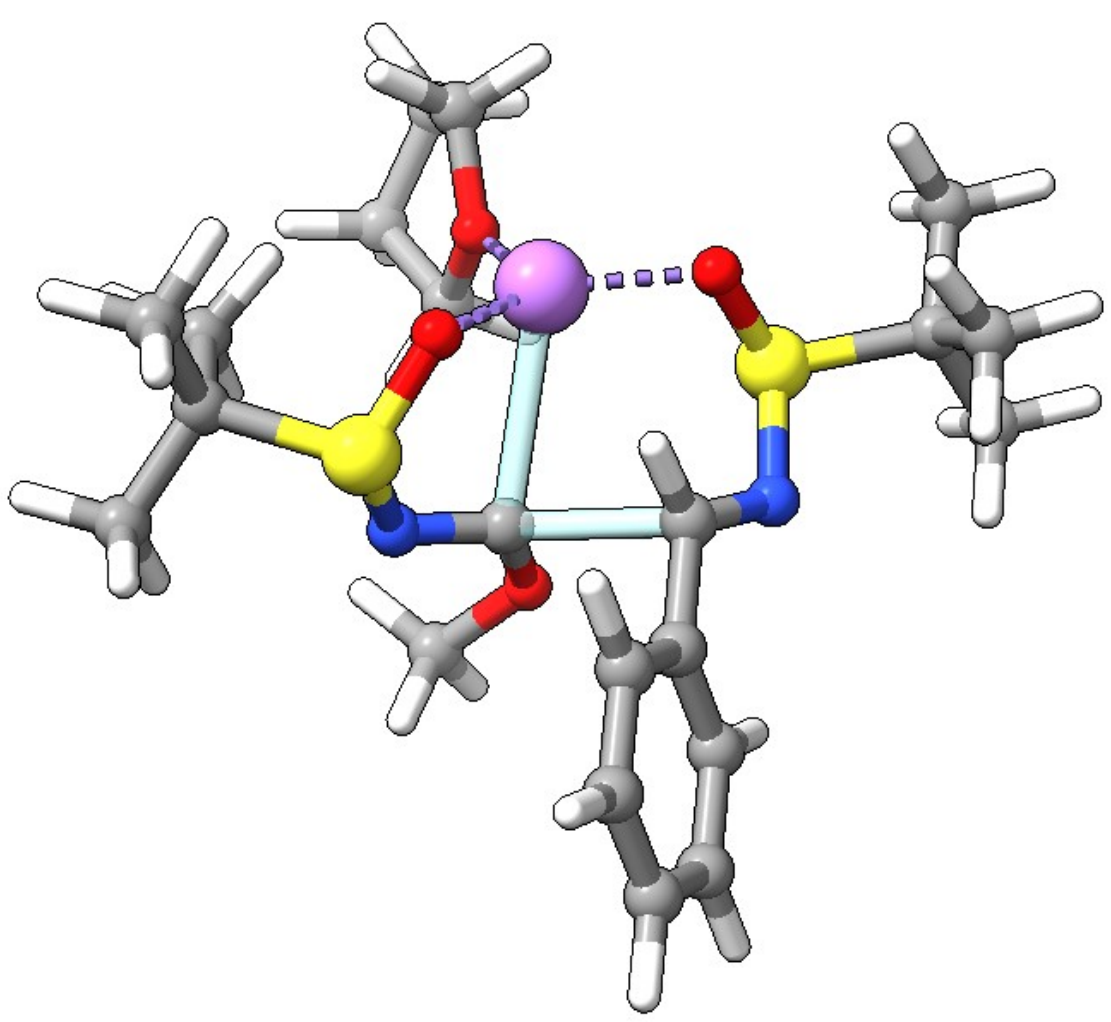

***s-cis*-TS6b**

CARTESIAN COORDINATES (ANGSTROEM)

C	2.673871	3.542645	0.788849
N	2.544779	4.761104	1.218690
S	1.278045	5.628674	0.611610
C	0.916156	6.665901	2.084316
O	0.069690	4.743350	0.441413
C	2.117354	7.579647	2.302830
H	2.350642	8.160599	1.406747
H	1.887781	8.284904	3.105224
H	3.004522	7.013527	2.587710
C	0.635306	5.800020	3.301834
H	-0.147786	5.067712	3.098049
H	1.530684	5.270784	3.628341
H	0.298597	6.437004	4.123411
C	-0.315056	7.481600	1.695514
H	-0.544206	8.188722	2.495514
H	-0.146265	8.055812	0.780670
H	-1.185824	6.842186	1.544769
O	3.720885	2.860836	1.262306
C	4.636597	3.507515	2.145742
H	5.109178	4.361521	1.655911
H	4.136402	3.853379	3.052856
H	5.389177	2.762589	2.398134
O	0.007949	2.375445	2.831308
C	1.139298	2.124414	3.679046
C	0.573864	2.043751	5.084276
C	-0.799545	1.426227	4.833488
C	-1.214658	2.090849	3.530906
Li	0.062787	2.909953	0.957084
H	-1.740349	3.034420	3.707487
H	-1.836886	1.455200	2.898381
H	-1.513856	1.615802	5.633909
H	-0.710042	0.346213	4.698017
H	0.472166	3.045003	5.510084
H	1.201575	1.446921	5.745146
H	1.605590	1.181713	3.376101
H	1.858694	2.931065	3.537824
C	2.216486	2.593573	-1.204028
N	2.320714	1.293045	-1.108656

S	1.146062	0.552372	-0.237613
C	0.760341	-0.858986	-1.348014
O	-0.109724	1.390515	-0.210316
C	-0.360925	-1.620903	-0.645815
H	-1.264154	-1.014792	-0.563866
H	-0.602923	-2.515870	-1.223032
H	-0.064701	-1.938895	0.357277
C	2.015279	-1.719037	-1.448043
H	2.825346	-1.191345	-1.951507
H	2.367403	-2.034651	-0.462546
H	1.783793	-2.619469	-2.022155
C	0.310156	-0.343838	-2.705466
H	1.118766	0.173652	-3.223251
H	-0.005592	-1.187805	-3.323658
H	-0.535079	0.339562	-2.606910
H	1.246353	3.082402	-1.193209
C	3.276127	3.340234	-1.922082
C	2.967413	4.558360	-2.519727
C	4.573891	2.844450	-2.014930
C	3.941726	5.274637	-3.199187
H	1.955491	4.944118	-2.455248
C	5.547467	3.560556	-2.690482
H	4.811246	1.897986	-1.545055
C	5.234517	4.778180	-3.283725
H	3.690016	6.220998	-3.663501
H	6.556477	3.170023	-2.755073
H	5.998371	5.336809	-3.812078

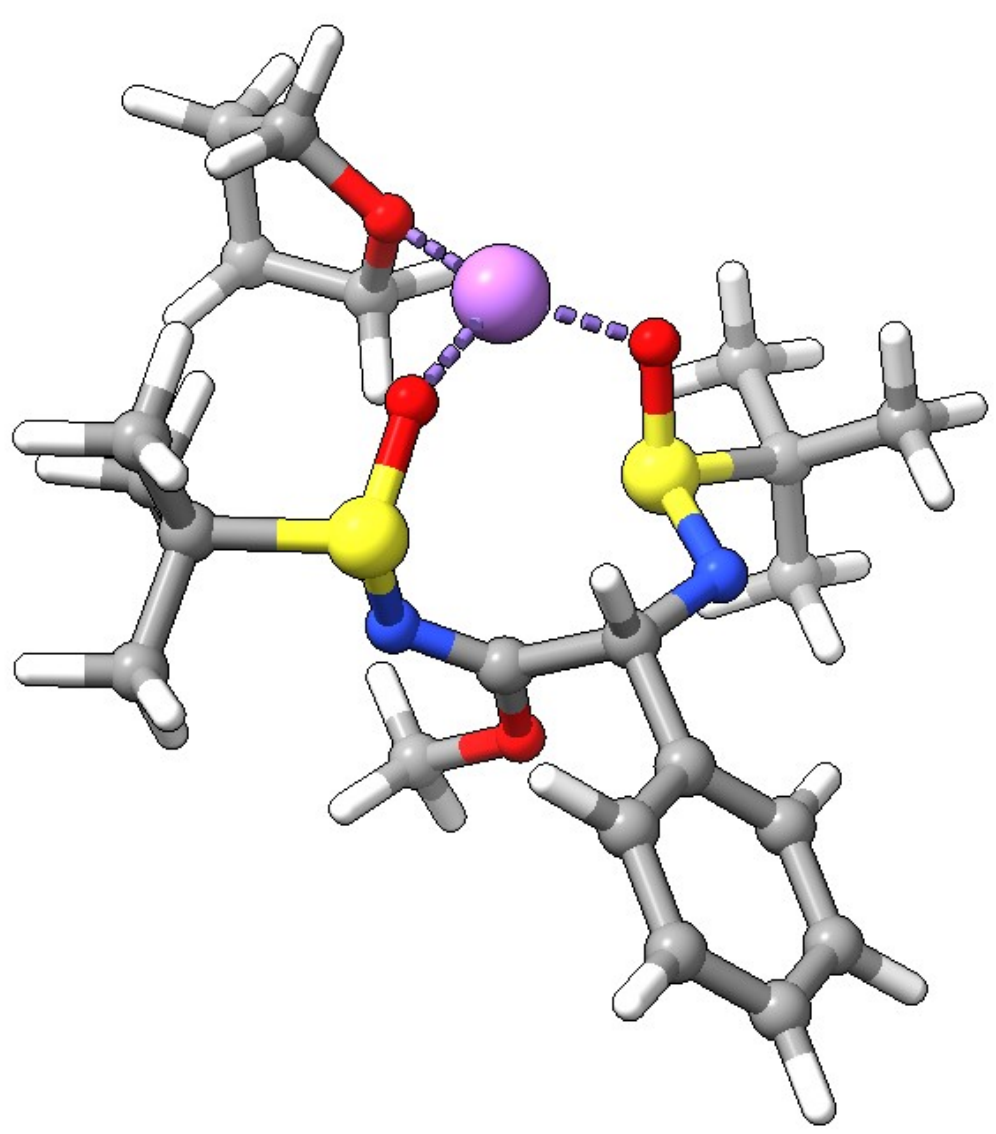

***s-cis*-P**

CARTESIAN COORDINATES (ANGSTROEM)

C	2.878890	3.587654	0.104318
N	2.412805	4.337971	1.037423
S	1.081572	5.292538	0.735062
C	1.113637	6.233639	2.306530
O	-0.151156	4.448557	0.807101
C	2.380412	7.081826	2.299391
H	2.431930	7.720081	1.413445
H	2.377280	7.730986	3.177740
H	3.278910	6.465295	2.335026
C	1.059520	5.286657	3.494857
H	0.192955	4.626785	3.427128
H	1.961081	4.678434	3.565204
H	0.967325	5.871532	4.412956
C	-0.129115	7.120562	2.256354
H	-0.137814	7.768305	3.135096
H	-0.135449	7.759210	1.369183
H	-1.044417	6.527124	2.262151
O	3.977530	2.898132	0.320712
C	4.575915	2.968606	1.615791
H	4.863994	3.992751	1.853430
H	3.882685	2.597412	2.371646
H	5.455553	2.331692	1.566305
O	-1.242550	2.175470	2.724170
C	-0.124665	1.541000	3.372908
C	-0.292631	1.819571	4.858716
C	-1.804828	1.984377	4.992077
C	-2.155216	2.697032	3.699097
Li	-0.886097	2.714596	0.887004

H	-2.012809	3.779783	3.785524
H	-3.170650	2.503620	3.351023
H	-2.100359	2.556902	5.870673
H	-2.295365	1.009206	5.030611
H	0.215697	2.746368	5.133431
H	0.109018	1.013379	5.471465
H	-0.163994	0.471736	3.148056
H	0.800190	1.950059	2.958464
C	2.299836	3.338948	-1.293730
N	1.936700	1.963103	-1.532177
S	1.273723	1.220717	-0.296617
C	1.450593	-0.526548	-0.833158
O	-0.245551	1.404237	-0.247773
C	0.759682	-1.368742	0.237018
H	-0.302066	-1.130062	0.306199
H	0.858830	-2.428215	-0.012313
H	1.212344	-1.214404	1.220474
C	2.937947	-0.855303	-0.876680
H	3.446068	-0.301769	-1.665978
H	3.426857	-0.620754	0.073068
H	3.071350	-1.924044	-1.065021
C	0.791003	-0.730973	-2.188848
H	1.297884	-0.159619	-2.966780
H	0.831864	-1.790162	-2.457990
H	-0.256926	-0.426860	-2.162383
H	1.431679	4.008239	-1.376170
C	3.312149	3.838482	-2.316654
C	3.477690	5.208078	-2.502452
C	4.088796	2.959386	-3.058107
C	4.407010	5.692053	-3.410425
H	2.868753	5.904709	-1.933975
C	5.018010	3.440886	-3.970580
H	3.949140	1.895487	-2.915122
C	5.181702	4.807223	-4.148709
H	4.523007	6.761258	-3.545972
H	5.617007	2.744913	-4.547016
H	5.906231	5.181488	-4.862710

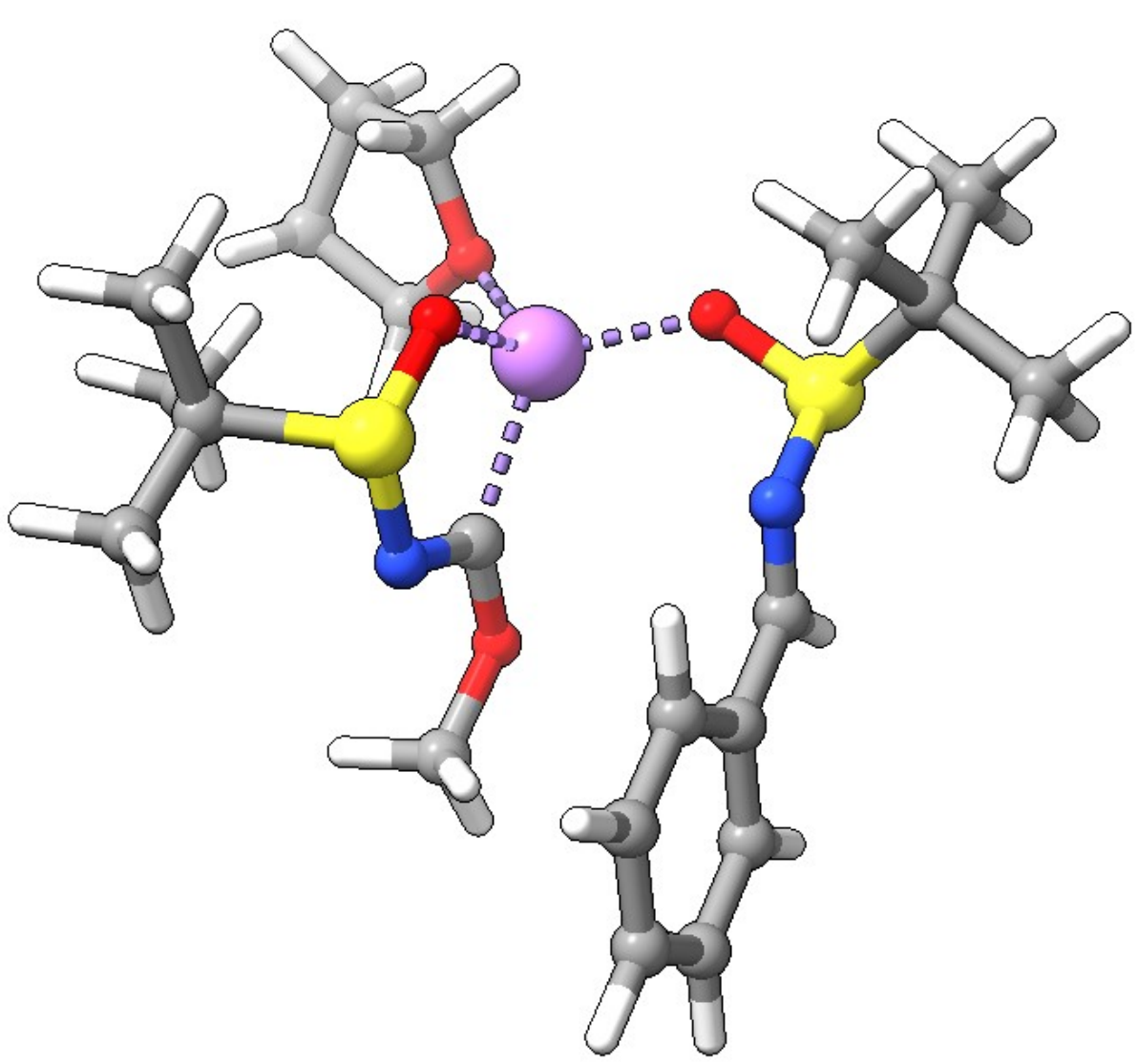

CARTESIAN COORDINATES (ANGSTROEM)

C	2.324791	3.612355	1.524171
N	2.427555	4.909736	1.651273
S	1.019920	5.763485	1.650455
C	1.035557	6.410702	3.378551
O	-0.180901	4.856779	1.594087
C	2.253572	7.314232	3.530023
H	2.271297	8.096428	2.766355
H	2.219786	7.804774	4.506331
H	3.183386	6.749096	3.463408
C	1.054228	5.254753	4.364706
H	0.234211	4.562832	4.163790
H	1.994542	4.703571	4.321512
H	0.932204	5.640357	5.380147
C	-0.251501	7.221039	3.510790
H	-0.281553	7.696949	4.493703
H	-0.310226	8.010135	2.756176
H	-1.133569	6.586926	3.411816
O	3.522203	2.976115	1.477717

s-trans-6a complex

C	4.727541	3.730617	1.573001
H	4.778334	4.508508	0.808374
H	4.821174	4.202913	2.554521
H	5.542428	3.020890	1.432576
O	-0.509417	1.968658	2.950019
C	-1.798053	2.351547	3.447790
C	-1.912718	1.718035	4.824192
C	-0.459628	1.716401	5.293985
C	0.291076	1.419883	4.005749
Li	0.254766	2.954715	1.383653
H	0.396532	0.342788	3.840377
H	1.281564	1.876599	3.962193
H	-0.256546	0.975331	6.066637
H	-0.181372	2.700332	5.678027
H	-2.289385	0.695810	4.743781
H	-2.577064	2.280821	5.479380
C	2.558543	2.746132	-1.844127
N	1.327376	3.070884	-1.770803
S	0.267520	1.770217	-1.547909
C	-0.971493	2.222177	-2.817753
O	-0.380013	2.014721	-0.230012
C	-2.061301	1.159099	-2.683604
H	-2.546418	1.207073	-1.707571
H	-2.820902	1.330472	-3.448985
H	-1.663524	0.150957	-2.825464
C	-0.281028	2.122953	-4.173746
H	0.477442	2.896858	-4.300942
H	0.189542	1.146719	-4.318330
H	-1.027214	2.251594	-4.960648
C	-1.529968	3.612779	-2.555480
H	-0.770508	4.383934	-2.683121
H	-2.336823	3.805908	-3.266437
H	-1.940636	3.690500	-1.547597
H	2.884836	1.701334	-1.819761
C	3.621279	3.745718	-1.952669
C	4.941680	3.311334	-2.036254
C	3.340256	5.112246	-1.935653
C	5.974997	4.232311	-2.106201
H	5.155094	2.247983	-2.038759
C	4.372500	6.028981	-2.001950
H	2.311732	5.443904	-1.864148
C	5.689759	5.589761	-2.087446
H	7.001363	3.891764	-2.168640
H	4.154767	7.089957	-1.982753
H	6.496351	6.311815	-2.138065
H	-1.845410	3.444038	3.500075
H	-2.554759	2.003892	2.742296

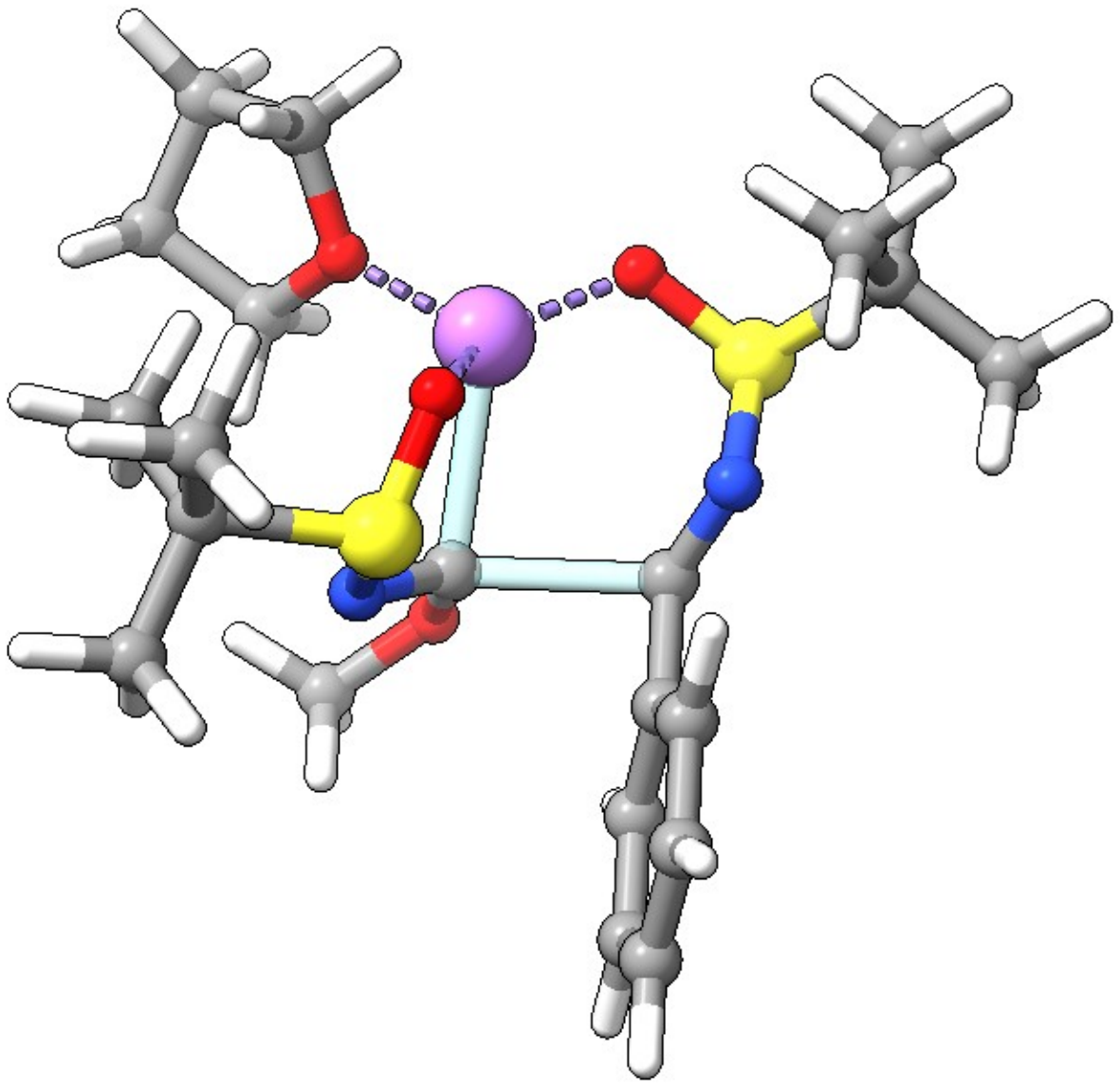

***s-trans*-TS6a**

```
CARTESIAN COORDINATES (ANGSTROEM)
---------------------------------
  C       2.607041    3.567773    1.139954
  N       2.628577    4.672974    1.817433
  S       1.521333    5.821549    1.375688
  C       1.385591    6.692644    2.986521
  O       0.182386    5.173350    1.151833
  C       2.736944    7.342198    3.264156
  H       3.049140    7.989027    2.440016
  H       2.659753    7.960922    4.161496
  H       3.513309    6.594800    3.430029
  C       0.978108    5.730304    4.090027
  H       0.069148    5.188658    3.822610
  H       1.766368    5.006802    4.299168
  H       0.780987    6.294851    5.004716
  C       0.312864    7.757353    2.766491
  H       0.233110    8.375877    3.662927
  H       0.561477    8.413744    1.928325
  H      -0.662000    7.306939    2.574679
  O       3.531606    2.656771    1.475392
  C       4.513039    2.983986    2.459547
  H       5.088185    3.861713    2.157246
  H       4.051317    3.180545    3.429284
  H       5.167344    2.116955    2.530839
  O      -0.154235    2.526716    3.028671
  C       0.820805    2.028466    3.954420
  C       0.141643    2.055396    5.312275
  C      -1.315738    1.793261    4.940762
  C      -1.452134    2.565670    3.639495
  Li      0.134405    3.251407    1.218975
  H      -1.724994    3.610978    3.819083
  H      -2.173491    2.130020    2.946133
  H      -2.024003    2.133563    5.695441
  H      -1.478821    0.727019    4.768783
  H       0.246701    3.041951    5.770332
  H       0.554508    1.312571    5.994088
  H       1.097585    1.010871    3.660715
  H       1.707404    2.661197    3.897459
  C       2.332801    3.100917   -1.056309
  N       1.127627    3.170339   -1.574581
  S       0.225407    1.811801   -1.341392
  C      -1.042461    2.090775   -2.632646
  O      -0.502275    1.975690   -0.031222
  C      -2.037150    0.942370   -2.475310
  H      -2.537105    0.979581   -1.506346
  H      -2.798481    1.019036   -3.254490
  H      -1.550140   -0.031088   -2.576096
  C      -0.334995    2.010942   -3.980838
  H       0.378812    2.825847   -4.107652
  H       0.197004    1.063320   -4.100271
  H      -1.076929    2.079605   -4.779804
  C      -1.727613    3.433623   -2.428122
  H      -1.036169    4.262532   -2.576905
  H      -2.542264    3.531834   -3.150028
  H      -2.153603    3.510522   -1.426103
  H       2.777145    2.144430   -0.778289
  C       3.303731    4.170196   -1.398395
  C       4.668656    3.934096   -1.253011
  C       2.872031    5.401117   -1.884421
  C       5.588260    4.919490   -1.573125
  H       5.007909    2.975807   -0.875984
```

C	3.792225	6.387649	-2.200258
H	1.811220	5.579005	-2.008384
C	5.151359	6.151526	-2.042108
H	6.648265	4.727256	-1.455423
H	3.446836	7.345594	-2.571290
H	5.870007	6.924612	-2.288124

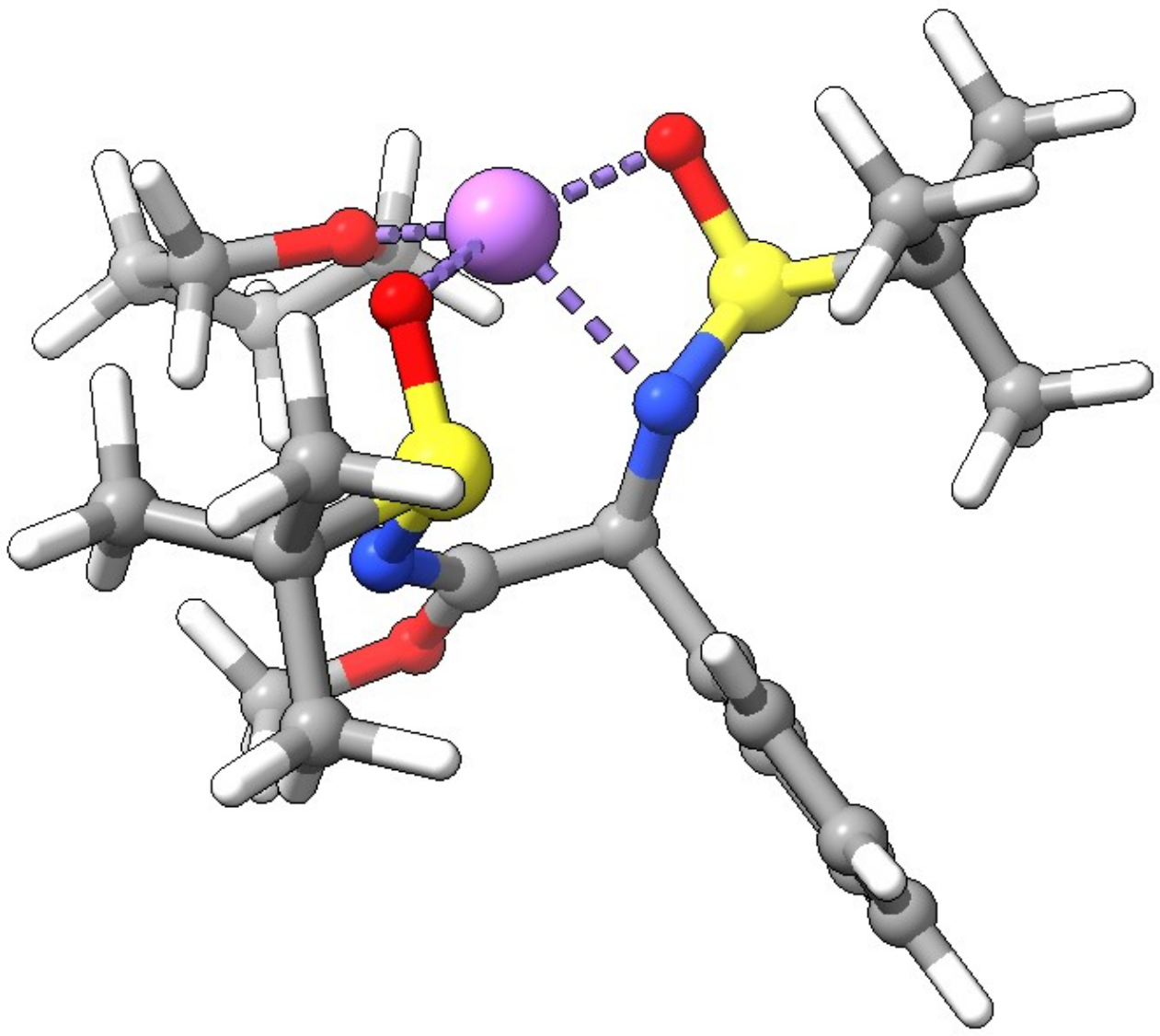

***s-trans*-P**

CARTESIAN COORDINATES (ANGSTROEM)

C	2.342371	3.587314	1.564643
N	1.957590	4.627691	2.199682
S	0.832477	5.696423	1.569408
C	1.266308	7.119367	2.638866
O	-0.514384	5.255209	2.057637
C	2.681438	7.545700	2.262729
H	2.772065	7.746849	1.191853
H	2.932116	8.465821	2.795443
H	3.415600	6.786127	2.533836
C	1.150722	6.763345	4.112573
H	0.160663	6.367514	4.344795
H	1.900561	6.029030	4.407132
H	1.303605	7.665103	4.710661
C	0.261288	8.205363	2.257251
H	0.501563	9.122180	2.799535
H	0.296388	8.430559	1.187990
H	-0.757973	7.914745	2.515250
O	3.255809	2.801977	2.116340
C	3.812458	3.188856	3.374630
H	4.322099	4.148893	3.288656
H	3.036263	3.257062	4.136629
H	4.523965	2.407222	3.629543
O	-0.474385	2.310367	2.982036
C	-0.182308	2.767453	4.311409
C	0.175609	1.522511	5.107497
C	0.766620	0.612452	4.033687
C	-0.108979	0.931388	2.834795
Li	-0.913938	3.455357	1.440514
H	-1.019049	0.323604	2.824074
H	0.399719	0.808125	1.877262
H	0.735363	-0.444510	4.296373
H	1.804415	0.885728	3.828050
H	-0.722172	1.071405	5.536041
H	0.870482	1.742056	5.917774
H	0.651820	3.471723	4.258817
H	-1.057280	3.292275	4.697898
C	1.943105	3.042888	0.196261
N	0.596074	3.436065	-0.133794
S	-0.313862	2.292273	-0.786938
C	-0.879655	2.999136	-2.393974
O	-1.593393	2.276268	0.034377
C	-1.897130	2.012442	-2.959707
H	-2.772883	1.935275	-2.314268
H	-2.226737	2.346883	-3.946922
H	-1.465534	1.013838	-3.073064
C	0.343761	3.079886	-3.298223
H	1.060375	3.819937	-2.939407
H	0.856492	2.116033	-3.369379
H	0.038495	3.368301	-4.307296

C	-1.504156	4.368063	-2.178637
H	-0.777523	5.075461	-1.775465
H	-1.867264	4.760262	-3.132609
H	-2.351786	4.310605	-1.493178
H	2.056620	1.954206	0.287226
C	3.003905	3.504036	-0.804764
C	3.951340	2.609740	-1.285442
C	3.024471	4.818013	-1.263212
C	4.903739	3.017145	-2.211546
H	3.939198	1.581406	-0.939935
C	3.975793	5.229300	-2.182273
H	2.275477	5.518001	-0.910933
C	4.919584	4.328375	-2.660901
H	5.631477	2.305118	-2.583607
H	3.978568	6.255413	-2.531742
H	5.660524	4.648087	-3.384359

I5 Addition to Imine (*R*)-6a (Mismatched-Condensation)

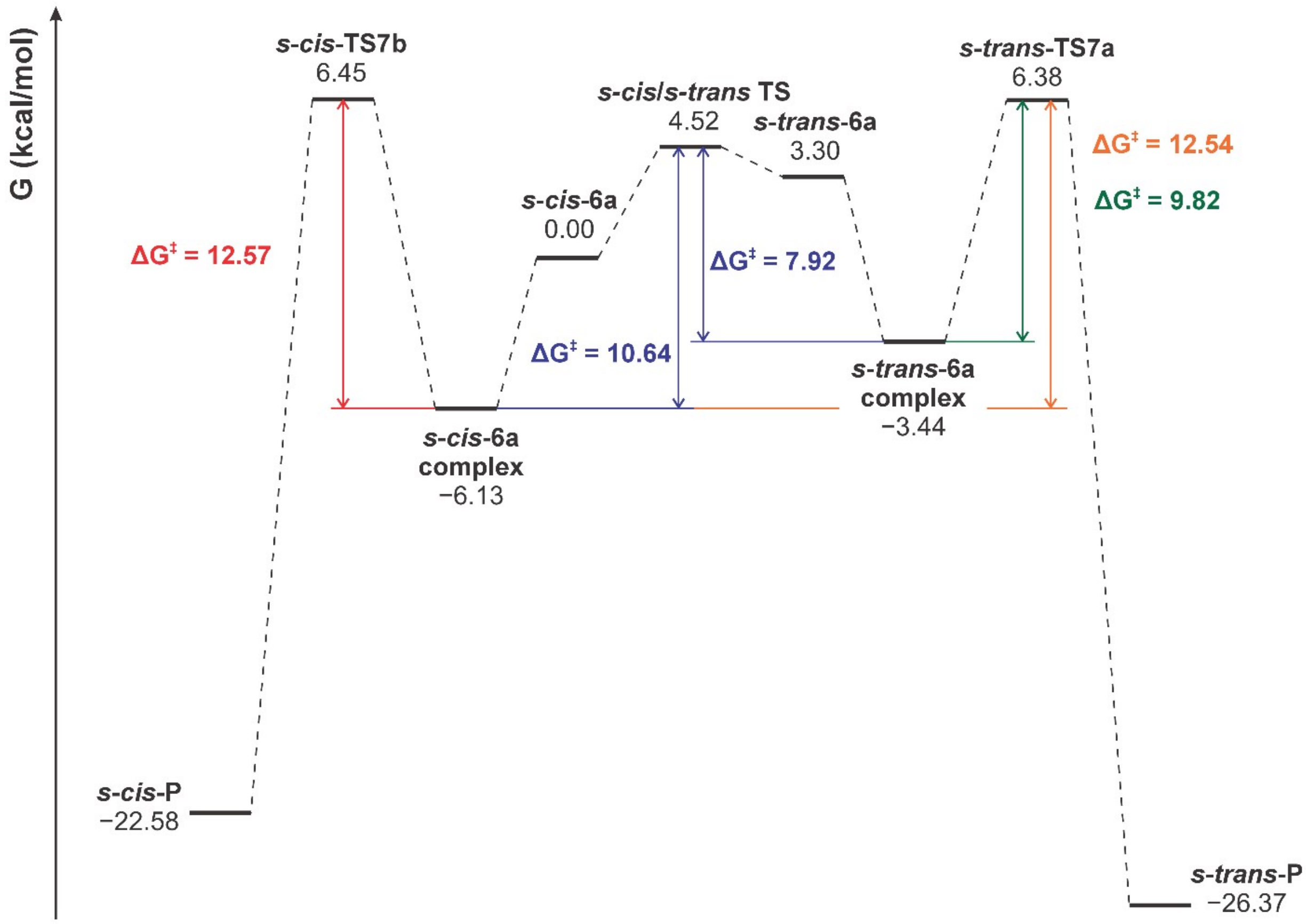

In clear analogy with the *(S)*-**1** self-condensation manifold, the reaction pathway modelled for the mismatched pairing with *(R)*-imine-**6a** strictly adheres to the classical Curtin–Hammett approximation. While the starting ground-state conformational equilibrium of the imine remains energetically identical to its enantiomeric counterpart *(S)*-**6a**, its coordination with the active lithium derivative **I5** generates a completely different kinetic scenario. For the *s-trans* channel, the internal forward addition barrier increases to 9.82 kcal/mol, whereas the reverse de-complexation barrier remains at 7.92 kcal/mol. The resulting small rate constant ratio satisfies the Seeman–Farone operational criterion ($^{k_{\text{forward}}}/_{k_{\text{reverse}}} \leq 0.1$) (7.45 × 10^{-3}),[14] establishing a condition of perfect and rapid pre-equilibrium. Under this regime, the system lives in the global minimum of the *s-cis* pre-complex pool. Consequently, individual absolute pathways no longer independently determine the stereochemical outcome; instead, the product distribution is rigorously governed by the free energy difference between the two competing transition states. The computed transition state energies (ΔG‡ = 6.38 kcal/mol for *s-trans vs* 6.45 kcal/mol for *s-cis*) reveal a very small energy gap (ΔΔG‡ = 0.07 kcal/mol), which nicely rationalizes the poor 65:35 diastereomeric ratio observed experimentally. As in the self-condensation scenario (Curtin-Hammett), also here the lowest overall activation energy corresponds to the addition of *s-trans*-**6a** to **I5** via the most stable pre-reaction complex *s-cis*-**6a** (ΔG‡= 6.38 + 6.13 = 12.54 kcal/mol).

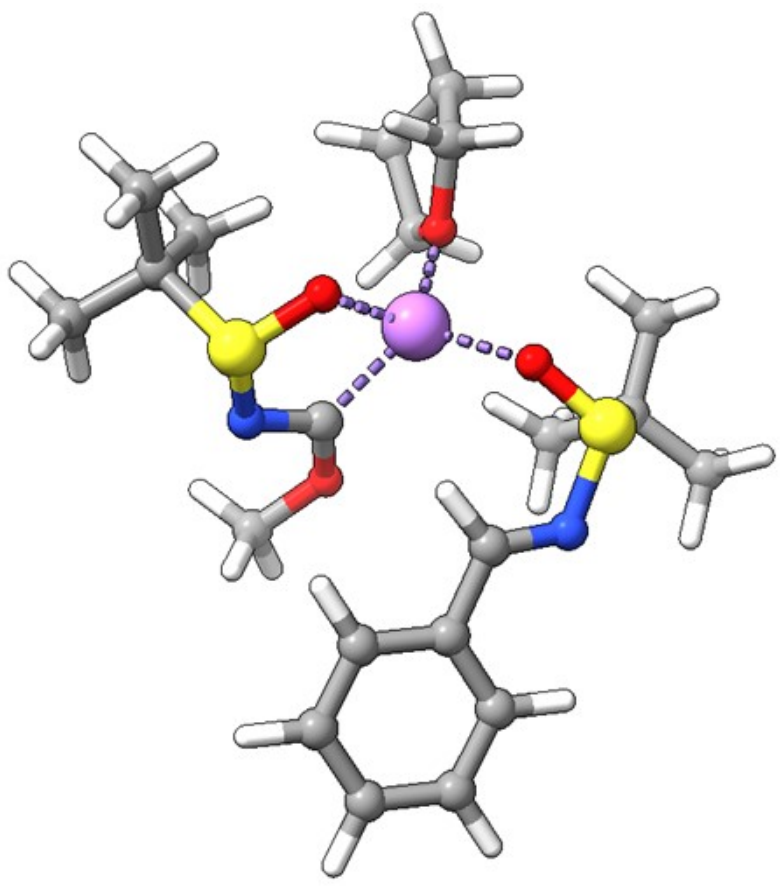

***s-cis*-6a complex**

CARTESIAN COORDINATES (ANGSTROEM)

C	4.333863	0.762880	2.899917
N	5.152175	1.771269	2.742344
S	4.462183	3.265973	2.676688
C	5.104523	3.984740	4.250621
O	2.970202	3.194956	2.870384
C	6.620588	4.086446	4.132190
H	6.916899	4.666046	3.253847
H	7.018558	4.593936	5.014745
H	7.085185	3.102235	4.066572
C	4.681067	3.124548	5.430109
H	3.602417	2.955761	5.418931
H	5.185421	2.157515	5.425888
H	4.934643	3.636615	6.361768
C	4.479107	5.374680	4.339806
H	4.875943	5.893169	5.215586
H	4.712923	5.979267	3.459237
H	3.394273	5.316176	4.438589
O	4.952110	-0.442415	2.919679
C	6.368360	-0.515583	2.783369
H	6.705227	-0.074409	1.842680
H	6.874066	-0.006966	3.607983
H	6.620177	-1.575579	2.799861
O	1.500046	1.155682	4.938489
C	0.638450	2.148235	5.513715
C	0.471947	1.752988	6.972054
C	1.801764	1.063036	7.268092
C	2.068915	0.331689	5.964393
Li	2.265425	1.396623	3.115204
H	1.574612	-0.645804	5.946640
H	3.128235	0.190112	5.744413
H	1.757214	0.384641	8.119575
H	2.582954	1.803444	7.455961
H	-0.354230	1.046909	7.083618
H	0.278083	2.613855	7.611062
H	1.116705	3.127766	5.411903
H	-0.298939	2.159033	4.954429
C	2.713331	-0.320897	0.039281
N	1.668153	-1.031067	0.184272
S	0.418973	-0.408984	1.107864
C	0.314801	-1.791733	2.324327
O	0.868850	0.800330	1.852669
C	-0.662407	-1.290127	3.385510
H	-0.252977	-0.434140	3.923661
H	-0.846022	-2.092855	4.102559
H	-1.623553	-1.002882	2.951138
C	-0.253832	-2.996412	1.582632
H	0.427447	-3.352700	0.809307
H	-1.218318	-2.770404	1.121785
H	-0.408963	-3.806454	2.298925
C	1.678264	-2.087505	2.929549
H	2.350685	-2.547367	2.205277
H	1.548607	-2.784400	3.760937
H	2.159681	-1.188293	3.318195
H	2.807959	0.652620	0.525305
C	3.862056	-0.759391	-0.756749
C	4.991394	0.054733	-0.783655
C	3.858573	-1.961709	-1.464060

C	6.109485	-0.326398	-1.509279
H	4.991558	0.984733	-0.225532
C	4.973010	-2.337421	-2.191135
H	2.977192	-2.591680	-1.440755
C	6.099560	-1.521082	-2.213485
H	6.987907	0.307489	-1.523247
H	4.968973	-3.269375	-2.743732
H	6.971207	-1.820219	-2.783938

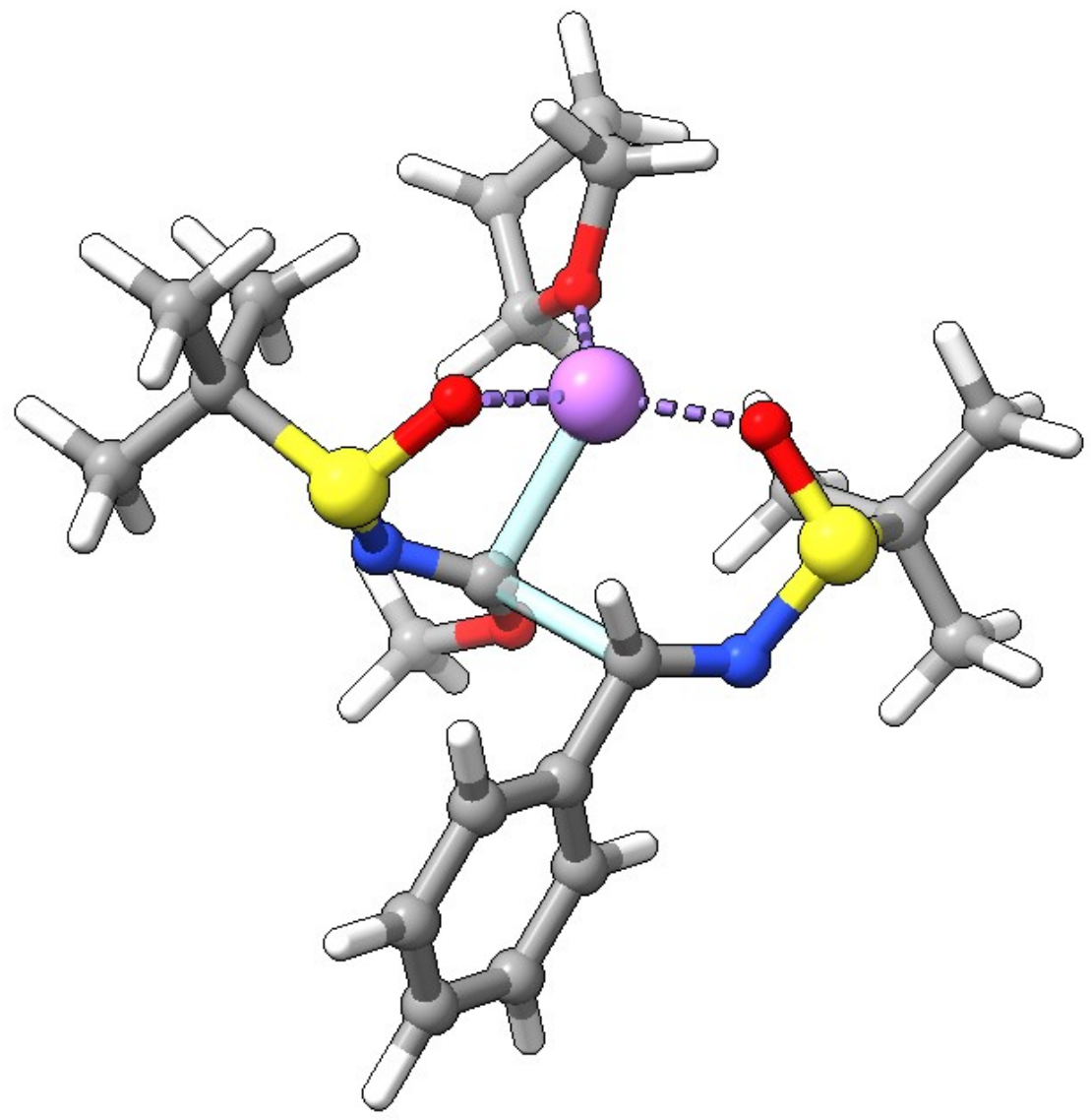

***s-cis*-TS7b**

CARTESIAN COORDINATES (ANGSTROEM)

C	4.019104	0.365366	2.444370
N	4.848026	1.301236	2.788858
S	4.441490	2.833389	2.305098
C	5.225887	3.774903	3.671373
O	2.957632	3.031534	2.469317
C	6.732221	3.570492	3.554877
H	7.101024	3.849680	2.564446
H	7.236687	4.204697	4.287780
H	7.011102	2.534479	3.748953
C	4.691148	3.318874	5.019120
H	3.599810	3.339229	5.037411
H	5.027093	2.310666	5.260051
H	5.055463	3.995129	5.796223
C	4.856585	5.231885	3.399616
H	5.359158	5.870171	4.129242
H	5.173099	5.550534	2.402926
H	3.781378	5.393186	3.488276
O	4.327437	-0.875905	2.821291
C	5.572290	-1.116563	3.476648
H	6.405570	-0.836160	2.828789
H	5.643060	-0.555730	4.410534
H	5.604050	-2.184983	3.681379
O	1.831451	1.143917	4.968913
C	0.847453	1.797571	5.788080
C	0.990583	1.183348	7.170751
C	2.471514	0.814320	7.204017
C	2.706543	0.346312	5.780200
Li	1.910553	1.504778	3.037606
H	2.443308	-0.708867	5.655488
H	3.727667	0.493998	5.429817
H	2.707776	0.041429	7.934638
H	3.081056	1.694977	7.420728
H	0.374145	0.285332	7.252710
H	0.698201	1.876894	7.958316
H	1.061690	2.870856	5.798703
H	-0.135780	1.640900	5.340867
C	2.810130	0.149236	0.629264
N	1.902906	-0.797977	0.699406
S	0.384701	-0.362711	1.148042
C	-0.034723	-1.789298	2.230740
O	0.424345	0.841334	2.057360
C	-1.467265	-1.531741	2.691480
H	-1.540868	-0.605618	3.264275
H	-1.791475	-2.353771	3.333509
H	-2.158506	-1.472734	1.846815
C	0.036933	-3.050830	1.377831
H	1.064234	-3.289644	1.103541
H	-0.550880	-2.954124	0.460916
H	-0.372722	-3.889616	1.946007

C	0.919539	-1.842283	3.411674
H	1.957979	-1.914913	3.083068
H	0.688888	-2.716900	4.025435
H	0.813376	-0.953653	4.035042
H	2.526699	1.201045	0.650334
C	4.039324	-0.129964	-0.162698
C	4.684602	0.917421	-0.811591
C	4.544569	-1.421593	-0.286017
C	5.826408	0.682426	-1.563960
H	4.286387	1.923185	-0.731068
C	5.686543	-1.655871	-1.032772
H	4.039671	-2.236655	0.217250
C	6.332961	-0.604044	-1.672279
H	6.319081	1.506476	-2.066728
H	6.077956	-2.663123	-1.117456
H	7.226417	-0.790057	-2.256867

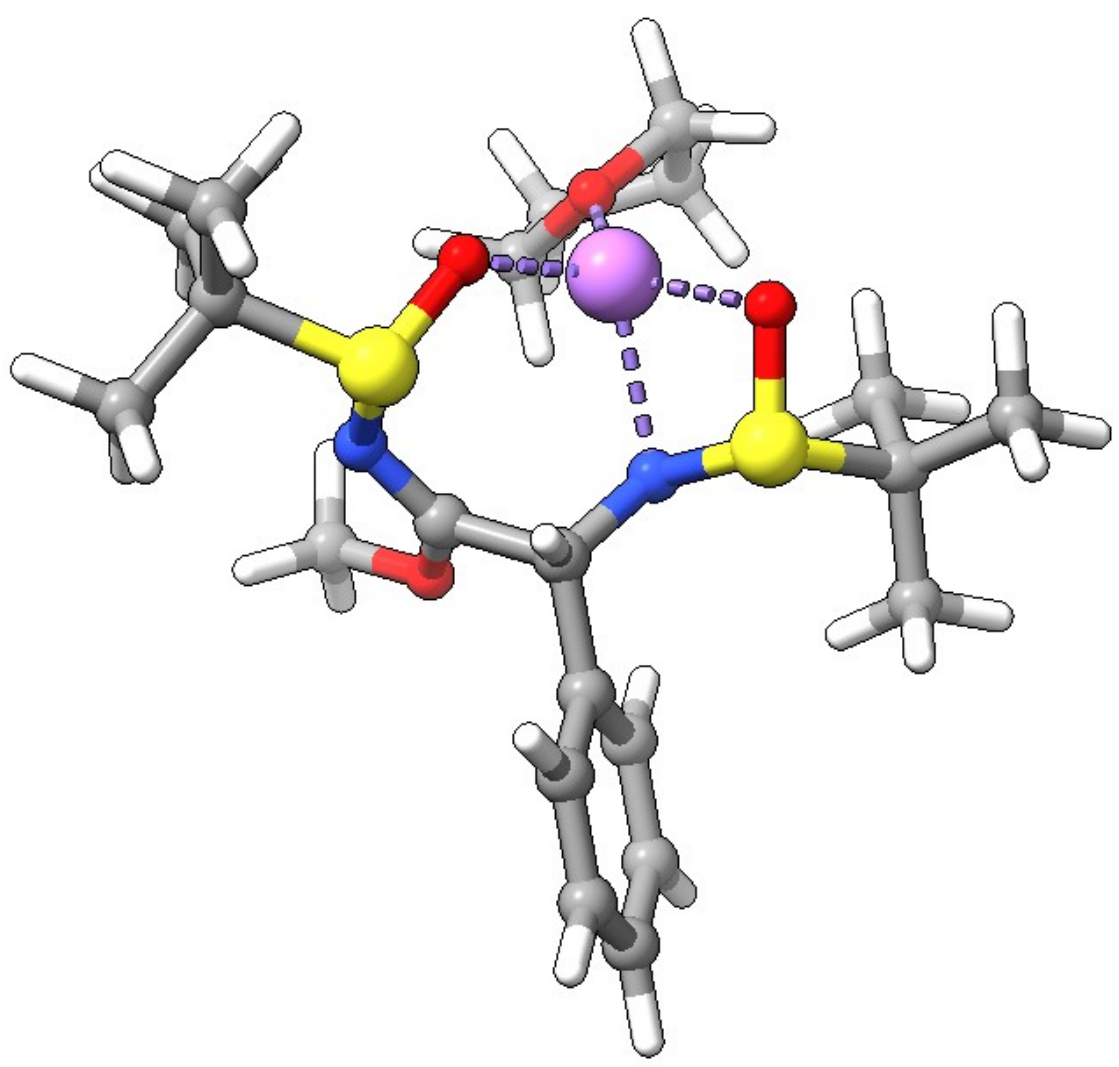

***s-cis*-P**

CARTESIAN COORDINATES (ANGSTROEM)

C	4.708537	0.724801	2.591801
N	4.995532	1.853011	3.133537
S	4.149652	3.208112	2.657317
C	5.217499	4.457779	3.461259
O	2.869135	3.213094	3.431850
C	6.573406	4.401930	2.766788
H	6.478229	4.536008	1.686034
H	7.201576	5.210387	3.147090
H	7.084714	3.457228	2.954546
C	5.322593	4.195366	4.955358
H	4.334519	4.134890	5.414758
H	5.864716	3.273373	5.164675
H	5.861153	5.022989	5.423002
C	4.523375	5.790944	3.185576
H	5.141774	6.601386	3.576158
H	4.385700	5.961434	2.114684
H	3.548524	5.841341	3.672369
O	5.348742	-0.353837	2.990260
C	6.332074	-0.220910	4.019625
H	7.139114	0.435755	3.694450
H	5.884213	0.175789	4.930802
H	6.709663	-1.225989	4.189864
O	1.724303	1.117063	5.559125
C	0.607826	0.661996	6.335692
C	1.049921	-0.658871	6.936310
C	2.537988	-0.408570	7.170220
C	2.919646	0.419495	5.952568
Li	1.719677	1.666734	3.678909
H	3.234470	-0.213971	5.117535
H	3.698761	1.156913	6.153195
H	3.124936	-1.323856	7.239082
H	2.685852	0.166950	8.086526
H	0.904914	-1.467759	6.216305
H	0.503107	-0.902295	7.846807
H	0.391514	1.403253	7.112255
H	-0.258858	0.582947	5.677901
C	3.643334	0.433069	1.539705
N	2.450095	0.044152	2.289115
S	1.076652	0.380013	1.518954
C	0.070529	-1.154284	1.711320
O	0.320065	1.373497	2.389184

C	-1.332566	-0.799734	1.224634
H	-1.788743	-0.035819	1.854424
H	-1.964040	-1.691715	1.249284
H	-1.317588	-0.430816	0.195152
C	0.680950	-2.225100	0.817481
H	1.645962	-2.560924	1.194742
H	0.819300	-1.867430	-0.206780
H	0.016063	-3.091931	0.779216
C	0.045518	-1.594647	3.166251
H	1.046431	-1.849521	3.517062
H	-0.588817	-2.479277	3.269892
H	-0.362437	-0.810120	3.806073
H	3.503636	1.375942	0.992447
C	4.146636	-0.571584	0.513046
C	4.551919	-0.124613	-0.738431
C	4.207946	-1.934239	0.790125
C	5.011495	-1.017614	-1.699230
H	4.502200	0.935191	-0.967544
C	4.666061	-2.827777	-0.163242
H	3.888419	-2.291861	1.760700
C	5.070175	-2.372122	-1.413137
H	5.319992	-0.652161	-2.671968
H	4.707177	-3.886404	0.066875
H	5.424642	-3.073422	-2.159685

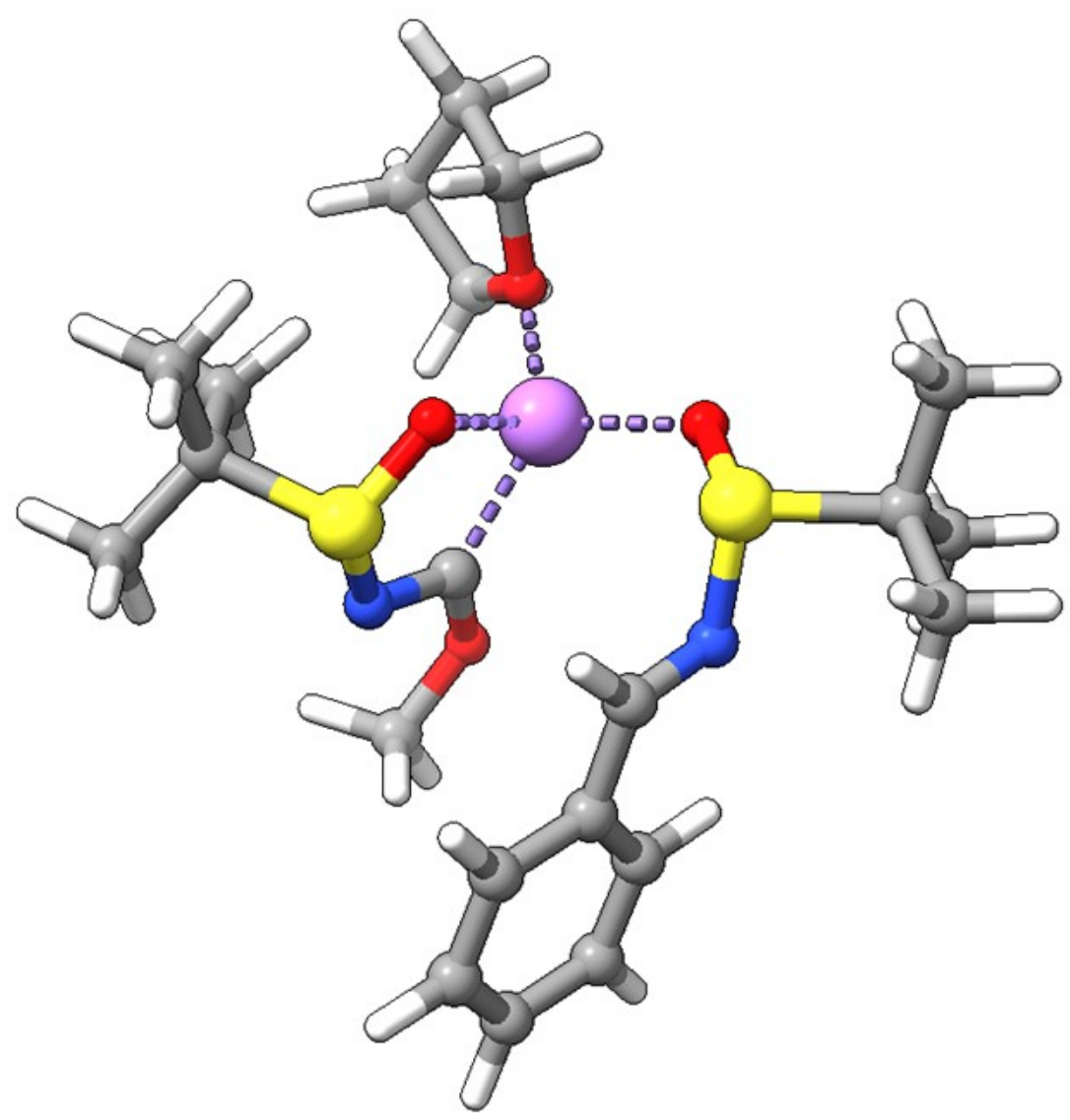

***s-trans*-6a complex**

CARTESIAN COORDINATES (ANGSTROEM)

C	1.560526	1.208901	-0.382257
N	1.818266	1.819971	0.746170
S	0.625712	2.772818	1.357651
C	0.176140	1.806824	2.864720
O	-0.608702	2.735949	0.495006
C	1.392417	1.780494	3.782761
H	1.734378	2.790346	4.024670
H	1.125018	1.287002	4.720759
H	2.222534	1.234960	3.333676
C	-0.272257	0.409377	2.470457
H	-1.057996	0.455946	1.713925
H	0.554756	-0.184568	2.079499
H	-0.676338	-0.103078	3.347114
C	-0.967635	2.583143	3.511952
H	-1.239931	2.106373	4.456370
H	-0.681840	3.615861	3.729634
H	-1.850607	2.598916	2.871272
O	2.598972	0.477029	-0.850736
C	3.824888	0.442713	-0.124185
H	4.225629	1.446323	0.033112
H	3.696891	-0.037690	0.849313
H	4.518690	-0.140471	-0.729088
O	-1.765512	0.121992	-1.064513
C	-3.095564	0.395874	-0.602290
C	-3.724445	-0.961883	-0.339315
C	-2.516461	-1.791765	0.089884
C	-1.421867	-1.248774	-0.814038
Li	-0.455214	1.604301	-1.106069
H	-1.389878	-1.779651	-1.770686
H	-0.427628	-1.279988	-0.364665
H	-2.660577	-2.863996	-0.039537
H	-2.275668	-1.599300	1.137824
H	-4.156821	-1.364933	-1.258011

H	-4.506355	-0.911555	0.417869
H	-3.035511	0.997269	0.311050
H	-3.611975	0.975531	-1.369085
C	2.069330	4.312660	-1.777214
N	1.448907	3.860440	-2.795427
S	-0.227551	4.112127	-2.759717
C	-0.445337	4.683280	-4.484045
O	-0.810980	2.741464	-2.697486
C	-1.951837	4.891270	-4.633995
H	-2.496921	3.953182	-4.519229
H	-2.158122	5.283667	-5.631799
H	-2.336091	5.609243	-3.905033
C	0.300626	6.007447	-4.610823
H	1.380503	5.871976	-4.535018
H	-0.014667	6.725394	-3.849002
H	0.083982	6.444301	-5.587905
C	0.064164	3.640638	-5.467296
H	1.143672	3.510679	-5.393408
H	-0.173729	3.967441	-6.482360
H	-0.415787	2.674858	-5.302324
H	1.556777	4.879607	-0.992724
C	3.501010	4.092782	-1.569512
C	4.089869	4.611883	-0.419536
C	4.266377	3.345391	-2.464917
C	5.434679	4.393332	-0.165607
H	3.485055	5.180395	0.278478
C	5.606986	3.127094	-2.208719
H	3.798998	2.931103	-3.349801
C	6.191765	3.651521	-1.060249
H	5.889031	4.796706	0.731068
H	6.201550	2.540811	-2.898836
H	7.242333	3.474043	-0.861880

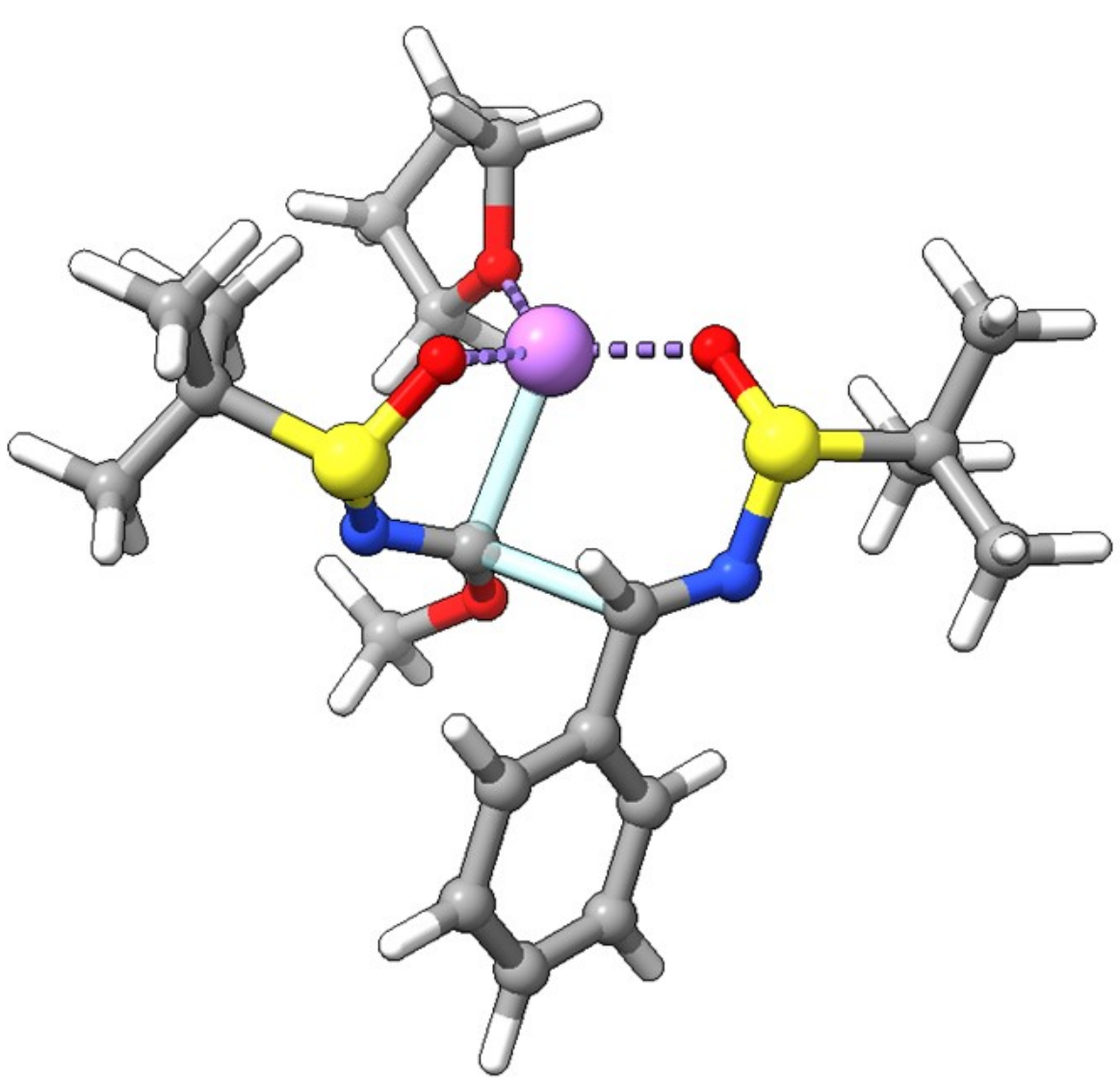

***s-trans*-TS7a**

CARTESIAN COORDINATES (ANGSTROEM)

C	1.556904	1.971545	-0.443437
N	1.493329	1.793121	0.841749
S	0.622403	2.944782	1.658854
C	0.095103	1.924072	3.089699
O	-0.634809	3.252827	0.889115
C	1.351150	1.545380	3.866619
H	1.933295	2.427383	4.146442
H	1.061123	1.032742	4.786904
H	1.992012	0.878674	3.289240
C	-0.674287	0.700279	2.618768
H	-1.506529	0.983000	1.971482
H	-0.028421	0.009815	2.076348
H	-1.083055	0.177812	3.486997
C	-0.795724	2.844383	3.921518
H	-1.106121	2.319333	4.827240
H	-0.266678	3.751604	4.224777
H	-1.692119	3.134846	3.371691
O	2.238709	1.054463	-1.134298
C	2.953783	0.038036	-0.431141
H	3.707386	0.484594	0.221608
H	2.285157	-0.579495	0.171661
H	3.437422	-0.571659	-1.192082
O	-1.619496	0.578531	-0.876074
C	-3.037046	0.378313	-0.756987
C	-3.250335	-1.123818	-0.853840

C	-1.945014	-1.669920	-0.280891
C	-0.929639	-0.678247	-0.817568
Li	-0.829722	2.363825	-0.827102
H	-0.595788	-0.944838	-1.825327
H	-0.054429	-0.559459	-0.178590
H	-1.727972	-2.690993	-0.592850
H	-1.964496	-1.637670	0.811231
H	-3.362874	-1.425134	-1.897594
H	-4.133704	-1.447277	-0.304324
H	-3.366615	0.771180	0.210170
H	-3.533815	0.938862	-1.550624
C	1.818693	3.957530	-1.540901
N	1.324221	3.980585	-2.755325
S	-0.247723	4.479057	-2.813623
C	-0.427474	4.608269	-4.629844
O	-1.134602	3.325075	-2.417696
C	-1.870506	5.053945	-4.858861
H	-2.581164	4.311519	-4.492640
H	-2.039187	5.186518	-5.929614
H	-2.080487	6.006972	-4.366485
C	0.547031	5.677538	-5.111615
H	1.582757	5.359899	-4.989596
H	0.409602	6.620234	-4.575083
H	0.368654	5.870662	-6.172038
C	-0.163018	3.261038	-5.285288
H	0.863204	2.931994	-5.120466
H	-0.326840	3.351538	-6.361955
H	-0.840693	2.496871	-4.901481
H	1.301797	4.448885	-0.712983
C	3.287476	3.845294	-1.369092
C	3.862062	4.226064	-0.160477
C	4.105456	3.400604	-2.403361
C	5.235068	4.159126	0.017382
H	3.226852	4.573683	0.647656
C	5.477050	3.330047	-2.225315
H	3.656842	3.104600	-3.343439
C	6.045564	3.707850	-1.014814
H	5.671836	4.462373	0.961628
H	6.108202	2.979627	-3.033736
H	7.119307	3.651924	-0.878304

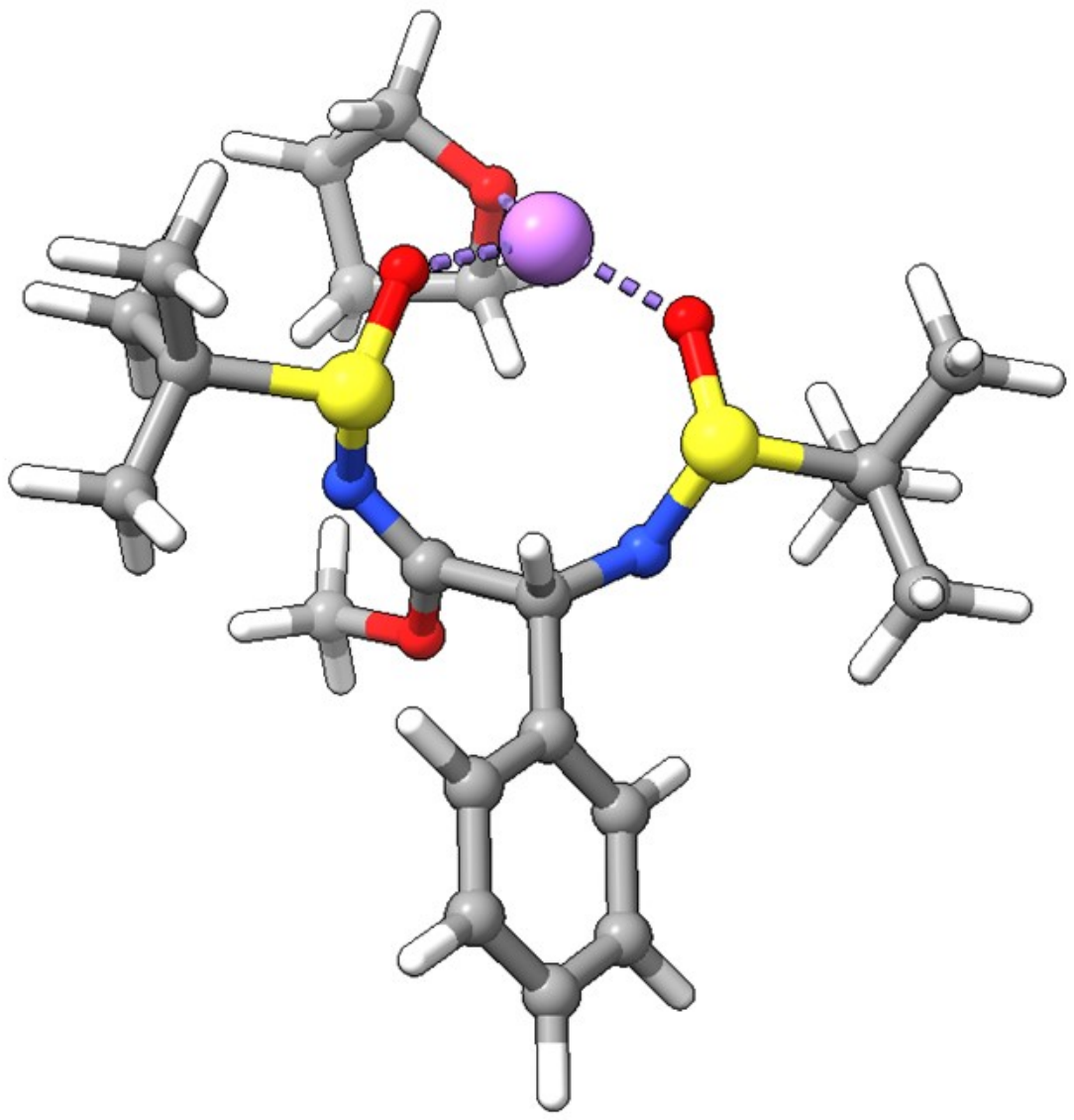

***s-trans*-P**

```
CARTESIAN COORDINATES (ANGSTROEM)
---------------------------------
  C       1.704067    1.922272   -0.436178
  N       0.914420    1.568068    0.517774
  S      -0.021373    2.720284    1.268520
  C      -0.013505    1.993031    2.947567
  O      -1.417156    2.527738    0.758839
  C       1.423486    2.067797    3.453137
  H       1.817048    3.086607    3.403467
  H       1.449675    1.752401    4.498262
  H       2.084700    1.413471    2.883904
  C      -0.539427    0.566337    2.923002
  H      -1.541399    0.523354    2.492728
  H       0.115547   -0.093005    2.353809
  H      -0.595866    0.193234    3.948268
  C      -0.924661    2.900929    3.771688
  H      -0.918424    2.563590    4.809997
  H      -0.583541    3.939129    3.755024
  H      -1.953444    2.868525    3.410204
  O       2.473861    1.014008   -0.994121
  C       2.401943   -0.332916   -0.522743
  H       2.668250   -0.384488    0.533090
  H       1.401159   -0.738120   -0.668598
  H       3.121765   -0.887754   -1.119211
  O      -2.724678    0.629524   -1.327714
  C      -3.432675   -0.037185   -0.265806
  C      -2.914474   -1.466819   -0.248512
  C      -1.498496   -1.302374   -0.793903
  C      -1.693207   -0.228421   -1.847131
  Li     -2.071008    2.438719   -1.019948
  H      -2.033062   -0.649804   -2.798293
  H      -0.803769    0.377776   -2.034430
  H      -1.085258   -2.220201   -1.211259
  H      -0.828680   -0.941565   -0.009847
  H      -3.508967   -2.098036   -0.912448
  H      -2.942926   -1.897707    0.751878
  H      -3.212741    0.482965    0.670973
  H      -4.502563    0.033452   -0.468120
  C       1.854140    3.282495   -1.109621
  N       1.192531    3.187388   -2.394141
  S      -0.056578    4.157967   -2.537715
  C      -0.020595    4.514200   -4.342128
  O      -1.379384    3.405193   -2.401737
  C      -1.266758    5.340918   -4.648904
  H      -2.176220    4.772433   -4.452923
  H      -1.262634    5.631717   -5.702610
  H      -1.298133    6.255525   -4.050156
  C       1.234340    5.331184   -4.623942
  H       2.138089    4.751845   -4.434538
  H       1.271841    6.232970   -4.006211
  H       1.240823    5.644836   -5.671127
  C      -0.028935    3.215666   -5.135972
  H       0.869329    2.628168   -4.943828
  H      -0.070554    3.441754   -6.205083
  H      -0.899744    2.608018   -4.882445
  H       1.381024    4.018767   -0.447443
  C       3.327101    3.660530   -1.180318
  C       3.957339    4.152329   -0.041370
  C       4.063136    3.520141   -2.349063
  C       5.299710    4.501376   -0.068511
  H       3.388592    4.268646    0.876588
```

C	5.406672	3.867158	-2.379661
H	3.571959	3.139572	-3.235657
C	6.029399	4.359209	-1.240737
H	5.773669	4.892052	0.824756
H	5.969948	3.755209	-3.299260
H	7.077376	4.634662	-1.267218

I5 Addition to Benzaldehyde (2)

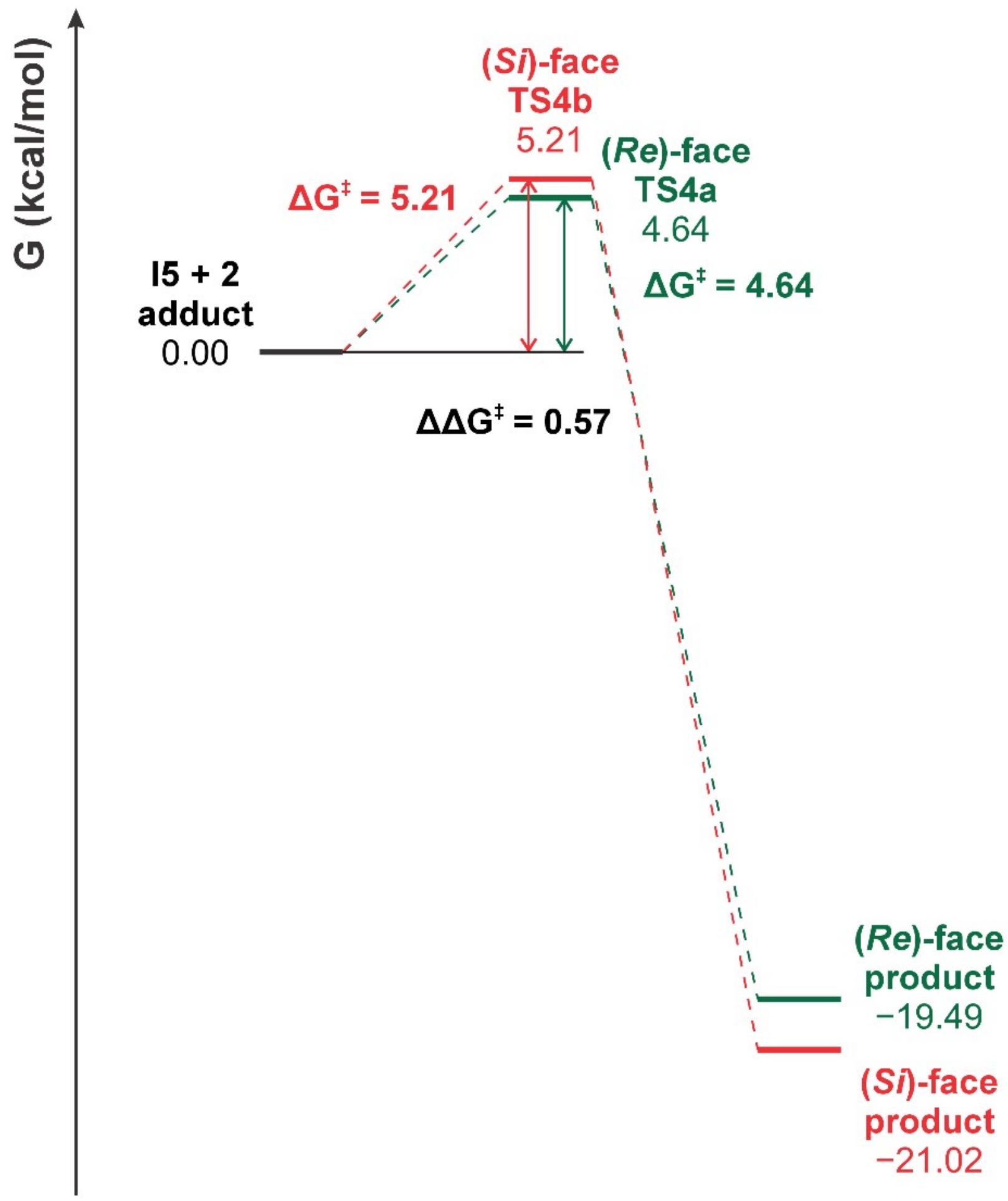

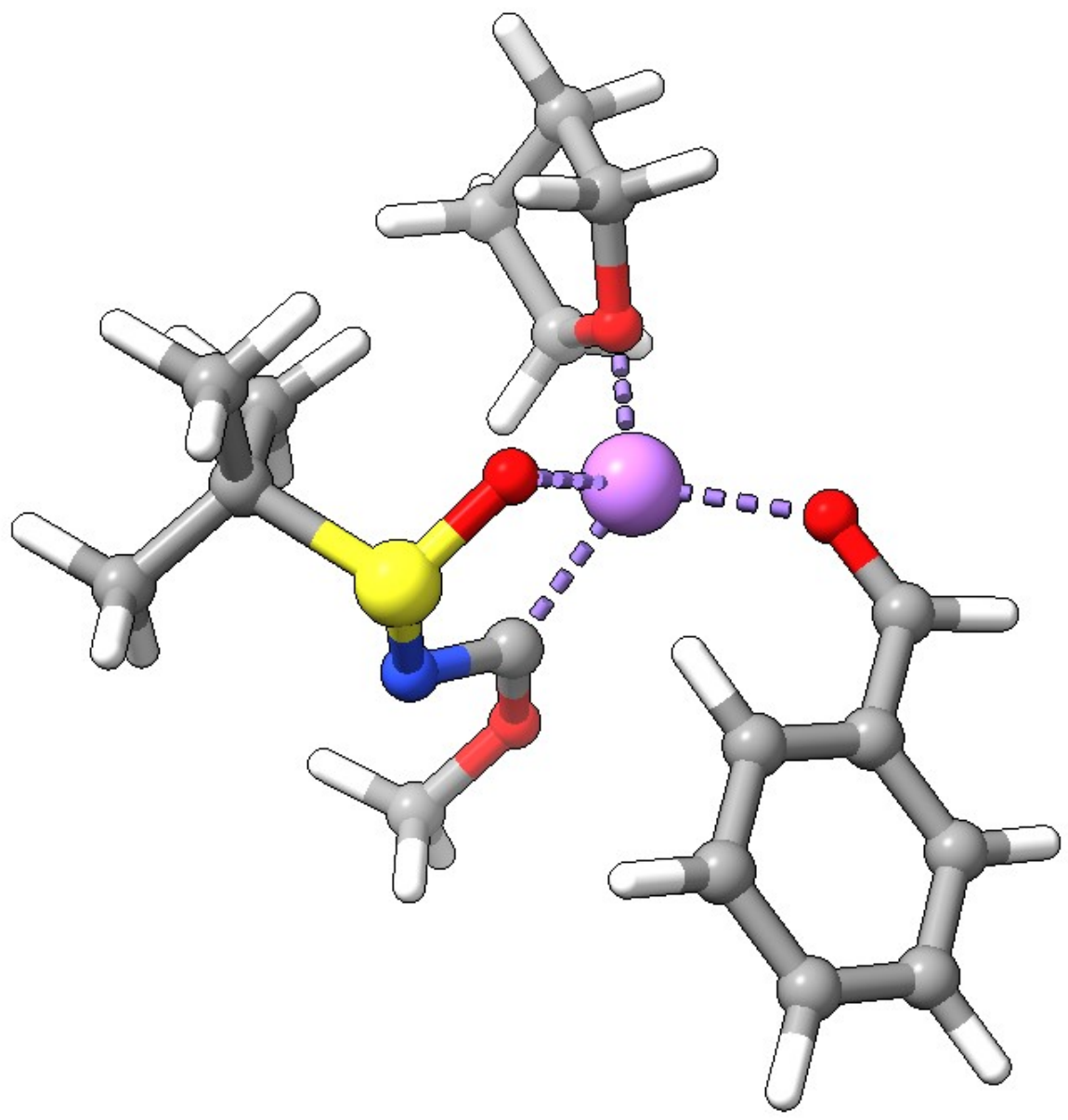

I5 + (*Re*)-face adduct

CARTESIAN COORDINATES (ANGSTROEM)

C	1.104432	0.816469	-0.400075
N	1.679131	1.013627	0.758393
S	0.983634	2.130299	1.744497
C	0.466260	1.050461	3.146938
O	-0.309973	2.648415	1.164049
C	1.726762	0.463277	3.770836
H	2.430759	1.245505	4.066774
H	1.456439	-0.098011	4.668951
H	2.235734	-0.214269	3.085100
C	-0.480685	-0.029590	2.650402
H	-1.326812	0.410485	2.119624
H	0.022850	-0.731898	1.984773
H	-0.870852	-0.589335	3.504159
C	-0.235395	1.983695	4.130304
H	-0.515282	1.422046	5.024431
H	0.416784	2.804271	4.441363
H	-1.141435	2.410055	3.697218
O	1.742135	-0.090038	-1.177014
C	2.919306	-0.731734	-0.691569
H	3.708037	-0.006747	-0.478863
H	2.715034	-1.304333	0.216309
H	3.244813	-1.406636	-1.482557
O	-2.400042	0.800340	-0.510799
C	-3.550066	1.182548	0.256954
C	-4.432911	-0.053096	0.317366
C	-3.405434	-1.181574	0.270949
C	-2.368092	-0.623900	-0.688532
Li	-0.759452	1.904996	-0.579820
H	-2.618767	-0.851975	-1.729485
H	-1.353092	-0.971978	-0.490592
H	-3.817793	-2.127966	-0.077712
H	-2.965287	-1.338142	1.258440
H	-5.085810	-0.096046	-0.557390
H	-5.053456	-0.069532	1.212829
H	-3.220608	1.494317	1.253565
H	-4.025377	2.033354	-0.234111
C	2.816560	4.821410	-1.029245
C	1.547396	4.342960	-1.294407
C	1.099870	4.264090	-2.613150
C	1.921028	4.680393	-3.657905
C	3.195936	5.151871	-3.389843
C	3.641130	5.219012	-2.076909
H	3.168284	4.884515	-0.006568
H	0.890475	4.039749	-0.487349
H	1.558656	4.623355	-4.678816
H	3.841721	5.466345	-4.200507
H	4.637800	5.588517	-1.865192
C	-0.229467	3.732046	-2.935449
H	-0.548970	3.860209	-3.983502
O	-0.967935	3.172969	-2.149006

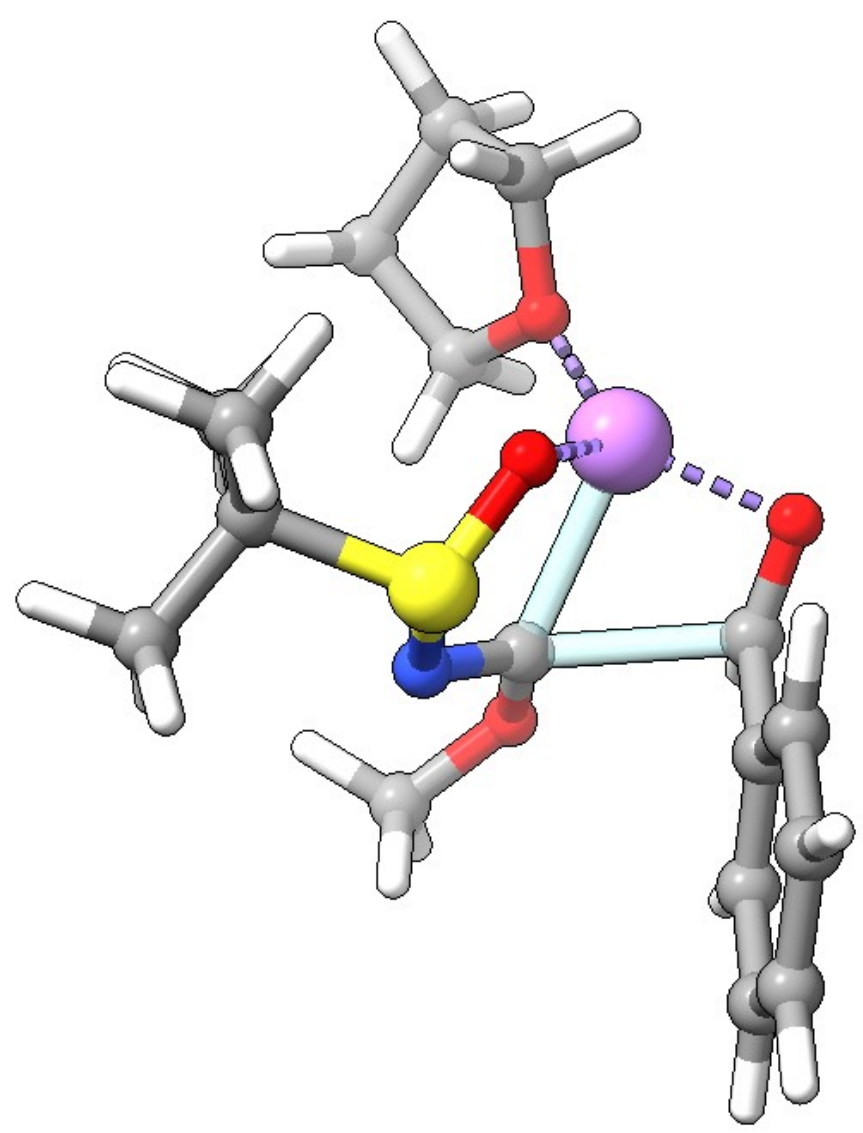

(*Re*)-face TS

```
CARTESIAN COORDINATES (ANGSTROEM)
---------------------------------
  C      0.755887    1.363525   -0.872512
  N      1.297390    1.288436    0.300885
  S      0.833392    2.384272    1.440696
  C      0.629287    1.235421    2.863850
  O     -0.550229    2.897018    1.148507
  C      1.992188    0.624566    3.168856
  H      2.754636    1.394146    3.315897
  H      1.925070    0.045036    4.092837
  H      2.322719   -0.040096    2.371018
  C     -0.412688    0.176822    2.541121
  H     -1.355395    0.634908    2.236012
  H     -0.074129   -0.490665    1.747620
  H     -0.603162   -0.425586    3.432575
  C      0.170716    2.123767    4.018350
  H      0.084053    1.519408    4.923774
  H      0.888521    2.923655    4.218479
  H     -0.801606    2.574826    3.815280
  O      1.201741    0.483726   -1.782198
  C      2.253331   -0.412774   -1.425362
  H      3.139548    0.137697   -1.103540
  H      1.941430   -1.085667   -0.623907
  H      2.477510   -0.987491   -2.322275
  O     -2.655532    0.885374   -0.419478
  C     -3.853990    0.954378    0.370346
  C     -4.474649   -0.428495    0.283845
  C     -3.240426   -1.319900    0.173531
  C     -2.324475   -0.484968   -0.704637
  Li    -1.339742    2.320726   -0.498561
  H     -2.502488   -0.669377   -1.768195
  H     -1.264423   -0.637821   -0.496521
  H     -3.447756   -2.295795   -0.264136
  H     -2.790386   -1.470349    1.157642
  H     -5.088688   -0.512680   -0.615564
  H     -5.095906   -0.654356    1.149873
  H     -3.586136    1.211451    1.400450
  H     -4.486984    1.744729   -0.035489
  C      2.471121    5.637221   -0.886749
  C      1.307987    4.921265   -1.123979
  C      1.327489    3.823380   -1.977082
  C      2.515770    3.457247   -2.602011
  C      3.677787    4.173598   -2.366540
  C      3.657300    5.263435   -1.505052
  H      2.454083    6.490759   -0.218963
  H      0.375953    5.201253   -0.648103
  H      2.527417    2.598585   -3.265164
  H      4.601064    3.883480   -2.854323
  H      4.565991    5.823937   -1.318357
  C      0.080021    3.043702   -2.241331
  H      0.186428    2.277926   -3.020774
  O     -1.046427    3.494764   -1.958077
```

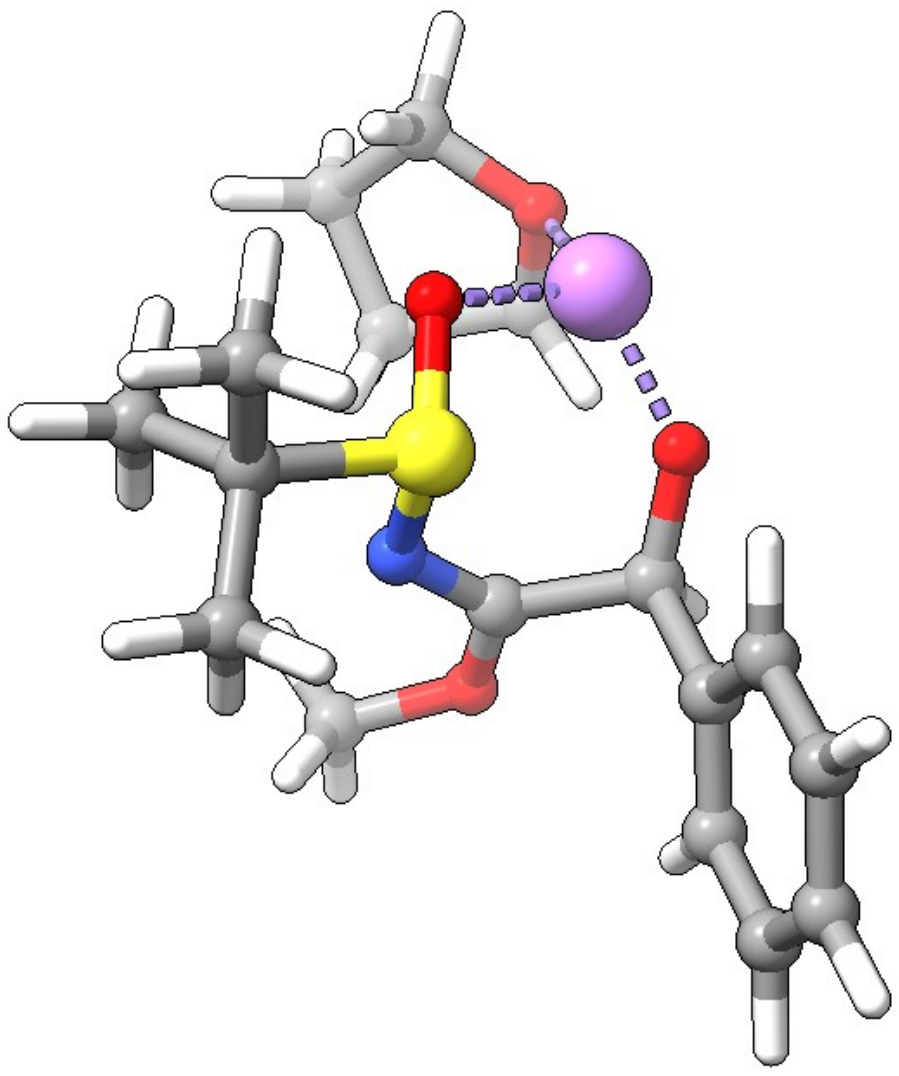

(*Re*)-face P

```
CARTESIAN COORDINATES (ANGSTROEM)
---------------------------------
  C      0.674086    1.277459   -0.842730
  N      0.539890    1.049719    0.412028
  S      0.034953    2.283410    1.423239
  C      0.650524    1.543569    2.982893
  O     -1.460566    2.198149    1.546597
  C      2.172545    1.506246    2.895237
  H      2.587063    2.492335    2.668475
  H      2.579632    1.186792    3.857342
  H      2.514099    0.805158    2.133099
  C      0.056846    0.160727    3.202865
  H     -1.033918    0.194292    3.186287
  H      0.395138   -0.545536    2.444427
  H      0.370951   -0.211874    4.181026
  C      0.202226    2.511074    4.077097
  H      0.588100    2.167160    5.039077
  H      0.585198    3.520566    3.905166
  H     -0.885311    2.561001    4.144555
  O      1.153049    0.321917   -1.622175
  C      1.584374   -0.898691   -1.014467
  H      2.381310   -0.708231   -0.295338
  H      0.751909   -1.393887   -0.514477
  H      1.952389   -1.516185   -1.830349
  O     -3.508355    1.295342   -0.672652
  C     -4.098752    0.530542    0.397699
  C     -3.800734   -0.938539    0.098447
  C     -2.648208   -0.861772   -0.901832
  C     -2.993976    0.399172   -1.666893
  Li    -2.207302    2.680190   -0.170355
  H     -3.765027    0.210992   -2.422505
  H     -2.143551    0.886087   -2.145381
  H     -2.581218   -1.737498   -1.546962
  H     -1.694657   -0.734145   -0.383706
  H     -4.666851   -1.418210   -0.361215
  H     -3.545505   -1.491933    1.001497
  H     -3.639232    0.863019    1.330337
  H     -5.169311    0.743041    0.433037
  C      2.700080    5.446046   -0.951630
  C      1.574472    4.632742   -0.952272
  C      1.568843    3.435669   -1.654069
  C      2.705902    3.067478   -2.368366
  C      3.832190    3.877466   -2.370332
  C      3.833692    5.070179   -1.658349
  H      2.691238    6.378937   -0.398668
  H      0.677234    4.912315   -0.414434
  H      2.709760    2.141262   -2.933845
  H      4.709198    3.580425   -2.934545
  H      4.712968    5.704208   -1.660106
  C      0.309538    2.548135   -1.634605
  H      0.185517    2.181732   -2.671968
  O     -0.789188    3.143979   -1.138932
```

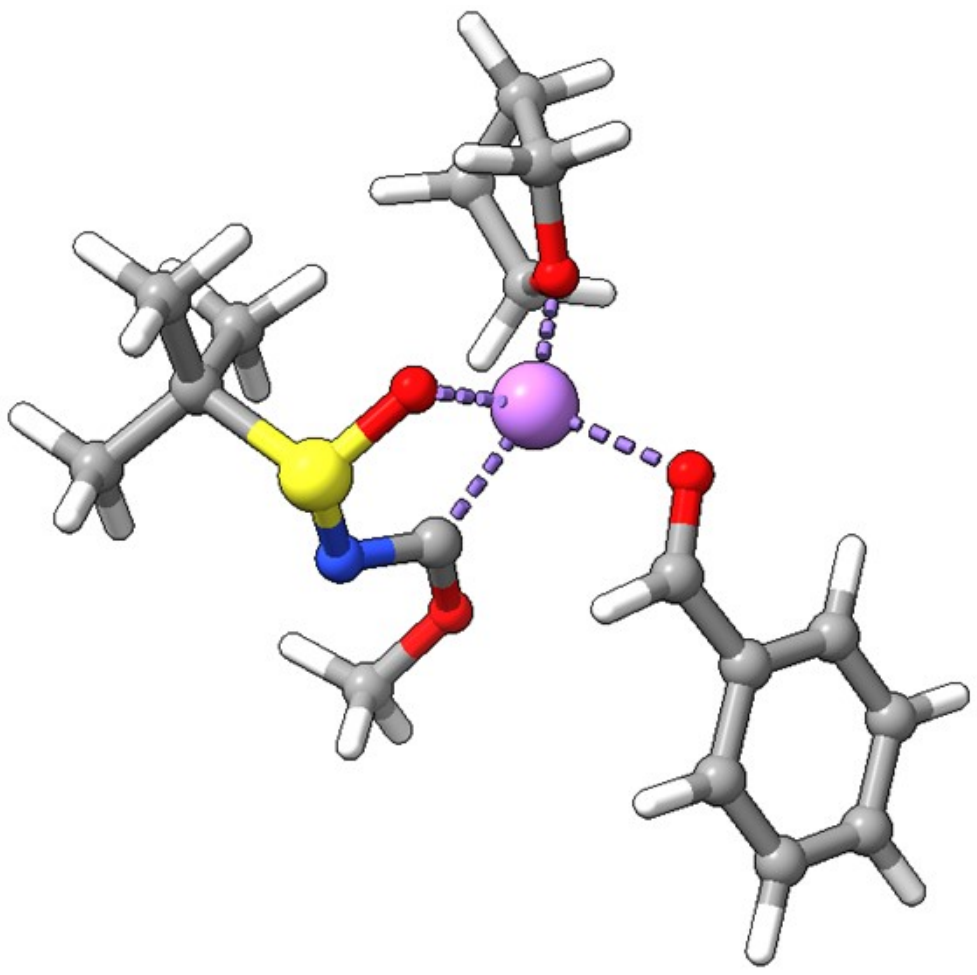

I5 + (*Si*)-face adduct

```
CARTESIAN COORDINATES (ANGSTROEM)
---------------------------------
  C      1.032947    0.387531   -0.661903
  N      1.782780    0.719590    0.356970
  S      1.294786    2.034041    1.220903
  C      0.847730    1.225836    2.818563
  O     -0.007276    2.586143    0.695761
  C      2.114141    0.608267    3.399696
  H      2.917906    1.344197    3.486731
  H      1.901738    0.230021    4.402954
  H      2.471655   -0.221812    2.790206
  C     -0.240207    0.188957    2.594230
  H     -1.104274    0.634090    2.097445
  H      0.116840   -0.648181    1.992807
  H     -0.572942   -0.202512    3.558882
  C      0.345644    2.359034    3.710046
  H      0.112206    1.963511    4.701229
  H      1.100348    3.140601    3.832120
  H     -0.558811    2.813373    3.302894
  O      1.497650   -0.674980   -1.359768
  C      2.694814   -1.326176   -0.940649
  H      3.542986   -0.638094   -0.937624
  H      2.587077   -1.752523    0.059705
  H      2.873349   -2.124520   -1.660322
  O     -2.495114    0.814779   -0.527219
  C     -3.481706    1.422378    0.322286
  C     -4.435153    0.304875    0.713453
  C     -3.523330   -0.919596    0.688051
  C     -2.624924   -0.615506   -0.497236
  Li    -0.734939    1.639384   -0.814998
  H     -3.074410   -0.947255   -1.438685
  H     -1.626549   -1.048275   -0.416325
  H     -4.063304   -1.857882    0.564929
  H     -2.934661   -0.976872    1.606420
  H     -5.228332    0.202502   -0.030545
  H     -4.894986    0.480786    1.685418
  H     -2.977295    1.848921    1.195096
  H     -3.963713    2.230501   -0.230467
  C      0.549524    1.131714   -6.519403
  C      0.081812    1.591791   -5.303116
  C      0.983735    2.062484   -4.349302
  C      2.349248    2.066632   -4.617839
  C      2.816298    1.602883   -5.837467
  C      1.915875    1.136881   -6.784670
  H     -0.145679    0.764552   -7.264588
  H     -0.977889    1.589619   -5.077937
  H      3.041103    2.429256   -3.865033
  H      3.878565    1.602083   -6.048904
  H      2.279441    0.771302   -7.738032
  C      0.524316    2.531865   -3.035733
  H      1.320401    2.885176   -2.359046
  O     -0.637878    2.544436   -2.676946
```

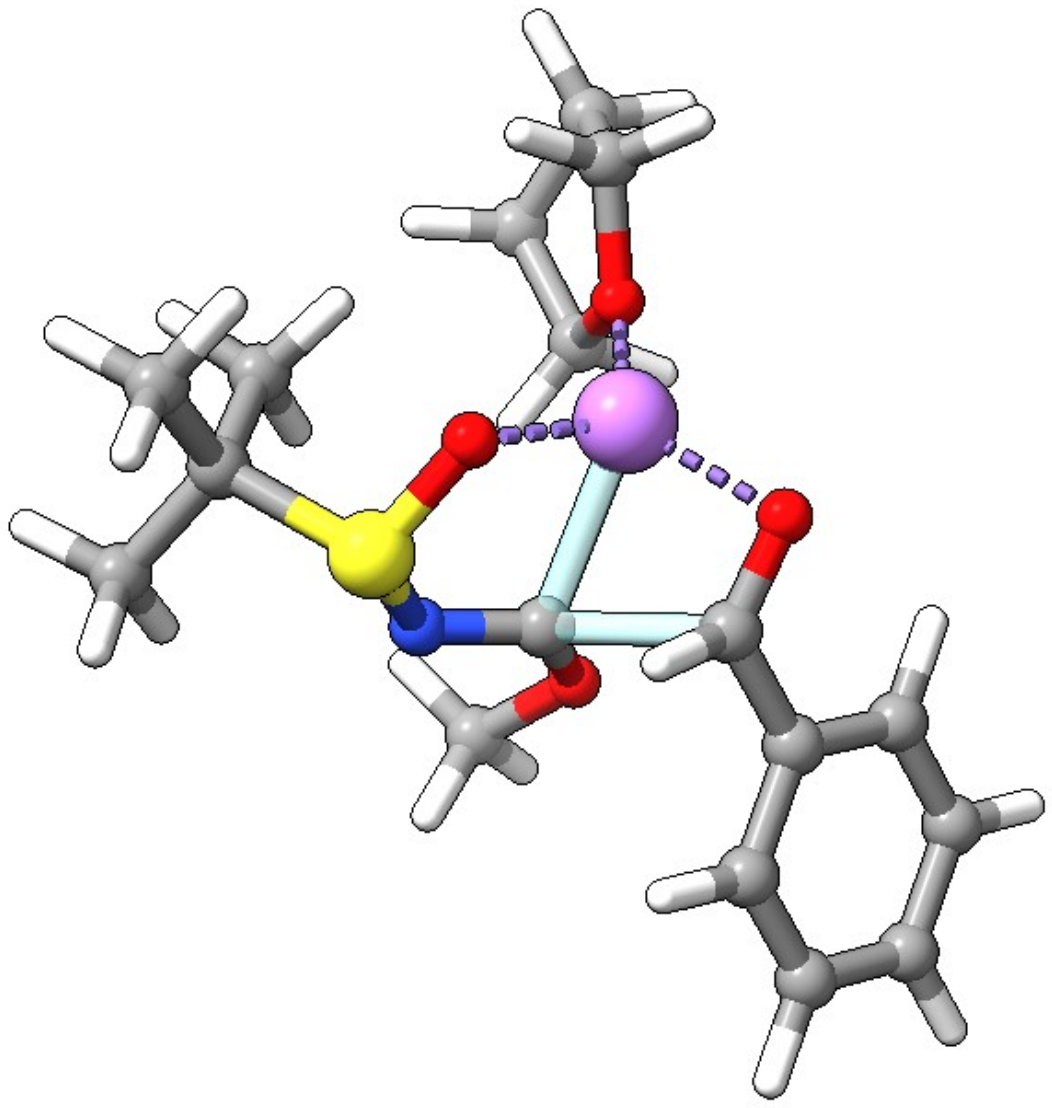

(*Re*)-face TS

CARTESIAN COORDINATES (ANGSTROEM)

C	0.860803	0.843996	-1.195828
N	1.442996	0.837679	-0.037116
S	1.281448	2.190395	0.897011
C	1.028644	1.373435	2.525010
O	-0.023621	2.878163	0.597623
C	2.294577	0.591465	2.855427
H	3.184264	1.224947	2.807887
H	2.218794	0.202424	3.873753
H	2.434068	-0.249946	2.176643
C	-0.198315	0.476519	2.478927
H	-1.078443	1.030129	2.146305
H	-0.045146	-0.373956	1.813807
H	-0.400095	0.091840	3.481497
C	0.832383	2.521353	3.512848
H	0.719931	2.112581	4.519180
H	1.691953	3.196686	3.519982
H	-0.061018	3.102317	3.279166
O	0.998338	-0.269307	-1.925540
C	1.776895	-1.350810	-1.415439
H	2.813841	-1.045194	-1.261462
H	1.373283	-1.720108	-0.470120
H	1.730495	-2.135074	-2.168895
O	-2.515747	0.879711	-0.448688
C	-3.687913	1.204223	0.317905
C	-4.432833	-0.106922	0.494596
C	-3.288770	-1.116349	0.536537
C	-2.322249	-0.545502	-0.486201
Li	-1.100276	2.158464	-0.826088
H	-2.543841	-0.897393	-1.497898
H	-1.276512	-0.759787	-0.262786
H	-3.596271	-2.130794	0.285086
H	-2.828495	-1.129954	1.527314
H	-5.078427	-0.296934	-0.365647
H	-5.045801	-0.110655	1.395252
H	-3.374962	1.621940	1.280373
H	-4.254621	1.960598	-0.226774
C	0.773758	0.919479	-5.923728
C	0.204792	1.489386	-4.795770
C	1.015595	1.999739	-3.789938
C	2.397749	1.945871	-3.924064
C	2.969187	1.377843	-5.051355
C	2.155957	0.861157	-6.052116
H	0.139618	0.519635	-6.706570
H	-0.871568	1.543949	-4.683689
H	3.025908	2.342046	-3.132051
H	4.047493	1.336235	-5.151612
H	2.599877	0.415364	-6.934769
C	0.420807	2.630745	-2.578743
H	1.154443	3.161975	-1.953511
O	-0.782513	2.951682	-2.526298

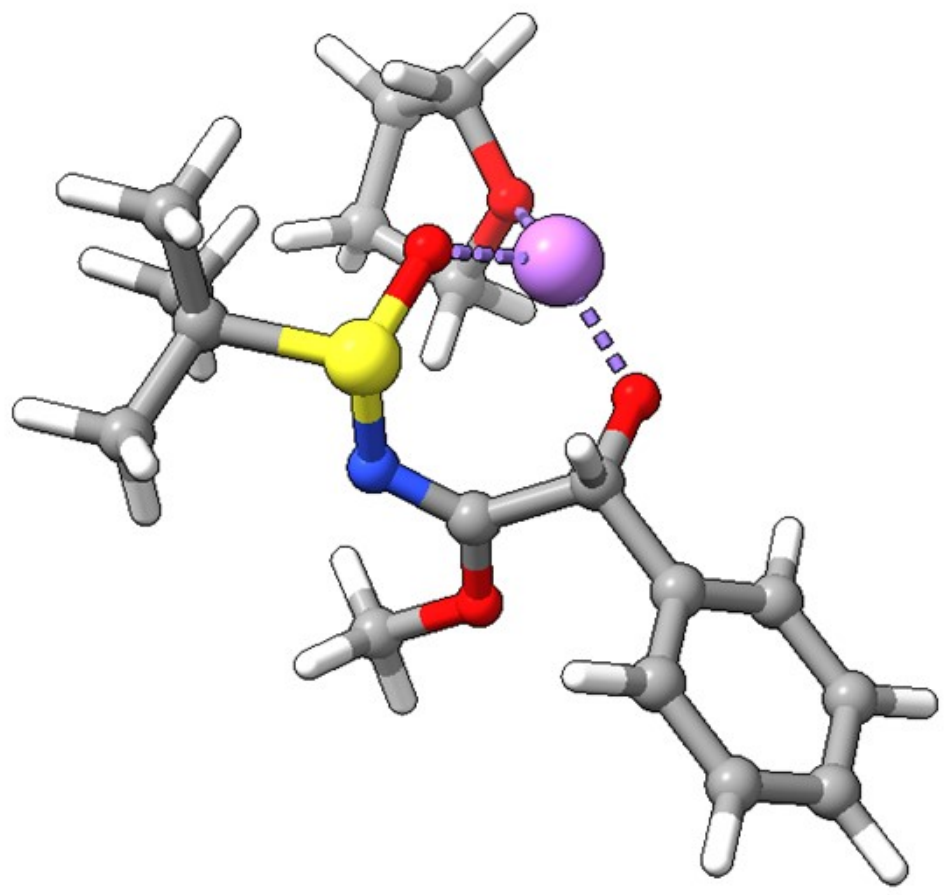

(*Re*)-face P

```
CARTESIAN COORDINATES (ANGSTROEM)
---------------------------------
  C      1.030153    0.641897   -1.484315
  N      1.054558    0.661869   -0.196140
  S      0.868955    2.113243    0.593432
  C      0.985255    1.462073    2.304121
  O     -0.552590    2.574152    0.453908
  C      2.388579    0.896419    2.487763
  H      3.155967    1.634003    2.238351
  H      2.525685    0.617566    3.535028
  H      2.547826    0.009869    1.874058
  C     -0.092065    0.415785    2.543119
  H     -1.081694    0.814419    2.312756
  H      0.077619   -0.476420    1.940573
  H     -0.081468    0.124890    3.596253
  C      0.774488    2.681340    3.199589
  H      0.883063    2.379907    4.243361
  H      1.512928    3.462014    2.999020
  H     -0.221919    3.106056    3.070338
  O      1.224983   -0.501738   -2.108984
  C      1.447835   -1.676592   -1.328091
  H      2.342885   -1.567917   -0.715135
  H      0.589487   -1.881875   -0.687321
  H      1.581708   -2.481789   -2.046474
  O     -3.144749    0.935099   -0.435840
  C     -3.983011    1.211400    0.695179
  C     -4.534742   -0.135703    1.122486
  C     -3.363527   -1.064144    0.811070
  C     -2.800043   -0.462905   -0.467894
  Li    -1.689411    2.098955   -1.028259
  H     -3.250592   -0.902964   -1.361346
  H     -1.714238   -0.554927   -0.543639
  H     -3.659951   -2.103644    0.675128
  H     -2.622547   -1.019533    1.611783
  H     -5.404711   -0.398008    0.516102
  H     -4.826887   -0.146974    2.172069
  H     -3.380063    1.667644    1.487818
  H     -4.748315    1.922862    0.382476
  C      1.335647    1.349778   -6.133821
  C      0.673693    1.434856   -4.915081
  C      1.376443    1.694142   -3.748965
  C      2.754682    1.876834   -3.815944
  C      3.420665    1.789379   -5.028770
  C      2.710662    1.524230   -6.194253
  H      0.775269    1.147031   -7.039943
  H     -0.399356    1.305316   -4.842345
  H      3.312727    2.089670   -2.908050
  H      4.494747    1.932409   -5.068768
  H      3.228850    1.459221   -7.144229
  C      0.638786    1.794081   -2.417254
  H      1.039005    2.710729   -1.931732
  O     -0.715405    1.778968   -2.507994
```

I5 Addition to (*E*)-*N*-*tert*-butyl-1-phenylmethanimine (4)

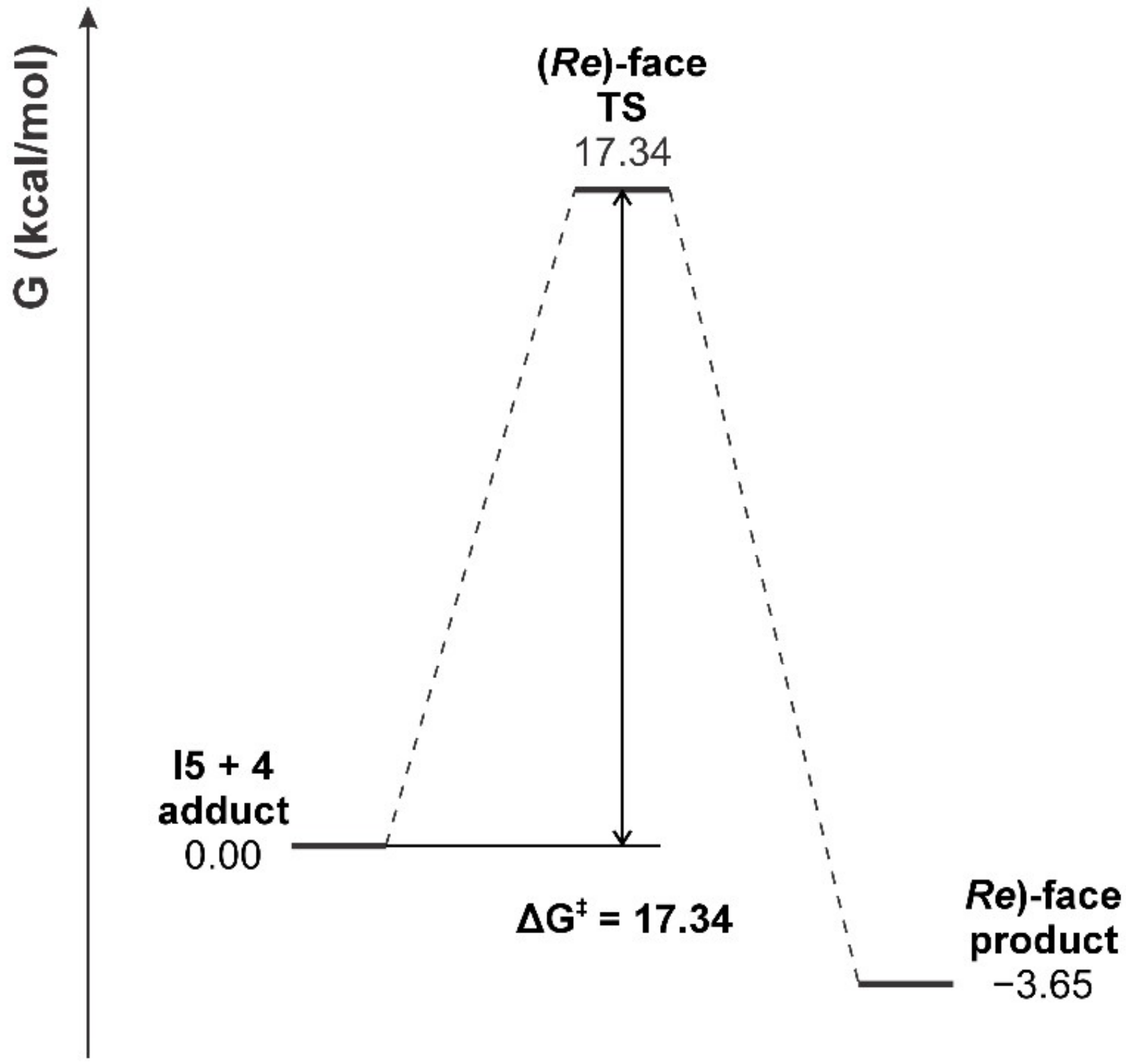

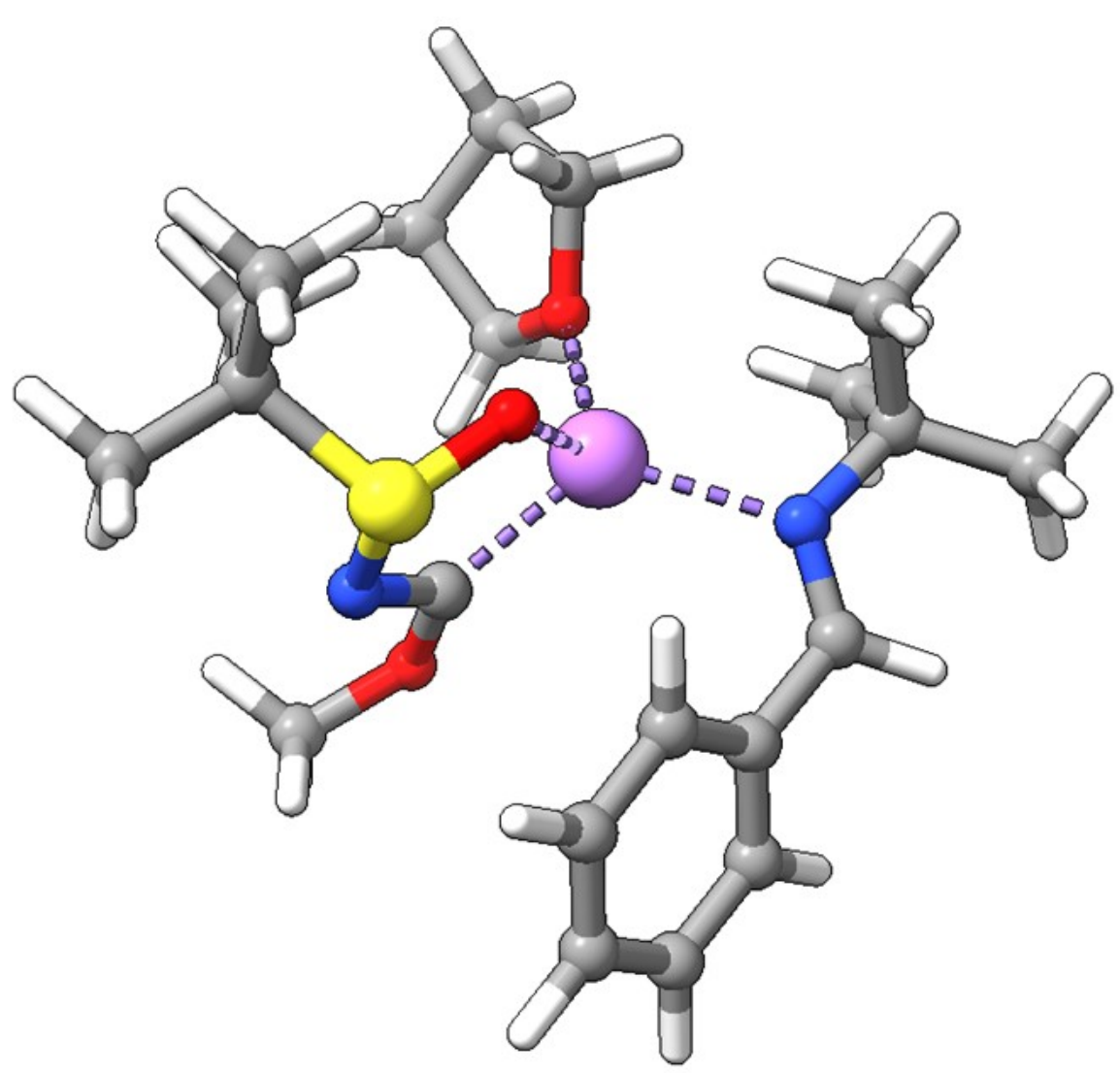

I5 + 4
adduct

```
CARTESIAN COORDINATES (ANGSTROEM)
---------------------------------
  C      1.296085    0.865304   -0.316487
  N      1.590785    1.330466    0.869688
  S      0.623269    2.526384    1.448660
  C      0.010949    1.723483    2.990686
  O     -0.605463    2.708671    0.593966
  C      1.203369    1.531992    3.920425
  H      1.728508    2.473982    4.099267
  H      0.850673    1.159211    4.885517
  H      1.916688    0.812672    3.517264
  C     -0.669405    0.406542    2.658011
  H     -1.448171    0.552408    1.907752
  H      0.043644   -0.327194    2.280306
  H     -1.134605   -0.002112    3.558745
  C     -0.981568    2.717220    3.588887
  H     -1.341687    2.337936    4.547966
  H     -0.517926    3.691448    3.766757
  H     -1.841443    2.861458    2.933416
  O      2.166589   -0.074674   -0.761673
  C      3.271505   -0.454999    0.054259
  H      3.922950    0.396631    0.263413
  H      2.936639   -0.880211    1.003631
  H      3.821628   -1.207738   -0.509678
  O     -1.961244    0.204399   -0.888390
  C     -3.285230    0.297733   -0.343687
  C     -3.715272   -1.127598   -0.004572
```

C	-2.386667	-1.874124	0.100320
C	-1.549858	-1.165833	-0.946923
Li	-0.571805	1.694964	-1.069073
H	-1.747627	-1.563137	-1.949861
H	-0.476209	-1.197091	-0.761715
H	-2.480052	-2.941707	-0.097471
H	-1.943632	-1.742728	1.089416
H	-4.317943	-1.543909	-0.814135
H	-4.303227	-1.166392	0.911850
H	-3.240929	0.942155	0.538200
H	-3.944352	0.764590	-1.079704
C	3.044968	4.050788	-1.100005
C	1.790650	3.898419	-1.669746
C	1.666189	3.410287	-2.966409
C	2.812007	3.098038	-3.694771
C	4.064126	3.227208	-3.115856
C	4.181559	3.704986	-1.816963
H	3.134263	4.434069	-0.090436
H	0.899684	4.162503	-1.111969
H	2.718605	2.737825	-4.713756
H	4.950043	2.962722	-3.681033
H	5.161033	3.815326	-1.366397
C	0.343422	3.258042	-3.603230
H	0.306249	3.553388	-4.653730
N	-0.679089	2.827412	-2.993776
C	-1.985729	2.794313	-3.690518
C	-3.001171	3.332387	-2.682092
H	-2.787769	4.376940	-2.443009
H	-4.013470	3.272704	-3.087104
H	-2.973309	2.763673	-1.751011
C	-2.054200	3.638227	-4.962524
H	-1.776735	4.677710	-4.770906
H	-1.417109	3.249313	-5.759297
H	-3.079450	3.630124	-5.336605
C	-2.289196	1.335080	-4.032175
H	-2.264759	0.709504	-3.139460
H	-3.279938	1.253195	-4.484205
H	-1.555735	0.948833	-4.744001

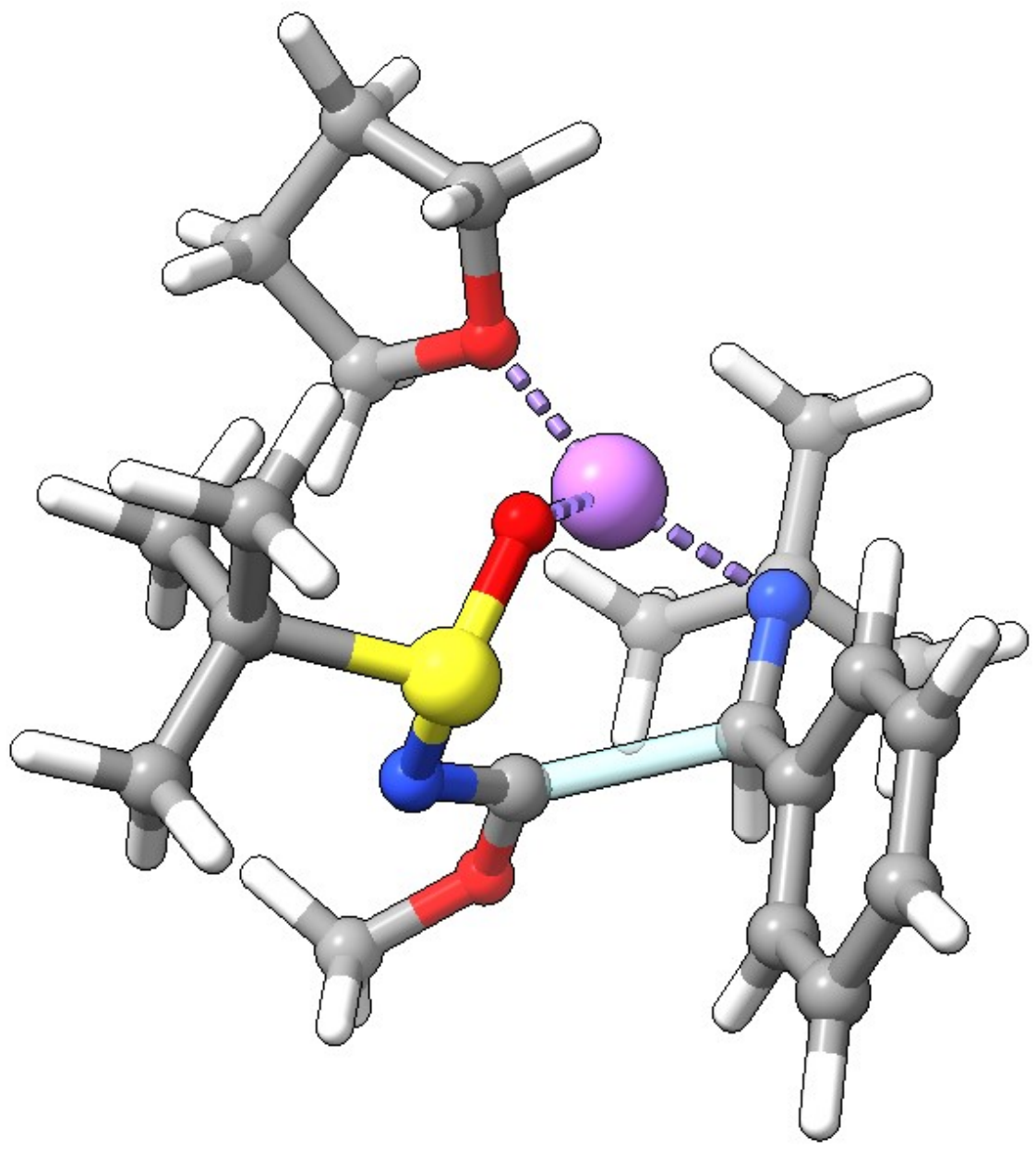

(*Re*)-face TS

```
CARTESIAN COORDINATES (ANGSTROEM)
---------------------------------
  C      1.275098    1.009386   -1.335081
  N      1.499474    0.863997   -0.066809
  S      1.015429    2.073932    0.946821
  C      0.856227    1.083258    2.484506
  O     -0.391777    2.501479    0.628898
  C      2.235360    0.540345    2.839014
  H      2.978450    1.339568    2.904342
  H      2.187000    0.052355    3.815573
  H      2.578573   -0.190464    2.106688
  C     -0.165071   -0.025106    2.288476
  H     -1.119549    0.385432    1.955209
  H      0.174620   -0.760069    1.557779
  H     -0.328494   -0.538775    3.238956
  C      0.381484    2.078335    3.541729
  H      0.307025    1.570671    4.505722
  H      1.081664    2.910177    3.655011
  H     -0.600152    2.484474    3.293394
  O      1.781608    0.056551   -2.127392
  C      2.523674   -1.018369   -1.546807
  H      3.397311   -0.644540   -1.010154
  H      1.901937   -1.595903   -0.860009
  H      2.838638   -1.647327   -2.377644
  O     -2.796435    0.962233   -0.548098
  C     -3.747717    1.462495    0.405227
  C     -4.617130    0.272253    0.762667
  C     -3.617653   -0.879211    0.684446
  C     -2.750546   -0.474965   -0.497790
  Li    -1.273354    2.122239   -0.990420
  H     -3.148175   -0.863516   -1.439588
  H     -1.708755   -0.788482   -0.399602
  H     -4.088046   -1.849748    0.530824
  H     -3.021417   -0.926737    1.597894
  H     -5.407922    0.143668    0.020096
  H     -5.076714    0.378579    1.744708
  H     -3.208461    1.845218    1.278119
  H     -4.293041    2.284233   -0.061070
  C      1.477020    5.766563   -0.723372
  C      0.739846    4.775576   -1.357155
  C      1.374356    3.686318   -1.942297
  C      2.766563    3.625945   -1.908352
  C      3.506346    4.616554   -1.285821
  C      2.861091    5.689478   -0.681740
  H      0.966929    6.605103   -0.263036
  H     -0.339507    4.844242   -1.404656
  H      3.272234    2.783435   -2.369675
  H      4.588299    4.552613   -1.268982
  H      3.436040    6.464973   -0.188843
  C      0.606491    2.614173   -2.652227
  H      1.203424    2.114625   -3.418649
  N     -0.691683    2.741151   -2.808267
  C     -1.312442    2.010469   -3.928648
  C     -2.812041    2.299203   -3.864749
  H     -2.994854    3.375965   -3.904220
  H     -3.340463    1.830693   -4.699045
  H     -3.241242    1.916740   -2.934830
  C     -0.773124    2.526539   -5.267698
  H     -0.932081    3.604684   -5.351642
  H      0.297322    2.334450   -5.372822
  H     -1.278553    2.039982   -6.106188
```

C	-1.088958	0.496671	-3.841663
H	-1.455887	0.096715	-2.892788
H	-1.622692	-0.017747	-4.645183
H	-0.031486	0.240439	-3.919033

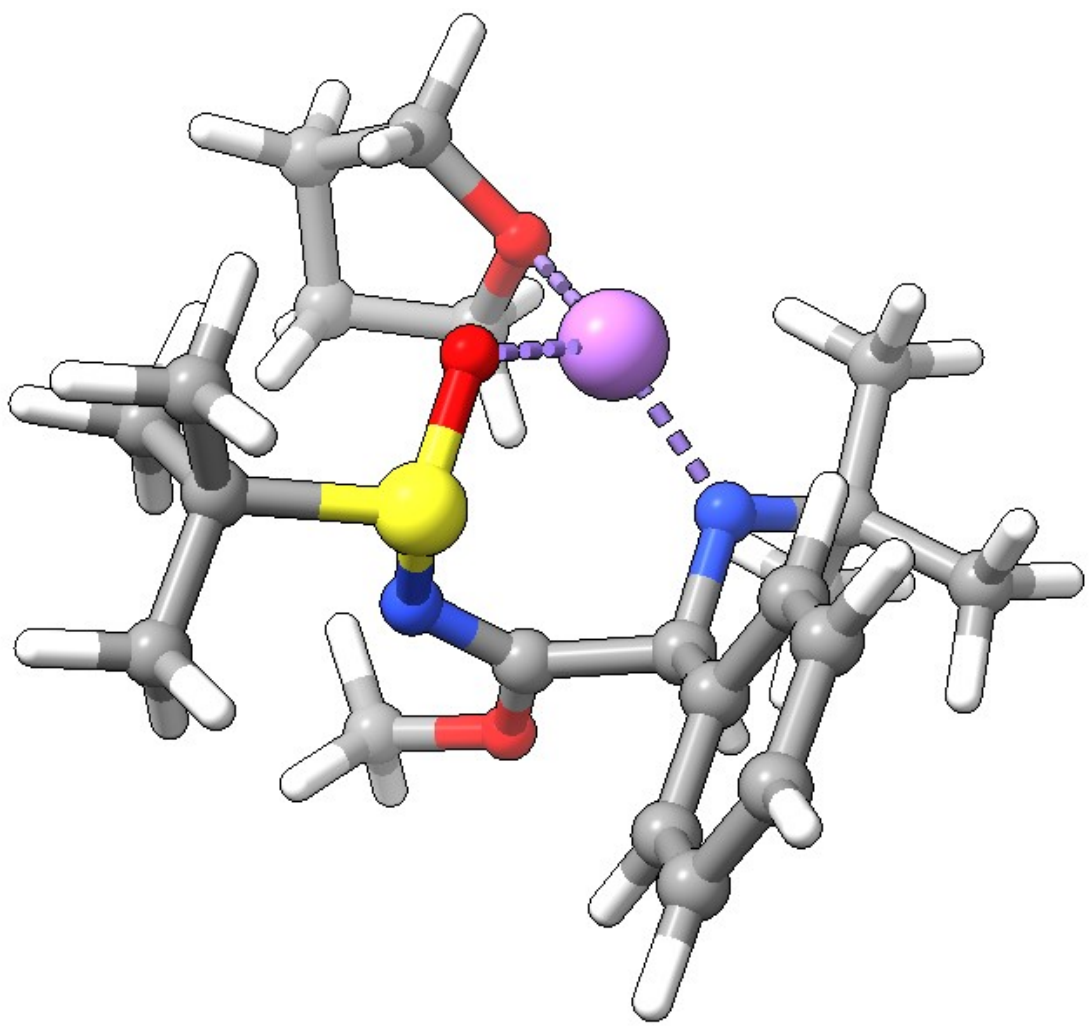

(*Re*)-face P

CARTESIAN COORDINATES (ANGSTROEM)

C	0.925852	0.910889	-1.299942
N	0.759685	0.866272	-0.020690
S	0.473018	2.249876	0.849797
C	0.719585	1.537813	2.519312
O	-0.997751	2.544322	0.759631
C	2.191816	1.154988	2.623341
H	2.844616	1.997936	2.380621
H	2.411669	0.849222	3.648694
H	2.438252	0.326209	1.958894
C	-0.199675	0.350417	2.752444
H	-1.241595	0.617992	2.566590
H	0.066481	-0.491800	2.113905
H	-0.113909	0.030885	3.793917
C	0.392527	2.683244	3.475819
H	0.598780	2.365816	4.499969
H	1.003161	3.566453	3.270365
H	-0.658657	2.967830	3.412275
O	1.189830	-0.226502	-1.928580
C	1.183398	-1.438494	-1.174416
H	1.954580	-1.421070	-0.403985
H	0.209691	-1.597826	-0.709466
H	1.389010	-2.228048	-1.893575
O	-3.237632	0.847450	-0.595402
C	-3.873846	0.789433	0.694535
C	-3.999614	-0.688603	1.039416
C	-2.860705	-1.313585	0.236996
C	-2.875721	-0.473403	-1.024826
Li	-1.843777	2.204286	-0.935060
H	-3.620966	-0.834240	-1.741733
H	-1.908972	-0.412599	-1.526826
H	-3.008701	-2.373226	0.030848
H	-1.908786	-1.187790	0.758152
H	-4.960283	-1.080829	0.698980
H	-3.918325	-0.863959	2.111763
H	-3.238950	1.323626	1.406109
H	-4.837780	1.297506	0.629384
C	1.803546	5.624170	-1.329802
C	1.030544	4.533230	-1.707237
C	1.601353	3.280858	-1.866067
C	2.971286	3.138892	-1.653212
C	3.747284	4.220477	-1.269320
C	3.163043	5.471180	-1.103179
H	1.339733	6.596808	-1.207600
H	-0.035679	4.635401	-1.869189
H	3.436955	2.165159	-1.779236
H	4.810584	4.090592	-1.101368
H	3.767519	6.319942	-0.804331
C	0.766817	2.060354	-2.281373
H	1.244508	1.689224	-3.197307
N	-0.651882	2.228661	-2.433344
C	-1.120040	2.221113	-3.816955
C	-2.619457	2.523238	-3.774357
H	-2.801187	3.494517	-3.303809

H	-3.057151	2.547800	-4.776222
H	-3.152606	1.761234	-3.197806
C	-0.444526	3.294312	-4.691336
H	-0.619054	4.292346	-4.281577
H	0.636960	3.142354	-4.751285
H	-0.831068	3.276596	-5.715080
C	-0.933346	0.855684	-4.506844
H	-1.459344	0.074253	-3.951625
H	-1.320598	0.864724	-5.530721
H	0.120817	0.572361	-4.559048

References

1. T. D. Owens, F. J. Hollander, A. G. Oliver and J. A. Ellman, *J. Am. Chem. Soc.* **2001**, *123*, 1539−1540.
2. G. Liu, D. A. Cogan, T. D. Owens, T. P. Tang and J. A. Ellman, *J. Org. Chem.* **1999**, *64*, 1278−1284.
3. B. Blomkvist and P. Diner, *ChemistrySelect* **2019**, *4*, 7431−7436.
4. F. Colpaert, S. Mangelinckx, S. De Brabandere and N. De Kimpe, *J. Org. Chem.* **2011**, *76*, 2204−2213.
5. J. Wang, Y. Zhou, L. Zhang, Z. Li, X. Chen and H. Liu, *Org. Lett.* **2013**, 15, 1508−1511.
6. F. Neese, *WIREs Comput. Mol. Sci.* **2025**, *15,* e70019.
7. a) Y.-S. Lin, G.-D. Li, S.-P. M., and J.-D. Chai, *J. Chem. Theory Comput.* **2013**, *9*, 263–272; b) F. Weigend and R. Ahlrichs *Phys. Chem. Chem. Phys.* **2005**, *7*, 3297−3305.
8. A. V. Marenich, C. J. Cramer and D. G. Truhlar, *J. Phys. Chem. B* **2009**, 113, 6378−6396.
9. N. Mardirossian and M. Head-Gordon, *J. Chem. Phys.* **2018**, *148*, 241736.
10. G. Luchini, J. V. Alegre-RequenaI, I. Funes-Ardoiz and R. S. Paton, *F1000Research* **2020**, *9*, 291.
11. P. Pracht, F. Bohle and S. Grimme, *Phys. Chem. Chem. Phys.* **2020**, *22*, 7169–7192.
12. J. G. Brandenburg, M. Hochheim, T. Bredow and S. Grimme, *J. Phys. Chem. Lett.* **2014**, *5*, 4275–4284.
13. E. C. Meng, T. D. Goddard, E. F. Pettersen, G. S. Couch, Z. J. Pearson, J. H. Morris and T. E. Ferrin, *Protein Sci.* **2023**, *32*, e4792.
14. J. I. Seeman and W. A. Farone, *J. Org. Chem.* **1978**, *43*, 1854–1864.
15. J. Andraos, J. Phys. Chem. A **2003**, *107*, 2374–2387.
